# Exosomal export of activated CASPASE-8 is linked to the prevention of TNF-induced cytotoxicity

**DOI:** 10.1038/s41467-026-75544-1

**Published:** 2026-08-25

**Authors:** Jon Huyghe, Dario Priem, Annelore Haems, Lien Lippens, Margaux De Meyer, Tom Delanghe, Yves Dondelinger, Inge Bruggeman, Peter Vandenabeele, Sven Eyckerman, An Hendrix, Mathieu JM Bertrand

**Affiliations:** 1 VIB Center for Inflammation Research, Ghent, Belgium; 2 Department of Biomedical Molecular Biology, Ghent University, Ghent, Belgium; 3 Laboratory of Experimental Cancer Research, Department of Human Structure and Repair, Ghent University, Ghent, Belgium; 4 Cancer Research Institute Ghent, Ghent, Belgium; 5 VIB-UGent Center for Medical Biotechnology, Ghent, Belgium; 6 Department of Biomolecular Medicine, Ghent University, Ghent, Belgium

**Keywords:** Cell death and immune response, Autophagy, Cell signalling

## Abstract

Tumor Necrosis Factor (TNF) is a key pro-inflammatory cytokine whose sensing by TNFR1 triggers gene activation or cell death induction. While TNF cytotoxicity can be beneficial during infections by supporting effective immune responses, its chronic or excessive induction is harmful and promotes inflammatory pathologies. Protective brakes, known as cell death checkpoints, normally repress TNF cytotoxicity and therefore constitute crucial safeguards against these diseases. Death by TNF mainly proceeds upon inactivation of a checkpoint by microbial effector proteins or pathological mutations. We previously identified lysosomal turnover of TNFR1 Complex II by TAX1BP1-mediated selective macro-autophagy as a brake on TNF cytotoxicity. Here, we propose an alternative mechanism that prevents TNF-induced RIPK1 kinase-independent apoptosis. We found that inhibiting the ESCRT machinery, HSC70 or TAX1BP1 interferes with the TNF-dependent targeting of activated CASPASE-8 into endosomal intralumenal vesicles (ILVs) and is associated with apoptosis induction. Furthermore, we identified TAX1BP1 and TNFR1 Complex II components as TNF-induced cargoes of extracellular vesicles, suggesting that exosomal release of TNFR1 Complex II serves as a parallel detoxification pathway to lysosomal turnover. Finally, we show that *Salmonella* Typhimurium and *Mycobacterium tuberculosis* effector proteins activate TNF cytotoxicity by inhibiting components of the ESCRT machinery involved in this detoxification process.

## Abstract (web summary)

The ESCRT machinery and the chaperone HSC70 package activated CASP8 into intralumenal vesicles preventing TNF-induced apoptosis. Microbial effector proteins interfere with this detoxification mechanism and sensitize cells to TNF cytotoxicity.

## Introduction

The cytokine tumor necrosis factor (TNF) plays a major role in orchestrating inflammatory responses that are initiated when the body is threatened by invading microbes or following tissue injuries. The protective properties of TNF are, however, counterbalanced by its role in the pathogenesis of multiple inflammatory disorders, highlighting the importance of the tight regulation of signaling pathways downstream of TNF^1^. The detrimental role of TNF in disease was long considered to result from its ability to directly drive inflammation by activating the mitogen-activated protein kinase (MAPK) and nuclear factor-kappa B (NF-κB) signaling pathways. It is now clear that TNF also indirectly promotes inflammation by triggering cell death, in the form of apoptosis, pyroptosis or necroptosis^1^. Death by TNF was reported to be beneficial in the context of microbial infection, by eliminating infected cells and by releasing intracellular factors that ignite inflammatory signaling in neighboring cells. Consequently, TNF cytotoxicity turns into a highly detrimental response when induced aberrantly or in excess, as it promotes an uncontrolled amplifying loop of inflammation that drives disease development^2,3^. Therefore, protective brakes, known as cell death checkpoints, normally inhibit TNF cytotoxicity and thereby protect the organism from its potentially detrimental consequences, a situation reverted by pathological mutations in genes encoding components of the cell death checkpoints^4^. During infection, death by TNF is instead transiently induced by the actions of microbial effector proteins that inadvertently inactivate the cell death checkpoints in an attempt to interfere with the host’s immune responses^4^. TNF-induced cell death was consequently postulated to have evolved as a backup mechanism from the host to ensure proper anti-microbial responses during immune hijacking^5^.

The sensing of TNF by TNFR1 leads to the successive assembly of two protein complexes^4^. The receptor-bound TNFR1 Complex I forms within seconds of TNF sensing, and predominantly leads to pro-inflammatory gene activation. TNFR1 Complex I relies on the binding of RIPK1 and TRADD to the cytosolic portion of the receptor, allowing the subsequent recruitment of the E3 ubiquitin ligases cIAP1/2 and LUBAC (composed of HOIP, HOIL-1, and SHARPIN) that respectively decorate TNFR1 Complex I components with K63- and linear (M1)-ubiquitin chains^6,7^. This network of ubiquitin chains stabilizes the receptor complex and serves as a scaffold to recruit the kinases TAK1 and IKKα/β that activate the MAPK and NF-κB signaling pathways^8,9^. The proteins RIPK1 and TRADD subsequently dissociate from the receptor, inducing migration of the complex from the receptor to the cytosol, where it further recruits FADD and CASPASE-8 (CASP8) to become the cytotoxic TNFR1 Complex II^10^. This cytotoxic complex serves as an activating platform for CASP8, which can, in turn, process downstream effector caspases to trigger apoptosis or instead cleave GSDMD to induce pyroptosis^1^. Depending on the cellular context, the activation of RIPK1 enzymatic activity can promote CASP8 activation within TNFR1 Complex II and/or allow the further recruitment and activation of the kinase RIPK3 to trigger MLKL-mediated necroptosis^11^. These TNF-mediated cell death modalities are normally repressed in cells by the actions of several checkpoints reported to limit the assembly, stability and/or activity of TNFR1 Complex II^4^. For instance, it was recently found that an LC3-independent form of selective macro-autophagy prevents induction of TNF-mediated RIPK1 kinase-independent apoptosis by targeting TNFR1 Complex II for lysosomal degradation^12–14^. More precisely, it was proposed that the selective autophagy receptor TAX1BP1 binds to the M1-ubiquitin chains conjugated to TNFR1 Complex II and directly recruits the autophagy initiation machinery to promote the in situ formation of an autophagosome around the cytotoxic complex^12^.

Next to selective macro-autophagy, other autophagy pathways co-exist to degrade, sometimes the same, cytosolic cargo^15–19^. Chaperone-mediated autophagy (CMA) and micro-autophagy both deliver cargo to lysosomes independently of the core macro-autophagy machinery and without forming double-membrane autophagosomes^20–22^. In CMA, the chaperone HSC70 recognizes proteins containing a KFERQ pentapeptide motif and transports them to the lysosomal receptor LAMP2A, which assembles into a channel that translocates the unfolded cargo into the lysosomal lumen^19,23–25^. In micro-autophagy, cargo is instead taken up into intralumenal vesicles (ILVs) formed by the ESCRT-dependent invagination of the lysosomal membrane^21,22^. A similar ESCRT-dependent process can occur on endosomes, producing multivesicular bodies (MVBs). As MVBs mature, they may fuse with lysosomes to degrade their ILVs and associated cargoes in a pathway known as endosomal micro-autophagy (eMI)^26^ or instead fuse with the plasma membrane to release the ILVs into the extracellular environment as a type of extracellular vesicles (EVs) referred to as exosomes^27^. Unlike lysosomal micro-autophagy, cargo recognition during eMI also depends on HSC70^19,26^, and, in some cases, on selective-autophagy receptors^16,17^. Notably, the receptor TAX1BP1 was recently shown to promote degradation of ferritin via both LC3-independent selective macro-autophagy and HSC70-mediated eMI^15,18^. Building on our earlier identification of TAX1BP1-dependent lysosomal turnover of TNFR1 Complex II via LC3-independent selective macro-autophagy, we now investigated whether eMI, in parallel, might also contribute to the targeting of TNFR1 Complex II.

We show that TNF sensing triggers the ESCRT- and HSC70-dependent engulfment of activated CASP8 into endosomal ILVs. Surprisingly, we found that this sequestration does not contribute to its lysosomal degradation. Instead, we demonstrate that activated CASP8 and other components of TNFR1 Complex II are targeted for extracellular release as part of exosomes downstream of TNF sensing. Moreover, we show that the engulfment of activated CASP8 into ILVs relies on RIPK1, linear ubiquitination and TAX1BP1, which is reminiscent of the selective targeting of TNFR1 Complex II into autophagosomes during LC3-independent macro-autophagy^12^. Finally, we found that this detoxification mechanism is subject to inhibition by microbial effector proteins that interfere with the ESCRT machinery, sensitizing infected cells to TNF-induced cell death.

## Results

### The ESCRT machinery prevents TNF-induced RIPK1 kinase-independent apoptosis independently of the known cell death checkpoints

**Fig. 1:**
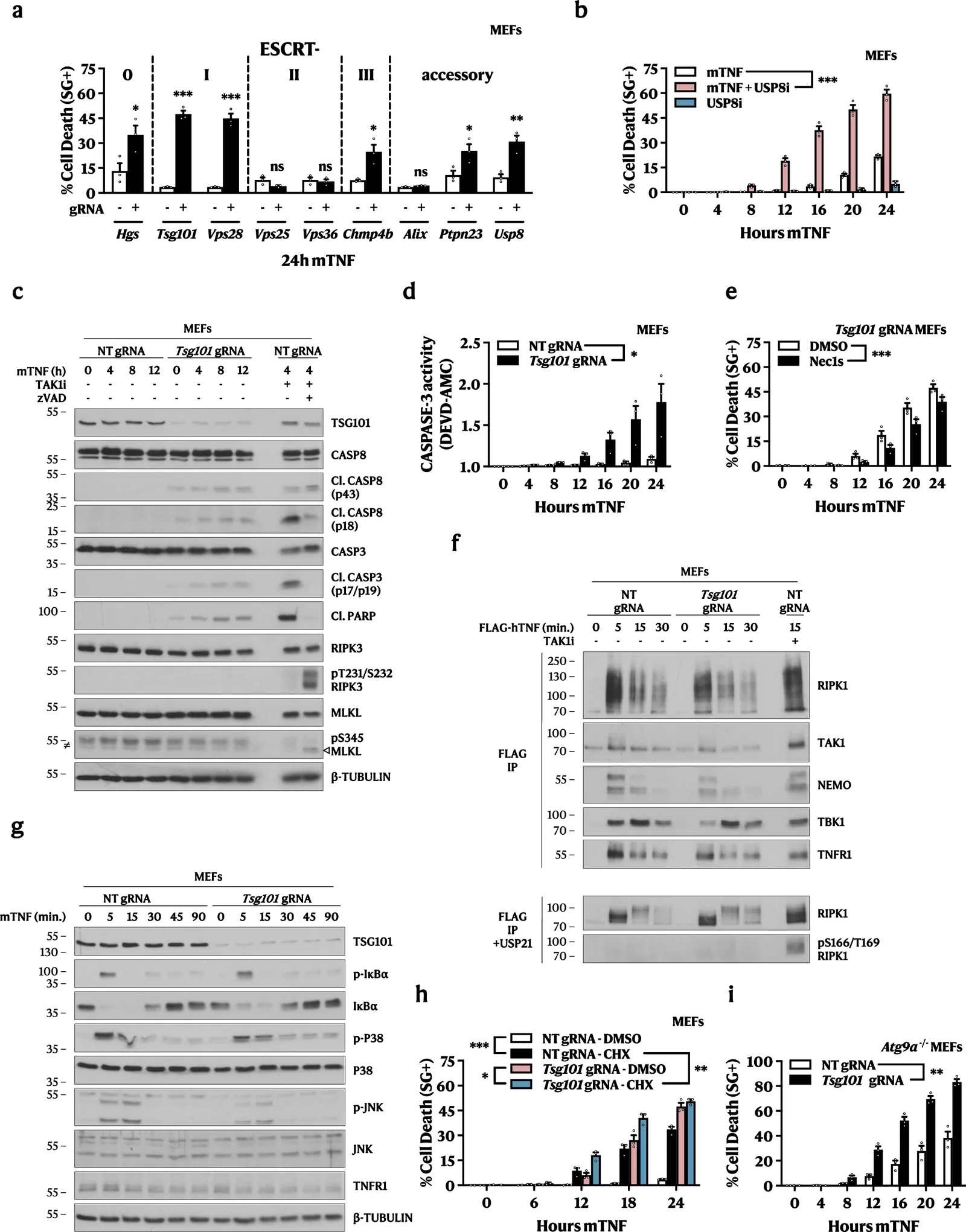
The ESCRT machinery prevents TNF-induced RIPK1 kinase-independent apoptosis independently of the known cell death checkpoints. **a** MEFs transduced with LentiCRISPRv2 constructs or electroporated with Cas9-RNPs targeting the indicated genes or a non-targeting sequence (NT) were stimulated with 20 ng/mL mTNF, and treatment-induced cell death was measured by SytoxGreen (SG) positivity. **b** MEFs were pre-treated with USP8i as indicated prior to stimulation with 20 ng/mL mTNF. Treatment-induced cell death was measured by SG positivity. **c**–**h** MEFs inducibly expressing Cas9 were transduced with inducible expression vectors encoding sgRNAs targeting *Tsg101* or NT, treated with 1 μg/mL doxycycline for 48 h and subsequently expanded for 72 h. **c** Cells were then pre-treated with TAK1 kinase inhibitor (TAK1i) and/or pan-caspase inhibitor (zVAD) as indicated and stimulated with 20 ng/mL mTNF. Protein levels were determined by immunoblot (≠ denote aspecific bands; ◁ indicates the pMLKL band). **d** Cells were stimulated with 20 ng/mL mTNF, and extracellular caspase activity was quantified using the fluorescent substrate DEVD-AMC. **e** Cells were pre-treated with Nec1s as indicated prior to stimulation with 20 ng/mL mTNF. Treatment-induced cell death was measured by SG positivity. **f** Cells were pre-treated with TAK1i as indicated prior to stimulation with 1 μg/mL FLAG-hTNF. TNFR1 Complex I was isolated by FLAG immunoprecipitation, followed by USP21 treatment as indicated. Protein levels were determined by immunoblot. **g** Cells were stimulated with 20 ng/mL mTNF, and protein levels were determined by immunoblot. **h** Cells were pre-treated with CHX as indicated prior to stimulation with 20 ng/mL mTNF. Treatment-induced cell death was measured by SG positivity. **i** *Atg9a* ^−/−^ MEFs obtained by clonal isolation following Cas9-RNP electroporation were additionally electroporated with *Tsg101*-specific or NT Cas9-RNPs. Cells were stimulated with 0.1 ng/mL mTNF, and treatment-induced cell death was measured by SG positivity. Immunoblots are representative of two independent experiments. Cell death and CASPASE-3 activity experiments are presented as mean ± SEM of three independent experiments, and statistical significance was determined using either multiple unpaired two-sided t-tests or two-way ANOVA. Significance between samples is indicated in the figure as follows: *p < 0.05; **p < 0.01; and ***p < 0.001; ns nonsignificant.

To investigate the potential protective role of eMI during TNF signaling, we first evaluated how targeting the ESCRT machinery, which is essential for the generation of ILVs during eMI, affected the TNF response. To do so, we used the CRISPR-Cas9 technology to generate mouse embryonic fibroblasts (MEFs) deficient for multiple components of the ESCRT machinery, and then monitored the induction of death following stimulation of these cells with TNF (Fig. 1a and Supplementary Fig. 1a). Interestingly, we found that deficiency in the ESCRT-0 component HGS, the ESCRT-I components TSG101 and VPS28 and the ESCRT-III component CHMP4B switched the TNF response to death (Fig. 1a and Supplementary Fig. 1a). Moreover, inhibition of the ESCRT accessory deubiquitinase USP8, obtained either genetically or pharmacologically by the USP8 inhibitor DC-U4106 (USP8i), also sensitized MEFs and human HT1080 cells to TNF cytotoxicity (Fig. 1a, b, and Supplementary Fig. 1a, g). Surprisingly, deficiency in the ESCRT-II components VPS25 and VPS36 or in the accessory protein ALIX did not have the same effect (Fig. 1a and Supplementary Fig. 1a). Moreover, this lack of phenotype could not be attributed to redundancy between ALIX and ESCRT-II, as simultaneous deletion of ALIX and the ESCRT-II component VPS36 likewise failed to shift the TNF response to death (Supplementary Fig. 1b, c). In line with a recent report^28^, we did, however, identify a cytoprotective function for the ALIX paralogue PTPN23 (Fig. 1a), which was recently shown to specifically contribute to eMI^29^. PTPN23 functionally cooperates with ESCRT-0, ESCRT-I, and ESCRT-III and is required to facilitate endosomal cargo sorting and MVB morphogenesis^29–31^. To better define the protective function of these ESCRT components, we set out to characterize the cell death modality induced by TNF in ESCRT-deficient cells. TNF has the capacity to kill cells by RIPK1 kinase-independent apoptosis, RIPK1 kinase-dependent apoptosis or RIPK1 kinase-dependent necroptosis. Cell death observed in TSG101-, VPS28- and USP8-deficient MEFs and USP8i-treated MEFs and HT1080 cells displayed typical features of extrinsic apoptosis, including cleavage of CASP8, CASP3, and PARP (Fig. 1c) and increased CASP3 activity (Fig. 1d and Supplementary Fig. 1d–f, h). The cell death was found not to rely on RIPK1 kinase activity, since it was not prevented by the RIPK1 kinase inhibitor Nec1s (Fig. 1e and Supplementary Fig. 1i–k). Accordingly, depleting TSG101 did not induce RIPK1 activation in TNFR1 Complex I, as monitored by autophosphorylation on S166/T169 (Fig. 1f and Supplementary Fig. 1l). Furthermore, we did not detect RIPK3 and MLKL phosphorylation (markers for necroptosis induction) in TSG101-deficient cells at the specified time points (Fig. 1c), confirming that these cells do not die by necroptosis. The identified protective role of the ESCRT components is consequently also independent from its previously reported function in plasma membrane repair during TNF-induced necroptosis^32^. We further excluded a contribution of ESCRT-mediated plasma membrane repair by interfering with extracellular calcium influx, which is required to trigger ESCRT recruitment to sites of membrane damage. The entry of extracellular Ca^2+^ through membrane pores was shown to activate the ESCRT-dependent outward budding and shedding of damaged membrane fragments, thereby restoring plasma membrane integrity^32–35^. In line with previous literature^32^, chelation of Ca^2+^ with BAPTA-AM, preventing ESCRT recruitment and activation at the plasma membrane, markedly sensitized cells to TNF-induced necroptosis obtained by pre-treatment with the pan-caspase inhibitor zVAD-fmk (zVAD) (Supplementary Fig. 1m, n). However, BAPTA-AM did not mimic ESCRT deficiency in the absence of zVAD, thereby excluding plasma membrane repair as the primary cytoprotective function of the ESCRT machinery downstream of TNF sensing.

TNF-mediated RIPK1 kinase-independent apoptosis is classically observed in conditions affecting the NF-κB checkpoint (consisting in the transcriptional upregulation of pro-survival factors, such as cFLIP, which counteract lethal CASP8 activation in TNFR1 Complex II) or the LC3-independent macro-autophagy checkpoint (consisting in the lysosomal detoxification of TNFR1 Complex II)^12,36,37^. The induction of TNF cytotoxicity through inactivation of the NF-κB checkpoint may be supported by a recent study attributing a role for the ESCRT machinery in the regulation of the endolysosomal trafficking of TNFR1 and, consequently, of TNF-mediated signaling to NF-κB^28^. In contrast to this report, we did not observe significant defects in the formation and disassembly of TNFR1 Complex I in MEFs deficient for the ESCRT component TSG101 (Fig. 1f and Supplementary Fig. 1l). Accordingly, we also did not observe significant differences in TNF-mediated NF-κB and MAPK signaling in these cells (Fig. 1g). Furthermore, we found that deficiency in TSG101, VPS28 and USP8 resulted in a significant increase in TNF-induced death and CASPASE activation in the presence of the translation inhibitor cycloheximide (CHX) (Fig. 1h and Supplementary Fig. 1o–q, y), thereby demonstrating that the pro-survival functions of these proteins is independent of gene activation and, consequently, of the NF-κB checkpoint. A cytoprotective role of USP8 downstream of TNF sensing has previously been attributed to its ability to deubiquitinate cFLIP, thereby preventing its proteasomal degradation^38^. Our data now strongly support an additional function for USP8 in counteracting TNF cytotoxicity that operates independently of NF-κB-mediated cFLIP upregulation. Of note, sensitization to TNF cytotoxicity in the presence of CHX was not observed in MEFs deficient in the ESCRT-II components VPS25 and VPS36 or in the ESCRT accessory protein ALIX (Supplementary Fig. 1r–t), which is consistent with our earlier findings (Fig. 1a). As the ESCRT machinery is additionally reported to be involved in the closure of phagophores during macro-autophagy^39–41^, the induced TNF cytotoxicity could alternatively originate from inactivation of the LC3-independent macro-autophagy checkpoint. To distinguish the function of the ESCRT machinery from its role during macro-autophagy, we investigated the impact of ESCRT inhibition in macro-autophagy-deficient conditions. We found that ESCRT deficiency increased the amount of cell death and CASPASE activation in ATG9A-deficient cells or cells pre-treated with the VPS34 inhibitor SAR405 (VPS34i) (Fig. 1i and Supplementary Fig. 1u–x, z), demonstrating a cytoprotective function that is independent of the macro-autophagy checkpoint. Notably, these findings also exclude the possibility that the sensitivity to TNF cytotoxicity observed in ESCRT-deficient cells exclusively originates from the previously reported non-canonical, lethal activation of CASP8 on unsealed phagophores^42^. Taken together, these data establish a previously unidentified role for the ESCRT machinery in counteracting TNF cytotoxicity.

### Endosomal ILV formation prevents TNF-induced RIPK1 kinase-independent apoptosis

**Fig. 2:**
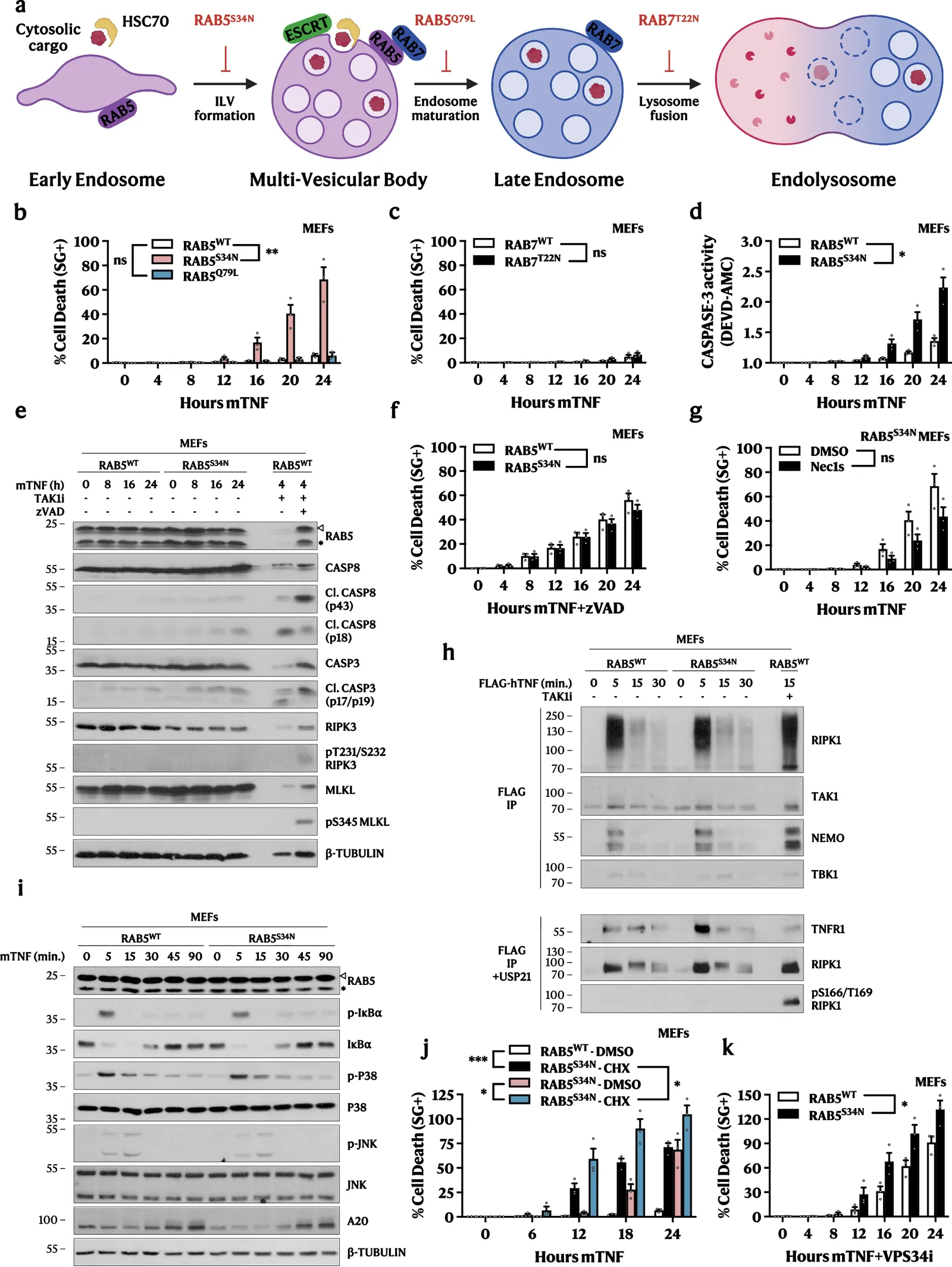
Endosomal ILV formation prevents TNF-induced RIPK1 kinase-independent apoptosis. **a** Graphical summary of the endolysosomal pathway and how to block the pathway using dominant-negative RAB mutants. **b** MEFs inducibly expressing 3xHA-tagged wild-type RAB5 (RAB5^WT^), or the dominant-negative RAB5 Q79L or S34N (RAB5^Q79L^ and RAB5^S34N^) were treated with 2 μg/mL doxycycline for 48 h before stimulation with 20 ng/mL mTNF. Treatment-induced cell death was measured by SytoxGreen (SG) positivity. **c** MEFs inducibly expressing 3xHA-tagged wild-type RAB7 (RAB7^WT^) or the dominant-negative RAB7 T22N (RAB7^T22N^) were treated with 2 μg/mL doxycycline for 48 h before stimulation with 20 ng/mL mTNF. Treatment-induced cell death was measured by SG positivity. **d**–**k** MEFs inducibly expressing RAB5^WT^ or RAB5^S34N^ were treated with 2 μg/mL doxycycline for 48 h. **d** Cells were then stimulated with 20 ng/mL mTNF, and extracellular caspase activity was quantified using the fluorescent substrate DEVD-AMC. **e** Cells were then pre-treated with TAK1i and/or zVAD as indicated and stimulated with 20 ng/mL mTNF. Protein levels were determined by immunoblot (* denotes the endogenously expressed RAB5; ◁ indicates the ectopically expressed 3xHA-tagged RAB5). **f** Cells were then pre-treated with zVAD and stimulated with 20 ng/mL mTNF. Treatment-induced cell death was measured by SG positivity. **g** MEFs expressing RAB5^S34N^ were pre-treated with Nec1s as indicated before stimulation with 20 ng/mL mTNF. Treatment-induced cell death was measured by SG positivity. **h** Cells were pre-treated with TAK1i as indicated before stimulation with 1 μg/mL FLAG-hTNF. TNFR1 Complex I was isolated by FLAG immunoprecipitation, followed by USP21 treatment as indicated. Protein levels were determined by immunoblot. **i** Cells were stimulated with 20 ng/mL mTNF and protein levels were determined by immunoblot. (* denotes the endogenously expressed RAB5; ◁ indicates the ectopically expressed 3×HA-tagged RAB5). **j**, **k** Cells were pre-treated with CHX or VPS34i as indicated prior to stimulation with 20 ng/mL mTNF. Treatment-induced cell death was measured by SG positivity. Immunoblots are representative of two independent experiments. Cell death and CASPASE-3 activity experiments are presented as mean ± SEM of three independent experiments, and statistical significance was determined using two-way ANOVA. Significance between samples is indicated in the figure as follows: *p < 0.05; **p < 0.01; and ***p < 0.001; ns nonsignificant. **a** Image created in BioRender. Delanghe, T. (2026) https://BioRender.com/gzutm2k.

To determine if the protective role of the ESCRT machinery is instead linked to ILV formation at the endosomal membrane, we next evaluated the impact of dominant-negative RAB5 and RAB7 mutants on TNF cytotoxicity (Fig. 2a). While both the Q79L and S34N mutants of RAB5 (RAB5^Q79L^ and RAB5^S34N^) perturb the endolysosomal network, they have opposite effects on the morphological and functional properties of the early endosome (EE)^43^. Expression of RAB5^S34N^ has a negative effect on the fusogenic properties of early endocytic vesicles and results in an early endocytic profile with very small vesicles that fail to internalize cargo by ILV formation^43^. In contrast, expression of RAB5^Q79L^ massively increases the fusion of early endocytic vesicles, leading to an enlarged early endocytic profile that boosts the internalization of cargo via ILV formation, but fails to mature to late endosomes (LEs)^43^. Finally, the T22N mutant of RAB7 (RAB7^T22N^) has a dominant-negative effect on the fusion of LEs with the lysosomes^44^. Thus, if the ESCRT-dependent formation of ILVs at the endosomal membrane is required to prevent TNF cytotoxicity, only RAB5^S34N^ is expected to switch the TNF response to death, as the formation of ILVs is unaffected in the other RAB mutants (Fig. 2a). Accordingly, we found that while ectopic expression of RAB5^Q79L^ and RAB7^T22N^ had no impact on TNF cytotoxicity, cells expressing RAB5^S34N^ died in response to TNF sensing (Fig. 2b, c, and Supplementary Fig. 2a). Like ESCRT inhibition, expression of RAB5^S34N^ triggered TNF-mediated RIPK1 kinase-independent apoptosis independently of the NF-κB and macro-autophagy checkpoints. Indeed, the RAB5^S34N^ mutant promoted the cleavage of CASP8 and CASP3, resulting in enhanced CASP3 activity, without any sign of RIPK3 or MLKL activation (Fig. 2d, e). Accordingly, expression of RAB5^S34N^ did not increase the extent of necroptosis induction downstream of TNF sensing when cells were pre-treated with the pan-caspase inhibitor zVAD-fmk (zVAD) (Fig. 2f). Moreover, TNF cytotoxicity in RAB5^S34N^-expressing MEFs was not prevented by the RIPK1 inhibitor Nec1s (Fig. 2g), which is in line with the absence of RIPK1 activation in TNFR1 Complex I (Fig. 2h and Supplementary Fig. 2b).

In line with a dominant-negative effect that did not affect the NF-κB checkpoint, no significant changes were observed in the formation of TNFR1 Complex I and subsequent NF-κB and MAPK signaling in MEFs expressing RAB5^S34N^ (Fig. 2h, i, and Supplementary Fig. 2b). Moreover, expression of RAB5^S34N^ increased TNF cytotoxicity when cells were pre-treated with CHX (Fig. 2j). Finally, cells expressing the dominant-negative RAB mutants showed no impairment in basal or starvation-induced LC3 lipidation, an established readout of macro-autophagy flux (Supplementary Fig. 2c–e). Accordingly, cells expressing RAB5^S34N^ were still sensitized when pre-treated with VPS34i, confirming that the dominant-negative effect of the mutant was independent of a defect in the macro-autophagy checkpoint (Fig. 1k). The striking similarities in the TNF response observed in ESCRT-deficient cells and cells expressing the RAB5^S34N^ mutant suggest that ILV formation at the endosomal membrane is part of a cell death checkpoint in the TNF pathway.

### ILV formation protects against TNF cytotoxicity independently of TNFR1 trafficking

**Fig. 3:**
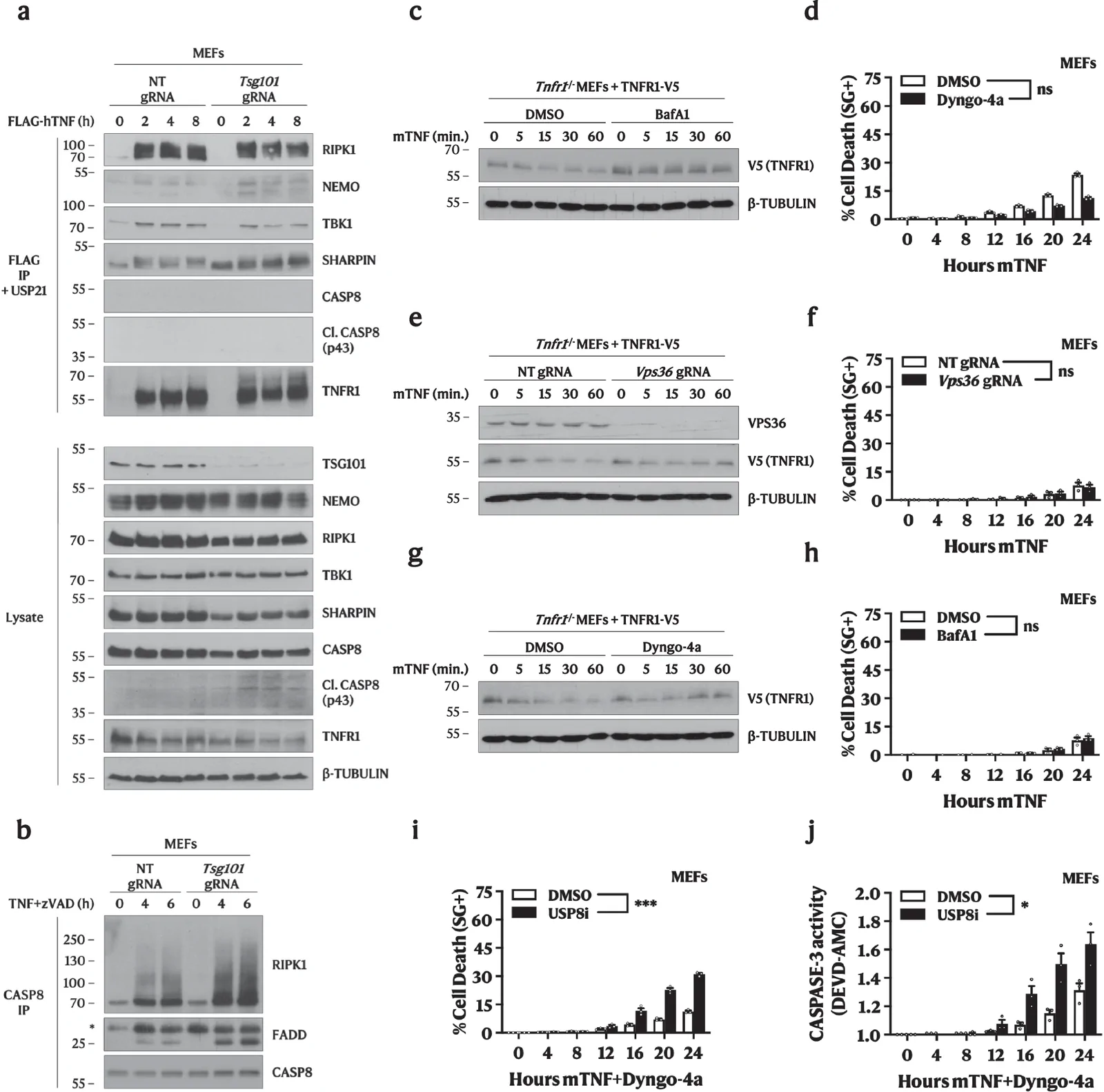
ILV formation protects against TNF cytotoxicity independently of TNFR1 trafficking. **a**, **b** MEFs inducibly expressing Cas9 were transduced with inducible expression vectors encoding sgRNAs targeting *Tsg101* or a non-targeting sequence (NT). Cells were treated with 1 μg/mL doxycycline for 48 h and subsequently expanded for 72 h. **a** Cells were stimulated with 1 μg/mL FLAG-hTNF. TNFR1 Complex I was isolated by FLAG immunoprecipitation, followed by USP21 treatment. Protein levels were determined by immunoblot. **b** Subsequently, cells were pre-treated with zVAD prior to stimulation with 20 ng/mL mTNF, and TNFR1 Complex II was isolated by CASP8 immunoprecipitation. Protein levels were determined by immunoblot (* denotes an IgG band). **c**, **e**, **g** *Tnfr1* ^−/−^ MEFs were transduced with an inducible expression vector encoding V5-tagged TNFR1. **e** Cells were electroporated with Cas9-RNPs targeting *Vps36* or NT. **f** MEFs were electroporated with Cas9-RNPs targeting *Vps36* or NT. **c**, **e**, **g** Cells were pre-treated with Dyngo-4a and bafilomycin A1 (BafA1) as indicated prior to stimulation with 20 ng/mL mTNF. Protein levels were determined by immunoblot. **h**–**j** MEFs were pre-treated with Dyngo-4a and USP8i as indicated prior to stimulation with 20 ng/mL mTNF. **d**, **f**, **h**, **i** Treatment-induced cell death was measured by SytoxGreen (SG) positivity. **j** Extracellular caspase activity was quantified over time using the fluorescent substrate DEVD-AMC. Cell death and CASPASE-3 activity experiments are presented as mean ± SEM of three independent experiments, and statistical significance was determined using a two-way ANOVA. Immunoblots are representative of two independent experiments.

The prototypical function of ESCRT-dependent ILV formation is to mediate the endolysosomal trafficking of plasma membrane receptors. TNFR1 is a well-known substrate of the endolysosomal pathway. Upon TNF sensing, TNFR1 is rapidly internalized via clathrin-mediated endocytosis (CME), and unlike many other receptors, TNFR1 is not recycled back to the plasma membrane, but rather targeted for lysosomal degradation^45^. In line with our data, a recent study demonstrated that ESCRT deficiency sensitizes cells to TNF cytotoxicity, and the authors attributed this effect to defective endolysosomal trafficking of TNFR1^28^. However, it is unclear how retention of TNFR1 at the endosomal membrane, due to impaired ILV formation, would switch the TNF response from life to death. Indeed, unlike other death receptor ligands, such as FasL and TRAIL, TNF does not promote the formation of a receptor-associated death-inducing signaling complex (DISC)^10^. Instead, the cytotoxic TNFR1 Complex II assembles as a cytosolic, receptor-dissociated complex. To determine whether this remains true when TNFR1 fails to enter ILVs and is retained on the endosomal membrane, we immunoprecipitated TNFR1 in TSG101-deficient MEFs at time points when CASP8 interacts with FADD and RIPK1. Our results show that CASP8 is not recruited to TNFR1 in either wild-type or TSG101-deficient cells (Fig. 3a, b, and Supplementary Fig. 3a), thereby excluding the formation of an endosomal DISC in ESCRT-deficient conditions. In line with the heightened sensitivity of ESCRT-deficient cells, we instead observed increased amounts of TNFR1 Complex II, as reflected by enhanced co-immunoprecipitation of FADD and ubiquitinated RIPK1 with CASP8 (Fig. 3b and Supplementary Fig. 3a). Immunoprecipitation of TNFR1 Complex II was performed in the presence of zVAD to prevent cleavage of RIPK1 by CASP8, as previously reported^11^.

To directly test whether altered trafficking of TNFR1 could trigger cell death, we systematically obstructed TNFR1 at distinct stages of the endolysosomal pathway: LEs, the endosomal membrane, and finally the plasma membrane. Treatment with the lysosomal fusion inhibitor Bafilomycin A1 (BafA1) confirmed that TNFR1 is targeted for lysosomal degradation following its engagement by TNF (Fig. 3c). However, preventing lysosomal delivery of TNFR1 with BafA1 did not affect cell viability (Fig. 3d)^12^. Consistently, blocking endolysosomal fusion through expression of RAB7^T22N^ also failed to sensitize MEFs to TNF cytotoxicity (Fig. 2c). Together, these results indicate that stabilizing TNFR1 within ILVs is not toxic to the cells. Next, to stall TNFR1 at the endosomal membrane, we interfered with its sequestration into ILVs by depleting the ESCRT-II component VPS36. Although VPS36-deficient MEFs exhibited impaired TNFR1 turnover (Fig. 3e), they did not succumb to TNF stimulation (Fig. 3f), which is in stark contrast to the loss of other ESCRT components (Fig. 1a). Finally, we blocked CME-dependent TNFR1 internalization using the DYNAMIN-1 inhibitor Dyngo-4a. Treatment with Dyngo-4a effectively inhibited TNFR1 turnover (Fig. 3g), but did not alter TNFR1 Complex I dynamics nor sensitize MEFs to TNF cytotoxicity (Fig. 3h and Supplementary Fig. 3b). These findings were confirmed using a dominant-negative DYNAMIN-1 mutant (DYN1^S45N^) and a dominant-negative AP180 fragment (AP180^C-term^) (Supplementary Fig. 3c, d), both described to effectively block CME^46,47^. Importantly, TSG101 deficiency, expression of RAB5^S34N^, or pre-treatment with Dyngo-4a did not significantly affect surface-accessible TNFR1 under steady-state conditions (Supplementary Fig. 3e–g).To further demonstrate that the cytoprotective effect of the ESCRTs is independent from the ESCRT-dependent sorting of TNFR1 into ILVs, we tested the effect of ESCRT inhibition by USP8i in cells in which TNFR1 internalization was blocked by Dyngo-4a. Remarkably, USP8i treatment still sensitized to TNF-mediated apoptosis in these conditions (Fig. 3i, j).

Taken together, these results demonstrate that neither the formation of TNFR1 Complex II nor its capacity to induce cell death depends on the endolysosomal trafficking of activated TNFR1. Accordingly, this demonstrates that ESCRT-dependent ILV formation prevents TNF-induced cell death through a mechanism that is independent of TNFR1 trafficking.

### Targeting of activated CASP8 into ILVs contributes to the prevention of TNF-induced cytotoxicity

**Fig. 4:**
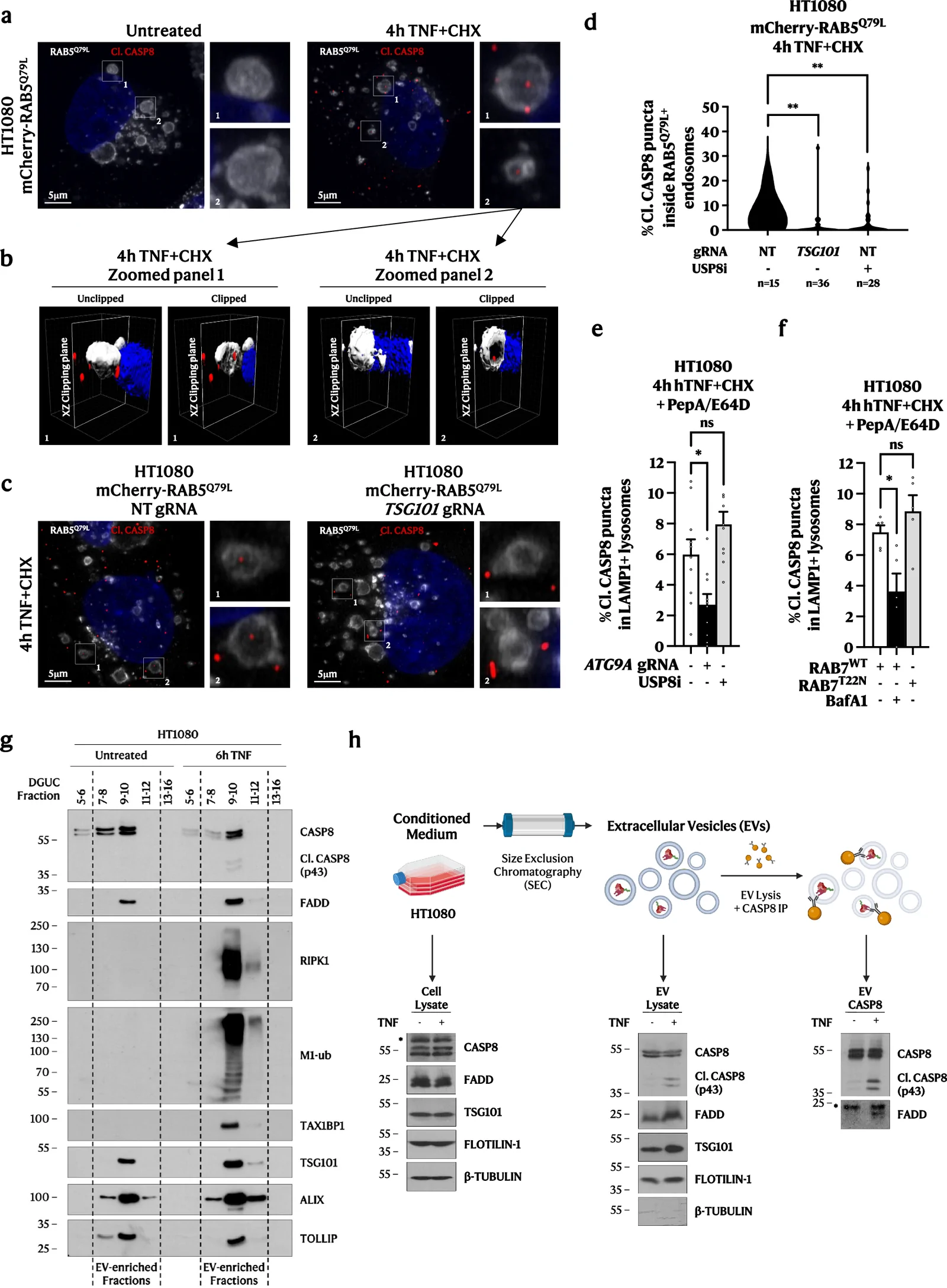
Targeting of activated CASP8 into ILVs contributes to the prevention of TNF-induced cytotoxicity. **a**, **b** HT1080 cells inducibly expressing mCherry-tagged RAB5 Q79L (mCherry-RAB5^Q79L^) were treated with 2 μg/mL doxycycline for 48 h prior to pre-treatment with CHX and stimulation with 1 µg/mL hTNF as indicated. **a** Representative maximum intensity projections of Cl. CASP8 immunostainings are shown. **b** 3D reconstruction of subpanel images are shown and clipped along an XZ-plane to allow visualization of the contents of mCherry-RAB5^Q79L+^ endosomes. **c**, **d** HT1080 cells inducibly expressing mCherry-RAB5^Q79L^ electroporated with Cas9-RNPs targeting *TSG101* or a non-targeting sequence (NT) were treated with 2 µg/mL doxycycline for 48 h before pre-treatment with CHX and USP8i as indicated and stimulation with 1 µg/mL hTNF. **c** Representative images of Cl. CASP8 immunostainings are shown for each genotype. **d** The percentage of Cl. CASP8 inside mCherry-RAB5^Q79L+^ endosomes was quantified. Violin plots represent the distribution over individual imaged cells. **e** HT1080 cells were electroporated with Cas9-RNPs targeting *ATG9A* or NT. **f** HT1080 cells inducibly expressing 3×HA-tagged wild-type RAB7 (RAB7^WT^) or the dominant-negative RAB7 T22N mutant (RAB7^T22N^) were treated with 2 µg/mL doxycycline for 48 h. **e**, **f** Cells were pre-treated with CHX, USP8i, bafilomycin A1 (BafA1) and pepstatin A (PepA)/E64D as indicated before stimulation with 2 μg/mL hTNF. Cells were stained for Cl. CASP8 and the lysosome marker LAMP1. The percentage of Cl. CASP8 puncta that colocalized with LAMP1^+^ lysosomes was quantified and presented as mean ± SEM across images. **g**, **h** HT1080 cells were treated with 2 µg/mL hTNF as indicated. **g** Conditioned medium was separated by density gradient ultracentrifugation (DGUC). Protein levels were determined by immunoblot. **h** Cells were lysed, and extracellular vesicles (EVs) were isolated using size exclusion chromatography (SEC) (fractions 4–6). A portion was used to isolate TNFR1 Complex II via CASP8 immunoprecipitation; the other portion was lysed. Protein levels were determined by immunoblot (* denotes an IgG band or an aspecific band). Immunoblots are representative of two independent experiments. Statistical significance was determined using a Kruskal–Wallis test or one-way ANOVA followed by a Dunn’s multiple comparisons test. Significance between samples is indicated in the figure as follows: *p < 0.05; **p < 0.01; and ***p < 0.001; ns non-significant. **h** Image created in BioRender. Delanghe, T. (2026) https://BioRender.com/gcxauyh.

Besides facilitating the removal and recycling of membrane receptors, the formation of endosomal ILVs also allows cytosolic material to be packaged and sent to lysosomes for degradation during eMI (Fig. 2a). Our previous work demonstrated that the cytosolic TNFR1 Complex II is targeted for lysosomal detoxification by an unconventional selective macro-autophagy pathway, preventing TNF-induced RIPK1 kinase-independent apoptosis^12^. Interestingly, interfering with ILV formation at the endosomal membrane, either by inhibition of the ESCRT machinery (Fig. 1) or the expression of the dominant-negative RAB5^S34N^ mutant (Fig. 2), switches the TNF response to the same cell death modality. This suggests that the sequestration of TNFR1 Complex II into ILVs would act as a parallel and non-redundant detoxification process that removes the complex from the cytosol. Consistently, we showed earlier that TSG101 deficiency in MEFs leads to the accumulation of TNFR1 Complex II (Fig. 3a and Supplementary Fig. 3a). To formally demonstrate this hypothesis, we monitored the presence of activated (cleaved (Cl.)) CASP8 (the catalytic subunit of TNFR1 Complex II) within ILVs of MVBs by fluorescence microscopy. Immunostainings were performed in MEFs and HT1080 cells ectopically expressing a fluorescently (mCherry) tagged version of the dominant-negative RAB5^Q79L^ mutant (mCherry-RAB5^Q79L^) (Supplementary Fig. 4a, d). Expression of mCherry-RAB5^Q79L^ drastically increases the size of MVBs, allowing the easy detection of cargo on the lumenal side of an MVB (Supplementary Fig. 4b, c, e)^43^. We performed these experiments in the presence of CHX or of the IKKα/β inhibitor TPCA-1 (IKKi) to boost TNFR1 Complex II formation and facilitate detection of activated CASP8. In line with our hypothesis, we were able to detect the TNF-dependent presence of activated CASP8 in the lumen of mCherry-RAB5^Q79L+^ endosomes (Fig. 4a and Supplementary Fig. 4f). 3D reconstruction images of the enlarged mCherry-RAB5^Q79L+^ endosomes confirmed that Cl. CASP8 puncta were present at the luminal side of these MVBs (Fig. 4b). In accordance with the endosomal targeting of Cl. CASP8, as part of the TNFR1 Complex II, additional immunostainings in FADD-deficient MEFs reconstituted with a GFP-tagged version of FADD revealed colocalization between TNF-induced FADD puncta and EEA1^+^ endosomes (Supplementary Fig. 4g, h). Moreover, consistent with the established role of the ESCRT machinery in transferring cytosolic material into ILVs, ESCRT inhibition, either genetically (TSG101 deletion) or pharmacologically (USP8i), significantly reduced the fraction of Cl. CASP8 puncta localized within mCherry-RAB5Q79L^+^ endosomes in HT1080 cells (Fig. 4c, d, and Supplementary Fig. 4i, j), without altering endosome number or size (Supplementary Fig. 4k). Next, to assess whether the ESCRT-dependent sequestration of TNFR1 Complex II into ILVs promotes its lysosomal turnover via eMI, we analyzed the effect of ESCRT inhibition on the co-localization of Cl. CASP8 puncta with the lysosomal marker LAMP1. These experiments were performed in the presence of the protease inhibitors Pepstatin A (PepA) and E64D to prevent lysosomal degradation and thereby allow accurate assessment of co-localization. Consistent with the targeting of TNFR1 Complex II by unconventional selective macro-autophagy^12^, ATG9A deficiency significantly reduced the fraction of LAMP1^+^ Cl. CASP8 puncta (Fig. 4e and Supplementary Fig. 4l). In contrast, ESCRT inhibition with USP8i did not alter the lysosomal localization of Cl. CASP8 (Fig. 4e), indicating that ESCRT-dependent ILV formation does not contribute to its targeting to lysosomes. Supporting these conclusions, we further observed that blocking all lysosomal fusion events with BafA1 markedly reduced the lysosomal localization of Cl. CASP8 puncta, whereas selective inhibition of Late Endosome-lysosome fusion through expression of RAB7^T22N^ had no such effect (Fig. 4f and Supplementary Fig. 4m). Together, these data indicate that the ESCRT-dependent targeting of activated CASP8 into ILVs does not lead to its lysosomal turnover.

Next to lysosomal delivery, extracellular release via exosomes is another possible outcome of the ESCRT-mediated targeting of activated CASP8 within ILVs. Consistently, using density gradient ultracentrifugation (DGUC) on the conditioned medium of TNF-treated HT1080 cells, we detected the presence of activated CASP8 inside EV-enriched density fractions that were positive for the exosome markers ALIX, TSG101, FLOTILIN-1, and TOLLIP (Fig. 4g and Supplementary Fig. 4n). Remarkably, FADD, ubiquitinated RIPK1, TAX1BP1, and M1-linked ubiquitin chains were also detected in these EV fractions, suggesting that activated CASP8 is targeted to exosomes as part of TNFR1 Complex II, potentially via a mechanism similar to the previously described LC3-independent macro-autophagy pathway^12^. These results were confirmed by isolating EVs using regular ultracentrifugation (Supplementary Fig. 4o) and size exclusion chromatography (SEC) (Supplementary Fig. 4p–r), an approach that allowed the further identification of TRADD and TRAF2, but not TRAF1, cIAP1 or cFLIP, as TNF-induced cargo of the isolated EVs (Supplementary Fig. 4r).

Importantly, the TNF concentration used in our experiments does not cause cytotoxicity in HT1080 cells (Supplementary Fig. 4s), making it unlikely that the detected TNFR1 Complex II components come from apoptotic bodies instead of exosomes. To rule this out, we generated apoptosis-resistant HT1080 cells lacking CASP3/7 (Supplementary Fig. 4u). Cl. CASP8 was still readily detected in EVs isolated from these cells (Supplementary Fig. 4v), confirming that the presence of TNFR1 Complex II components in EVs is not merely a by-product of apoptosis. Notably, because CHX increases the cellular levels of TNFR1 Complex II beyond the detection limit of our microscopy assays, CHX pre-treatment also increased the amount of Cl. CASP8 in EVs isolated from both wild-type and CASP3/7-deficient HT1080 cells (Supplementary Fig. 4t, v). Importantly, we were able to show that the TNFR1 Complex II components identified in EVs are most likely part of an active and intact complex, as FADD specifically co-immunoprecipitated with CASP8 when isolated from EVs obtained from TNF-treated cells (Fig. 4h). Of note, TNFR1 was not detected in EVs (Supplementary Fig. 4q), which is in accordance with the fact that TNFR1 Complex II assembles as a cytosolic complex and not as a DISC (Fig. 3a, b)^12^.

Together, these data demonstrate that sequestration of activated CASP8 into ILVs constitutes a parallel, non-redundant detoxification pathway that limits TNF-induced RIPK1 kinase-independent apoptosis and may facilitate its extracellular release as part of TNFR1 Complex II via exosomes. Interestingly, neither the quantity nor the biochemical and morphological properties of the released EVs changed significantly after TNF stimulation (Fig. 4g, h, and Supplementary Fig. 4o, q, r, t, v–y). This indicates that TNF sensing does not alter exosomes biogenesis itself, but instead promotes the targeting of TNFR1 Complex II for extracellular release.

### Linear ubiquitination of RIPK1 promotes the TAX1BP1- and HSC70-dependent targeting of activated CASP8 into ILVs

**Fig. 5:**
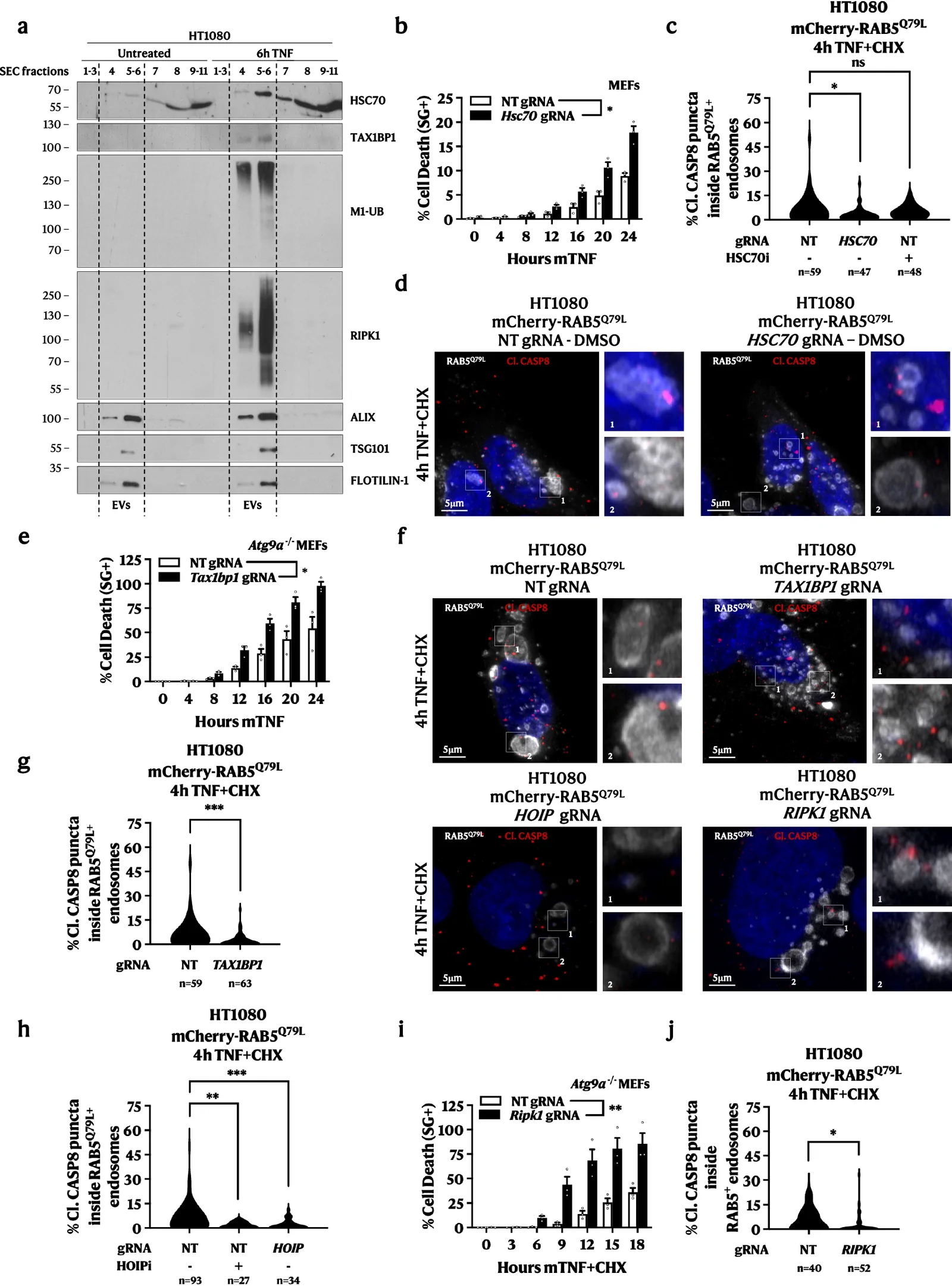
Linear ubiquitination of RIPK1 promotes the TAX1BP1- and HSC70-dependent targeting of activated CASP8 into ILVs. **a** HT1080 cells were stimulated with 2 µg/mL hTNF as indicated. Cells were lysed, and conditioned medium was harvested and separated into different fractions using size exclusion chromatography (SEC). Protein levels in each fraction were determined by immunoblot. **b** MEFs were electroporated with Cas9-RNPs targeting *Hsc70* or a non-targeting sequence (NT). Cells were then stimulated with 20 ng/mL mTNF, and treatment-induced cell death was measured by SytoxGreen (SG) positivity. **c**, **d**, **f**–**h**, **j** HT1080 cells inducibly expressing mCherry-tagged RAB5 Q79L mutant (mCherry-RAB5^Q79L^) were electroporated with Cas9-RNPs targeting the indicated genes or NT. Cells were treated with 2 µg/mL doxycycline (Dox) for 48 h. Subsequently, cells were pre-treated with CHX and 50 µM HSC70i or 30 µM of the HOIP inhibitor HOIPIN-8 (HOIPi) as indicated prior to stimulation with 1 µg/mL hTNF. Cells were then stained for Cl. CASP8. **c**, **g**, **h**, **j**, The percentage of Cl. CASP8 found inside mCherry-RAB5^Q79L+^ endosomes was quantified. Violin plots represent the distribution over individual imaged cells. **d**, **f** Representative maximum intensity projections of Cl. CASP8 immunostainings are shown. **e**, **i** *Atg9a* ^−/−^ MEFs obtained by clonal isolation of the following Cas9-RNP electroporation were additionally electroporated with the indicated genes or NT Cas9-RNPs. Cells were stimulated with 0.1 ng/mL mTNF. Treatment-induced cell death was measured by SG positivity. Immunoblots are representative of two independent experiments. Cell death experiments are presented as mean ± SEM of three independent experiments, and statistical significance was determined using either two-way ANOVA or multiple unpaired two-sided t-tests. Statistical significance of the microscopy data was determined using either a Kruskal–Wallis test followed by a Dunn’s multiple comparisons test or a two-sided Mann–Whitney test. Significance between samples is indicated in the figure as follows: *p < 0.05; **p < 0.01; and ***p < 0.001; ns nonsignificant.

HSC70 (encoded by the *Hspa8* gene) has been implicated in the selective loading of cytosolic cargo into ILVs^19,26^. Consistently, we detected HSC70 in EVs isolated from HT1080 cells, and its levels increased following TNF stimulation (Fig. 5a). Supporting a functional role for HSC70 in the ILV-mediated detoxification of activated CASP8, we found that HSC70 deficiency in MEFs led to a significant increase in TNF cytotoxicity (Fig. 5b). As previously reported, HSC70’s role in cargo selection does not depend on its ATPase activity^48^, and accordingly, pharmacological inhibition of its ATPase function with VER-155008 (HSC70i) did not shift the TNF response to death (Supplementary Fig. 5a). Of note, LAMP2 deficiency did not sensitize MEFs to TNF cytotoxicity (Supplementary Fig. 5c, d), indicating that HSC70’s cytoprotective effect is not due to its role in CMA^49^. Furthermore, in HT1080 cells, loss of HSC70 - but not its pharmacological inhibition - significantly reduced the amount of Cl. CASP8 puncta within mCherry-RAB5^Q79L+^ endosomes (Fig. 5c, d, and Supplementary Fig. 5b) without altering endosome number or size (Supplementary Fig. 5e), confirming that HSC70 supports the ILV-dependent detoxification of TNFR1 Complex II.

The targeting of TNFR1 Complex II via LC3-independent macro-autophagy was shown to rely on the autophagy receptor TAX1BP1, which associates with TNFR1 Complex II by binding to the M1-ubiquitin chains conjugated to RIPK1^12^. Interestingly, TAX1BP1 was recently shown to simultaneously target another cargo, ferritin, for lysosomal degradation via both LC3-independent macro-autophagy and its sequestration into ILVs during eMI^15,18^. Notably, we found that depleting TAX1BP1 further sensitized ATG9A-deficient MEF cells to TNF cytotoxicity (Fig. 5e and Supplementary Fig. 5f) and resulted in a significant increase in CASP3 activity (Supplementary Fig. 5g), suggesting an additional macro-autophagy-independent function of TAX1BP1 during TNF signaling. Interestingly, we also identified TAX1BP1 as a selective cargo of exosomes isolated from TNF-treated HT1080 cells (Figs. 4g, 5a, and Supplementary Fig. 4q). To directly determine whether TAX1BP1 contributes to the ILV-dependent detoxification of activated CASP8, we next evaluated its requirement to transfer Cl. CASP8 from the cytosol to the endosomal lumen. Remarkably, we found that depletion of TAX1BP1 in HT1080 cells resulted in a significant reduction of TNF-induced Cl. CASP8 puncta found in mCherry-RAB5^Q79L+^ endosomes (Fig. 5f, g, and Supplementary Fig. 5h), confirming that TAX1BP1 is indeed involved in the selective targeting of activated CASP8 into ILVs. We also identified M1-ubiquitin chains and ubiquitinated RIPK1 as selective cargoes of exosomes isolated from TNF-treated HT1080 cells (Figs. 4g, 5a, and Supplementary Fig. 4o, q, r). Interestingly, we found that M1-ubiquitination is similarly required for the targeting of activated CASP8 into ILVs. Indeed, HOIP inhibition, obtained either genetically or pharmacologically using the HOIP inhibitor HOIPIN-8 (HOIPi), significantly decreased the percentage of Cl. CASP8 puncta were found in mCherry-RAB5^Q79L+^ endosomes (Fig. 5f, h, and Supplementary Fig. 5j, k). RIPK1 is the primary M1-ubiquitinated substrate in both TNFR1 Complex I and Complex II, and is a critical regulator of the signaling outcomes downstream of TNF sensing. Intriguingly, we identified a cytoprotective function of RIPK1 downstream of TNFR1 activation that is independent of its essential scaffolding functions as M1-ubiquitinated substrate in the NF-κB and macro-autophagy checkpoint. Indeed, RIPK1 deficiency significantly increased cell death and CASP3 activation in ATG9A-deficient MEFs (inhibition of the macro-autophagy checkpoint) treated with TNF in combination with CHX (inhibition of the NF-κB checkpoint) (Fig. 5i and Supplementary Fig. 5m, n). In support of a model in which M1-ubiquitinated RIPK1 serves as a scaffold for the recognition of TNFR1 Complex II by TAX1BP1, we found that RIPK1 deletion in HT1080 cells prevented the transition of Cl. CASP8 from the cytosol to the inside of mCherry-RAB5^Q79L+^ endosomes (Fig. 5f, j, and Supplementary Fig. 5o). Notably, inhibition of TAX1BP1, HOIP, or RIPK1 did not negatively impact on endosome number or size (Supplementary Fig. 5i, l), arguing against a role for these factors in endosome biogenesis or stability and instead supporting their involvement in the targeting of activated CASP8. Interestingly, HSC70 was recently reported to directly interact with the RHIM domain of RIPK1, thereby limiting TNF-induced necroptosis by acting as an amyloidase in an ATPase-dependent manner^50^. To assess whether this interaction also contributes to the HSC70-dependent detoxification of activated CASP8, we reconstituted RIPK1-deficient MEFs with a RIPK1 mutant that fails to interact with HSC70 (RIPK1^M536G^)^50^. Cells reconstituted with RIPK1^M536G^ were not sensitized to TNF cytotoxicity compared to those reconstituted with wild-type RIPK1 (RIPK1^WT^) (Supplementary Fig. 5p, q), arguing against the involvement of the identified interaction in the selective targeting of activated CASP8 into ILVs. This observation also suggests that the primary function of HSC70 in counteracting TNF cytotoxicity is not related to its role as an ATPase-dependent amyloidase during necroptosis, but rather consists in its ATPase-independent role in targeting activated CASP8 into ILVs.

### Microbial effector proteins shift the TNF response from life to death by interfering with the ILV-dependent detoxification of activated CASP8

**Fig. 6:**
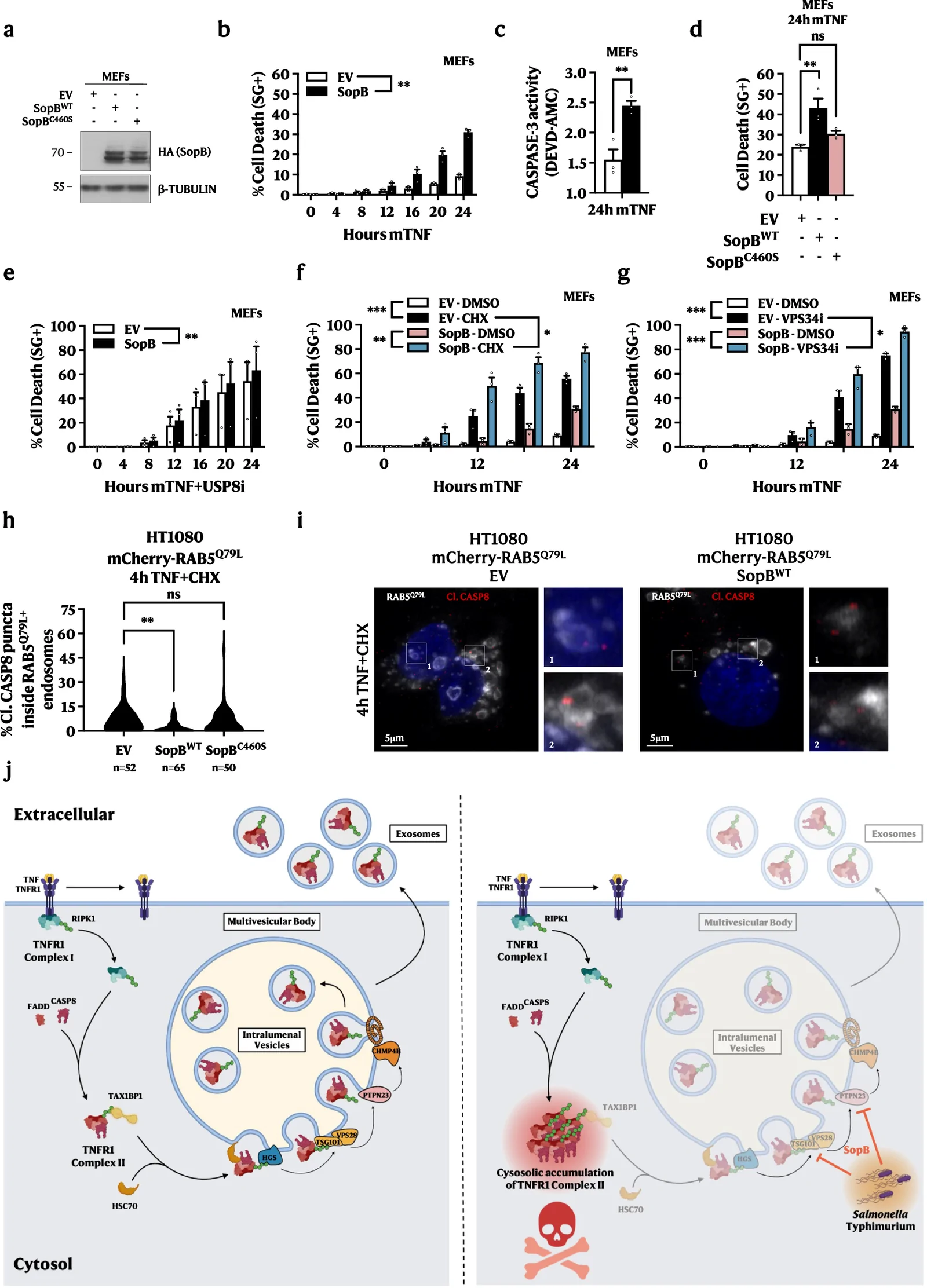
Microbial effector proteins shift the TNF response from life to death by interfering with the ILV-dependent detoxification of activated CASP8. **a**–**g** MEFs inducibly expressing an empty vector (EV) or the *Salmonella* Typhimurium effector protein SopB wild-type (SopB^WT^) or C460S (SopB^C460S^) were treated with 2 µg/mL doxycycline for 48 h prior to pre-treatment with CHX, VPS34i, USP8i as indicated and stimulation with 20 ng/mL mTNF. **b**, **e–g** Treatment-induced cell death was measured by SytoxGreen (SG) positivity. **c** Extracellular caspase activity was quantified using the fluorescent substrate DEVD-AMC. **d** Cell death was measured by SG positivity. **h**, **i** HT1080 cells inducibly expressing mCherry-tagged RAB5 Q79L (mCherry-RAB5^Q79L^) were additionally transduced with inducible expression vectors encoding SopB^WT^ or SopB^C460S^. Cells were treated with 2 µg/mL doxycycline for 48 h prior to pre-treatment with CHX and stimulation with 1 µg/mL hTNF. Cells were then stained for Cl. CASP8. **h** The percentage of Cl. CASP8 found inside mCherry-RAB5^Q79L+^ endosomes was quantified. Violin plots represent the distribution over individual imaged cells. **i** Representative maximum intensity projections of Cl. CASP8 immunostainings are shown. Cell death and CASPASE-3 activity experiments are presented as mean ± SEM of three independent experiments, and statistical significance was determined using either two-way ANOVA, unpaired two-sided t-test, or one-way ANOVA followed by a Dunnett’s multiple comparisons test. Statistical significance of the microscopy data was determined using a Kruskal-Wallis test followed by a Dunn’s multiple comparisons test. Significance between samples is indicated in the figure as follows: *p < 0.05; **p < 0.01; and ***p < 0.001; ns non-significant. **j** Graphical overview of the ESCRT-dependent detoxification of activated CASP8 by extracellular release via exosomes. LUBAC decorates RIPK1 with M1-linked ubiquitin chains, which are recognized by the autophagy receptor TAX1BP1. Together with HSC70, TAX1BP1 then promotes the recruitment of activated CASP8 to the limiting membrane of endosomes and its ESCRT-dependent incorporation into intralumenal vesicles (ILVs). The multivesicular body (MVB) then fuses with the plasma membrane to release activated CASP8 into the extracellular environment as part of exosomes. Disruption of this pathway - for example, due to the action of SopB - prevents detoxification, promoting the cytosolic accumulation of activated CASP8 and the induction of TNF-induced RIPK1 kinase-independent apoptosis. Image created in BioRender. Delanghe, T. (2026) https://BioRender.com/ahbo3r6.

TNF and programmed cell death (including apoptosis) play important roles in the control of infections caused by the intracellular pathogens *Salmonella* Typhimurium (*S*. Typhimurium) and *Mycobacterium tuberculosis* (*Mtb*)^51–55^. While it is known that infections by these pathogens trigger death of the host cell, the cause of death remains largely unknown. Interestingly, both pathogens produce microbial effector proteins that interfere with the host ESCRT machinery. Indeed, the *S*. Typhimurium effector protein SopB is a phosphoinositide phosphatase that interferes with a subset of ESCRT proteins interacting with these phosphorylated lipids^56^. The *Mtb* effector protein EsxH, in complex with EsxG, directly inhibits the ESCRT-0 component HGS to disrupt delivery of the bacterium to the lysosomes^57^. Given the importance of the ESCRT machinery for ILV formation, we hypothesized that these effector proteins inactivate the ILV-dependent detoxification of activated CASP8 and expose cells to TNF cytotoxicity. While expression of SopB in MEFs or EsxG-EsxH in HT1080 cells was not toxic in itself (Fig. 6a and Supplementary Fig. 6a, e, f), it affected cell viability and promoted CASP3 activity upon TNF stimulation (Fig. 6b, c, and Supplementary Fig. 6g). Of note, similar to RAB5^S34N^ (Fig. 2f), expression of SopB did not sensitize MEFs to TNF-induced necroptosis when pre-treated with zVAD (Supplementary Fig. 6b). Importantly, expression of a catalytically inactive mutant of SopB (SopB^C460S^)^56^ did not have any impact on TNF cytotoxicity (Fig. 6a, d). Similarly, mutating the HGS interaction site in EsxH (EsxH^H76A/E77A^)^57^ cancelled its cytotoxic potential (Supplementary Fig. 6e–g), confirming that the effect of these effector proteins is due to their ability to interfere with the ESCRT machinery. This notion was further supported by the fact that SopB and EsxG-H have no additional sensitizing effect when the ESCRT machinery is already inhibited by pre-treatment with USP8i (Fig. 6e and Supplementary Fig. 6h). Moreover, the sensitizing effect of SopB and EsxG-H was found to be independent of the NF-κB and macro-autophagy checkpoint. Indeed, expression of both effector proteins additionally sensitized cells when pre-treated with CHX or VPS34i (Fig. 6f, g, and Supplementary Fig. 6i). In line with the idea that SopB would inhibit ILV formation, we found that the expression of SopB, but not the catalytic inactive SopB^C460S^, significantly reduced the amount of Cl. CASP8 found in mCherry-RAB5^Q79L+^ endosomes (Fig. 6h, i, and Supplementary Fig. 6c, d). These results indicate that the phosphoinositide phosphatase activity of SopB inactivates the ILV-dependent detoxification of activated CASP8. Similar results were obtained for EsxG-H and the EsxG-H^H76A/E77A^ mutant (Supplementary Fig. 6j–l), confirming that by interfering with the ESCRT machinery, SopB and EsxG-H inhibit the ILV-dependent detoxification of activated CASP8 and thereby shift the TNF response to death.

## Discussion

TNF sensing by TNFR1 leads to the assembly of two successive protein complexes: TNFR1 Complex I forms at the plasma membrane and is responsible for signaling to the MAPK and NF-κB pathways. TNFR1 Complex II is instead cytosolic and requires the dissociation of TNFR1 Complex I components from the receptor and the subsequent recruitment of FADD and CASP8. Activation of CASP8 within TNFR1 Complex II can induce extrinsic apoptosis by processing the downstream executioner caspases CASP3/7. However, despite the assembly of the TNFR1 Complex II, apoptosis is not the standard response of most cells to TNF sensing. Indeed, cell death checkpoints within the pathway limit CASP8 activity to prevent cell death induction. The lysosomal degradation of TNFR1 Complex II by an unconventional form of selective macro-autophagy is one of these checkpoints^12^. Here, we provide data demonstrating that TNFR1 Complex II is additionally detoxified via sequestration into ILVs and subsequent extracellular release as part of exosomes. Indeed, we show that upon TNF sensing, TNFR1 Complex II is targeted to the endosomal membrane, where the ESCRT machinery packages the complex into ILVs in preparation for its extracellular disposal as part of exosomes. We show that genetic or pharmacological inhibition of the engulfment of TNFR1 Complex II into ILVs results in the cytosolic accumulation of the complex and subsequent induction of RIPK1 kinase-independent apoptosis. Intriguingly, while deficiency in the ESCRT-0, -I, and -III components shifted the TNF response from life to death, this was not the case upon inactivation of ESCRT-II components or the accessory protein ALIX. Moreover, we found that this difference was not attributable to potential redundancy between ESCRT-II and ALIX in bridging ESCRT-I and ESCRT-III^58^. Instead, this function appeared to be mediated by the ALIX paralogue PTPN23^30^, whose deletion also activates TNF cytotoxicity. Recent results from an unbiased, genome-wide CRISPR knockout screen support the idea that the sequestration of cytosolic cargo into ILVs specifically requires PTPN23 - rather than ESCRT-II or ALIX - to mediate the handoff of cargo from ESCRT-I to ESCRT-III. In this screen, loss of PTPN23 uniquely impaired ILV-dependent degradation of ubiquitinated Tau aggregates by eMI^29^. In agreement with our findings, repression of TNF cytotoxicity by PTPN23 was also recently reported in a human leukemia cell line^28^. In that study, the authors attribute the protective effect of PTPN23 to the ESCRT-dependent endolysosomal trafficking of TNFR1, which would terminate TNF-mediated NF-κB/MAPK signaling and, through an unknown mechanism, also prevent cell death induction^28^. In contrast to that report, we did not observe substantial alterations in the assembly or disassembly of TNFR1 Complex I, nor did we detect clear defects in TNF-induced NF-κB or MAPK signaling. The basis for this discrepancy remains unclear, but may reflect cell type-specific differences in the extent to which TNFR1 Complex I is routed to the endolysosomal system. Importantly, our data provide direct mechanistic evidence that TNF cytotoxicity resulting from impaired ILV formation cannot be attributed to altered endolysosomal TNFR1 trafficking. Instead, we formally demonstrate that it arises from defective sequestration of TNFR1 Complex II into ILVs. Unlike other death receptors, TNFR1 does not recruit FADD and CASP8 to form a membrane-bound DISC^10^. Consistently, we show that even when ILV formation is blocked through ESCRT inhibition, and TNFR1 remains on endosomal membranes, TNFR1 is still unable to recruit FADD and CASP8. Instead, TNFR1 Complex II continues to assemble exclusively in the cytosol rather than forming a receptor-associated DISC. Moreover, we found that perturbing TNFR1 trafficking at various points along the endolysosomal pathway does not induce TNF-mediated cell death, indicating that altered receptor trafficking cannot account for the phenotype of ESCRT-deficient cells. Instead, our findings show that once formed, the cytotoxic TNFR1-dissociated Complex II is selectively targeted into ILVs through an ESCRT-dependent mechanism that operates independently of TNFR1 trafficking. Blocking this ILV-mediated sequestration leads to the cytosolic accumulation of TNFR1 Complex II and the induction of TNF-driven apoptosis. These results now provide a direct and robust mechanistic explanation for why defects in ILV formation sensitize cells to TNF cytotoxicity.

The ESCRT machinery has also previously been shown to protect cells from TNF-induced necroptosis by facilitating the extracellular shedding of MLKL pores from the plasma membrane^32,59^. However, phosphorylation of RIPK3 and MLKL (markers of necroptosis induction) was not observed upon stimulation of either wild-type or ESCRT-deficient MEFs with TNF. ESCRT deficiency in these cells, rather, resulted in the typical features of extrinsic apoptosis, a cell death modality in which plasma membrane integrity is maintained. Accordingly, preventing the activation of ESCRT-dependent plasma membrane repair^32–35^, by blocking the influx of extracellular Ca^2+^, did not switch the TNF response to death. The ESCRT machinery has additionally been reported to be involved in the closure of autophagosomes during macro-autophagy^39–41^. Consequently, the protective function of the ESCRTs against TNF cytotoxicity could have originated from its contribution to the LC3-independent macro-autophagy checkpoint^12,39–41^. While this hypothesis may not be formally excluded, we demonstrated that ESCRT deficiency sensitizes ATG9A-deficient cells to TNF-induced apoptosis, thereby establishing an additional macro-autophagy-independent role of the ESCRT machinery in preventing TNF cytotoxicity. Of note, as the role of the ESCRTs in plasma membrane repair was shown to additionally rely on ATG9A^59^, these results also further strengthen the notion that the primary function of the ESCRT components in TNF signaling is independent of plasma membrane repair. Moreover, the observation that ESCRT deficiency sensitizes cells to TNF cytotoxicity even in the absence of macro-autophagy also distinguishes our findings from the previously reported non-canonical, lethal activation of CASP8 on unsealed phagophores^42^. This conclusion is reinforced by the fact that expression of RAB5^S34N^ sensitizes cells to TNF-induced death without exerting any measurable effect on the macro-autophagy pathway.

We found that inhibition of the endolysosomal pathway at the level of ILV formation displayed striking similarities with the effects observed in ESCRT-deficient cells, leading to the hypothesis that the sequestration of activated CASP8 into ILVs functions as a detoxification mechanism. Accordingly, we established the TNF-dependent targeting of activated CASP8 to MVBs. Intriguingly, we found that the sequestration of activated CASP8 into ILVs does not contribute to its lysosomal degradation, but appears to exclusively target it for extracellular release as part of exosomes. The identification of Cl. CASP8, along with other TNFR1 Complex II components (FADD, TRADD, TRAF2, ubiquitinated RIPK1, M1-ubiquitin chains, and TAX1BP1) in isolated exosomes, suggests the transfer of the whole active complex. Consistently, we detected TNF-induced FADD puncta in EEs and a TNF-dependent interaction between FADD and CASP8 in exosomes isolated from TNF-treated cells. While we did not detect post-translational modifications of TRADD previously associated with TNFR1 Complex II^10,60^, we believe that this could reflect the dynamic and context-dependent nature of these modifications, or be a consequence of limited antibody specificity. Indeed, these modifications were also not detected in a recent study utilizing the same TRADD antibody^61^. In contrast, the presence of higher-molecular-weight forms of TRAF2 in EVs is consistent with its reported TNF-induced post-translational modification^62,63^ and further supports the incorporation of assembled, signaling-competent complexes into EVs. Although further interaction mapping would be required, these findings collectively suggest that activated CASP8 is targeted to exosomes as part of TNFR1 Complex II.

Exosomes, and EVs in general, are known to promote cellular homeostasis by removing harmful material from the cells, justifying their name as “cellular garbage bags.”^64^ Consistent with this role, we found that blocking the targeting of activated CASP8 to ILVs leads to its accumulation in the cytosol and triggers apoptosis. This finding establishes two independent and non-redundant detoxification arms: a lysosomal branch that relies on the macro-autophagy machinery and an exosomal branch that relies on the ESCRT machinery. Interestingly, emerging evidence supports an additional role of EVs in intercellular communication, a function by which they would contribute to many aspects of physiology and disease^27^. It is therefore tempting to speculate that the targeting of activated CASP8 to exosomes would extend beyond intracellular detoxification and may also contribute to intercellular communication during TNF-driven inflammatory responses^65,66^. Supporting this idea, several studies have reported immunomodulatory and cytotoxic effects of EVs derived from TNF-stimulated cells^67–69^. However, the identity of the responsible cargo and the underlying molecular mechanisms have remained unclear. The identification of activated CASP8, along with other components of the TNFR1 Complex II in exosomes, now provides compelling candidates. Indeed, the exosomal transfer of activated CASP8 may modulate cell death sensitivity in recipient cells. In addition to its cytotoxic activity, TNFR1 Complex II has been described as a ubiquitinated signaling platform capable of triggering NF-κB and MAPK activation^70,71^. Given the observed enrichment of M1-linked ubiquitin chains within exosomes released by TNF-treated cells - and the central role of these chains in triggering NF-κB signaling - we further hypothesize that exosomal transfer of TNFR1 Complex II components could activate this pathway in recipient cells. Such a mechanism would provide a ligand-independent means of propagating TNF-driven inflammatory signaling between cells. This model may potentially also explain why two independent and non-redundant detoxification pathways exist to neutralize the same toxic cargo. Alternatively, it is conceivable that the amount of activated CASP8 generated upon TNF sensing simply exceeds the capacity of each individual pathway, necessitating the combined activity of both detoxification branches.

Detoxification of TNFR1 Complex II by LC3-independent macro-autophagy was shown to rely on the selective autophagy receptor TAX1BP1, which associates with the complex by binding to the M1-ubiquitin chains conjugated to RIPK1^72,73^ and subsequently directs the in situ formation of an autophagosome around the complex^12–14^. Interestingly, we found that the targeting of activated CASP8 into ILVs also requires TAX1BP1, which is in line with the recently reported ability of TAX1BP1 to simultaneously target ferritin for both LC3-independent macro-autophagy and eMI^15,18^. Moreover, we found that the ILV-dependent detoxification of activated CASP8 additionally requires RIPK1 and M1-ubiquitination, suggestive of a targeting process comparable to the one used during LC3-independent macro-autophagy. In accordance with the scaffolding role of the chaperone HSC70 for eMI^19,26^, we found that the selective targeting of activated CASP8 additionally relied on HSC70. Nevertheless, the transfer of activated CASP8 into ILVs by HSC70 was independent of its ATPase activity and of its direct interaction with RIPK1, distinguishing this function of HSC70 with its recently reported role as RHIM-amyloidase counteracting TNF-induced necroptosis^50^. How TAX1BP1 and HSC70 cooperate to promote the engulfment of activated CASP8 into ILVs is currently unclear. HSC70 was shown to specifically recognize protein cargoes containing a KFERQ pentapeptide motif^19,74^. This suggests that one or multiple KFERQ motifs in activated CASP8 (or contained within TNFR1 Complex II components) would be required to promote ILV-dependent detoxification and subsequent transfer to exosomes. The identification of such critical KFERQ-containing protein (or proteins) will, however, be challenging. Indeed, numerous, potentially redundant, KFERQ motifs are present in multiple TNFR1 Complex II components. Moreover, non-canonical KFERQ-like motifs can additionally be generated via post-translational modifications of putative sequences, making the identification of this motif even more demanding. Finally, it is also possible that HSC70 binds to activated CASP8 or TNFR1 Complex II via a KFERQ-independent interaction^50^.

In this study, we report on the identification and characterization of an additional cell death checkpoint in the TNF pathway. It has been postulated that these checkpoints serve as fuse sensors to detect and react to pathogenic interference with host immune responses^4^. Indeed, a plethora of microbial effector proteins allows pathogens to temper with host immune signaling pathways and cellular anti-microbial mechanisms that would otherwise promote their elimination. In turn, cell death evolved as a backup response from the host to ensure proper immune responses in conditions of microbial hijacking. Due to the variety of processes it regulates, the ESCRT machinery is very often targeted by intracellular pathogens, including bacteria, viruses, and intracellular parasites. These microbes either inhibit the ESCRTs to prevent their elimination or instead require and hijack the ESCRT machinery as part of their replication cycle. By interfering with ESCRT components, pathogens would inadvertently inactivate the identified ILV-dependent checkpoint and trigger the death of the infected cells, ultimately favoring the elimination of the microbial invaders. Remarkably, we found that the *S*. Typhimurium effector protein SopB activates TNF cytotoxicity via the turnover of phosphoinositides, thereby interfering with ESCRT components that rely on these phosphorylated lipids. Similarly, we found that the *Mtb* effector complex EsxG-EsxH switches the TNF response from survival to death by inhibiting the ESCRT-0 component HGS. By targeting the ESCRT machinery, these effector proteins inhibit the transfer of activated CASP8 from the cytosol to endosomes, thereby causing the lethal cytosolic accumulation of the complex. While it is known that the control of infections with these pathogens relies on TNF and programmed death of the host cell^51,52,54,55,75^, the cause of death had remained largely unknown. This study now provides a mechanistic explanation as to how these pathogens cause TNF cytotoxicity. In line with this idea, deletion of SopB has been shown to increase the pathogenicity of *S*. Typhimurium strains (ΔSopB)^76^, as demonstrated by increased bacterial loads in multiple organs and in a higher lethality rate in mice^76^. It remains to be determined if the increased virulence of these ΔSopB strains originates from a reduced sensitivity of the infected cells to TNF-mediated killing.

While transient inactivation of a cell death checkpoint can be beneficial in the context of infection, its constitutive inhibition is instead detrimental and causes pathogenesis^1^. Interestingly, numerous human mutations in components of the ESCRT machinery have been identified across patients presenting with diverse clinical symptoms^77^. Because the ESCRT components fulfil many functions beyond ILV formation, pinpointing the specific contribution of impaired TNFR1 Complex II detoxification to these disease manifestations will be challenging. Nevertheless, our findings indicate that excessive TNF-induced cell death may contribute to the pathology in at least a subset of cases. Notably, together with recent reports, our data suggest a more specific role of PTPN23 in directing cytosolic cargo into ILVs^15,29^. Homozygous or compound heterozygous mutations in PTPN23 cause the neurologic disorder NEDBASS, characterized by neurodevelopmental delay, structural brain abnormalities, impaired intellectual development with delayed or absent speech, axial hypotonia, and peripheral spasticity^78–80^. It will be important to determine whether these mutations disrupt the ILV-dependent detoxification of activated CASP8 and whether uncontrolled TNF-induced cell death contributes to disease progression. If so, anti-TNF biologics could represent a promising therapeutic avenue for affected patients.

## Methods

### Antibodies and reagents

The following primary antibodies were used for immunoblot analysis:

**Table:** 

Antibody	Source	Identifier	Concentration used
A20	Santa Cruz Biotechnology	sc-166692	1/1000
ACTIN-HRP	Proteintech	HRP-66009	1/10.000
ALIX	Santa Cruz Biotechnology	sc-271975	1/200
ATG9A	Abcam	Ab108338	1/1000
CALNEXIN	Abcam	ab10286	1/1000
CASPASE 3	Cell Signaling Technology	#9662	1/1000
CASPASE 8	Abnova	MAB3429	1/1000
CASPASE 8	Prof. P. Krammer		1/1000
cFLIP	Enzo Life Sciences	ALX-804-127	1/1000
CHMP4B	Abcam	ab105767	1/1000
cIAP1	Enzo Life Sciences	ALX-803-335	1/1000
Cleaved CASPASE 8	Abcam	ab75852	1/1000
Cleaved PARP	Cell Signaling Technology	#9544	1/1000
FLAG	Sigma Aldrich	A8592	1/7500
FLOTILIN-1	Abcam	ab133497	1/10.000
HA	Covance	MMS-101R	1/1000
HOIP	Abcam	ab46322	1/1000
HSC70	Santa Cruz Biotechnology	sc-24	1/1000
IκBα	Cell Signaling Technology	#9242	1/1000
phospho-IκBα	Cell Signaling Technology	#9246	1/1000
JNK	Cell Signaling Technology	#9252	1/1000
Phospho-JNK	Invitrogen	44682G	1/1000
LAMP2A	Abcam	ab18528	1/1000
LC3	Sigma Aldrich	L7543	1/1000
M1-UBIQUITIN	Life Sensors	AB130	1/2000
MLKL	Merck Millipore	# MABC604	1/1000
pS345 MLKL	Cell Signaling Technology	#37333	1/1000
NEMO	Pasparakis lab	Vlantis et al. ^87^	1/1000
P38	Cell Signaling Technology	#9212	1/1000
Phospho-P38	Cell Signaling Technology	#9211	1/1000
RAB5	Abcam	ab18211	1/1000
RAB7	Cell Signaling Technology	#9367	1/2000
RFP	MBL	PM005	1/1000
RIPK1	Cell Signaling Technology	#3493	1/2000
RIPK1	Santa Cruz Biotechnology	sc-7881	1/1000
Phoshpo-S166/T169 RIPK1	ThermoFisher	Custom made	1/1000
RIPK3	Cell Signaling Technology	#10188	1/1000
pT231/S232 RIPK3	Cell Signaling Technology	#57220	1/1000
SHARPIN	Proteintech	14626-1-AP	1/1000
TAK1	Santa Cruz Biotechnology	sc-7162	1/1000
TAX1BP1	Abcam	ab176572	1/1000
TBK1	Cell Signaling Technology	#3013	1/1000
TNFR1	Cell Signaling Technology	#13377	1/1000
TNFR1	Santa Cruz Biotechnology	sc-8436	1/1000
TOLLIP	Abcam	ab187198	1/1000
TRADD	Cell Signaling Technology	#3694	1/1000
TRAF1	Santa Cruz Biotechnology	Sc-7186	1/1000
TRAF2	Cell Signaling Technology	#4724	1/1000
TSG101	Santa Cruz Biotechnology	sc-7964	1/1000
V5-HRP	Invitrogen	R961-25	1/5000
VPS28	Santa Cruz Biotechnology	sc-166537	1/100
VPS36	Novus Bio	NBP2-13519	1/1000
VSV	Sigma Aldrich	A 5977	1/3000
β-TUBULIN-HRP	Abcam	ab21058	1/10000

The following secondary antibodies were used for detection: donkey anti-rabbit IgG-HRP (NA934, GE Healthcare), sheep anti-mouse IgG-HRP (NA931, GE Healthcare), and goat anti-rat IgG (NA935, GE Healthcare).

The following recombinant proteins were used: λPPase (P0753L, New England Biolabs). Recombinant mTNF, hTNF, FLAG-hTNF, and Cas9-GFP were produced by the VIB protein service facility. Recombinant USP21 was produced in-house.

The following compounds were used:

**Table:** 

Compound	Source	Identifier	Concentration used
Bafilomycin A1	Sigma Aldrich	B1793	100 nM
BAPTA-AM	Invitrogen	B1205	10 µM
CHX	Sigma-Aldrich	C7698	2 µg/mL
Doxycycline (Dox)	Sigma-Aldrich	D9891	1 µg/mL
Dyngo-4a	Abcam	Ab120689	50 µM
HOIPIN-8 (HOIP inhibitor)	Axon MedChem	2972	30 µM
Nec1s	Antwerp University	UAMC02197	10 µM
SAR405 (VPS34 inhibitor)	MedChemExpress	HY-12481	20 µM for HT1080 cells 1 µM for MEFs
9-epimer-11,12-dihydro-(5Z)−7-oxozeanol (TAK1-inhibitor)	Analyticon Discovery	NP-0009245	1 µM
DC-U4106 (USP8 inhibitor)	MedChemExpress	HY-150505	2 µM
VER-155008 (HSC70 inhibitor)	MedChemExpresss	HY-10941	50 µM, unless indicated otherwise
zVAD-fmk	Bachem Biochemica	BACE4026865.0005	50 µM

### Plasmids

Guide RNA sequences were cloned into lentiCRISPRv2 using a BsmBI-mediated Golden Gate cloning strategy, as previously described^81^. In short, to each sgRNA sequence, BsmBI recognition sites were appended along with the appropriate overhang sequences (underlined). In the case that the sgRNA target sequence does not start with a G(uanine), a G should be added in the top oligo, and a C should be added in the bottom oligo at the indicated sites. Guanine is the transcription start site following the U6 promoter. The final oligo sequences are: top oligo – 5′-CACC(G)(20 bp guide sequence)-3′ and bottom oligo – 5′AAAC(reverse complement 20 bp guide sequence)(C)-3′. Oligo’s were subsequently annealed and phosphorylated with T4 PNK (M4101, Promega) according to the manufacturer’s instructions. Oligo’s were finally diluted and combined with the lentiCRISPRv2 backbone in a BsmBI-mediated (FD0454, Thermo Fisher Scientific) Golden Gate cloning strategy using T4 DNA ligase (M1801) in tango buffer (BY5, Thermo Fisher Scientific). The sequences encoding FADD-GFP, RAB5, RAB7, RIPK1, TNFR1, the dominant negative DYNAMIN-1 S45N (DYN1^S45N^) mutant, the dominant-negative C-terminal fragment of AP1080 (AP180^C-term^) and the effector proteins SopB and EsxG-EsxH were ordered as synthetic DNA fragments and cloned into pENTR™3C (A10464, ThermoFisher) using the cloneEZ PCR cloning kit (L00339, GenScript). Mutated sequences were obtained with the QuickChange II Site-Directed Mutagenesis kit (#200524, Agilent Technologies), according to the manufacturer’s instructions. Sequences from the pENTR™3C plasmids were then transferred into the pSIN-3x-HA or pSIN-V5 (Puro or Blast) destination vectors using the Gateway™ LR Clonase™ II Enzyme mix (11791020, Invitrogen). For the generation of inducible knockout cell, the Lenti-iCas9-Neo plasmid (Addgene #85400) and tet-pLKO-sgRNA-puro (Addgene #104321) were used. Guide sequences were cloned into tet-pLKO-sgRNA-puro as described^82^.

### Cell lines

MEFs, HT1080 (ATCC CCL-121) and HEK293T (ATCC CRL-3216) cells were cultured in Dulbecco’s modified Eagle’s medium supplemented with 10% fetal calf serum, L-glutamine (200 mM) and sodium pyruvate (400 mM) in normoxic conditions (5% CO_2_). *Atg9a*^−/−^, *Ripk1*^*−/−*^, and *Tnfr1*^*−/−*^ MEFs were described earlier^12,83,84^. CRISPR-mediated knockout cell lines were obtained either via transduction with LentiCRISPRv2-generated lentiviral vectors or via electroporation of Cas9-RNPs. In brief, lentiviral supernatants were harvested 48 h following co-transfection of psPAX2, p-CMV-VSV-G, and lentiCRISPRv2 plasmids in HEK293T cells via Calcium Phosphate transfection. Lentiviral supernatant was subsequently used to transduce target cells in the presence of 8 µg/mL polybrene (H9268, Sigma-Aldrich). The next day, transduced cells were selected with 2 µg/mL puromycin (P-7255, Sigma-Aldrich) for the duration of 1 week. In the case of electroporation, 0.2 nmol crRNA (IDT) and 0.2 nmol tracrRNA (#1072532, IDT) were mixed, denatured at 95 °C for 5 min. and re-annealed for 20 min at room temperature. Alt-R® CRISPR-Cas9 negative control crRNA #1 (#1072544, IDT) was used as the NT control. Subsequently, 20 µg Cas9-eGFP was added, and the mixture was incubated for 10 min. at room temperature. Next, Cas9-RNPs were mixed with 0.2 nmol electroporation enhancer (1075915, IDT) and incubated with 1.25 × 10^6^ cells in a total volume of 100 µL Opti-MEM. Finally, cells were electroporated using the NEPA21 electroporator (MEFs: poring pulse – 140 V, length 10 ms, interval 50 ms, 2 pulses, 10% decay, + polarity – transfer pulse – 20 V, length 50 ms, interval 50 ms, 5 pulses, 40% decay, ± polarity; HT1080 cells: poring pulse – 200 V, length 5 ms, interval 50 ms, 2 pulses, 10% decay, + polarity – transfer pulse − 20 V, length 50 ms, interval 50 ms, 5 pulses, 40% decay, ± polarity). The next day, GFP^+^ cells were sorted using the BD FACSMelody sorter. For the generation of inducible knockout cells, cells were first transduced with Lenti-iCas9-Neo-generated lentiviral vectors. Transduced cells were subsequently treated with 1 µg/mL Doxycycline overnight. GFP^+^ cells were sorted using the BD FACSMelody sorter. The sorted cells were subsequently expanded and transduced with tet-pLKO-sgRNA-puro-generated lentiviral vectors containing gene-specific guide sequences.

The following CRISPR guide sequences were used to generate gene-specific knockouts:

**Table:** 

Gene	Sequence	Type
*ATG9A*	GTGACCTTGACGGGTTCAGT	Cas9-RNP
*CASP3*	AATGGACTCTGGAATATCCC	Cas9-RNP
*CASP7*	GGTACAAACGAGGACCGGTC	Cas9-RNP
*Chmp4b*	AAGCAGCTGGCACAAATTGA	Cas9-RNP
*Fadd*	CCTGTCGGGCAACGATCTGA	Cas9-RNP
*Hgs*	CTCCAACAGAAGCTGGCTGG	Cas9-RNP
*Hspa8 (Hsc70)*	GAAAGCAACATAGCTTGGCG	Cas9-RNP
*HSPA8 (HSC70)*	GACACTGAACGGTTGATGTT	Cas9-RNP
*Lamp2*	CCTGACAAGGCGACACACGA	Cas9-RNP
Non-targeting	CTGAAAAAGGAAGGAGTTGA	LentiCRISPRv2/tet-pLKO-sgRNA-puro
*Pdcd6ip (Alix)*	AGATGCCATCATAGCTAAGC	Cas9-RNP
*Ptpn23*	CTACAGAGTCGGGTGCCCAT	Cas9-RNP
*Ripk1*	TGTGAAAGTCACGATCAACG	Cas9-RNP
*RIPK1*	TACACATCCGACTTCTCTGT	Cas9-RNP
*RNF31 (HOIP)*	TCCATGGAACAGGTGGTCAC	Cas9-RNP
*Tax1bp1*	TATACGGAGTTAAGGTGTAA	Cas9-RNP
*TAX1BP1*	CCACATCCAAAAGATTGGGT	Cas9-RNP
*Tsg101*	AGGGAGCTGGTGAACCTCAC	Cas9-RNP/tet-pLKO-sgRNA-puro
*TSG101*	AGCCCCAACAAGTCTGACTG	Cas9-RNP
*Usp8*	ATTAACGGCGAGAAGAGTGA	Cas9-RNP
*Vps25*	TACAGCCGAACGTGGACACC	LentiCRISPRv2
*Vps28*	TGATCCTCTCCATAGCAAGT	Cas9-RNP
*Vps36*	GGTGCGCGTCTACGACGGCG	LentiCRISPRv2

Other transduced cells were generated using the doxycycline-inducible pSIN 3×HA lentiviral vectors as described higher. Transduced cells were selected with 2 µg/mL puromycin (P-7255, Sigma-Aldrich) or 2 µg/mL blasticidin S (R210-01, Invitrogen) for the duration of 1 week.

### Cell death assay

For cell death assays in MEFs or HT1080 cells, cells were seeded the day before the experiment. Transduced cells were treated with 2 µg/mL doxycycline 48 h prior to stimulation to induce expression. Before stimulation, cells were pre-treated with the indicated compounds for 30 min. and then stimulated with TNF in the presence of 2.5 μM SYTOX® Green Nucleic Acid Stain (S7020, Molecular Probes) and 20 μM Ac-DEVD-AMC (3171-V, PeptaNova). SytoxGreen intensity and CASP3 activation were measured at intervals of 1 h using a Fluostar Omega fluorescence plate reader, with an excitation filter of 485 nm (SytoxGreen) or 360 nm (Ac-DEVD-AMC), an emission filter of 520 nm (SytoxGreen) or 460 nm (Ac-DEVD-AMC), gains set at 1100, 20 flashes per well, and orbital averaging with a diameter of 3 mm. The maximal fluorescence is obtained by full permeabilization of the cells by using Triton X-100 at a final concentration of 0.1%. The percentage of treatment-induced cell death was calculated as (induced fluorescence − background fluorescence)/ (maximum fluorescence − background fluorescence) × 100. The fold-induction of CASP3 activity was determined as (induced fluorescence)/(background fluorescence at 0 h).

### TNFR1 Complex I immunoprecipitation

TNFR1 Complex I was isolated as described^85^. In brief, MEFs were pre-treated as indicated in the figure legends and subsequently stimulated (or not) with 1 µg/mL FLAG-hTNF for the indicated amount of time. For TNFR1 immunoprecipitations under steady-state conditions, cells were treated with ice-cold medium containing 1 µg/mL FLAG-hTNF. Cells were then incubated on ice for 2 h. Cells were then washed twice in ice-cold PBS and lysed in NP-40 lysis buffer (10% glycerol, 1% NP-40, 150 mM NaCl, and 10 mM Tris-HCl, pH 8) supplemented with PhosSTOP™ tablets (4906845001, Roche) and cOmplete™, EDTA-free Protease Inhibitor Cocktail (4693132001, Roche). The cell lysates were then cleared by centrifugation at 20,000 × *g* for 15 min at 4 °C, and the supernatants were incubated overnight with ANTI-FLAG M2 Affinity Gel (A2220, Sigma-Aldrich). The next day, the beads were washed three times in NP-40 lysis buffer. Lysates or beads were then resuspended in Laemmli buffer for direct analysis. However, when indicated, protein complexes were additionally deubiquitinated (by USP21 treatment) post-IP. To do so, beads were resuspended after the final washing step in DUB-buffer (50 mM Tris-HCl, pH 8, 50 mM NaCl, 5 mM DTT, and 1 mM MnCl_2_). Subsequently, 1.91 μg USP21 was added, and the enzymatic reaction was allowed to proceed for 30 min at 37 °C. Finally, the reaction was quenched by the addition of 5× Laemmli buffer. Samples were subsequently analyzed by immunoblotting.

### TNFR1 Complex II immunoprecipitation from cell lysates

MEFs were seeded the day before the experiment. MEFs were then pre-treated with zVAD before stimulation with 20 ng/mL mTNF for the indicated amount of time. Cells were then washed twice in ice-cold PBS and lysed in NP-40 lysis buffer (10% glycerol, 1% NP-40, 150 mM NaCl, and 10 mM Tris-HCl, pH 8) supplemented with PhosSTOP™ tablets (4906845001, Roche) and cOmplete™, EDTA-free Protease Inhibitor Cocktail (4693132001, Roche). The cell lysates were then cleared by centrifugation at 20,000 × *g* for 15 min at 4 °C, and the supernatants were incubated overnight with Protein G Sepharose® 4 Fast Flow beads (GE17-0618-05, GE Healthcare) in combination with homemade polyclonal rabbit anti-mouse CASPASE-8 antibodies. The next day, the beads were washed three times in lysis buffer and subsequently resuspended in Laemmli buffer for direct analysis via immunoblotting.

### Immunofluorescence

HT1080 cells transduced with the doxycycline-inducible lentiviral vector encoding the mCherry-tagged mutant RAB5^Q79L^ were treated with 2 µg/mL doxycycline for 48 h to induce expression. On the day of stimulation, cells were pre-treated for 30 min as indicated in the figure legends and stimulated (or not) with 2 µg/mL hTNF. FADD-deficient MEFs transduced with the doxycycline-inducible lentiviral vector encoding FADD-GFP were treated with 100 ng/mL doxycycline for 24 h to induce expression. Cells were then stimulated with 20 ng/mL mTNF. Subsequently, cells were fixed for 10 min at 37 °C with 2% PFA. Next, cells were washed three times with PBS, after which cells were permeabilized with 0.1% Triton X-100 in PBS for 15 min at room temperature. Samples were then blocked with goat serum diluted 1/500 in PBS containing 0.1% Triton X-100 for 1 h at room temperature. Finally, cells were incubated overnight at 4 °C with primary antibodies diluted in PBS containing 0.1% Triton X-100: cleaved CASPASE-8 antibody (#9496, Cell Signaling Technology, 1/400), EEA1 (ab2900, Abcam, 1/500), and LAMP1 (sc-20011, Santa Cruz Biotechnology, 1/500). The next day, samples were washed three times with PBS. Subsequently, samples were incubated for 90 min at room temperature in the dark with secondary antibody (35553, ThermoFisher, 1/1000; 35563, ThermoFisher, 1/1000; or 35513, ThermoFisher, 1/1000) and Hoechst (B2261, Sigma-Aldrich, 1/1000) diluted in PBS supplemented with 0.1% Triton X-100. Finally, samples were washed three times with PBS and stored at 4 °C prior to imaging. Ready-to-image samples were analyzed on the LSM880 Airy Scan confocal microscope (Zeiss) in super-resolution FAST mode with a 63× Plan-apochromat (1.40 oil DIC M27) objective (pixel size 0.04 µm × 0.04 µm). Z-stacks were taken according to the Nyquist sampling criterion (interval 0.16 µm). Cl. CASP8 immunofluorescence (DyLight™ 488) and FADD-GFP fluorescence were imaged after excitation with the AR laser. Hoechst was excited with a 405 nm diode laser. Both dyes were detected after passage through a double band pass filter with specs: BP 420-480+BP495-550. The 561 nm laser was used to detect the RAB5 Q79L mutant (mCherry). The emission filter used was BP555-620+LP645. The 633 nm laser used to detect LAMP1 and EEA1 immunofluorescence (DyLight™ 633). The emission filter used was BP570-620+LP645. All signals were captured by the AiryScan detector. Images acquired in FAST mode were processed using ZEN software (Zeiss, Jena). The processing included pixel reassignment and default Wiener filtering. AiryScan-processed images were finally subjected to extended depth of focus (EDF) projection using the EDF module (maximum projection) on ZEN Blue software. Cl. CASP8 puncta were detected using the ArivisPro 4.2.2. Software (Zeiss). A blobfinder algorithm was built using the following settings: average diameter 0.2 µm, probability threshold 35%, and split sensitivity 50%. The total amount of puncta per cell were quantified. The mCherry signal was used to determine the relative position of puncta to the endosomal membrane. Puncta located inside endosomes were quantified. The ratio of puncta inside endosomes to the total amount of puncta was made for each individual cell. The number and size of mCherry-RAB5^Q79L+^ endosomes were determined using a blobfinder algorithm with the following settings: average diameter 1 µm, probability threshold 20%, and split sensitivity 65%. 3D reconstruction images of Z-stacks were made using the ArivisPro 4.2.2. software (Zeiss) using volumetric 3D reconstruction, threshold opacity, and 4D clipping along a selected XZ plane. EEA1^+^ endosomes ArivisPro 4.2.2. Software (Zeiss). The number and size of A blobfinder algorithm was built to detect LAMP1^+^ lysosomes using the following settings: average diameter 0.5 µm, probability threshold 50%, and split sensitivity 100%. Total amount of Cl. CASP8 puncta per image were quantified, as well as the amount of puncta that colocalized with LAMP1^+^ lysosomes. The ratio of Cl. CASP8 puncta that colocalized with LAMP1^+^ lysosomes to the total amount of puncta was made for each individual image.

### Isolation and purification of extracellular vesicles (EVs)

Prior to stimulation, cells were washed twice with pre-warmed Dulbecco’s modified Eagle’s medium without supplements to remove EVs present in the fetal calf serum. HT1080 cells were then pre-treated for 30 min as indicated and stimulated in EV-depleted medium calf serum for the indicated duration with 2 µg/mL hTNF. EV-depleted medium consisted of Dulbecco’s modified Eagle’s medium supplemented with L-glutamine (200 mM), sodium pyruvate (400 mM), and 10% fetal calf serum that was depleted of EVs by ultracentrifugation for 16 h at 100,000 × *g* at 4 °C.

Following stimulation, the medium was harvested, and cells were lysed in Laemmli buffer. The medium was then centrifuged at 250 × *g* for 5 min at 4 °C to remove dead cells. The supernatant was subsequently centrifuged again at 1000 × *g* for 10 min at 4 °C to remove cell debris. Particle concentration and average particle size were analyzed by Nanoparticle Tracking Analysis (ZetaView®, Particle Metrix) using the following parameters: 30 fps, shutter 70–100, min. bright 30, max. area 1000, min. area 10 and tracelength 15. The cleared supernatant was subsequently subjected to DGUC, SEC, or regular ultracentrifugation.

DGUC was performed as previously described^86^. In short, the cleared medium was first concentrated to a volume of 1 mL using a Centricon® Plus-70 MWCO 10 kDa Centrifugal Filter Unit (UFC701008, Millipore). The concentrated medium was then loaded on top of an OptiPrep™ Density Gradient. The discontinuous iodixanol gradient was prepared by layering 4 mL of 40%, 4 mL of 20%, 4 mL of 10%, and 3.5 mL of 5% iodixanol on top of each other in a 16.8 mL open top polyallomer tube (337986, Beckman Coulter). Iodixanol solutions were made by mixing appropriate amounts of a homogenization buffer (0.25 M sucrose, 1 mM EDTA, 10 mM Tris-HCl, [pH 7.4]) and a 50% iodixanol working solution. The working solution was prepared by combining a working solution buffer (0.25 M sucrose, 6 mM EDTA, 60 Mm Tris-HCl, [pH 7.4]) and a stock solution of OptiPrep (60% (w/v) aqueous iodixanol solution). The cleared and concentrated medium was then overlaid on top of the gradient, followed by 18 h ultracentrifugation at 100,000 × *g* and 4 °C using a SW32.1 Ti rotor (Beckman Coulter). Afterwards, 16 DGUC fractions of 1 mL were collected from the top of the gradient (EV-enriched fractions 9 and 10—corresponding to 1.09–1.11 g/mL densities). Fractions were then diluted to 15 mL with PBS, followed by a 3 h ultracentrifugation at 100,000 × *g* and 4 °C using a SW32.1 Ti rotor. Finally, the supernatant was removed, and the invisible pellet of each fraction was resuspended in Laemmli buffer.

In the case of SEC, the cleared medium was first concentrated to a volume of 2 mL using a Centricon® Plus-70 MWCO 10 kDa Centrifugal Filter Unit (UFC701008, Millipore). The concentrated medium was then loaded on top of a gel filtration column containing 10 mL 2% BCL agarose bead standard (50–150 µm) (A-1021S, Agarose Bead Technology), and subsequently 1 mL fractions were collected. Fractions were then concentrated using Amicon® Ultra-0.5 MWCO 10 kDa Centrifugal Filter Unit (UFC501096, Millipore). Concentrated samples were then lysed using Laemmli buffer.

In the case of regular ultracentrifugation, the cleared supernatant was then ultracentrifuged at 100,000 × *g* for 70 min at 4 °C to pellet EVs. Supernatant was removed, and the invisible pellet was subsequently resuspended and washed in PBS. Then, the resuspended pellet was ultracentrifuged again at 100,000 × *g* for 70 min at 4 °C. The supernatant was removed, and the invisible pellet was lysed in Laemmli buffer.

### TNFR1 Complex II immunoprecipitation from EVs

Following isolation of EVs by SEC, as described earlier, lysis buffer (20 mM Tris-HCl pH 7.4, 150 mM NaCl, 0.2% NP-40, 10% glycerol) supplemented with PhosSTOP™ tablets (4906845001, Roche) and cOmplete™, EDTA-free Protease Inhibitor Cocktail (4693132001, Roche) was added to the concentrated EVs. Lysed EVs were then incubated overnight with Protein G Sepharose® 4 Fast Flow beads (GE17-0618-05, GE Healthcare) in combination with CASPASE-8 antibodies (sc-6136, Santa Cruz Biotechnology). The next day, the beads were washed three times in lysis buffer and subsequently resuspended in Laemmli buffer for direct analysis via immunoblotting.

### Transmission electron microscopy

For the morphological analysis of HT1080 cells expressing the dominant negative RAB5 Q79L mutant by transmission electron microscopy (TEM), cells were seeded on 12 mm cover slips and fixed for 3 h at room temperature with a buffer containing 4% PFA (15712-13, Electron Microscopy Services), 2.5% glutaraldehyde (16220, Electron Microscopy Services) and 0.1 M cacodylate (11652-54, Electron Microscopy Services). Samples were then subjected to secondary fixation with 1% osmium tetroxide (16220, Electron Microscopy Services) before contrasting with 1% uranyl acetate (02545, Structure Probe, Inc.). Samples were subsequently dehydrated with increasing amounts of ethanol before infiltration with increasing amounts of Lowicryl® HM20 resin (14340, Electron Microscopy Services) and polymerization at 65 °C. Ultrathin sections of 70 nm were obtained using the EM UC7 (Leica Microsystems) microtome and post-stained in 1% uranyl acetate (02545, Structure Probe, Inc.) and 3% lead citrate (17800, Electron Microscopy Services) in the EM AC20 (Leica Microsystems). Imaging was performed at 80 kV on the JEM-1400 Plus (JEOL).

For the morphological analysis of isolated EVs, EVs were first blotted on 400-mesh copper grids (CF400-Cu-50, Electron Microscopy Services), which were coated with 3 nm carbon and glow-discharged for 40 s at 15 mA right before sample loading. Blotted samples were dried and washed five times in water. Samples were then incubated for 1 min with 25% uranyl acetate replacement stain (22405, Electron Microscopy Services). Excess stain was removed, and grids were air-dried for 1 h before imaging. Imaging was performed at 80 kV on the JEM-1400 Plus (JEOL).

### Reporting summary

Further information on research design is available in the Nature Portfolio Reporting Summary linked to this article.

## Supplementary information

Supplementary Information

Reporting Summary

Transparent Peer Review File

## Source data

Source Data

Source Data - Uncropped Blots

## Data Availability

Source data are provided with this paper. Requests for unprocessed microscopy images, resources, reagents, plasmids, cell lines used in this study, and additional information should be directed to the lead contact, Mathieu JM Bertrand (mathieu.bertrand@irc.vib-ugent.be). Source data are provided with this paper.

## References

[1] van Loo, G. & Bertrand, M. J. M. Death by TNF: a road to inflammation. *Nat. Rev. Immunol.* **23**, 289–303 (2023). 10.1038/s41577-022-00792-3 PMC9665039 36380021

[2] Peltzer, N. & Walczak, H. Cell death and inflammation—a vital but dangerous liaison. *Trends Immunol.* **40**, 387–402 (2019). 10.1016/j.it.2019.03.006 31003931

[3] Newton, K., Dixit, V. M. & Kayagaki, N. Dying cells fan the flames of inflammation. *Science* **374**, 1–6 (2021). 10.1126/science.abi5934 34822265

[4] Huyghe, J., Priem, D. & Bertrand, M. J. M. Cell death checkpoints in the TNF pathway. *Trends Immunol.* **44**, 628–643 (2023). 10.1016/j.it.2023.05.007 37357102

[5] Peterson, L. W. et al. RIPK1-dependent apoptosis bypasses pathogen blockade of innate signaling to promote immune defense. *J. Exp. Med.* **214**, 3171–3182 (2017). 10.1084/jem.20170347 PMC5679171 28855241

[6] Bertrand, M. J. M. et al. cIAP1 and cIAP2 facilitate cancer cell survival by functioning as E3 ligases that promote RIP1 ubiquitination. *Mol. Cell* **30**, 689–700 (2008). 10.1016/j.molcel.2008.05.014 18570872

[7] Haas, T. L. et al. Recruitment of the linear ubiquitin chain assembly complex stabilizes the TNF-R1 signaling complex and is required for TNF-mediated gene induction. *Mol. Cell* **36**, 831–844 (2009). 10.1016/j.molcel.2009.10.013 20005846

[8] Kanayama, A. et al. TAB2 and TAB3 activate the NF-κB pathway through binding to polyubiquitin chains. *Mol. Cell* **15**, 535–548 (2004). 10.1016/j.molcel.2004.08.008 15327770

[9] Rahighi, S. et al. Specific recognition of linear ubiquitin chains by NEMO is important for NF-κB activation. *Cell* **136**, 1098–1109 (2009). 10.1016/j.cell.2009.03.007 19303852

[10] Micheau, O. & Tschopp, J. Induction of TNF receptor I-mediated apoptosis via two sequential signaling complexes. *Cell* **114**, 181–190 (2003). 10.1016/s0092-8674(03)00521-x 12887920

[11] Ye, K., Chen, Z. & Xu, Y. The double-edged functions of necroptosis. *Cell Death Dis.* **14**, 163 (2023). 10.1038/s41419-023-05691-6 PMC9969390 36849530

[12] Huyghe, J. et al. ATG9A prevents TNF cytotoxicity by an unconventional lysosomal targeting pathway. *Science* **378**, 1201–1207 (2022). 10.1126/science.add6967 36520901

[13] Priem, D., Huyghe, J., Bertrand, M. J. M., Priem, D. & Huyghe, J. LC3-independent autophagy is vital to prevent TNF cytotoxicity. *Autophagy* **19**, 2585–2589 (2023). 10.1080/15548627.2023.2197760 PMC10392734 37014272

[14] Yang, Y. & Klionsky, D. J. A novel role of ATG9A and RB1CC1/FIP200 in mediating cell-death checkpoints to repress TNF cytotoxicity. *Autophagy* **19**, 1617–1618 (2023). 10.1080/15548627.2023.2187609 PMC10262787 36892222

[15] Goodwin, J. M. et al. Autophagy-independent lysosomal targeting regulated by ULK1/2-FIP200 and ATG9. *Cell Rep.* **20**, 2341–2356 (2017). 10.1016/j.celrep.2017.08.034 PMC5699710 28877469

[16] Mejlvang, J. et al. Starvation induces rapid degradation of selective autophagy receptors by endosomal microautophagy. *J. Cell Biol.* **217**, 3640–3655 (2018). 10.1083/jcb.201711002 PMC6168274 30018090

[17] Olsvik, H. L. et al. Endosomal microautophagy is an integrated part of the autophagic response to amino acid starvation. *Autophagy* **15**, 182–183 (2019). 10.1080/15548627.2018.1532265 PMC6287684 30295124

[18] Ohshima, T., Yamamoto, H., Sakamaki, Y., Saito, C. & Mizushima, N. NCOA4 drives ferritin phase separation to facilitate macroferritinophagy and microferritinophagy. *J. Cell Biol.* **221**, e202203102 (2022). 10.1083/jcb.202203102 PMC9452830 36066504

[19] Krause, G. J. et al. Molecular determinants of the crosstalk between endosomal microautophagy and chaperone-mediated autophagy. *Cell Rep.* **42**, 113529 (2023). 10.1016/j.celrep.2023.113529 PMC10807933 38060380

[20] Kaushik, S. & Cuervo, A. M. The coming of age of chaperone-mediated autophagy. *Nat. Rev. Mol. Cell Biol.* **19**, 365–381 (2018). 10.1038/s41580-018-0001-6 PMC6399518 29626215

[21] Oku, M. et al. Evidence for ESCRT- and clathrin-dependent microautophagy. *J. Cell Biol.* **216**, 3263–3274 (2017). 10.1083/jcb.201611029 PMC5626533 28838958

[22] Iwama, R. & Ohsumi, Y. Analysis of autophagy activated during changes in carbon source availability in yeast cells. *J. Biol. Chem.* **294**, 5590–5603 (2019). 10.1074/jbc.RA118.005698 PMC6462502 30755486

[23] Agarraberes, F. A., Terlecky, S. R. & Dice, J. F. An intralysosomal hsp70 is required for a selective pathway of lysosomal protein degradation. *J. Cell Biol.* **137**, 825–834 (1997). 10.1083/jcb.137.4.825 PMC2139836 9151685

[24] Agarraberes, F. A. & Dice, J. F. A molecular chaperone complex at the lysosomal membrane is required for protein translocation. *J. Cell Sci.* **114**, 2491–2499 (2001). 10.1242/jcs.114.13.2491 11559757

[25] Chiang, H. L., Terlecky, S. R., Plant, C. P. & Dice, J. F. A role for a 70-kilodalton heat shock protein in lysosomal degradation of intracellular proteins. *Science* **246**, 382–385 (1989). 10.1126/science.2799391 2799391

[26] Sahu, R. et al. Microautophagy of cytosolic proteins by late endosomes. *Dev. Cell* **20**, 131–139 (2011). 10.1016/j.devcel.2010.12.003 PMC3025279 21238931

[27] Kalluri, R. & LeBleu, V. S. The biology, function, and biomedical applications of exosomes. *Science* **367**, eaau6977 (2020). 10.1126/science.aau6977 PMC7717626 32029601

[28] Song, D. et al. PTPN23-dependent ESCRT machinery functions as a cell death checkpoint. *Nat. Commun.* **15**, 10364 (2024). 10.1038/s41467-024-54749-2 PMC11604704 39609437

[29] Men, Y. et al. ESCRT-I and PTPN23 mediate microautophagy of ubiquitylated tau aggregates. *J. Cell Biol.* **224**, e202406120 (2025). 10.1083/jcb.202406120 PMC11977513 40197510

[30] Doyotte, A., Mironov, A., McKenzie, E. & Woodman, P. The Bro1-related protein HD-PTP/PTPN23 is required for endosomal cargo sorting and multivesicular body morphogenesis. *Proc. Natl. Acad. Sci. USA* **105**, 6308–6313 (2008). 10.1073/pnas.0707601105 PMC2359801 18434552

[31] Gahloth, D. et al. The open architecture of HD-PTP phosphatase provides new insights into the mechanism of regulation of ESCRT function. *Sci. Rep.* **7**, 1–11 (2017). 10.1038/s41598-017-09467-9 PMC5567221 28831121

[32] Gong, Y. N. et al. ESCRT-III acts downstream of MLKL to regulate necroptotic cell death and its consequences. *Cell* **169**, 286–300 (2017). 10.1016/j.cell.2017.03.020 PMC5443414 28388412

[33] Jimenez, A. J. et al. ESCRT machinery is required for plasma membrane repair. *Science* **343**, 1247136 (2014). 10.1126/science.1247136 24482116

[34] Scheffer, L. L. et al. Mechanism of Ca^2+^-triggered ESCRT assembly and regulation of cell membrane repair. *Nat. Commun.* **5**, 5646 (2014). 10.1038/ncomms6646 PMC4333728 25534348

[35] Rühl, S. et al. ESCRT-dependent membrane repair negatively regulates pyroptosis downstream of GSDMD activation. *Science* **362**, 956–960 (2018). 10.1126/science.aar7607 30467171

[36] Van Antwerp, D. J., Martin, S. J., Kafri, T., Green, D. R. & Verma, I. M. Suppression of TNF-α-induced apoptosis by NF-κB. *Science* **274**, 787–789 (1996). 10.1126/science.274.5288.787 8864120

[37] Wang, L., Du, F. & Wang, X. TNF-α induces two distinct caspase-8 activation pathways. *Cell* **133**, 693–703 (2008). 10.1016/j.cell.2008.03.036 18485876

[38] Jeong, M. et al. USP8 suppresses death receptor-mediated apoptosis by enhancing FLIP L stability. *Oncogene* **36**, 458–470 (2017). 10.1038/onc.2016.215 27321185

[39] Zhen, Y. et al. ESCRT-mediated phagophore sealing during mitophagy. *Autophagy* **16**, 826–841 (2020). 10.1080/15548627.2019.1639301 PMC7158923 31366282

[40] Takahashi, Y. et al*.* An autophagy assay reveals the ESCRT-III component CHMP2A as a regulator of phagophore closure. *Nat. Commun*. **9**, 2855 (2018). 10.1038/s41467-018-05254-w PMC6054611 30030437

[41] Zhou, F. et al. Rab5-dependent autophagosome closure by ESCRT. *J. Cell Biol.* **218**, 1908–1927 (2019). 10.1083/jcb.201811173 PMC6548130 31010855

[42] Hattori, T. et al. Targeting the ESCRT-III component CHMP2A for noncanonical caspase-8 activation on autophagosomal membranes. *Cell Death Differ.* **28**, 657–670 (2021). 10.1038/s41418-020-00610-0 PMC7862398 32807832

[43] Stenmark, H. et al. Inhibition of Rab5 GTPase activity stimulates membrane fusion in endocytosis. *EMBO J.* **13**, 1287–1296 (1994). 10.1002/j.1460-2075.1994.tb06381.x PMC394944 8137813

[44] Guerra, F. & Bucci, C. Multiple roles of the small GTPase Rab7. *Cells* **5**, 34 (2016). 10.3390/cells5030034 PMC5040976 27548222

[45] Schneider-Brachert, W., Heigl, U. & Ehrenschwender, M. Membrane trafficking of death receptors: Implications on signalling. *Int. J. Mol. Sci.* **14**, 14475–14503 (2013). 10.3390/ijms140714475 PMC3742255 23852022

[46] Ford, M. C. J. et al. Simultaneous binding of PtdIns(4,5)P2 and clathrin by AP180 in the nucleation of clathrin lattices on membranes. *Science* **291**, 1051–1055 (2001). 10.1126/science.291.5506.1051 11161218

[47] Damke, H., Binns, D. D., Ueda, H., Schmid, S. L. & Baba, T. Dynamin GTPase domain mutants block endocytic vesicle formation at morphologically distinct stages. *Mol. Biol. Cell* **12**, 2578–2589 (2001). 10.1091/mbc.12.9.2578 PMC59696 11553700

[48] Uytterhoeven, V. et al. Hsc70-4 deforms membranes to promote synaptic protein turnover by endosomal microautophagy. *Neuron* **88**, 735–748 (2015). 10.1016/j.neuron.2015.10.012 26590345

[49] Tekirdag, K. & Cuervo, A. M. Chaperone-mediated autophagy and endosomal microautophagy: joint by a chaperone. *J. Biol. Chem.* **293**, 5414–5424 (2018). 10.1074/jbc.R117.818237 PMC5900761 29247007

[50] Wu, E. et al. HSPA8 acts as an amyloidase to suppress necroptosis by inhibiting and reversing functional amyloid formation. *Cell Res.* **33**, 851–866 (2023). 10.1038/s41422-023-00859-3 PMC10624691 37580406

[51] Everest, P., Roberts, M. & Dougan, G. Susceptibility to *Salmonella* Typhimurium infection and effectiveness of vaccination in mice deficient in the tumor necrosis factor alpha p55 receptor. *Infect. Immun.* **66**, 3355–3364 (1998). 10.1128/iai.66.7.3355-3364.1998 PMC108352 9632605

[52] Flynn, J. A. L. et al. Tumor necrosis factor-α is required in the protective immune response against *Mycobacterium tuberculosis* in mice. *Immunity* **2**, 561–572 (1995). 10.1016/1074-7613(95)90001-2 7540941

[53] Ali, T. et al. Clinical use of anti-TNF therapy and increased risk of infections. *Drug. Healthc. Patient Saf.* **5**, 79–99 (2013). 10.2147/DHPS.S28801 PMC3615849 23569399

[54] Doerflinger, M. et al. Flexible usage and interconnectivity of diverse cell death pathways protect against intracellular infection. *Immunity* **53**, 533–547 (2020). 10.1016/j.immuni.2020.07.004 PMC7500851 32735843

[55] Stutz, M. D. et al. Macrophage and neutrophil death programs differentially confer resistance to tuberculosis. *Immunity* **54**, 1758–1771 (2021). 10.1016/j.immuni.2021.06.009 34256013

[56] Dukes, J. D. et al. The secreted *Salmonella dublin* phosphoinositide phosphatase, SopB, localizes to PtdIns(3)P-containing endosomes and perturbs normal endosome to lysosome trafficking. *Biochem. J.* **395**, 239–247 (2006). 10.1042/BJ20051451 PMC1422764 16396630

[57] Mehra, A. et al. *Mycobacterium tuberculosis* type VII secreted effector EsxH targets host ESCRT to impair trafficking. *PLoS Pathog*. **9**, e1003734 (2013). 10.1371/journal.ppat.1003734 PMC3814348 24204276

[58] Williams, R. L. & Urbé, S. The emerging shape of the ESCRT machinery. *Nat. Rev. Mol. Cell Biol.* **8**, 355–368 (2007). 10.1038/nrm2162 17450176

[59] Claude-Taupin, A. et al. ATG9A protects the plasma membrane from programmed and incidental permeabilization. *Nat. Cell Biol.* **23**, 846–858 (2021). 10.1038/s41556-021-00706-w PMC8276549 34257406

[60] Füllsack, S., Rosenthal, A., Wajant, H. & Siegmund, D. Redundant and receptor-specific activities of TRADD, RIPK1 and FADD in death receptor signaling. *Cell Death Dis*. **10**, 122 (2019). 10.1038/s41419-019-1396-5 PMC6370826 30741924

[61] Liu, R. et al. Adenosine metabolic clearance maintains liver homeostasis by licensing arginine methylation of RIPK1. *J. Exp. Med*. **223**, e20250603 (2026). 10.1084/jem.20250603 PMC12517274 41081715

[62] Blackwell, K. et al. TRAF2 phosphorylation modulates tumor necrosis factor alpha-induced gene expression and cell resistance to apoptosis. *Mol. Cell. Biol.* **29**, 303–314 (2009). 10.1128/MCB.00699-08 PMC2612514 18981220

[63] Zhang, L., Blackwell, K., Altaeva, A., Shi, Z. & Habelhah, H. TRAF2 phosphorylation promotes NF-κB-dependent gene expression and inhibits oxidative stress-induced cell death. *Mol. Biol. Cell* **22**, 128–140 (2011). 10.1091/mbc.E10-06-0556 PMC3016971 21119000

[64] Vidal, M. Exosomes: revisiting their role as “garbage bags”. *Traffic* **20**, 815–828 (2019). 10.1111/tra.12687 31418976

[65] Sanwlani, R. & Gangoda, L. Role of extracellular vesicles in cell death and inflammation. *Cells* **10**, 1–19 (2021). 10.3390/cells10102663 PMC8534608 34685643

[66] Buzas, E. I. The roles of extracellular vesicles in the immune system. *Nat. Rev. Immunol.* **23**, 236–250 (2023). 10.1038/s41577-022-00763-8 PMC9361922 35927511

[67] Zhang, H.-G. et al. A membrane form of TNFα presented by exosomes delays T cell activation-induced cell death. * J. Immunol.* **177**, 2025–2025 (2006). 10.4049/jimmunol.176.12.7385 16751383

[68] Zhang, Q.-C. et al. TNF-α-stimulated nucleus pulposus cells induce cell apoptosis through the release of exosomal miR-16 targeting IGF-1 and IGF-1R in rats. *Ann. Transl. Med.* **9**, 1376–1376 (2021). 10.21037/atm-21-227 PMC8506555 34733928

[69] Nakao, Y. et al. Exosomes from TNF-α-treated human gingiva-derived MSCs enhance M2 macrophage polarization and inhibit periodontal bone loss. *Acta Biomater.* **122**, 306–324 (2021). 10.1016/j.actbio.2020.12.046 PMC7897289 33359765

[70] Yatim, N. et al. RIPK1 and NF-kB signaling in dying cells determines cross-priming of CD8^+^ T cells. *Science* **350**, 328–334 (2015). 10.1126/science.aad0395 PMC4651449 26405229

[71] Davidovich, P., Higgins, C. A., Najda, Z., Longley, D. B. & Martin, S. J. cFLIPL acts as a suppressor of TRAIL- and Fas-initiated inflammation by inhibiting assembly of caspase-8/FADD/RIPK1 NF-κB-activating complexes. *Cell Rep.* **42**, 113476 (2023). 10.1016/j.celrep.2023.113476 37988267

[72] De Almagro, M. C., Goncharov, T., Newton, K. & Vucic, D. Cellular IAP proteins and LUBAC differentially regulate necrosome-associated RIP1 ubiquitination. *Cell Death Dis.* **6**, 1–11 (2015). 10.1038/cddis.2015.158 PMC4669837 26111062

[73] De Almagro, M. C. et al. Coordinated ubiquitination and phosphorylation of RIP1 regulates necroptotic cell death. *Cell Death Differ.* **24**, 26–37 (2017). 10.1038/cdd.2016.78 PMC5260504 27518435

[74] Dice, F. Peptide sequences that target cytosolic proteins for lysosomal proteolysis. *Trends Biochem. Sci.* **15**, 305–309 (1990). 10.1016/0968-0004(90)90019-8 2204156

[75] Kinsella, R. L. & Stallings, C. L. A flexible and deadly way to control *Salmonella* infection. *Immunity* **53**, 471–473 (2020). 10.1016/j.immuni.2020.08.009 32937145

[76] Hu, G. Q. et al. Salmonella outer protein B suppresses colitis development via protecting cell from necroptosis. *Front. Cell. Infect. Microbiol.* **9**, 1–10 (2019). 10.3389/fcimb.2019.00087 PMC6465518 31024858

[77] Migliano, S. M., Wenzel, E. M. & Stenmark, H. Biophysical and molecular mechanisms of ESCRT functions, and their implications for disease. *Curr. Opin. Cell Biol.* **75**, 102062 (2022). 10.1016/j.ceb.2022.01.007 35248976

[78] Bend, R. et al. Phenotype and mutation expansion of the PTPN23 associated disorder characterized by neurodevelopmental delay and structural brain abnormalities. *Eur. J. Hum. Genet.* **28**, 76–87 (2020). 10.1038/s41431-019-0487-1 PMC6906308 31395947

[79] Sowada, N. et al. Mutations of PTPN23 in developmental and epileptic encephalopathy. *Hum. Genet.* **136**, 1455–1461 (2017). 10.1007/s00439-017-1850-3 29090338

[80] Khalaf-Nazzal, R. et al. Final exon frameshift biallelic ptpn23 variants are associated with microcephalic complex hereditary spastic paraplegia. *Brain Sci.* **11**, 1–9 (2021). 10.3390/brainsci11050614 PMC8151426 34064836

[81] Doench, J. G. et al. Optimized sgRNA design to maximize activity and minimize off- target effects of CRISPR-Cas9. *Nat. Biotechnol.* **34**, 184–191 (2016). 10.1038/nbt.3437 PMC4744125 26780180

[82] Huang, H. T. et al. MELK is not necessary for the proliferation of basal-like breast cancer cells. *Elife* **6**, 1–29 (2017). 10.7554/eLife.26693 PMC5605198 28926338

[83] Dondelinger, Y. et al. RIPK3 contributes to TNFR1-mediated RIPK1 kinase-dependent apoptosis in conditions of cIAP1/2 depletion or TAK1 kinase inhibition. *Cell Death Differ.* **20**, 1381–1392 (2013). 10.1038/cdd.2013.94 PMC3770330 23892367

[84] Priem, D. et al. ATG9A-mediated autophagy prevents inflammatory skin disease by limiting TNFR1-driven STING activation and ZBP1-dependent cell death. *Immunity* **10**, 2972–2988 (2025). 10.1016/j.immuni.2025.09.019 41118755

[85] Priem, D., Dondelinger, Y. & Bertrand, M. J. M. Monitoring RIPK1 phosphorylation in the TNFR1 signaling complex. in *Methods in Molecular Biology*, Vol. 1857 (Springer Nature, 2018). 10.1007/978-1-4939-8754-2_17 30136241

[86] Van Deun, J. et al. The impact of disparate isolation methods for extracellular vesicles on downstream RNA profiling. *J. Extracell. Vesicles* **3**, 1–14 (2014). 10.3402/jev.v3.24858 PMC4169610 25317274

[87] Vlantis K. et al. NEMO Prevents RIP Kinase 1-Mediated Epithelial Cell Death and Chronic Intestinal Inflammation by NF-κB-Dependent and -Independent Functions. *Immunity* **44**, 553–567 (2016). 10.1016/j.immuni.2016.02.020 PMC4803910 26982364

