# Supplementary material for: Exosomal export of activated CASPASE-8 is linked to the prevention of TNF-induced cytotoxicity
Source: Nat Commun. 2026 Aug 25;17:10178. doi: 10.1038/s41467-026-75544-1 (PMC13612411; doi:10.1038/s41467-026-75544-1)
Supplement: Supplementary file 1 [file 41467_2026_75544_MOESM1_ESM.pdf]

Exosomal Export of Activated CASPASE-8 is linked to the prevention of TNF-Induced Cytotoxicity

Jon Huyghe[1,2,*], Dario Priem[1,2], Annelore Haems[1,2], Lien Lippens[3,4], Margaux De Meyer[5,6], Tom Delanghe[1,2], Yves Dondelinger[1,2], Inge Bruggeman[1,2], Sven Eyckerman[5,6], An Hendrix[3,4], Mathieu JM Bertrand[1,2,*]

[1]VIB Center for Inflammation Research; 9052 Ghent, Belgium. [2]Department of Biomedical Molecular Biology, Ghent University; 9052 Ghent, Belgium. [3]Laboratory of Experimental Cancer Research, Department of Human Structure and Repair, Ghent University, Ghent, Belgium. [4]Cancer Research Institute Ghent, Ghent, Belgium. [5]VIB-UGent Center for Medical Biotechnology, Ghent, Belgium. [6]Department of Biomolecular Medicine, Ghent University, Ghent, Belgium.

*Corresponding authors: jon.huyghe@irc.vib-ugent.be, mathieu.bertrand@irc.vib-ugent.be

Supplementary Figure 1 | The ESCRT machinery prevents TNF-induced RIPK1 kinase-independent apoptosis independently of the known cell death checkpoints

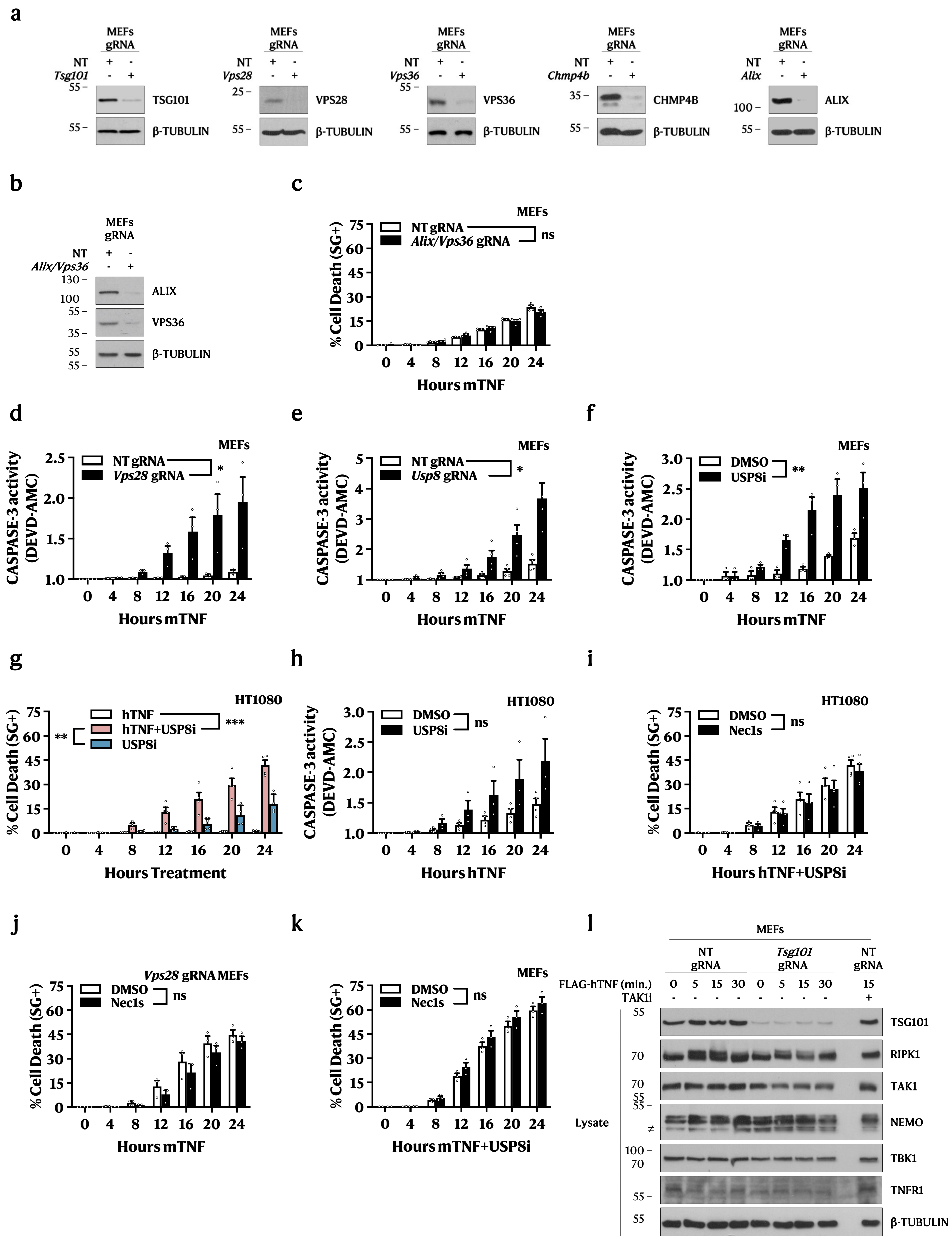

Supplementary Figure 1 continued | The ESCRT machinery prevents TNF-induced RIPK1 kinase-independent apoptosis independently of the known cell death checkpoints

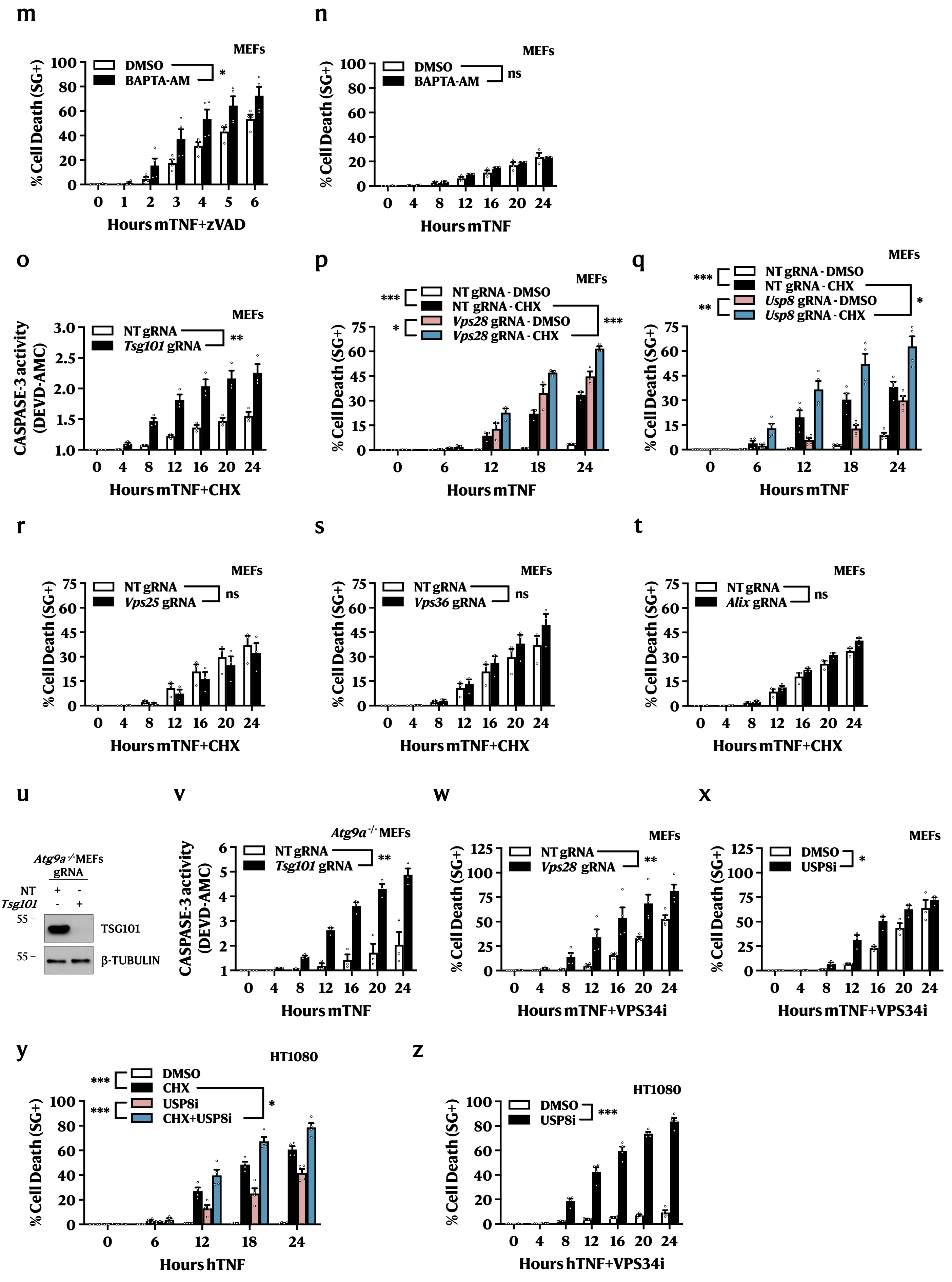

Supplementary Figure 1 | The ESCRT machinery prevents TNF-induced RIPK1 kinase-independent apoptosis independently of the known cell death checkpoints. **a-e,j,p-t,w**, MEFs were transduced with LentiCRISPRv2 constructs or electroporated with Cas9-RNPs targeting the indicated genes or a non-targeting sequence (NT). **a-b**, Knockout efficiency was validated by immunoblot. **c-e**, Cells were stimulated with 20 ng/mL mTNF. **c**, Treatment-induced cell death was measured by SytoxGreen (SG) positivity. **d-e**, Extracellular caspase activity was quantified using the fluorescent substrate DEVD-AMC. **f**, MEFs were pre-treated, or not, with USP8i before stimulation with 20 ng/mL mTNF. Extracellular caspase activity was quantified using DEVD-AMC. **g-i**, HT1080 cells were pre-treated USP8i and Nec1s as indicated prior to stimulation with 20 ng/mL hTNF as indicated. **g,i**, Treatment-induced cell death was measured by SytoxGreen (SG) positivity. **h**, Extracellular caspase activity was quantified using DEVD-AMC. **j**, Cells were pre-treated with Nec1s as indicated prior to stimulation with 20 ng/mL mTNF. Treatment-induced cell death was measured by SG positivity. **k**, MEFs were pre-treated with USP8i and Nec1s as indicated prior to stimulation with 20 ng/mL mTNF. Treatment-induced cell death was measured by SG positivity. **l,o**, MEFs inducibly expressing Cas9 were transduced with inducible expression vectors encoding sgRNAs targeting *Tsg101* or NT. Cells were treated with 1 µg/mL Doxycycline for 48h and subsequently expanded for 72h. **l**, Cells were pre-treated with TAK1i as indicated prior to stimulation with 1 µg/mL FLAG-hTNF. TNFR1 Complex I was isolated by FLAG immunoprecipitation, followed by USP21 treatment as indicated. Protein levels were determined by immunoblot (≠ denotes an aspecific band). **m-n**, MEFs were pre-treated with zVAD and the Ca^{2+}-chelator BAPTA-AM as indicated prior to stimulation with 20 ng/mL mTNF. Treatment-induced cell death was measured by SG positivity. **o-t**, Cells were pre-treated with CHX as indicated prior to stimulation with 20 ng/mL mTNF. **o**, Extracellular caspase activity was quantified using DEVD-AMC. **p-t**, Treatment-induced cell death was measured by SG positivity. **u-v**, *Atg9a* $^{-/-}$ MEFs obtained by isolation following Cas9-RNP electroporation were additionally electroporated with *Tsg101* -specific or NT Cas9-RNPs. **u**, Knockout efficiency was validated by immunoblot. **v**, Cells were stimulated with 0.1 ng/mL mTNF and extracellular caspase activity was quantified using DEVD-AMC. **w**, Cells were pre-treated with the VPS34 inhibitor SAR405 (VPS34i) prior to stimulation with 20 ng/mL mTNF. Treatment-induced cell death was measured over time by SG positivity. **x,z**, MEFs or HT1080 cells were pre-treated with VPS34i and USP8i as indicated prior to stimulation with 20 ng/mL mTNF or hTNF. Treatment-induced cell death was measured by SG positivity. **y**, HT1080 cells were pre-treated with CHX and USP8i as indicated prior to stimulation with 20 ng/mL hTNF. Treatment-induced cell death was measured by SG positivity. Immunoblots are representative of two independent experiments. Cell death and CASPASE-3 activity experiments are presented as mean ± SEM of three independent experiments and statistical significance was determined using two-way ANOVA. Significance between samples is indicated in the figure as follows: *p < 0.05; **p < 0.01; ***p < 0.001; ns non-significant.

Supplementary Figure 2 | Endosomal ILV formation prevents TNF-induced RIPK1 kinase-independent apoptosis

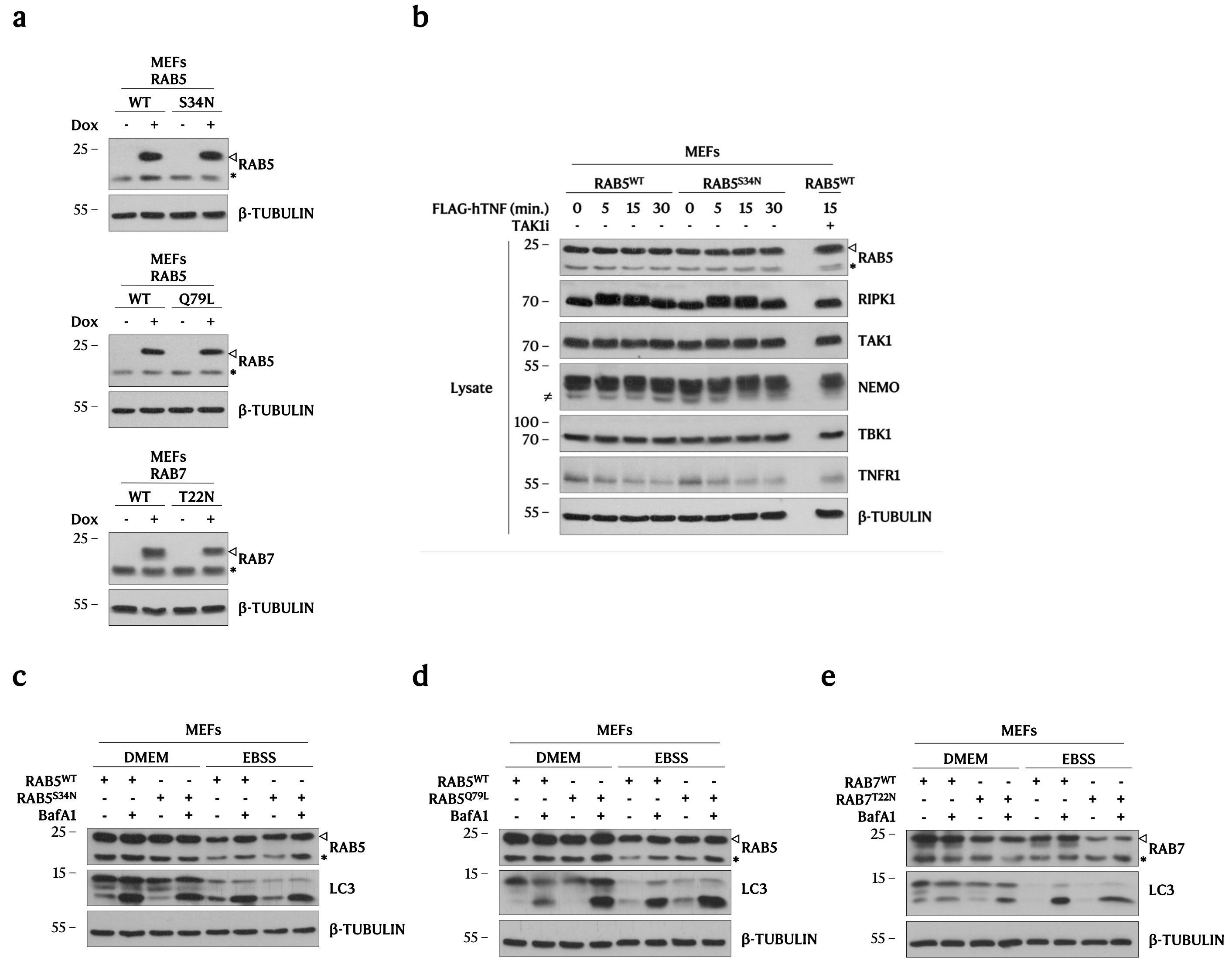

Supplementary Figure 2 | Endosomal ILV formation prevents TNF-induced RIPK1 kinase-independent apoptosis. **a-e**, MEFs inducibly expressing 3xHA-tagged wild-type RAB5 ($RAB5^{WT}$) or RAB7 ($RAB7^{WT}$), or the dominant-negative RAB5 Q79L or S34N or the dominant-negative RAB7 T22N ($RAB5^{Q79L}$, $RAB5^{S34N}$ and $RAB7^{T22N}$) were treated with 2 µg/mL Doxycycline (Dox) for 48h as indicated . **a**, Protein levels were determined by immunoblot. **b**, Cells were pre-treated with TAK1i as indicated prior to stimulation with 1 µg/mL FLAG-hTNF. TNFR1 Complex I was isolated by FLAG immunoprecipitation, followed by USP21 treatment as indicated. Protein levels were determined by immunoblot. **c-e**, Cells were treated with EBSS for 4h in the presence of BafA1 as indicated. Protein levels were determined by immunoblot (* denotes the endogenously expressed RAB5 or RAB7; ◁ indicates the ectopically expressed 3xHA-tagged RAB5 or RAB7; ≠ denotes an aspecific band). Immunoblots are representative of two independent experiments.

Supplementary Figure 3 | ILV formation protects against TNF cytotoxicity independently of TNFR1 trafficking

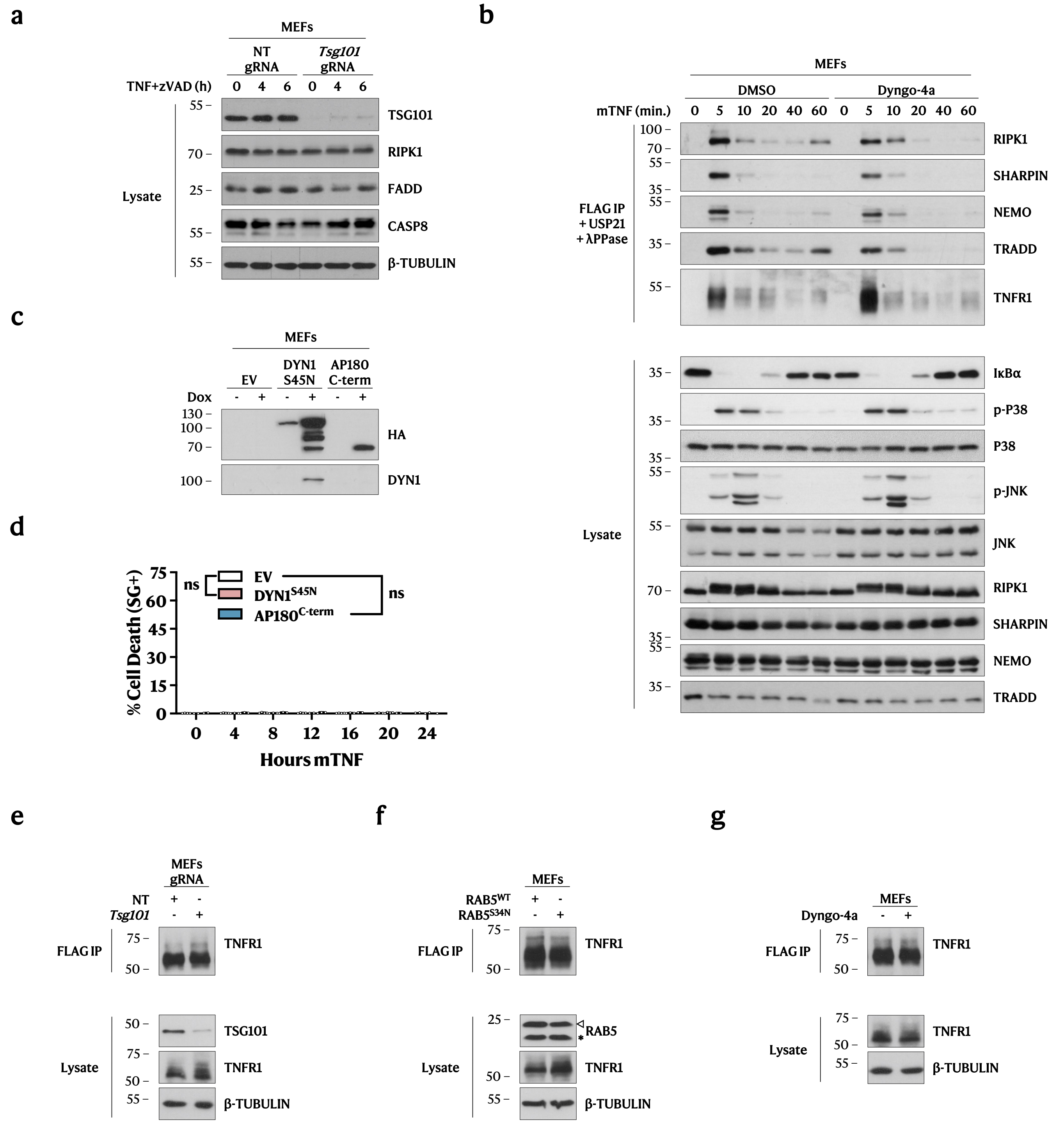

Supplementary Figure 3 | ILV formation protects against TNF cytotoxicity independently of TNFR1 trafficking. **a**,**e**, MEFs inducibly expressing Cas9 were transduced with inducible expression vectors encoding sgRNAs targeting *Tsg101* or a non-targeting sequence (NT). Cells were treated with 1 µg/mL Doxycycline (Dox) for 48h and subsequently expanded for 72h. **a**, Subsequently, cells were pre-treated with zVAD prior to stimulation with 20 ng/mL mTNF and TNFR1 Complex II was isolated by CASP8 immunoprecipitation. Protein levels were determined by immunoblot. **b**, Cells were pre-treated with Dyngo-4a as indicated and subsequently stimulated with 1 µg/mL FLAG-hTNF. TNFR1 Complex I was FLAG-immunoprecipitated, followed by USP21 and λPPase treatment. Protein levels were determined by immunoblot. **c**-**d**, MEFs inducibly expressing HA-tagged versions of the dominant negative DYNAMIN-1 S45N ($DYN1^{S45N}$), the dominant-negative C-terminal fragment of AP180 ($AP180^{C\text{-}term}$) or an empty vector (EV) were treated with 1 µg/mL Dox for 24h as indicated. **c**, Protein levels were determined by immunoblot. **d**, Cells were stimulated with 20 ng/mL mTNF. Treatment-induced cell death was measured by SytoxGreen (SG) positivity. **f**, MEFs inducibly expressing 3xHA-tagged wild-type RAB5 ($RAB5^{WT}$) or the dominant-negative RAB5 S34N ($RAB5^{S34N}$) were treated with 2 µg/mL Doxycycline (Dox) for 48h as indicated. **g**, MEFs were pre-treated with Dyngo-4a as indicated. **e**-**g**, Cells were subsequently incubated with ice-cold medium containing 1 µg/mL FLAG-hTNF for 2h. Surface-accessible TNFR1 was subsequently isolated by FLAG immunoprecipitation. Protein levels were determined by immunoblot (* denotes the endogenously expressed RAB5; ◁ indicates the ectopically expressed 3xHA-tagged RAB5). Cell death experiments are presented as mean ± SEM of three independent experiments and statistical significance was determined using a two-way ANOVA. Immunoblots are representative of two independent experiments.

Supplementary Figure 4 | Targeting of activated CASP8 into ILVs prevents TNF-induced cytotoxicity

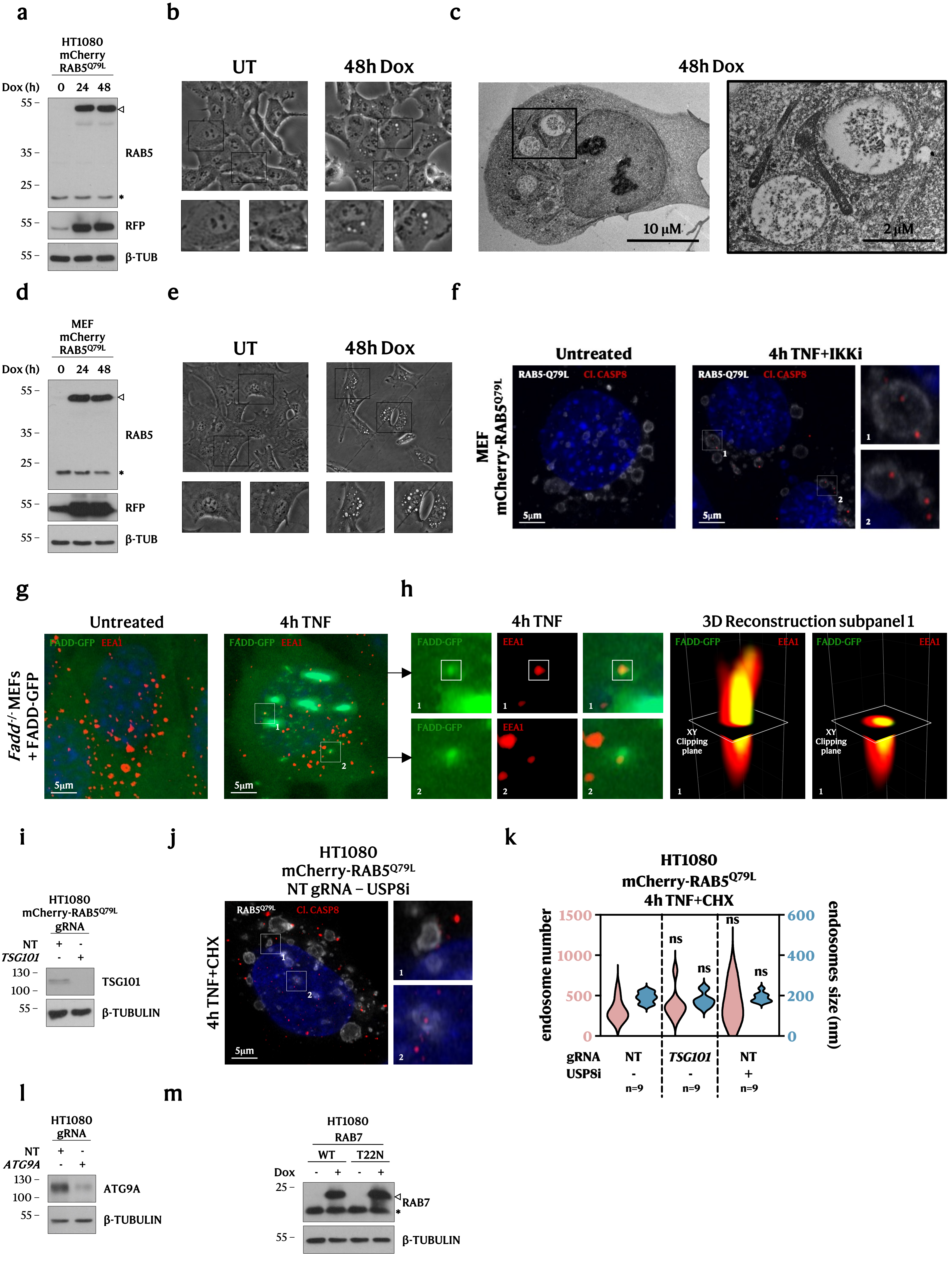

Supplementary Figure 4 continued | Targeting of activated CASP8 into ILVs prevents TNF-induced cytotoxicity

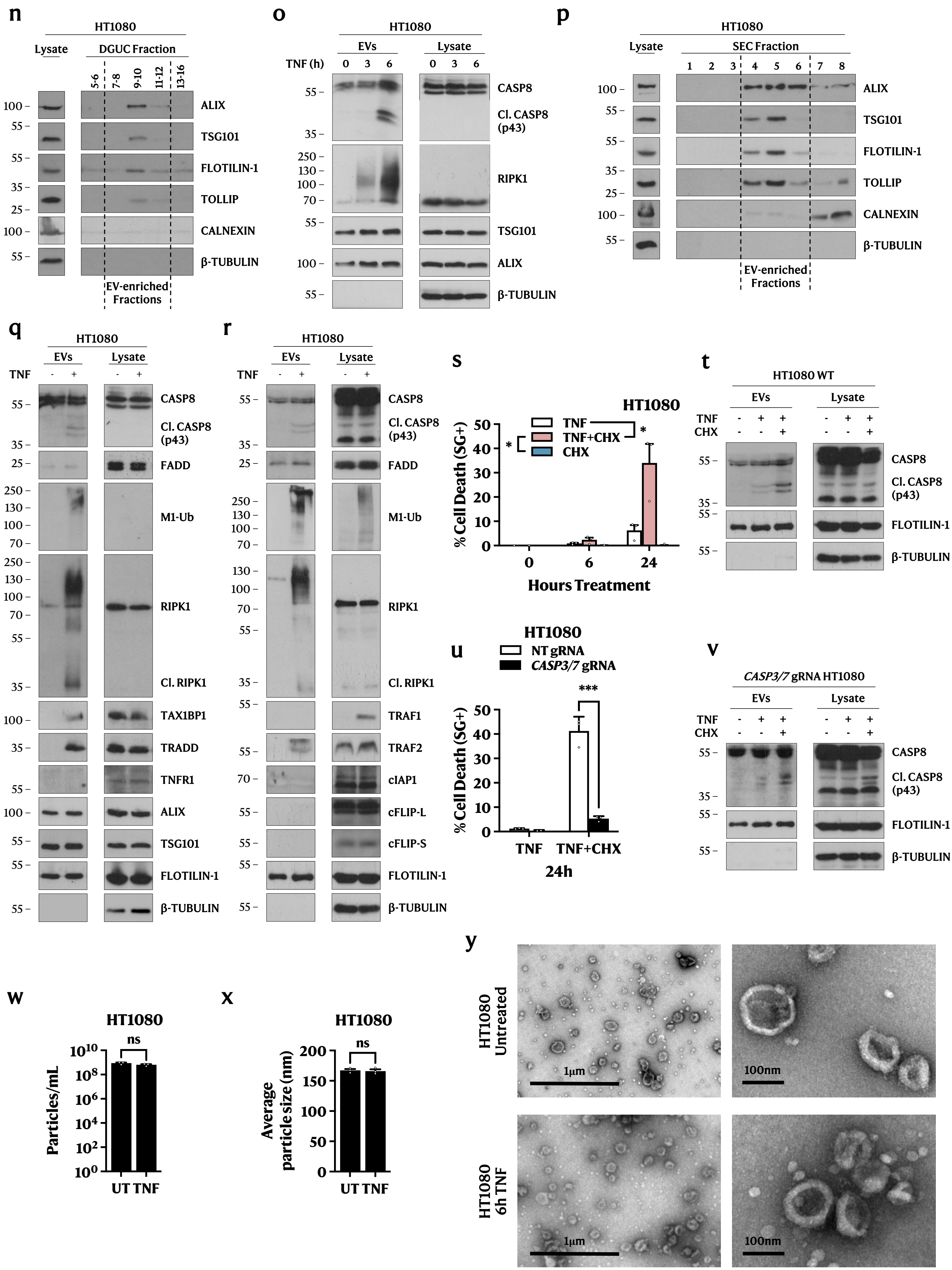

Supplementary Figure 4 | Targeting of activated CASP8 into ILVs prevents TNF-induced cytotoxicity. **a-c**, HT1080 cells inducibly expressing mCherry-tagged RAB5 Q79L (mCherry-RAB5^{Q79L}) were treated with 2 μg/mL Doxycycline (Dox) as indicated. **a**, Protein levels were determined by immunoblot (* denotes the endogenously expressed RAB5; ◁ indicates the ectopically expressed mCherry-RAB5^{Q79L}). **b**, Representative brightfield images of cells are shown. **c**, Representative transmission electron microscopy (TEM) images of cells are shown. **d-f**, MEFs inducibly expressing mCherry-RAB5^{Q79L} were treated with 2 μg/mL Dox as indicated. **d**, Protein levels were determined by immunoblot (* denotes the endogenously expressed RAB5; ◁ indicates the ectopically expressed mCherry-RAB5^{Q79L}). **e**, Representative brightfield images of cells are shown. **f**, Cells were pre-treated with IKKα/β inhibitor (IKKi) prior to stimulation with 1 μg/mL mTNF as indicated. Cells were then stained for Cl. CASP8. Representative maximum intensity projections of Cl. CASP8 immunostainings are shown. **g-h**, *Fadd* $^{-/-}$ MEFs obtained following Cas9-RNP electroporation were reconstituted with an inducible expression vector encoding GFP-tagged FADD. Cells were treated with 100 ng/mL Doxycycline for 24h. Subsequently, cells were stimulated or with 20 ng/mL mTNF as indicated. Cells were then stained for the early endosome marker EEA1. **g**, Representative maximum intensity projections of EEA1 immunostainings are shown. **h**, Single and merged channel images of subpanels of cells treated with TNF are shown, including a 3D reconstruction of subpanel 1. The reconstructed image was clipped along an XY-plane to allow visualization of the contents of EEA1^{+} endosomes. **i-k**, HT1080 inducibly expressing mCherry-RAB5^{Q79L} were electroporated with Cas9-RNPs targeting *TSG101* or a non-targeting sequence (NT). **i**, Knockout efficiency was validated by immunoblot. **j-k**, Cells were treated with 2 μg/mL Dox for 48h. Subsequently, cells were pre-treated with CHX and USP8i as indicated prior to stimulation with 1 μg/mL hTNF. Cells were then stained for Cl. CASP8. **j**, Representative maximum intensity projections of Cl. CASP8 immunostainings are shown. **k**, The average number and size of mCherry-RAB5^{Q79L+} endosomes were quantified. Violin plots represent the distribution across images. **l**, HT1080 cells were electroporated with Cas9-RNPs targeting *ATG9A* or NT. Knockout efficiency was validated by immunoblot. **m**, HT1080 cells inducibly expressing 3xHA-tagged wild-type RAB7 (RAB7^{WT}), or the dominant-negative RAB7 T22N mutants (RAB7^{T22N}) were treated with 2 μg/mL Doxycycline for 48h as indicated. Protein levels were determined by immunoblot (* denotes the endogenously expressed RAB7; ◁ indicates the ectopically expressed HA-tagged RAB7). **n**, Conditioned medium of untreated HT1080 cells was harvested after 6h and separated by density gradient ultracentrifugation (DGUC). Protein levels were determined by immunoblot. **o**, HT1080 cells were stimulated with 2 μg/mL hTNF as indicated. Cells were lysed and extracellular vesicles (EVs) were pelleted by ultracentrifugation. Protein levels were determined by immunoblot. **p**, Conditioned medium of untreated HT1080 cells was harvested after 6h and separated by size exclusion chromatography (SEC). Protein levels in each fraction were determined by immunoblot. **q-r**, HT1080 cells were stimulated with 2 μg/mL hTNF for 6h as indicated. Cells were lysed and extracellular vesicles (EVs) were isolated by SEC (Fractions 4-6). Protein levels were determined by immunoblot. **s**, HT1080 cells were pre-treated with CHX as indicated prior to stimulation with 2 μg/mL hTNF. Treatment-induced cell death was measured by SytoxGreen (SG) positivity. **t**, Cells were pre-treated with CHX as indicated prior to stimulation with 2 μg/mL hTNF for 6h. Cells were lysed and extracellular vesicles (EVs) were isolated by size exclusion chromatography (SEC) (Fractions 4-6). Protein levels were determined by immunoblot. **u-v**, HT1080 cells were electroporated with Cas9-RNPs targeting *CASP3* and *CASP7* or NT. **u**, Cells were pre-treated with CHX as indicated prior to stimulation with 2 μg/mL hTNF. Treatment-induced cell death was measured by SytoxGreen (SG) positivity. **v**, Cells were pre-treated with CHX as indicated prior to stimulation with 2 μg/mL hTNF for 6h. Cells were lysed and extracellular vesicles (EVs) were isolated by size exclusion chromatography (SEC) (Fractions 4-6). Protein levels were determined by immunoblot. **w-y**, HT1080 cells were stimulated with 2 μg/mL hTNF for 6h as indicated. Conditioned medium was harvested and separated using DGUC. **w-x**, The original particle concentration and average particle size in the conditioned medium were determined by Nanoparticle Tracking Analysis (NTA). **y**, Representative TEM images of the isolated EVs are shown. Cell death experiments are presented as mean ± SEM of three independent experiments and statistical significance was determined using a two-way ANOVA or multiple unpaired t-tests. Immunoblots are representative of two independent experiments. NTA data are presented as mean ± SEM of three independent experiments and statistical significance was determined using unpaired two-sided t-tests. Significance between samples is indicated in the figure as follows: *$p < 0.05$; **$p < 0.01$; ***$p < 0.001$; ns non-significant.

Supplementary Figure 5 | Linear ubiquitination of RIPK1 promotes the TAX1BP1- and HSC70-dependent targeting of activated CASP8 into ILVs

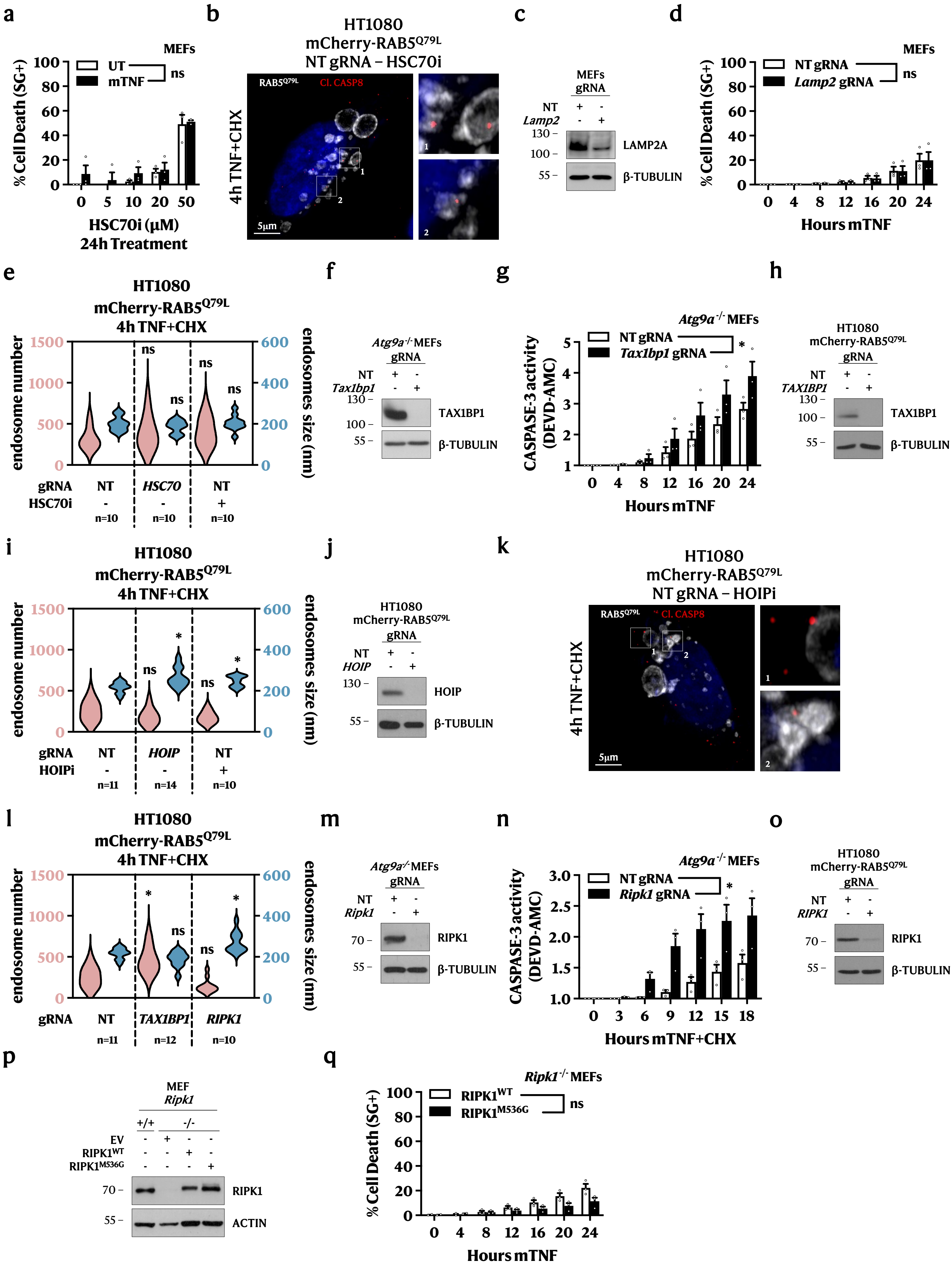

Supplementary Figure 5 | Linear ubiquitination of RIPK1 promotes the TAX1BP1- and HSC70-dependent targeting of activated CASP8 into ILVs. **a**, MEFs were pre-treated with different concentrations of HSC70 ATPase inhibitor (HSC70i) prior to stimulation with 20 ng/mL mTNF. Treatment-induced cell death was measured by SytoxGreen (SG) positivity. **b**, HT1080 cells inducibly expressing mCherry-tagged RAB5 Q79L (mCherry-RAB5^{Q79L}) were electroporated with Cas9-RNPs targeting a non-targeting sequence (NT). Cells were treated with 2 μg/mL Doxycycline for 48h. Subsequently, cells were pre-treated with CHX and 50 μM HSC70i before stimulation with 1 μg/mL hTNF. Cells were then stained for Cl. CASP8. Representative maximum intensity projections of Cl. CASP8 immunostainings are shown. **c-d**, MEFs were electroporated with Cas9-RNPs targeting *Lamp2* or NT. **c**, Knockout efficiency was validated by immunoblot. **d**, Cells were stimulated with 20 ng/mL mTNF and treatment-induced cell death was measured by SG positivity. **e,i,l**, HT1080 cells inducibly expressing mCherry-RAB5^{Q79L} were electroporated with Cas9-RNPs targeting the indicated genes or NT. Cells were treated with 2 μg/mL Doxycycline for 48h. Subsequently, cells were pre-treated with CHX and 50 μM HSC70i prior to stimulation with 1 μg/mL hTNF. Cells were then stained for Cl. CASP8. The average number and size of mCherry-RAB5^{Q79L+} endosomes were quantified. Violin plots represent the distribution across images. **f-g,m-n**, *Atg9a* $^{-/-}$ MEFs obtained by clonal isolation following Cas9-RNP electroporation were additionally electroporated with Cas9-RNPs targeting the indicated genes or NT. **f,m**, Knockout efficiency was validated by immunoblot. **g,n**, Extracellular caspase activity was quantified using the fluorescent substrate DEVD-AMC. **h,j-k,o**, HT1080 cells inducibly expressing mCherry-RAB5^{Q79L} were electroporated with Cas9-RNPs targeting the indicated genes or NT. **h,j,o**, Knockout efficiency was validated by immunoblot. **k**, Cells were treated with 2 μg/mL Doxycycline for 48h. Subsequently, cells were pre-treated with CHX and 30 μM HOIPi prior to stimulation with 1 μg/mL hTNF. Cells were then stained for Cl. CASP8. Representative maximum intensity projections of Cl. CASP8 immunostainings are shown. **p,q**, *Ripk1* $^{-/-}$ MEFs inducibly expressing either wild-type RIPK1 (RIPK1^{WT}), M536G RIPK1 (RIPK1^{M536G}) or an empty vector (EV) were treated with 100 ng/mL Doxycycline for 24h. **p**, Reconstitution was validated and compared to *Ripk1* $^{+/+}$ MEFs via immunoblotting. **q**, Cells were treated with 20 ng/mL mTNF and treatment-induced cell death was measured by SG positivity. Cell death and CASPASE-3 activity experiments are presented as mean ± SEM of three independent experiments and statistical significance was determined using two-way ANOVA. Significance between samples is indicated in the figure as follows: *$p < 0.05$; **$p < 0.01$; ***$p < 0.001$; ns non-significant.

Supplementary Figure 6 | Microbial effector proteins shift the TNF response from life to death by interfering with the ILV-dependent detoxification of activated CASP8

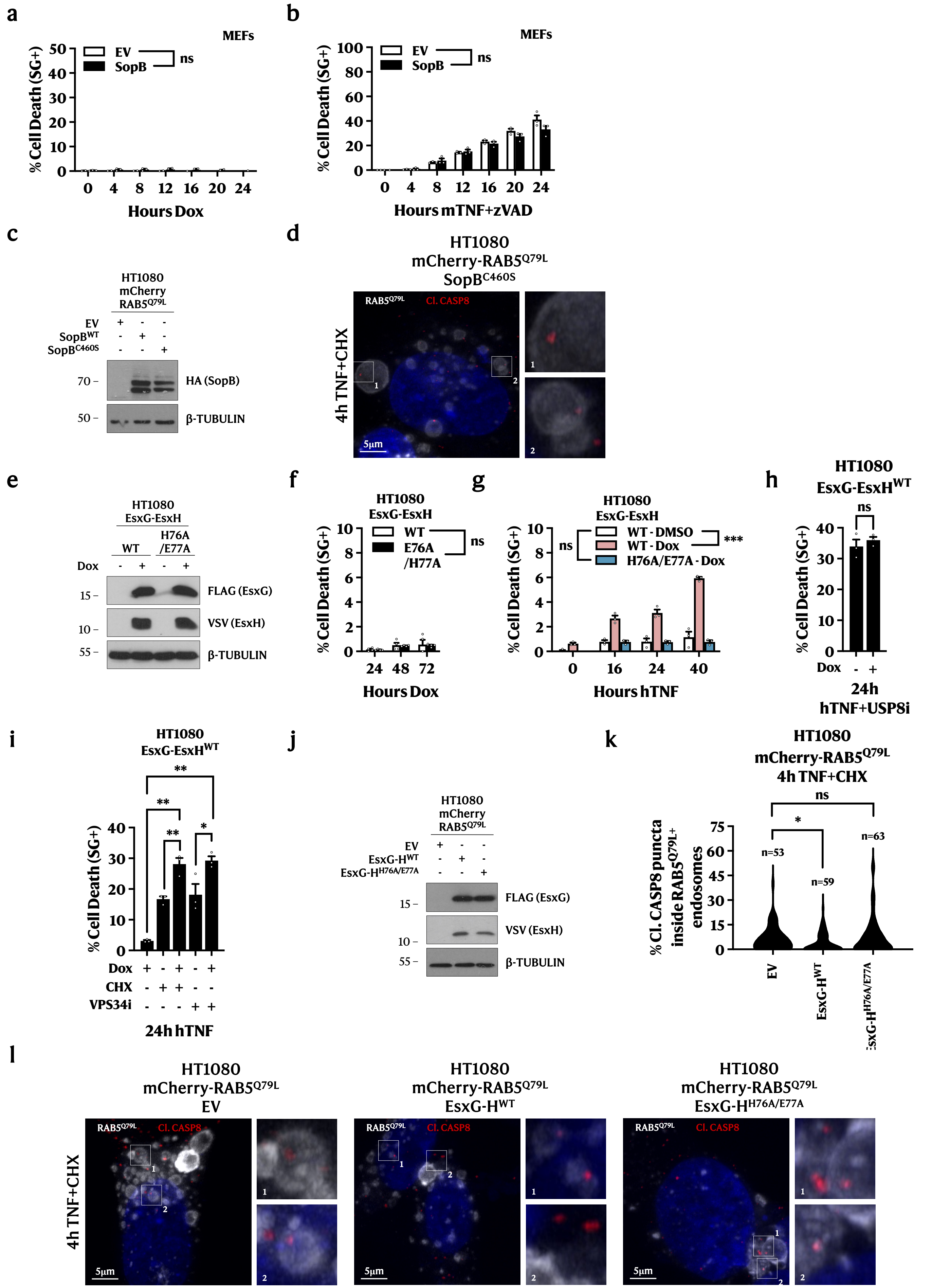

Supplementary Figure 6 | Microbial effector proteins shift the TNF response from life to death by interfering with the ILV-dependent detoxification of activated CASP8. **a-b**, MEFs inducibly expressing an empty vector (EV) or the *Salmonella* Typhimurium effector protein SopB were treated with 2 μg/mL Doxycycline (Dox) for 48h. **a**, Subsequently, treatment-induced cell death was measured in the presence of Dox by SytoxGreen (SG) positivity. **b**, Cells were then were pre-treated with zVAD prior to stimulation with 20 ng/mL mTNF. Treatment-induced cell death was measured by SG positivity. **c-d**, HT1080 cells inducibly expressing mCherry-tagged RAB5 Q79L (mCherry-RAB5^{Q79L}) were additionally transduced with inducible expression vectors encoding the *Salmonella* Typhimurium effector protein SopB wild-type (SopB^{WT}) or C460S (SopB^{C460S}). Cells were treated with 2 μg/mL Dox for 48h. **c**, Protein levels were determined by immunoblot. **d**, Subsequently, cells were pre-treated with CHX prior to stimulation with 1 μg/mL hTNF. Cells were then stained for Cl. CASP8. Representative maximum intensity projections of Cl. CASP8 immunostainings are shown. **e-i**, HT1080 inducibly expressing the *Mycobacterium tuberculosis* (*Mtb*) effector proteins EsxG-H WT (EsxG-H^{WT}) or H76A-E77A (EsxG-H$^{H76A\text{-}E77A}$) were treated with 1 μg/mL Dox for 24h as indicated. **e**, Protein levels were determined by immunoblot. **f-i**, Cells were then pre-treated with CHX, VPS34i or USP8i as indicated prior to stimulation with 20 ng/mL hTNF. Treatment-induced cell death was measured by SG positivity. **j-l**, HT1080 cells inducibly expressing mCherry-RAB5^{Q79L} were additionally transduced with an empty vector (EV) or inducible expression vectors encoding the *Mtb* effector proteins EsxG-H^{WT} or EsxG-H$^{H76A\text{-}E77A}$. Cells were then treated with 2 μg/mL Dox for 48h. **j**, Protein levels were determined by immunoblot. **k-l**, Subsequently, cells were pre-treated with CHX prior to stimulation with 1 μg/mL hTNF. Cells were then stained for Cl. CASP8. **k**, The percentage of Cl. CASP8 found inside mCherry-RAB5^{Q79L+} endosomes was quantified. Violin plots represent the distribution over individual imaged cells. **l**, Representative maximum intensity projections of Cl. CASP8 immunostainings are shown. Cell death experiments are presented as mean ± SEM of three independent experiments and statistical significance was determined using either two-way ANOVA followed by a Dunnett's multiple comparisons test, one-way ANOVA followed by a Šídák multiple comparisons test or unpaired two-sided t-test. Statistical significance of the microscopy data was determined using a Kruskal-Wallis test followed by a Dunn's multiple comparisons test. Significance between samples is indicated in the figure as follows: *$p < 0.05$; **$p < 0.01$; ***$p < 0.001$; ns non-significant.
