# Supplementary material for: Exosomal export of activated CASPASE-8 is linked to the prevention of TNF-induced cytotoxicity
Source: Nat Commun. 2026 Aug 25;17:10178. doi: 10.1038/s41467-026-75544-1 (PMC13612411; doi:10.1038/s41467-026-75544-1)
Supplement: Supplementary file 2 [file 41467_2026_75544_MOESM2_ESM.pdf]

nature portfolio

Corresponding author(s): Jon Huyghe
Mathieu JM Bertrand

Last updated by author(s): Jun 8, 2026

Reporting Summary

Nature Portfolio wishes to improve the reproducibility of the work that we publish. This form provides structure for consistency and transparency in reporting. For further information on Nature Portfolio policies, see our Editorial Policies and the Editorial Policy Checklist.

Statistics

For all statistical analyses, confirm that the following items are present in the figure legend, table legend, main text, or Methods section.

n/a	Confirmed	
☐	☒	The exact sample size (n) for each experimental group/condition, given as a discrete number and unit of measurement
☐	☒	A statement on whether measurements were taken from distinct samples or whether the same sample was measured repeatedly
☐	☒	The statistical test(s) used AND whether they are one- or two-sided<br>*Only common tests should be described solely by name; describe more complex techniques in the Methods section.*
☐	☒	A description of all covariates tested
☐	☒	A description of any assumptions or corrections, such as tests of normality and adjustment for multiple comparisons
☐	☒	A full description of the statistical parameters including central tendency (e.g. means) or other basic estimates (e.g. regression coefficient) AND variation (e.g. standard deviation) or associated estimates of uncertainty (e.g. confidence intervals)
☐	☒	For null hypothesis testing, the test statistic (e.g. F, t, r) with confidence intervals, effect sizes, degrees of freedom and P value noted<br>*Give P values as exact values whenever suitable.*
☒	☐	For Bayesian analysis, information on the choice of priors and Markov chain Monte Carlo settings
☒	☐	For hierarchical and complex designs, identification of the appropriate level for tests and full reporting of outcomes
☒	☐	Estimates of effect sizes (e.g. Cohen's d, Pearson's r), indicating how they were calculated

Our web collection on statistics for biologists contains articles on many of the points above.

Software and code

Policy information about availability of computer code

Data collection	All confocal microscopy signals were captured by the AiryScan detector. Images acquired in FAST mode were processed using ZEN software (Zeiss, Jena). The processing included pixel reassignment and default Wiener filtering. AiryScan processed images were finally subjected to Extended Depth of Focus (EDF) projection using the EDF module (maximum projection) on ZEN Blue software. Puncta were detected using the ArivisPro 4.2.2. software (Zeiss). 3D reconstruction images of Z-stacks were made using the ArivisPro 4.2.2. software (Zeiss).
Data analysis	GraphPad Prism 10 was used to generate figures and to perform statistical analysis.

For manuscripts utilizing custom algorithms or software that are central to the research but not yet described in published literature, software must be made available to editors and reviewers. We strongly encourage code deposition in a community repository (e.g. GitHub). See the Nature Portfolio guidelines for submitting code & software for further information.

Data

Policy information about availability of data

All manuscripts must include a data availability statement. This statement should provide the following information, where applicable:

- Accession codes, unique identifiers, or web links for publicly available datasets
- A description of any restrictions on data availability
- For clinical datasets or third party data, please ensure that the statement adheres to our policy

Requests for unprocessed microscopy data, resources, reagents, plasmids and cell lines used in this study and additional information should be directed to the lead contact Mathieu JM Bertrand (mathieu.bertrand@irc.vib-ugent.be)

Research involving human participants, their data, or biological material

Policy information about studies with human participants or human data. See also policy information about sex, gender (identity/presentation), and sexual orientation and race, ethnicity and racism.

Reporting on sex and gender	Not applicable
Reporting on race, ethnicity, or other socially relevant groupings	Not applicable
Population characteristics	Not applicable
Recruitment	Not applicable
Ethics oversight	Not applicable

Note that full information on the approval of the study protocol must also be provided in the manuscript.

Field-specific reporting

Please select the one below that is the best fit for your research. If you are not sure, read the appropriate sections before making your selection.

☒ Life sciences ☐ Behavioural & social sciences ☐ Ecological, evolutionary & environmental sciences

For a reference copy of the document with all sections, see nature.com/documents/nr-reporting-summary-flat.pdf

Life sciences study design

All studies must disclose on these points even when the disclosure is negative.

Sample size	Predetermination of samples sizes was not performed prior to the experiments presented in this study.
Data exclusions	No generated data was excluded from this study.
Replication	Quantitative measurements are presented as mean ± SEM of at least three independent biological replicates. Qualitative experiments are represented as representative images of at least two independent biological replicates.
Randomization	Not applicable
Blinding	Not applicable

Reporting for specific materials, systems and methods

We require information from authors about some types of materials, experimental systems and methods used in many studies. Here, indicate whether each material, system or method listed is relevant to your study. If you are not sure if a list item applies to your research, read the appropriate section before selecting a response.

Materials & experimental systems

n/a	Involved in the study
☐	☒ Antibodies
☐	☒ Eukaryotic cell lines
☒	☐ Palaeontology and archaeology
☒	☐ Animals and other organisms
☒	☐ Clinical data
☒	☐ Dual use research of concern
☒	☐ Plants

Methods

n/a	Involved in the study
☒	☐ ChIP-seq
☒	☐ Flow cytometry
☒	☐ MRI-based neuroimaging

Antibodies

Antibodies used

A20 Santa Cruz Biotechnology sc-166692 1/1000
ACTIN-HRP Proteintech HRP-66009 1/10.000
ALIX Santa Cruz Biotechnology sc-271975 1/200
ATG9A Abcam Ab108338 1/1000
CALNEXIN Abcam ab10286 1/1000
CASPASE 3 Cell Signaling Technology #9662 1/1000
CASPASE 8 Abnova MAB3429 1/1000
CASPASE 8 Prof. P. Krammer 1/1000
cFLIP Enzo Life Sciences ALX-804-127 1/1000
CHMP4B Abcam ab105767 1/1000
cIAP1 Enzo Life Sciences ALX-803-335 1/1000
Cleaved CASPASE 8 Abcam ab75852 1/1000
Cleaved PARP Cell Signaling Technology #9544 1/1000
FLAG Sigma Aldrich A8592 1/7500
FLOTILIN-1 Abcam ab133497 1/10.000
HA Covance MMS-101R 1/1000
HOIP Abcam ab46322 1/1000
HSC70 Santa Cruz Biotechnology sc-24 1/1000
IκBα Cell Signaling Technology #9242 1/1000
phospho-IκBα Cell Signaling Technology #9246 1/1000
JNK Cell Signaling Technology #9252 1/1000
phospho-JNK Invitrogen 44682G 1/1000
LAMP2A Abcam ab18528 1/1000
LC3 Sigma Aldrich L7543 1/1000
M1-UBIQUITIN Life Sensors AB130 1/2000
MLKL Merck Millipore # MABC604 1/1000
pS345 MLKL Cell Signaling Technology #37333 1/1000
NEMO Pasparakis lab Vlantis et al. 2016 1/1000
P38 Cell Signaling Technology #9212 1/1000
phospho-P38 Cell Signaling Technology #9211 1/1000
RAB5 Abcam ab18211 1/1000
RAB7 Cell Signaling Technology #9367 1/2000
RFP MBL PM005 1/1000
RIPK1 Cell Signaling Technology #3493 1/2000
RIPK1 Santa Cruz Biotechnology sc-7881 1/1000
phoshpo-S166/T169 RIPK1 ThermoFisher Custom made 1/1000
RIPK3 Cell Signaling Technology #10188 1/1000
pT231/S232 RIPK3 Cell Signaling Technology #57220 1/1000
SHARPIN Proteintech 14626-1-AP 1/1000
TAK1 Santa Cruz Biotechnology sc-7162 1/1000
TAX1BP1 Abcam ab176572 1/1000
TBK1 Cell Signaling Technology #3013 1/1000
TNFR1 Cell Signaling Technology #13377 1/1000
TNFR1 Santa Cruz Biotechnology sc-8436 1/1000
TOLLIP Abcam ab187198 1/1000
TRADD Cell Signaling Technology #3694 1/1000
TRAF1 Santa Cruz Biotechnology Sc-7186 1/1000
TRAF2 Cell Signaling Technology #4724 1/1000
TSG101 Santa Cruz Biotechnology sc-7964 1/1000
V5-HRP Invitrogen R961-25 1/5000
VPS28 Santa Cruz Biotechnology sc-166537 1/100
VPS36 Novus Bio NBP2-13519 1/1000
VSV Sigma Aldrich A 5977 1/3000
β-TUBULIN-HRP Abcam ab21058 1/10000

Validation

Antibodies were used according to the instructions and validation of the manufacturer's website.

Eukaryotic cell lines

Policy information about cell lines and Sex and Gender in Research

Cell line source(s)	Mouse embryonic fibroblasts (MEFs), HT1080 (ATCC CCL-121) and HEK293T (ATCC CRL-3216) cells were cultured in Dulbecco's modified Eagle's medium supplemented with 10% fetal calf serum, L-glutamine (200 mM) and sodium pyruvate (400 mM) in normoxic conditions (5% CO2). Atg9a-/-, Ripk1-/- and Tnfr1-/- MEFs were described earlier (Huyghe et al. Science 2022, Dondelinger et al. Cell Death Differ. 2013, Priem et al. Immunity 2025) . CRISPR-mediated knockout cell lines were obtained either via transduction with LentiCRISPRv2-generated lentiviral vectors or via electroporation of Cas9-RNPs.
Authentication	All cell lines were generated in house or obtained from a trusted source. Cell lines were kept at low passages in order to maintain their identity and all cell lines were authenticated by morphology check and growth curve analysis.
Mycoplasma contamination	All cell lines used in this study routinely tested negative for potential Mycoplasma contamination.
Commonly misidentified lines (See ICLAC register)	No commonly misidentified cell lines were used in this study.

Plants

Seed stocks	Not applicable
Novel plant genotypes	Not applicable
Authentication	Not applicable
