# Supplementary material for: Exosomal export of activated CASPASE-8 is linked to the prevention of TNF-induced cytotoxicity
Source: Nat Commun. 2026 Aug 25;17:10178. doi: 10.1038/s41467-026-75544-1 (PMC13612411; doi:10.1038/s41467-026-75544-1)
Supplement: Supplementary file 3 [file 41467_2026_75544_MOESM3_ESM.pdf]

nature portfolio

Peer Review File

Exosomal Export of Activated CASPASE-8 is Linked to the Prevention of TNF-Induced Cytotoxicity

Corresponding Author: Professor Mathieu Bertrand

This file contains all reviewer reports in order by version, followed by all author rebuttals in order by version.

Attachments originally included by the reviewers as part of their assessment can be found at the end of this file.

Version 0:

Reviewer comments:

Reviewer #1

(Remarks to the Author)
Bertrand and colleagues report a previously unidentified survival checkpoint of TNF complex II regulated by endosomal micro-autophagy and dependent on the ESCRT machinery and HSC70. Furthermore, this mechanism is defined as parallel and non-redundant to the LC3-independent macro-autophagy mechanism of complex II degradation. The findings reported here highlight the importance of "detoxification" mechanisms in the regulation of TNF-induced cell death and report a physiologically relevant model where this mechanism is inhibited in the context of infection, sensitizing to TNF cytotoxicity.

These findings align with established literature in some respects, while simultaneously challenging recent reports making this work significant to the field, with potential to refine the current knowledge in regards to TNF-induced cell death regulation.

With the purpose of supporting the conclusions made in this report there is need of some additional information.

Figure 1:

1. In order to clarify the cytoprotective function of USP8 in the context of TNF-induced complex II formation the authors would need to test the previously described (Jeong et al., Oncogene. 2017 Jan 26;36(4):458-470. doi: 10.1038/onc.2016.215) USP8 mediated regulation of cFLIP-L via its deubiquitylation, thereby protecting it from proteasomal degradation. Furthermore, it would be important to speculate on why the chemical inhibition of USP8 with DC-U4106 proves to be a stronger sensitizer compared to its genetic removal (Fig 1a and 1b).

2. The protective role of ESCRT components on TNF-induced necroptosis should be further explored by treatment of the ESCRT-deficient KO lines with TNF together with zVAD. This would be important since the same methodology is used for inhibition of ESCRT-machinery activation with Ca2+ chelator on Extended Data Figure 1k-l.

3. The addition of cycloheximide was tested for MEFs lacking different components of the ESCRT machinery and for HT1080 under USP8 inhibition (Fig 1h; Extended Data Fig. 1m-o,w). Here, the authors show bar plots with the direct comparison of ESCRT-proficient and -deficient cells under the challenge of TNF+CHX; however, there is lack of the direct comparison with TNF treatment alone as a control. Only with this comparison it is possible to understand if the pro-survival effects are dependent or not on gene activation. By comparing Fig 1e DMSO and 1h on Tsg101 KO MEFs it appears that the sensitization to TNF-induced cell death is lost; it would be important to speculate on why this is the case as this would further strengthen the claim of a new checkpoint on complex II.

Figure 3:

4. In Figure 3 the authors aim to demonstrate the presence of TNF complex II in intralumenal vesicles. The use of immunofluorescence for assessment of localization of cleaved caspase 8 should be complemented with the detection of other components of complex II, furthermore, immunostaining of full-length caspase 8 would be essential to complement Fig3 f-g, where full length caspase 8 is found in extracellular vesicles, both with and without TNF stimulation. Similarly,

Caspase 8 immunoprecipitation in Figure 3e should include markers of intralumenal vesicles such as Rab5 to corroborate the findings from the immunofluorescence panels.

5. Later, the authors explore the fate of the vesicles originated by ESCRT-dependent endosomal micro-autophagy. As mentioned in the discussion “lysosomal degradation is a possible convergence point of both macro-autophagy and eMI”, as such, the localization of complex II onto the lysosomal compartment should be evaluated by immunofluorescence by co-localization of cleaved caspase 8 and known lysosomal markers such as LAMP1/LAMP2; this should be performed under Atg9a-proficient and -deficient background. Another possible approach could be the immunoprecipitation of LAMP1 as used in reference 12 of the present manuscript, previous work by the same group.

Figure 4:

6. In Figure 4, a model is proposed where M1-Ub RIPK1 is proposed as a scaffold for TAXBP1 promoting its transfer to endosomes. However, in cells that were not challenged with TNF, caspase 8 was found in extracellular vesicles (Figure 3f-g) without the presence of RIPK1, TAXBP1 or M1-ubiquitin. It would be important to understand how this is regulated and if Caspase 8 could be the initial substrate of eMI cargo selection.

7. On the contrary, in previous work not mentioned in this manuscript (Wu et al., Cell Res. 2023 Nov;33(11):851-866. doi: 10.1038/s41422-023-00859-3), and supporting a role for RIPK1 as a potential scaffold for eMI initiation, HSC70 has been reported to suppress necroptosis by specifically recognizing RHIM-containing proteins. In this report this is attributed to the ATPase activity of HSC70 but does not exclude the recognition of RIPK1 RHIM domain through a hydrophobic hexapeptide motif. This could be explored by reconstitution of RIPK1 KO MEFs with a RHIM mutant version of RIPK1 or any equivalent strategies.

Reviewer #2

(Remarks to the Author)
TFN signaling is critical for cells to regulate pathogen infection as part of an immune response. One way to control pathogens is to induce death of infected cells. Needless to say, TNF signaling thus must be tightly controlled to prevent excessive responses/cell death. Here, the authors identify another mechanism to turn off TNF signaling via packaging TNF receptor complexes into ILVs in MVBs followed by their secretion as EVs, in a process the authors call eMI.
In more detail, the authors show that KO of several ESCRT subunits (but not ESCRTII) causes cell death upon treatment with TNF in culture via extrinsic, RIPK1 independent apoptosis. It is also independent of a NFkB transcriptional response or Atg9 dependent macroautophagy. It is thus hypothesized that the TNF complex II inducing apoptosis is inactivated via incorporation into ILV in endosomes. This is supported by showing that blocking ILV formation is sufficient to induce apoptosis by TNF. Similarly, upon increasing the size of MVBs using Rab5Q79L that blocks maturation of endosomes, ESCRT activity was found necessary for the localization of active Casp8 to the inside of Rab5 positive endosomes and an increase of TNF complex II, consistent with eMI (or rather the secretion of complex II in EVs, as shown). The authors further show that complex II removal also depends on HSC70 (but likely not its ATPase activity), TAX1BP1 and ubiquitination of RIPK1, all consistent with a function of TAX1BP1 in eMI in additional to its previously identified role in MA mediated TNF complex degradation. Lastly, it is shown that bacterial proteins of S. typh and Mtb that interfere with ESCRT function are sufficient to prevent ILV formation and thus sensitize cells to TNF (increasing apoptosis), indicating that these bacterial proteins are key to mediating antibacterial clearance by death of the host cells. Importantly, catalytically inactive variants of the toxins or variants unable to interact with the ESCRT machinery do not show this effect, making a very strong argument about the mechanism.
Overall this is a very well written article with clean data showing these intriguing and highly relevant findings that are of great interest for a general readership including immunologists, microbiologists, as well as proteostasis people. Nevertheless, some items need to be addressed (detailed below). Most importantly, the authors use the term endosomal microautophagy without ever showing endosomal or lysosomal degradation (which would be interesting per se). Rather than true eMI, their process, EV generation and secretion, is requiring the same initial machinery, but ends up in exosome secretion rather than degradation and thus does not follow the strict definition of eMI.

Major:
• Since the effort is on EV formation upon MVB formation, it has to be questioned why the process is called Microautophagy. Simple ‘export’ rather than degradation. Thus, unless degradation is shown well, this is not eMI in the strict sense. It is not addressed here if true eMI actually also contributes to desensitization: is the complex stabilized in purified late endosomes upon inhibition of proteases? Perhaps in conjunction with prevention of exosome secretion? This would be required for the exact mechanism, although the authors show that protection from apoptosis is already achieved by sequestration of complex II from the cytoplasm.
• P8 Line 18 statement about effects or Rab variants on eMI degradation: There is a fast eMI that is starvation inducible and a slower one that is not. In addition to lacking a reference, it needs to be made sure that this statement is true in general.
• P9 Line 10…: the vps34 inhibitor argument about MA not being involved (Fig. 2K, not 1K) makes only sense if it shown that RabS34N itself does not affect MA (as it messes up the endosomal system).
• P14 Line 22…: While it is clear that the bacterial toxins that inhibit ESCRT components sensitize for death, the interpretation that this effect is lost upon treatment with the ESCRT inhibitor USP8i is flawed and needs to be revised: both show the same effect that at one point is saturated. A geneticist would argue that the factors act in the same pathway. More telling would be a test for synergy using low doses of the inhibitor with sub-effect expression level of the toxins.
• Discussion why ESCRT II lack has no effect: one of the proposed reasons (redundancy) is testable. ESCRT II is used by

all forms of eMI described in mammals and flies to the best knowledge of this reviewer.

Minor
• Fig 1F: no lambda PPase in figure (but in legend). It would also be helpful to systematically mention the cells used (e.g. MEFs or not e.g. in Fig 1G; Fig S3 H, I).
• Fig S3 C: resolution not good enough to see ILVs in what is shown as MVBs.
• Endosomal localization of components: as these are enlarged MVBs this should be noted and they should be called something along 'Rab5S34N-endosomes'.

Reviewer #3

(Remarks to the Author)
Dear Authors, in this manuscript, you propose that endosomal microautophagy (eMI) plays a protective role in TNF-induced cell death (TNF-ICD) by facilitating the removal of TNFR1 Complex II components via extracellular vesicles (EVs). This process appears to depend on HSC70 and the ESCRT machinery, ultimately resulting in the "detoxification" of cytotoxic signaling complexes.
You further demonstrate that checkpoint inhibitors and microbial effector proteins modulate this pathway, either enhancing or preventing TNF-ICD depending on their interaction with the ESCRT–eMI axis. Interestingly, you also provide a potential physiopathological situation sustaining your conclusions and propose that bacterial effectors from Salmonella Typhimurium and Mycobacterium tuberculosis can block this checkpoint and thereby promote TNF-induced death. This builds on prior work of your groupe (Bertrand et al.) showing that ATG9 and TAX1BP1 can redirect Complex II to lysosomal degradation, supporting a broader link between autophagy-related pathways and inflammatory cell death.
While the conceptual framework is promising and of potential interest to Nature Communications, there are several important issues that currently preclude acceptance of your manuscript in its current form. In particular:
• It is not yet clear whether the observed effects on TNF-ICD are directly due to EV-mediated detoxification of Complex II, or if they may instead result from alterations in TNFR1 trafficking, which could independently influence receptor signaling and cell death sensitivity.
• Additionally, it remains uncertain whether the vesicles analyzed under TNF treatment truly represent extracellular vesicles or apoptotic bodies, which could change the interpretation of the biochemical data you present.
I therefore recommend a major revision, and suggest the following points be addressed through additional experiments and clarifications:

Main comments:
• TNFR1 trafficking must be assessed: Given that many of the proteins under study (HSC70, ESCRT components, RAB5, microbial effectors) are involved in receptor trafficking, TNFR1 surface expression should be analyzed by flow cytometry across all relevant cell lines (parental, CRISPR/CAS9 KD/KO, and overexpression models). This is critical to rule out receptor mislocalization as a confounding factor. Figure 2g shows increased Complex I–bound TNFR1 in RAB5-S34N cells. This supports the above concern. Please provide TNFR1 surface levels in all experimental conditions.
• Clarify experimental design across conditions: There is inconsistency in the use of TNF Alone vs TNF + CHX, in WT or ATG9a–/– cells. A systematic side-by-side comparison of TNF-ICD in WT vs ATG9a–/– cells in the presence or absence of CHX would greatly improve interpretability and readability.
• Validate the nature of EVs:To confirm that the analyzed vesicles are not apoptotic bodies:
o Please include experiments using caspase inhibitors (e.g., ZVAD or QVD).
o Exclude Annexin V+ vesicles during EV isolation.
o EV profiling should be expanded to include the following TNFR1 CII components TRAF2, TRADD, c-FLIP, cIAP1, TRAF1, and compared to EVs obtained from cells stimulated with FasL and TRAIL to assess whether this detoxification process is TNF-specific.
• Broaden your discussion: Extend discussion to other ligands of the family ie : FasL and TRAIL ie the following published work (See https://www.cell.com/cell/abstract/S0092-8674(25)00728-7 and https://pubmed.ncbi.nlm.nih.gov/32807832/)

Specific comments :
Figure 1:
• Please include immunoprecipitation of TNFR1 at time zero from lyzed cells (Anti-FLAG + FLAG-TNF pulldown from untreated cellular extracts) for proper validation.
• Comment and confirm the reduced RIPK1 ubiquitination in TSG101-deficient cells. Is this reduction seen in the other experiments ? Have they been performed at least 3 times independently ?. If so please indicate so in the manuscript and show for the referee the other two repeats.
Figures S1S:
• It is unclear why VPS34 inhibition (with drug) enhances TNF-induced death in MEF cells, while not in HT1080 cells (e.g., S1U-V vs S1X).
• Please clarify these discrepancies and include side-by-side comparisons of VPS34 KD (gRNA) vs inhibitor treatments.
Figure 3:
• Were DGUC/SEC and EV analyses done in the presence or absence of CHX? This is not stated and is crucial for interpretation. If not a control including CHX would be needed as this would match confocal data.
• Include caspase inhibitors (e.g., ZVAD or QVD) to prevent apoptotic body formation and ensure that EV-associated Complex II components are not merely originating from apoptotic debris.
• Alternatively, exclude Annexin V+ vesicles from EV analysis to control for apoptotic contamination.

• Why is FADD not detected in HT1080 EVs (Fig. S3J)?
Figure 4:
• The presence of HSC70 in EVs is not shown. Only indirect evidence via sensitivity to TNF is provided to point to HSC70 as a cargo protein for eMI. Please include HSC70 in your biochemical EV analyses.

Minor Comments:
• Clearly label each panel with the relevant cell line (MEF, HT1080, etc.), as it is not always obvious.
• Figure S1S: Immunoblots showing TSG101 deficiency in MEF-ATG9a–/– cells should include WT MEFs for reference.
• Figure 3g: The caspase-8 blot shows three bands—please clarify the molecular identity of each (e.g., p55, p43/p41, p18).
• Figure 5:Textual Issue: The sentence "…sensitizing effects of SopB and EsxG-H were lost when cells were pre-treated with USPsi…" contradicts Fig. 5b vs 5e. At most, you can conclude that SopB's effect is additive to or masked by USP8 inhibition, but not abolished. Please revise for accuracy.
• "..The discrepancy between the two studies concerning the effect of ESCRT deletion on TNF-mediated NF- κB/MAPK signaling is unclear, but may reflect the cell type-specific targeting of TNFR1 Complex Ito the endolysosomal system…" Which studies, please add references.
• "…phosphorylation of RIPK3 and MLKL (markers of necroptosis induction) were not observed upon stimulation of either wild type (WT) or ESCRT-deficient MEFs with TNF…" Could you dd the precision of the time at which you did this analysis as this may not be true at other time points !
• The Original title: "Endosomal micro-phagy prevents TNF-induced cell death by targeting TNFR1 complex II" is vague, could you consider the following one "Endosomal Microautophagy Limits TNF-Induced Apoptosis by Redirecting TNFR1 Complex II for Exosomal Secretion" to describe ore precisely your conclusions ?

Reviewer #4

(Remarks to the Author)
The manuscript "Endosomal micro-autophagy prevents TNF cytotoxicity by targeting TNFR1 complex II" by Huyghe etal explores a novel and interesting aspect of the prevention of TNF cytotoxicity by ESCRT and HSC-70 dependent endosomal microautophagy. The manuscript has sufficiently explained the novel mechanism and following are minor points to be included
1. Graphical abstract to explain the mechanism in the manuscript
2. More detailed explanation of medical implications of the pathway explored is needed
3. Fig 1 shows USP8 inhibitor, along with the TNF, induces TNF-mediated cell death. Additionally, TNF-mediated cell death is been induced in ESCRT mutant components.
Fig 1c- Why there are repeated cleaved Casp. 3 blots? It is mentioned in the text (page 5, line 25) for the PARP but not shown in the results.
Fig 1b, c, d- No information for the used cell lines.
Most of the graphs of % cell death (Fig 1e, extended 1g) show high cell death (more than or up to 50%) in the DMSO. How is it true?
Page 6, line 23 "In contrast to….". This statement cannot be aligned with the fig 1f.
Ext. fig. 1J- TNFR1 blot is not clear

Version 1:

Reviewer comments:

Reviewer #1

(Remarks to the Author)
The authors have adequately addressed several of the major points I previously raised whereas others are still in need of the authors' additional attention as laid out in my point-by-point (points 1-7) reply to their response.

1. Regarding my original point 1 ("In order to clarify the cytoprotective function of USP8..."), the authors now present sufficient evidence to address this point and the further sensitization of TNF+CHX treatment with USP8 inactivation is highly suggestive of an independent role of USP8 besides cFLIP stabilization.

2. Regarding my original point 2 ("The protective role of ESCRT components on TNF-induced necroptosis should be further explored..."), if the WT MEFs die at < 50% with TNF+zVAD, this means they are capable of dying by necroptosis. If the detoxification pathway in ILVs is via RIPK1 and its M1-Ub (and RIPK1 is necessary in complex II to allow for necroptosis), how can disruption of this pathway not impact cell death? This remains to be clarified.

3. My original point 3 ("The addition of cycloheximide was tested for MEFs...") has been addressed adequately and sufficient evidence in support of the authors' claims has been provided.

4. Regarding my original point 4 ("In Figure 3, the authors aim to demonstrate the presence of TNF complex II..."), the claim that FADD and EEA1 colocalize is not substantiated by the data presented. For this claim to be made there would need to be a 3D reconstitution or other type of analysis. In the presented images it could well be that they are simply sitting on top of

each other. The presence of Caspase 8 and FADD in basal conditions is the control for this experiment. The authors show that only in TNF they co-immunoprecipitate, yet they are still there in the control. In my view, addressing thi spoint is not only in the scope of this study but needs to be adequantely addressed in it.

5. My original point 5 ("Later, the authors explore the fate of the vesicles..." The addition of cycloheximide was tested for MEFs ...") has been addressed adequately and sufficient evidence in support of the authors' claims has been provided.

6. Regarding my original point 6 ("In Figure 4, a model is proposed where M1-Ub RIPK1 is proposed as a scaffold for TAXBP1..."), to formally conclude that Lin-Ub is necessary for the cargo recognition, the possibility that Lin-Ub is involved in the formation and stability of these vesicles would need to be excluded. In the figures shown in 5f the number of vesicles in the mCherry-RAB5Q79L HOIP gRNA conditions appears significantly lower than in other genotypes (in addition, not only the number but also size of these vesicles should be evaluated). The authors show a clear decrease in cleaved Caspase 8 punctae in RIPK1 KO cells. Furthermore, they see RIPK1 KO sensitizing Atg9a-deficient MEFs. However, does RIPK1 KO MEFs WT vs Atg9a look any different in terms of cell death sensitivity? This important controls for the cell death experiments needs to be included.

7. My original point 7 ("On the contrary, in previous work not mentioned in this manuscript...") has been addressed adequately and sufficient evidence in support of the authors' claims has been provided.

There are a few additional points that also require the authors' attention:

8. No pRIPK1 is shown in any input blot, only in the IPs. This is, however, an essential control and needs to be included.

9. The exepriments for all these graphs added in the revision to check TNFR1 degradation employ exogenous TNF-V5. The authgors need to test whether or not the V5 tag plays a role in rendering TNFR1 more susceptible to degradation via selective autophagy.

Reviewer #2

(Remarks to the Author)
The authors addressed my comments and overall added many more experiments substantiating their findings. On a side note, the discussion is now very long, but in part due to the coments of the initial reviewers (including myself).

I have only two minor comments:

Legend of Fig S3A: The asterisk is not explained. I assume that that band is abackgroundband and the real one is the smaller one for FADD.

P14, line 25: TNF treatment leads to targeting of receptor complex II to exosomes…. Delete the word ‘selective’ or specify why selective. There likely still are other proteins in these exosomes. Key is, the complex goes there.

(See attachment for my comments to Rev#1’s report)

Reviewer #3

(Remarks to the Author)
Dear Authors, thanks for your extensive revised version of the manuscript intitled “Exosomal Export of TNFR1 Complex II Prevents TNF-Induced Cytotoxicity”

Despite extensive revision, your manuscript still does not fully support your claim that complex II is being “detoxified” at the steady state preventing TNF-induced cell death. First of all because you fail to demonstrate convincingly that the complex II is present in these vesicles.

Please find enclose my comments to your answers (C:)

1. It is not yet clear whether the observed effects on TNF-ICD are directly due to EVmediated detoxification of Complex II, or if they may instead result from alterations in TNFR1 trafficking, which could independently influence receptor signaling and cell death sensitivity. TNFR1 trafficking must be assessed: Given that many of the proteins under study (HSC70, ESCRT components, RAB5, microbial effectors) are involved in receptor trafficking, TNFR1 surface expression should be analyzed by flow cytometry across all relevant cell lines (parental, CRISPR/CAS9 KD/KO, and overexpression models). This is critical to rule out receptor mislocalization as a confounding factor. Figure 2g shows increased Complex I–bound TNFR1 in RAB5-S34N cells. This supports the above concern. Please provide TNFR1 surface levels in all experimental conditions.
> We thank the reviewer for this important remark. TNFR1 has indeed been reported to engage the endolysosomal pathway, and its trafficking is therefore likely affected under several of the conditions we examined. To distinguish defective ILV-mediated detoxification of TNFR1 Complex II from altered TNFR1 trafficking as the cause of the observed TNF cytotoxicity, we directly tested whether blocking TNFR1 trafficking impacts cell viability. First, we verified that TNFR1 is normally targeted for lysosomal degradation by comparing its protein levels in the presence or absence of the lysosomal fusion inhibitor Bafilomycin A1 (BafA1) (Fig. 3c). Notably, preventing lysosomal delivery of TNFR1 with BafA1 did not affect cell viability (Fig. 3d). We next interfered with earlier steps of TNFR1 transport through the endosomal system. Inhibition of

TNFR1 sorting into ILVs through loss of the ESCRT-II subunit VPS36—whose absence does not impairTNFR1 Complex II targeting—stabilized TNFR1 (Fig. 3e) but again failed to induce TNF cytotoxicity (Fig. 3f). Finally, blocking DYNAMIN-1–mediated endocytosis of TNFR1 using the pharmacological inhibitor Dyngo-4a similarly prevented receptor degradation without triggering cell death (Fig. 3g-h). Consistent results were obtained using the dominant-negative DYN1S45N mutant or the dominant-negative AP180 C-terminal fragment (Extended Data Fig. 3c-d). Together, these results demonstrate that interfering with TNFR1 trafficking at multiple stages of the endolysosomal pathway does not induce cell death.
> To further demonstrate that the cytoprotective effect of the ESCRTs is independent from the ESCRT-dependent sorting of TNFR1 into ILVs, we tested the effect of ESCRT inhibition by USP8i in cells in which TNFR1 internalization is blocked by Dyngo-4a. Remarkably, USP8i treatment still sensitized the cells to TNF-mediated apoptosis in these conditions (Fig. 3i-j).
> Together, these results demonstrate that the formation and cytotoxic potential of TNFR1 Complex II are independent of TNFR1 trafficking. The phenotypes observed in the manuscript cannot be attributed to receptor mislocalization but instead support the model in which the ESCRT machinery prevents TNF cytotoxicity by mediating the ILV-dependent sequestration and ultimately extracellular release- of TNFR1 Complex II. These new data have been incorporated into an entirely new figure in the revised manuscript dedicated to dissecting TNFR1 trafficking and its relationship to TNF-induced cell death (Fig. 3 and Extended Data Fig. 3).
> Given these findings, we believe that measuring TNFR1 surface expression by flow cytometry, as suggested by the reviewer, would not meaningfully contribute to the interpretation of our data. Moreover, ESCRT deficiency, RAB5S34N expression, or microbial effector proteins do not impair internalization; they act downstream, by preventing ILV formation and thereby trapping receptors within endosomal compartments—not at the plasma membrane.

C: While the additional experiments addressing TNFR1 trafficking are appreciated, they do not fully resolve the concern raised. In particular, the authors do not directly address the observation highlighted in Figure 2g, where increased amounts of TNFR1 are detected in Complex I in RAB5-S34N–expressing cells. This point is neither discussed nor reconciled with the revised model, and the removal of the TNFR1 blot from the revised figure (now Fig. 2h) further complicates interpretation. More fundamentally, although TNFR1 is not retained within Complex II after engagement, the formation of TNFR1 Complex II depends on multiple upstream events, including proteolytic processing of TNFR1 by γ-secretases (see doi:10.1074/jbc.M115.679076). The activity, localization, or availability of these proteases, as well as the pool of membrane-associated TNFR1, could plausibly be altered in cells stably expressing RAB5-S34N or lacking ESCRT components. Such effects could influence Complex II formation independently of ILV-mediated sequestration
Given that most experimental models used in this study involve stable expression or stable knockout of trafficking regulators, a direct assessment of TNFR1 surface expression would be straightforward and informative. Measuring membrane-bound TNFR1 by flow cytometry across the relevant conditions would help exclude the possibility that variations in TNFR1 availability at the plasma or altered receptor processing contribute, at least in part, to the increased sensitivity to TNF-induced cell death. Moreover, because the presence of TNFR1 Complex II within extracellular vesicles has not been fully established, in my opinion (see point 10) such controls remain important to support the authors' central conclusion.

2. Clarify experimental design across conditions: There is inconsistency in the use of TNF Alone vs TNF + CHX, in WT or ATG9a–/– cells. A systematic side-by-side comparison of TNF-ICD in WT vs ATG9a–/– cells in the presence or absence of CHX would greatly improve interpretability and readability.
> We thank the reviewer for this helpful suggestion. To improve clarity, we have updated the labeling of all relevant figure panels to more clearly indicate the specific treatment conditions (TNF alone vs. TNF + CHX) and the corresponding genotypes (WT vs. ATG9Adeficient cells). This should enhance interpretability and ensure consistent comparison across experiments. Regarding a systematic side-by-side analysis of TNF-induced cell death in WT and ATG9A-deficient cells with or without CHX, we would like to refer the reviewer to our previous work (Huyghe et al., Science 2022, Fig. 1H), in which this comparison was performed.
C: The clarification regarding figure annotation and labeling is appreciated. However, the second part of my concern is not fully addressed. While reference to prior work is useful, the current manuscript would benefit from a clear, systematic side-by-side comparison of TNF-induced cell death in WT and ATG9A-deficient cells, in the presence and absence of CHX, performed within the same experimental framework. Such direct comparison would substantially improve the readability and interpretability of the results and would allow the reader to assess more clearly how ATG9A loss modulates TNF-induced cytotoxicity under different conditions. Relying exclusively on previously published data limits the self-contained nature of the present study.

3. Validate the nature of EVs: To confirm that the analyzed vesicles are not apoptotic bodies: Please include experiments using caspase inhibitors (e.g., ZVAD or QVD). Exclude Annexin V+ vesicles during EV isolation.
> We agree with the reviewer that it is essential to exclude the possibility that the TNFR1 Complex II components detected in EVs originate from apoptotic bodies released by dying cells. Several lines of evidence in the original submission already argue strongly against this interpretation. First, HT1080 cells do not undergo apoptosis in response to the TNF concentration used in this study, as previously shown (Huyghe et al., Science 2022, Fig. S5C) and confirmed again in the revised manuscript (Extended Data Fig. 4r). Notably, this remains true at the 6-hour time point used for EV isolation. Second, apoptotic bodies are substantially larger than exosomes; however, nanoparticle tracking analysis (NTA) revealed no increase in EV particle size following TNF stimulation (Extended Data Fig. 4x). Third, ultrastructural analysis by electron microscopy showed no appearance of apoptotic bodies or morphological alterations in EV preparations from TNF-treated cells (Extended Data Fig. 3w). To address the reviewer's concern even more directly, we generated CASP3/7-deficient HT1080 cells, which are completely resistant to TNF-induced apoptosis (Extended Data Fig.
4t) and therefore incapable of forming apoptotic bodies. EVs isolated from these apoptosis resistant cells after TNF treatment still contained activated (Cl.) CASP8 (Extended Data Fig.
4u), definitively ruling out contamination by apoptotic bodies as the source of TNFR1 Complex II components. These new results have been added to the revised manuscript.

C: The additional experiments using CASP3/7-deficient cells and the accompanying analyses are appreciated and represent a step toward addressing the concern that the detected vesicles might originate from apoptotic cells. Nevertheless, the evidence provided remains incomplete. Rather than reporting average EV size distributions, the manuscript should present full particle size distributions under all relevant conditions, including WT cells, CASP3/7-deficient cells, and cells treated with a pan-caspase inhibitor, to more rigorously exclude contamination by vesicles derived from spontaneous or low-level cell death, which is common in cell culture. Moreover, the central concern remains that FADD is absent or barely detectable in EVs isolated by size exclusion chromatography, even following TNF stimulation. While the DGUC experiments are convincing in that FADD, CASP8, and RIPK1 (including modified forms) are detected in specific density fractions after TNF treatment (Fig. 4g), the manuscript would be strengthened by additional comparisons. In particular, given that both SEC and DGUC experiments were performed in the absence of CHX, it would be informative to include DGUC analyses in the presence of CHX and in the presence of a pan-caspase inhibitor, in both WT and CASP3/7-deficient cells. These controls would help exclude the possibility that apoptotic vesicles of exosome-like size contribute to the detected signal.
Similarly, extending DGUC analyses to WT and RAB5-S34N MEFs, with and without TNF stimulation, would further strengthen the claim that EV-mediated detoxification of TNFR1 Complex II operates under the proposed conditions.

4. EV profiling should be expanded to include the following TNFR1 CII components TRAF2, TRADD, c-FLIP, cIAP1, TRAF1, and compared to EVs obtained from cells stimulated with FasL and TRAIL to assess whether this detoxification process is TNF-specific.
> We thank the reviewer for this suggestion. Regarding TRADD, the reviewer may have overlooked that its presence in EVs was already shown in the original manuscript (Extended Data Fig. 4p). Regarding the additional components proposed by the reviewer, many of these - such as TRAF2, cIAP1 and TRAF1 - are primarily associated with TNFR1 Complex I rather than being bona fide components of TNFR1 Complex II. In contrast, our profiling already demonstrates the presence of multiple wellestablished TNFR1 Complex II components (RIPK1, M1-Ub, TRADD, FADD, Cl. CASP8, TAX1BP1), providing strong evidence that the EVs indeed contain TNFR1 Complex II. Nevertheless, to address the reviewer's concern, we performed additional SECbased EV isolations and examined the suggested proteins. While all factors were readily detectable in cell lysates, only TRAF2 exhibited a clear TNF-dependent enrichment in EVs. These new results have been incorporated into the revised manuscript (Extended Data Fig. 4q).
> We do not believe that comparing EV cargoes from TNF-, FasL-, or TRAIL-treated cells would yield mechanistically meaningful insight. It is well established that TNFR1 Complex II is a cytosolic complex that forms after dissociation of TNFR1 Complex I components from the receptor and their subsequent association with FADD and CASP8 (Micheau and Tschopp, Cell 2003). This distinguishes
TNF signaling from FasL and TRAIL, whose DeathInducing Signaling Complexes (DISCs) remain receptor-bound. The molecular mechanism that brings TNFR1 Complex II into EVs is therefore intrinsically different from the one that would bring those DISCs into EVs. To exclude the possibility that a DISC-like complex could eventually form at TNFR1 when ILV formation is blocked, we performed additional TNFR1 Complex I immunoprecipitations in Tsg101-deficient MEFs. Even at time points where activated (Cl.) CASP8 is readily detectable in the cytosol, we did not detect CASP8 or Cl. CASP8 in TNFR1 Complex I (Fig. 3b), demonstrating that DISC formation does not occur at the level of TNFR1 even when endolysosomal trafficking is impaired. For these reasons, we consider cross-comparison with FasL- or TRAIL-derived EVs not informative for the present study

C: Thank you, indeed TRADD was present. However, if TRADD originates from TNFR1 Complex II, one might expect it to display post-translational modifications, as reported in previous studies (e.g., Fullsack et al., doi:10.1038/s41419-019-1396-5; Micheau and Tschopp, Cell 2003). The absence of such modifications raises questions about the nature of the TRADD species detected in EVs. The authors may wish to consider testing additional TRADD antibodies to assess whether modified forms can be detected. Conversely, the newly presented data show post-translationally modified TRAF2 in EVs (Fig. 4q). It remains unclear to me whether this observation is expected or mechanistically consistent with the proposed model. Additional clarification or discussion would be helpful.
While the technical effort required to extend EV profiling to FasL- or TRAIL-stimulated cells is appreciated, the rationale for excluding such comparisons is not entirely convincing. Although the reviewer fully recognizes the mechanistic differences between TNFR1 Complex II and DISC formation downstream of Fas or TRAIL receptors, it remains plausible that cells may deploy similar protective strategies to eliminate cytotoxic signaling complexes under suboptimal or limiting activation conditions. In this context, it would not be surprising if ESCRT inhibition also sensitized cells to FasL- or TRAIL-induced cell death. At minimum, this possibility should be discussed explicitly; ideally, limited experimental testing would further strengthen the manuscript.
5. Broaden your discussion: Extend discussion to other ligands of the family ie : FasL and
TRAIL ie the following published work (See https://www.cell.com/cell/abstract/S0092-8674(25)00728-7 and https://pubmed.ncbi.nlm.nih.gov/32807832/)

C: The authors are encouraged to expand the Discussion to more explicitly consider whether EV-mediated disposal of cytotoxic signaling complexes might represent a broader protective mechanism applicable to other death ligands, such as FasL and TRAIL, as suggested by recent literature (e.g., Cell S0092-8674(25)00728-7; PMID:32807832). Even if not experimentally addressed here, a thoughtful discussion would help place the current findings in a broader biological context.

6. Please include immunoprecipitation of TNFR1 at time zero from lyzed cells (Anti-FLAG + FLAG-TNF pulldown from untreated cellular extracts) for proper validation.
> The control suggested by the reviewer is sometimes included in TNFR1 Complex I immunoprecipitation experiments, but

our laboratory (Priem et al., Methods Mol. Biol., 2018) and others have routinely used the control strategy presented in our original manuscript without encountering difficulties in data interpretation or observing meaningful differences relative to time-zero controls. Importantly, none of our conclusions depend on subtle variations in baseline TNFR1 pulldown efficiency, and the newly added data - which formally demonstrate that ILV formation regulates TNF cytotoxicity independently of TNFR1 trafficking - further reinforce that including an additional time-zero control would not alter the interpretation of the results. For these reasons, we respectfully believe that repeating all TNFR1 immunoprecipitations with an additional time-zero control would not provide added value or lead to different conclusions.
Comment and confirm the reduced RIPK1 ubiquitination in TSG101-deficient cells. Is this reduction seen in the other experiments? Have they been performed at least 3 times independently? If so please indicate so in the manuscript and show for the referee the other two repeats.
> While we acknowledge the reviewer's observation of a modest decrease in RIPK1 ubiquitination in TNFR1 Complex I at 5 minutes after TNF stimulation in TSG101-deficient cells, we do not consider this difference biologically meaningful. The apparent reduction correlates with a slightly lower overall amount of TNFR1 and associated TNFR1 Complex I components recovered in the immunoprecipitation from TSG101-deficient cells, suggesting a technical rather than functional origin. Importantly, we do not observe RIPK1 activation within TNFR1 Complex I - a hallmark of defective ubiquitination - and TNF-induced cell death in TSG101-deficient cells is clearly RIPK1 kinase-independent (Fig. 1e-f). Both observations support the conclusion that RIPK1 ubiquitination remains functionally intact in the absence of TSG101. As indicated in the figure legends, all western blot experiments were performed independently at least twice. For transparency, we now provide the data from the additional independent repeat for the reviewer's inspection (Rebuttal Fig. 1).

C: While the authors note that time-zero TNFR1 immunoprecipitation controls are not routinely included in their experimental workflow, the inclusion of such controls would help exclude the possibility of steady-state differences in TNFR1 abundance or trafficking prior to TNF stimulation. This is particularly relevant given the observed alterations in MAPK/NF-κB signaling, TNFR1 levels, and RIPK1 ubiquitination in some experimental conditions (e.g., Fig. 2i; comparison of RAB5-WT and RAB5-S34N cells). Inclusion of a time-zero control would therefore strengthen confidence that the reported differences arise from TNF-induced signaling rather than pre-existing alterations.
7. Comment and confirm the reduced RIPK1 ubiquitination in TSG101-deficient cells. Is this reduction seen in the other experiments? Have they been performed at least 3 times independently? If so please indicate so in the manuscript and show for the referee the other two repeats.
> While we acknowledge the reviewer's observation of a modest decrease in RIPK1 ubiquitination in TNFR1 Complex I at 5 minutes after TNF stimulation in TSG101-deficient cells, we do not consider this difference biologically meaningful. The apparent reduction correlates with a slightly lower overall amount of TNFR1 and associated TNFR1 Complex I components recovered in the immunoprecipitation from TSG101-deficient cells, suggesting a technical rather than functional origin. Importantly, we do not observe RIPK1 activation within TNFR1 Complex I - a hallmark of defective ubiquitination - and TNF-induced cell death in TSG101-deficient cells is clearly RIPK1 kinase-independent (Fig. 1e-f). Both observations support the conclusion that RIPK1 ubiquitination remains functionally intact in the absence of TSG101. As indicated in the figure legends, all western blot experiments were performed independently at least twice. For transparency, we now provide the data from the additional independent repeat for the reviewer's inspection (Rebuttal Fig. 1).
C: The authors in their rebbutal show a second immunoblot after TNF IP, which like the first one displays limited but consistent reduction of ubiquitinated RIPK1 in TSG101 KO cells. And contrary to their rebuttal, this clearly suggests that ESCORT I KO affects also complex I formation, potentially explaining the subtle differences observed in MAPK and NFkB signalling (see pont N°16). One may also conclude from this observation that in TSG101 KO cells, the transition from CI to CII occurs faster than in WT cells, which would also fit the proposed working hypothesis.

8. It is unclear why VPS34 inhibition (with drug) enhances TNF-induced death in MEF cells, while not in HT1080 cells (e.g., S1U-V vs S1X). Please clarify these discrepancies and include side-by-side comparisons of VPS34 KD (gRNA) vs inhibitor treatments.
> We acknowledge the reviewer's observation that VPS34 inhibition results in higher TNF cytotoxicity in MEFs than HT1080 cells. Although the basis for this difference remains unclear, the effect is consistent with the results of our previous study, where it was found not to be specific to VPS34. Indeed, similar differences were also observed upon inactivation of other components of the LC3-independent macro-autophagy pathway, such as ATG9A or FIP200 (Huyghe et al., Science 2022 - Fig. S1F–G). These results therefore indicate that HT1080 cells are more resistant than MEFs to inactivation of the LC3-independent macroautophagy checkpoint. In the same study, we also directly compared pharmacological inhibition of VPS34 with SAR405 to CRISPR-mediated VPS34 deletion and observed no substantial differences between these two approaches (Huyghe et al., Science 2022 - Fig. 2C, Fig. S3E–F).

C: Ok fine
9. Were DGUC/SEC and EV analyses done in the presence or absence of CHX? This is not stated and is crucial for interpretation. If not a control including CHX would be needed as this would match confocal data.
> We thank the reviewer for highlighting the importance of clarifying whether CHX was used during EV isolation. As stated in the original figure legends, all DGUC/SEC-based EV isolations were performed exclusively on cells not pre-treated with CHX. CHX was only used in microscopy experiments, where it is necessary to amplify CASP8 activation sufficiently to allow reliable detection of Cl. CASP8 puncta by confocal imaging. Without CHX, the signal remains below the single-cell detection threshold. To directly bridge the microscopy and EV datasets, we additionally performed EV isolations from TNF-treated cells in the presence of CHX. As expected, CHX pre-treatment increased the amount of Cl. CASP8 detected in EVs (Extended Data Fig. 4s), consistent with its ability to increase the cellular pool of TNFR1 Complex II above the imaging detection limit. However, CHX is a potent sensitizer of apoptosis (Extended Data Fig. 4r), raising the possibility of apoptotic body contamination— a concern also raised by the reviewer. To exclude this, we repeated the experiment in apoptosis-resistant CASP3/7-deficient HT1080 cells (Extended Data Fig. 4t), which cannot form apoptotic bodies. Even in this

background, CHX pre-treatment similarly increased Cl. CASP8 levels in EVs (Extended Data Fig. 4u) confirming that the additional signal does not originate from apoptotic bodies. Importantly, and central to our conclusions, TNFR1 Complex II is readily detectable in EVs even in the absence of CHX, demonstrating that this phenomenon does not depend on artificially boosting CASP8 activation. We have now incorporated these additional data into the revised manuscript for clarity (Extended Data Fig. 4r-u).

C: Thanks for your answer. The clarification regarding the use of CHX during DGUC/SEC-based EV isolations is indeed important. Please see new comment (Point N°3). I would probably have generated a caspase-8 KO HT1080 cell line and re-introduced a Protease-deficient version of the caspase-8 to formely demonstrate that ILV-mediated secretion of complex II is occuring independent of caspase activation.
10. Why is FADD not detected in HT1080 EVs (Fig. S3J)?
> A closer examination of the figure (Extended Data Fig. 4p) shows weaker bands corresponding to FADD, which is consistent with our other data on the EV content of TNFtreated cells. The reduced signal in this particular experiment most likely reflects a shorter exposure time. Importantly, the EV lysates analyzed in Fig. 4h were prepared using the same SEC protocol and show much stronger FADD signals, confirming the reproducibility of this finding.
C: You are right, looking closer shows a feint band of FADD, yet there is no change whether the cells have been treated or not with TNF, arguing against your working hypothesis, as one may expect to see some more in stimulated cells, given that complex II is not supposed to be formed in the absence of TNF.
11. The presence of HSC70 in EVs is not shown. Only indirect evidence via sensitivity to TNF is provided to point to HSC70 as a cargo protein for eMI. Please include HSC70 in your biochemical EV analyses.
> We thank the reviewer for this valuable suggestion. In the original manuscript, we demonstrated that HSC70—independently of its ATPase activity—is required for the transfer of TNFR1 Complex II into endosomes and for protection against TNF cytotoxicity. However, we agree that directly assessing the presence of HSC70 in EVs provides important complementary evidence. To address this, we performed additional biochemical analyses of EVs isolated by SEC following TNF stimulation. In addition to the expected TNF-dependent enrichment of linear ubiquitin chains, ubiquitinated RIPK1, and TAX1BP1, we now observe increased levels of HSC70 in SEC fractions enriched for established exosome markers (TSG101, ALIX, and FLOTILLIN-1) (Fig. 5a). These findings are fully consistent with the proposed role of HSC70 in selectively facilitating the loading of TNFR1 Complex II into ILVs. The new data have been incorporated into the revised manuscript.
C: Thanks for adding HSC70

12. Clearly label each panel with the relevant cell line (MEF, HT1080, etc.), as it is not always obvious.
> We thank the reviewer for this suggestion and have updated the labeling of the figure panels accordingly to improve interpretability and readability.
C: Thanks for the labelling, the figures are now clearer
13. Figure S1S: Immunoblots showing TSG101 deficiency in MEF-ATG9a–/– cells should include WT MEFs for reference.
> The purpose of introducing TSG101 deficiency into ATG9A-deficient MEFs was solely to assess the impact of ESCRT loss in a macro-autophagy-deficient background. At no point in the manuscript do we directly compare ATG9A-deficient cells with wild-type MEFs. For this reason, we respectfully disagree that including such a comparison would represent an informative control or alter the interpretation of our results.
C: Albeit, I respectfully disagree with your objection, but given that more important experiments are still needed to convince the referee of your finding I will consider this concern closed (see above comments).

14. Figure 3g: The caspase-8 blot shows three bands—please clarify the molecular identity of each (e.g., p55, p43/p41, p18).
> We thank the reviewer for this suggestion and have updated the labeling of the figure panels accordingly to improve interpretability and readability.
C: Thanks for this, yet, you give no explanation why 3 bands are seen in this blot, while one may expect only two !

15. Figure 5: Textual Issue: The sentence "…sensitizing effects of SopB and EsxG-H were lost when cells were pre-treated with USPsi…" contradicts Fig. 5b vs 5e. At most, you can conclude that SopB's effect is additive to or masked by USP8 inhibition, but not abolished. Please revise for accuracy.
> We agree with the reviewer that the interpretation of these results was not correctly phrased in this sentence. We have therefore rephrased to increase clarity: "This notion was further supported by the fact that SopB and EsxG-H have no additional sensitizing effect when the ESCRT machinery is already inhibited by pre-treatment with USP8i (Fig. 6e and Extended Data Fig. 6h)."- Page 17, lines 29-31.
C: Thanks for this revision
16. "..The discrepancy between the two studies concerning the effect of ESCRT deletion on TNF-mediated NF- κB/MAPK signaling is unclear, but may reflect the cell type-specific targeting of TNFR1 Complex Ito the endolysosomal system…" Which studies, please add references.
> We note that the study by Song et al. was referenced earlier in the manuscript when discussing discrepancies with our findings. However, we agree with the reviewer that an additional citation at this point is helpful to improve clarity and have therefore added this to the revised manuscript.
C: Thank you

17. "…phosphorylation of RIPK3 and MLKL (markers of necroptosis induction) were not observed upon stimulation of either wild type (WT) or ESCRT-deficient MEFs with TNF…" Could you dd the precision of the time at which you did this analysis as this may not be true at other time points!
> We thank the reviewer for pointing this out and have rephrased the Results section to describe the data more precisely.

C: Thank you

18. The Original title: "Endosomal micro-phagy prevents TNF-induced cell death by targeting TNFR1 complex II" is vague, could you consider the following one "Endosomal Microautophagy Limits TNF-Induced Apoptosis by Redirecting TNFR1 Complex II for Exosomal Secretion" to describe ore precisely your conclusions?
> We thank the reviewer for this constructive comment. In line with remarks from other reviewers, we performed additional experiments to evaluate whether ESCRT-dependent sequestration of TNFR1 Complex II into ILVs leads to lysosomal degradation. The new results indicate that this process primarily mediates extracellular release of the complex via exosomes rather than lysosomal targeting. These data have been added to the revised manuscript. Since this mechanism does not strictly qualify as autophagy, we have rephrased the title and adjusted the manuscript to more accurately reflect our findings: “Exosomal Export of TNFR1 Complex II Prevents TNF-Induced Cytotoxicity”.

C: Thank you, this title would better fit your working hypothesis

Reviewer #4

(Remarks to the Author)
The manuscript titled "Exosomal Export of TNFR1 Complex II Prevents TNF-Induced Cytotoxicity" by Huyghe etal has explored the mechanism of ESCRT pathway and HSC70 for exosome mediated disposal of CASPASE-8 activating TNFR1 Complex II and provides detailed analysis of Salmonella Typhimurium and Mycobacterium tuberculosis mediated TNF cytotoxicity. The reviewers comments have been sufficiently addressed and therefore the manuscript can be accepted after revision.

Reviewer #5

(Remarks to the Author)
I co-reviewed this manuscript with one of the reviewers who provided the listed reports. This is part of the Nature Communications initiative to facilitate training in peer review and to provide appropriate recognition for Early Career Researchers who co-review manuscripts.

Version 2:

Reviewer comments:

Reviewer #1

(Remarks to the Author)
Dear Dr Bertrand, dear Mathieu and colleagues,

all points I raised in my review of the revised verson of your manuscript have been adequately addressed in this re-revised version.

My congratulations on an superb study!

Best wishes,

Henning

Reviewer #3

(Remarks to the Author)
Your revised manuscript does not adequately address the major concerns raised in my initial review, and significant mechanistic and experimental gaps remain unresolved. Your responses rely largely on speculative explanations rather than providing the necessary experimental evidence, particularly regarding the existence, integrity, and specificity of TNFR1 Complex II within EVs. As a result, the central claim of your study remain insufficiently supported in the current revised version of your manuscript.

Open Access This Peer Review File is licensed under a Creative Commons Attribution 4.0 International License, which permits use, sharing, adaptation, distribution and reproduction in any medium or format, as long as you give appropriate credit to the original author(s) and the source, provide a link to the Creative Commons license, and indicate if changes were made.
In cases where reviewers are anonymous, credit should be given to 'Anonymous Referee' and the source.
The images or other third party material in this Peer Review File are included in the article's Creative Commons license, unless indicated otherwise in a credit line to the material. If material is not included in the article's Creative Commons license and your intended use is not permitted by statutory regulation or exceeds the permitted use, you will need to obtain permission directly from the copyright holder.
To view a copy of this license, visit https://creativecommons.org/licenses/by/4.0/

We thank the editor and reviewers for their positive feedback and for the constructive comments on our study. In response, we have conducted several additional experiments to address all points raised by the reviewers. The revised manuscript now includes 41 new or adapted figure panels, some of which have been assembled into an entirely new figure. We believe that these new data substantially strengthen the conclusions of the study and improve the overall quality of the manuscript.

Please find below our point-by-point responses to the reviewers' remarks. New figure panels and textual revisions in the manuscript are highlighted in **blue**.

Reviewer #1

1. In order to clarify the cytoprotective function of USP8 in the context of TNF-induced complex II formation the authors would need to test the previously described (Jeong et al., Oncogene. 2017 Jan 26;36(4):458-470. doi: 10.1038/onc.2016.215) USP8 mediated regulation of cFLIP-L via its deubiquitylation, thereby protecting it from proteasomal degradation. Furthermore, it would be important to speculate on why the chemical inhibition of USP8 with DC-U4106 proves to be a stronger sensitizer compared to its genetic removal (Fig 1a and 1b).

*> We thank the reviewer for this valuable remark. It is correct that USP8 was previously reported to protect cells from TNF-induced death by promoting cFLIP-L stabilization. Our work does not dispute these earlier findings. Instead, we identify an additional protective function of USP8 in this pathway-namely, the ESCRT-dependent sequestration of cytosolic TNFR1 Complex II into ILVs. In support of this, we show that USP8 inhibition, like TSG101 inactivation, significantly reduces the endosomal localization of cleaved (Cl.) CASP8 puncta (**Fig. 4c-d, Extended Data Fig. 4j**), a readout that is independent of USP8's role in regulating cFLIP.*

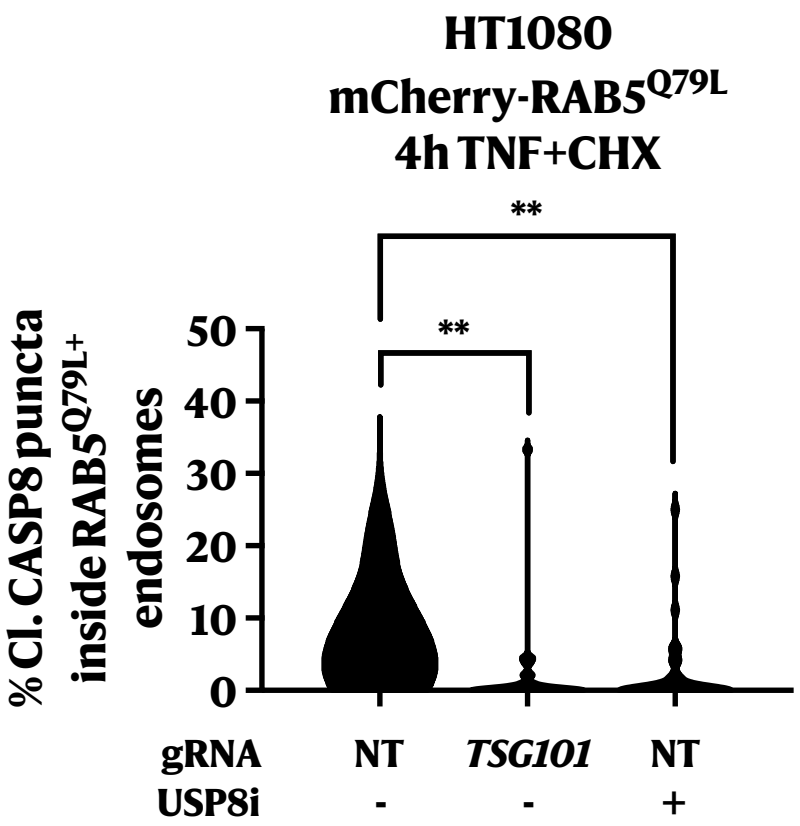

Extended Data Fig. 4d. HT1080 cells were transduced with an inducible expression vector encoding an mCherry-tagged version of the RAB5 Q79L mutant (RAB5^{Q79L}). Transduced cells were electroporated with Cas9-RNPs targeting *TSG101* or a non-targeting sequence (NT). Cells were treated with 2 μg/mL Doxycycline for 48h. Subsequently, cells were pre-treated with CHX and USP8i as indicated before stimulation with 1 μg/mL hTNF for 4h. Quantification of the percentage of Cl. CASP8 found inside mCherry^{+} endosomes was performed. Violin plots represent the distribution of the determined percentage over individual imaged cells. Statistical significance of the microscopy data was determined using a Kruskal-Wallis test followed by a Dunn's multiple comparisons test.

*> We agree, however, that the cytotoxicity assays in **Fig. 1a-b** cannot discriminate between these two protective functions of USP8. To disentangle them in this specific context, we additionally assessed TNF-induced cell death upon USP8 inactivation in the presence of the translation inhibitor cycloheximide (CHX). Since cFLIP is an NF-κB target gene whose cytoprotective effect depends on TNF-induced NF-κB–mediated upregulation, CHX is*

*routinely used to block its synthesis and abolish its inhibitory function on CASP8 activation. Importantly, our results show that USP8 inactivation further sensitizes cells to TNF cytotoxicity even when CHX is present (**Extended Data Fig. 1q, 1y**). These findings therefore suggest a cFLIP-independent protective role of USP8. Notably, the microscopy assay showing USP8-dependent targeting of TNFR1 Complex II into ILVs (**Fig. 4d, Extended Data Fig. 4j**) was also performed in the presence of CHX, further excluding any contribution of cFLIP to this readout. We have clarified this point and included the reference in the text: "A cytoprotective role of USP8 downstream of TNF sensing has previously been attributed to its ability to deubiquitinate cFLIP, thereby preventing its proteasomal degradation[38]. Our data now strongly support an additional function for USP8 in counteracting TNF cytotoxicity that operates independently of NF-κB-mediated cFLIP upregulation." – page 7, lines 7-12.*

ED Fig. 1q

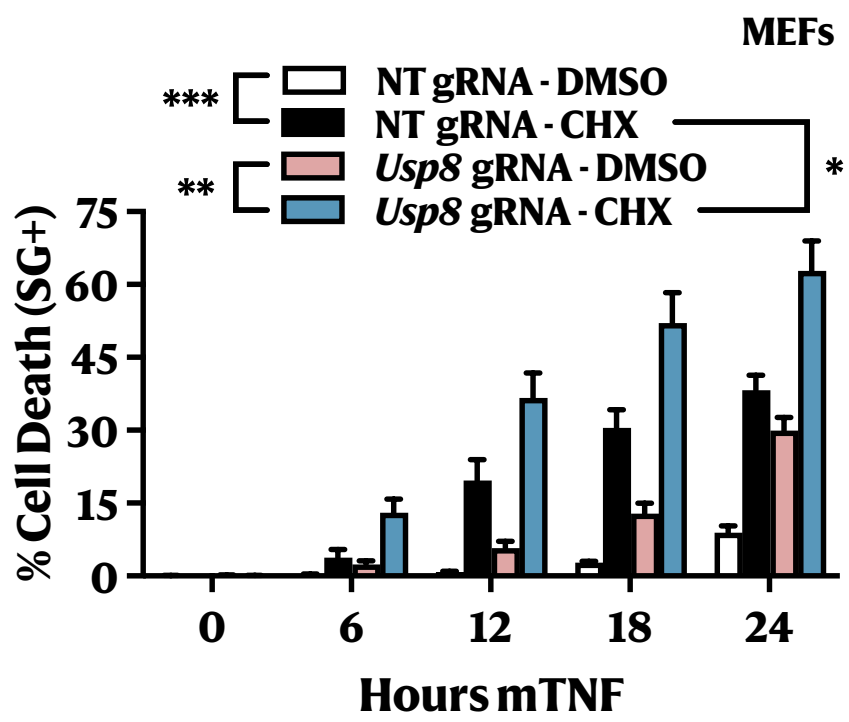

ED Fig. 1y

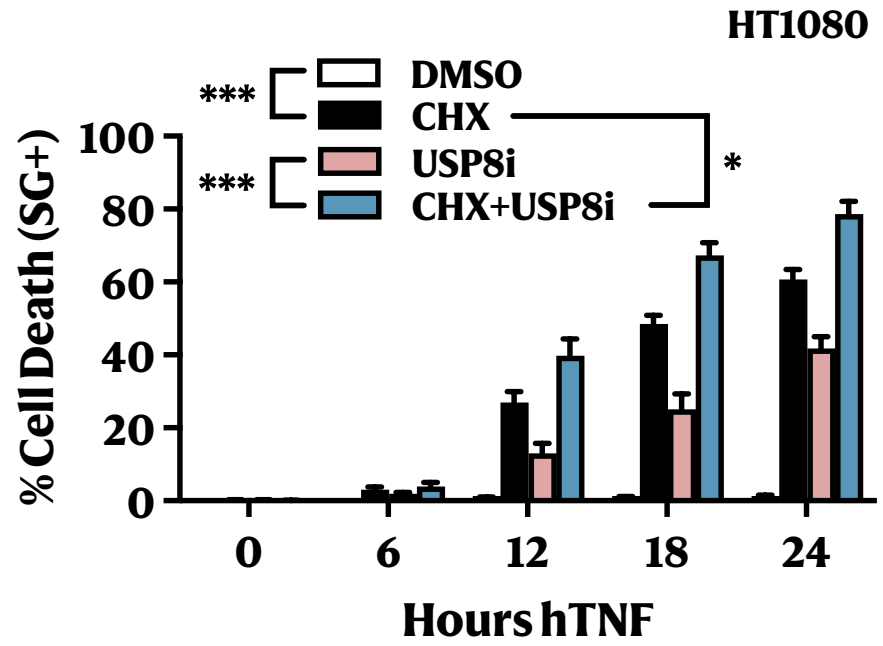

Extended Data (ED) Fig. 1q and 1y. ED Fig. 1q, MEFs electroporated with Cas9-RNPs targeting *Usp8* or NT were pre-treated with CHX as indicated before stimulation with 20 ng/mL mTNF. **ED Fig. 1y**, HT1080 cells were pre-treated with CHX and USP8i as indicated before stimulation with 20 ng/mL hTNF. Cell death was measured over time by SG positivity. Cell death experiments are presented as mean ± SEM of three independent experiments and statistical significance was determined using a two-way ANOVA. Significance between samples is indicated in the figure as follows: *p < 0.05; **p < 0.01; ***p < 0.001; ns non-significant.

> Regarding the stronger sensitizing effect of the USP8 inhibitor DC-U4106 compared to CRISPR-mediated depletion of USP8, we believe this difference arises from the incomplete nature of the genetic knockout at the population level. CRISPR-Cas9 editing is inherently stochastic, and despite enrichment for gene-targeted cells, residual USP8-proficient cells are likely to remain within the population. These cells can partially mask the cytotoxic phenotype, leading to an apparently weaker effect compared to acute pharmacological inhibition, which uniformly suppresses USP8 catalytic activity across the entire culture. We therefore attribute the discrepancy primarily to this technical limitation.

2. The protective role of ESCRT components on TNF-induced necroptosis should be further explored by treatment of the ESCRT-deficient KO lines with TNF together with zVAD. This would be important since the same methodology is used for inhibition of ESCRT-machinery activation with Ca2+ chelator on Extended Data Figure 1k-l.

*> The cytoprotective role of ESCRT components in TNF-induced necroptosis is well established and has been attributed to ESCRT-mediated shedding of damaged plasma membrane regions (Gong et al., Cell 2017). The experiments suggested by the reviewer were already addressed in that study, where ESCRT deficiency was shown to sensitize cells to TNF in the presence of zVAD (Fig. 4, Fig. S5). In our work, we specifically used the Ca^{2+} chelator BAPTA-AM to block this ESCRT-dependent plasma membrane repair function without interfering with the other roles of the ESCRTs. Our data show that BAPTA-AM sensitizes cells to TNF only when combined with zVAD, whereas ESCRT deficiency sensitizes cells even in the absence of caspase inhibition. This distinction demonstrates that ESCRT components also act downstream of TNFR1 activation via a mechanism that is independent of plasma membrane repair. We rephrased this section in the revised manuscript for improved clarity: "We further excluded a contribution of ESCRT-mediated plasma membrane repair by interfering with extracellular calcium influx, which is required to trigger ESCRT recruitment to sites of membrane damage. The entry of extracellular Ca^{2+} through membrane pores was shown to activate the ESCRT-dependent outward budding and shedding of damaged membrane fragments, thereby restoring plasma membrane integrity[32–35]. In line with previous litterature[32], chelation of Ca^{2+} with BAPTA-AM, preventing ESCRT recruitment and activation at the plasma membrane, markedly sensitized cells to TNF-induced necroptosis obtained by pre-treatment with the pan-caspase inhibitor zVAD-fmk (zVAD) (**Extended Data Fig. 1m-n**). However, BAPTA-AM did not mimic ESCRT deficiency in the absence of zVAD, thereby excluding plasma membrane repair as the primary cytoprotective function of the ESCRT machinery downstream of TNF sensing." – page 6, lines 10-21.*

3. The addition of cycloheximide was tested for MEFs lacking different components of the ESCRT machinery and for HT1080 under USP8 inhibition (Fig 1h; Extended Data Fig. 1m-o, w). Here, the authors show bar plots with the direct comparison of ESCRT-proficient and -deficient cells under the challenge of TNF+CHX; however, there is lack of the direct comparison with TNF treatment alone as a control. Only with this comparison it is possible to understand if the pro-survival effects are dependent or not on gene activation. By comparing Fig 1e DMSO and 1h on Tsg101 KO MEFs it appears that the sensitization to TNF-induced cell death is lost; it would be important to speculate on why this is the case as this would further strengthen the claim of a new checkpoint.

*> We agree with the reviewer that a direct comparison with TNF treatment alone is essential for full interpretation. We have consequently included these additional results to the cell death graphs in the revised version of the manuscript (**Fig. 1h**, **Extended Data Fig. 1p-q, y**). The new graphs show that either ESCRT component deficiency or CHX treatment is sufficient to shift the TNF response toward cell death, and that the combination of both conditions results in even greater cytotoxicity than either treatment alone. This demonstrates that the NF-κB checkpoint and the newly discovered ESCRT-dependent checkpoint operate independently of one another. Together, these results strengthen the conclusion that ESCRT-mediated sequestration of TNFR1 Complex II into ILVs constitutes a distinct cell death checkpoint within the TNF pathway.*

Fig. 1h

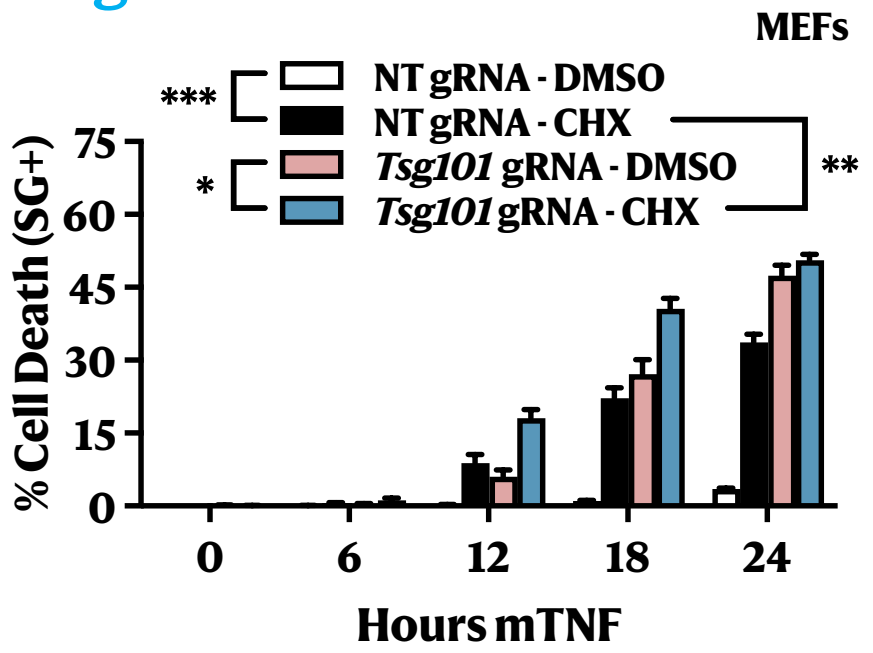

ED Fig. 1p

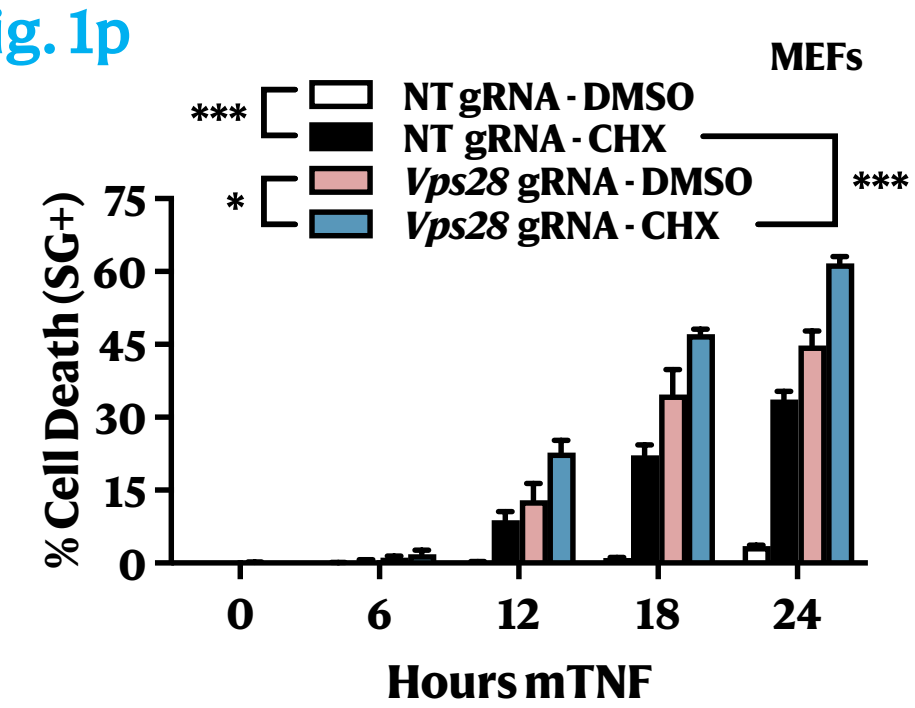

ED Fig. 1q

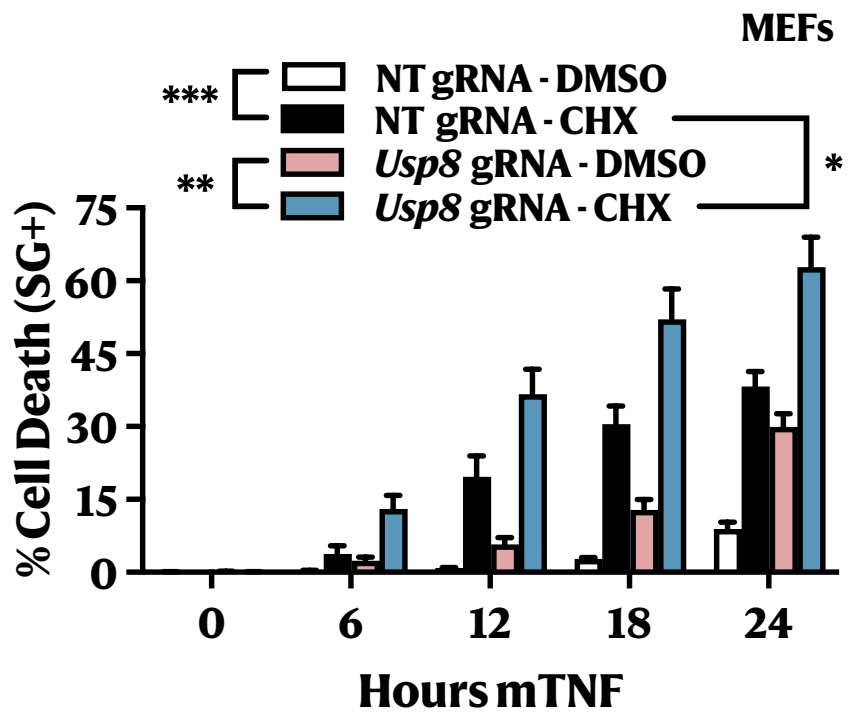

ED Fig. 1y

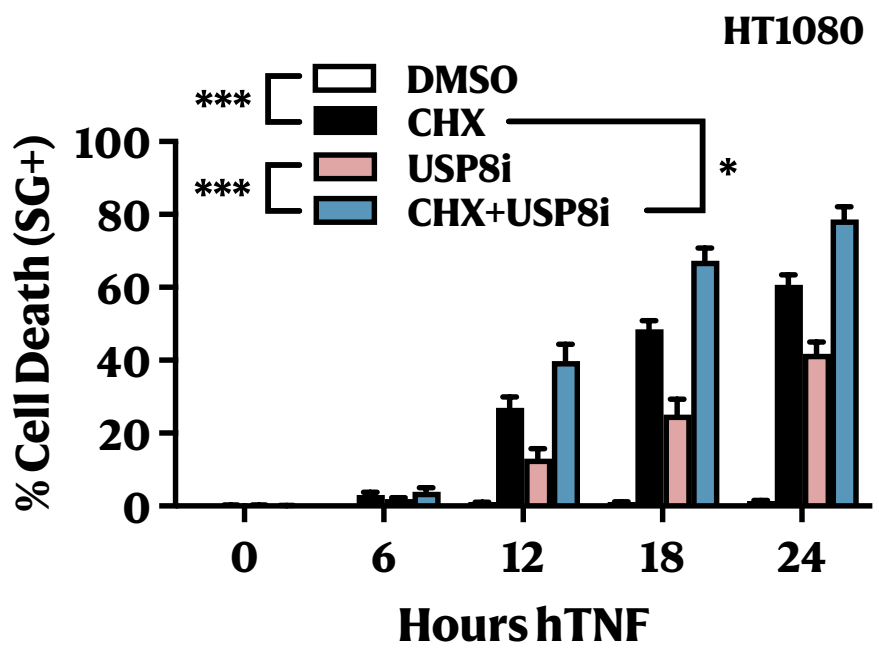

Fig. 1h, Extended Data (ED) Fig. 1p-q, y.and Fig. 2J. **Fig. 1h**, MEFs were transduced with an inducible expression vector encoding Cas9 (hereafter referred to as iCas9 MEFs) and subsequently transduced with inducible expression vectors encoding sgRNAs targeting *Tsg101* or NT. Cells were treated with 1 µg/mL Doxycycline for 48h and subsequently expanded for 72h. Cells were pre-treated with CHX, as indicated, before stimulation with 20 ng/mL mTNF. Cell death was measured over time by SG positivity. **ED Fig. 1p-q**, MEFs were transduced with LentiCRISPRv2 constructs or electroporated with Cas9-RNPs targeting the indicated genes or a non-targeting sequence (NT). Cells were pre-treated with CHX, as indicated, before stimulation with 20 ng/mL mTNF. Cell death was measured over time by SG positivity. **ED Fig. 1y**, HT1080 cells were pre-treated with CHX and USP8i, as indicated, before stimulation with 20 ng/mL hTNF. Cell death was measured over time by SG positivity.

4. In Figure 3 the authors aim to demonstrate the presence of TNF complex II in intralumenal vesicles. The use of immunofluorescence for assessment of localization of cleaved caspase 8 should be complemented with the detection of other components of complex II, furthermore, immunostaining of full-length caspase 8 would be essential to complement Fig3 f-g, where full length caspase 8 is found in extracellular vesicles, both with and without TNF stimulation. Similarly, Caspase 8 immunoprecipitation in Figure 3e should include markers of intralumenal vesicles such as Rab5 to corroborate the findings from the immunofluorescence panels.

*> To complement the extracellular vesicle (EV) data and further strengthen the immunofluorescence-based evidence, we followed the reviewer's suggestion and attempted additional stainings of endogenous FADD and RIPK1 (other components of TNFR1 Complex II). Unfortunately, available commercial antibodies did not yield reliable immunofluorescence signals (Data not shown). To overcome this limitation, we generated MEFs expressing GFP-tagged FADD (Fadd$^{-/-}$ MEFs transduced with a Doxycycline-inducible FADD-GFP construct). Of all prototypical TNFR1 Complex II core components, CASP8 and FADD are the best suited for the specific monitoring of this complex by microscopy as they are absent from TNFR1 Complex I (unlike TRADD and RIPK1). FADD-GFP appeared as a diffuse signal in untreated MEFs, while it formed distinct puncta 4h after TNF stimulation, consistent with TNFR1 Complex II assembly (**Extended Data Fig. 4g**). Importantly, a subset of these puncta colocalized with the early endosome marker EEA1 (**Extended Data Fig. 4h**). These findings therefore support the transfer of an additional component of TNFR1 Complex II into the endosomal system prior to its exosomal release. These additional data have been included in the revised manuscript (**Extended Data Fig. 4g-h**).*

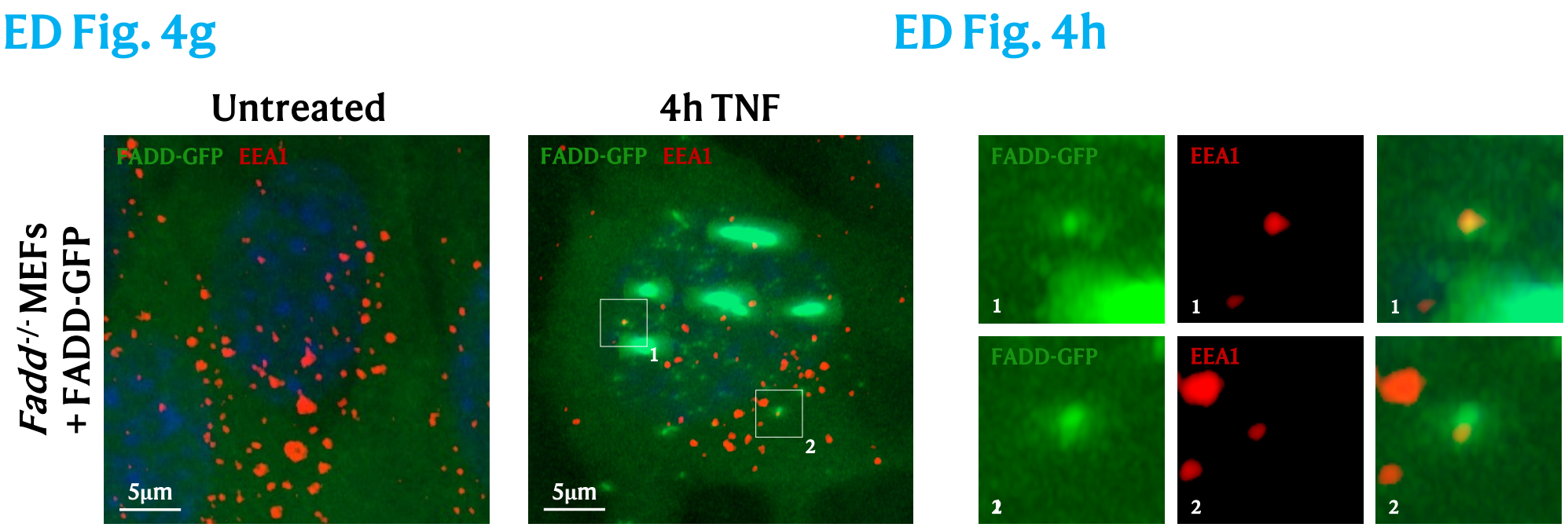

Extended Data (ED) Fig. 4g-h. *Fadd$^{-/-}$* MEFs were reconstituted with an inducible expression vector encoding GFP-tagged FADD. Cells were treated with 100 ng/mL Doxycycline for 24h. Subsequently, cells were stimulated with 20 ng/mL mTNF for 4h. Cells were then stained for the early endosome marker EEA1. **ED Fig. 4g**, Representative images of EEA1 immunostainings are shown. **ED Fig. 4h**, Single and merged channel images of the zoomed subpanels of cells treated with TNF for 4h are shown.

*> Regarding the presence of full-length CASP8 and unbound FADD in EVs isolated from unstimulated cells (**Fig. 4g-h**), we agree that this is an interesting observation and likely reflects a biologically meaningful—though mechanistically distinct—process. However, the focus of the present manuscript is specifically on the regulation and fate of TNFR1 Complex II, which is only detected upon TNF stimulation (**Fig. 4g-h**). A detailed investigation of the mechanisms governing basal incorporation of unbound FADD or CASP8 into EVs would therefore extend beyond the scope of this study.*

> We thank the reviewer for the suggestion to include ILV markers to the CASP8 immunoprecipitations. Unfortunately, spatial relationship between TNFR1 Complex II and ILV markers (such as RAB5 or EEA1) cannot be expected from a co-immunoprecipitation. Indeed, the detergent contained in the lysis buffer disrupts endosomes and their ILVs, releasing TNFR1 Complex II into the cytosolic fraction.

5. Later, the authors explore the fate of the vesicles originated by ESCRT-dependent endosomal micro-autophagy. As mentioned in the discussion "lysosomal degradation is a possible convergence point of both macro-autophagy and eMI", as such, the localization of complex II onto the lysosomal compartment should be evaluated by immunofluorescence by co-localization of cleaved caspase 8 and known lysosomal markers such as LAMP1/LAMP2; this should be performed under Atg9a-proficient and -deficient background. Another possible approach could be the immunoprecipitation of LAMP1 as used in reference 12 of the present manuscript, previous work by the same group.

*> The sequestration of cargo into intralumenal vesicles (ILVs) is a decisive event that can direct cargo toward either lysosomal degradation or extracellular release in exosomes. In the original submission, we provided strong evidence that ILV sequestration of TNFR1 Complex II leads to its extracellular release via exosomes. To address the reviewer's question of whether this process also contributes to lysosomal degradation of TNFR1 Complex II, we followed the suggestion to compare the effects of macro-autophagy versus eMI inhibition on the co-localization of cleaved (Cl.) CASP8 puncta with the lysosome marker LAMP1. These experiments were performed in the presence of the protease inhibitors PepA/E64D to prevent lysosomal degradation and thereby allow accurate assessment of co-localization. Consistent with our previous work demonstrating that LC3-independent macro-autophagy mediates lysosomal degradation of TNFR1 Complex II, we found that ATG9A deficiency significantly reduced the proportion of LAMP1^{+} Cl. CASP8 puncta. In contrast, ESCRT inhibition with the USP8 inhibitor (USP8i) did not alter lysosomal localization of Cl. CASP8, indicating that ESCRT-dependent ILV formation does not contribute to the targeting TNFR1 Complex II to lysosomes (**Fig. 4e, Extended Data Fig. 4k**). Supporting these conclusions, we further observed that blocking all lysosomal fusion events with Bafilomycin A1 (BafA1) markedly reduced the lysosomal localization of Cl. CASP8 puncta, whereas selective inhibition of Late Endosome-lysosome fusion through expression of the dominant-negative RAB7^{T22N} mutant had no such effect (**Fig. 4f, Extended Data Fig. 4l**).*

*> Together, these findings indicate that the ESCRT-dependent sequestration of TNFR1 Complex II into ILVs primarily serves to mediate its extracellular release via exosomes, rather than targeting the complex for lysosomal degradation. These important new findings have been added to and are discussed in the revised manuscript (**Fig. 4e-f** and **Extended Data Fig. 4k-l**). We have additionally adapted the title of the manuscript to better reflect the nature of the newly identified process: "Exosomal Export of TNFR1 Complex II Prevents TNF-Induced Cytotoxicity".*

ED Fig. 4k

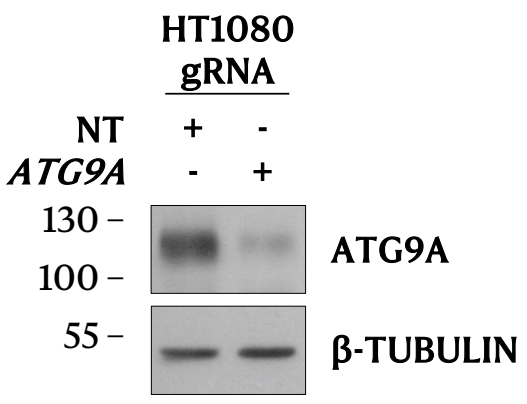

Fig. 4e

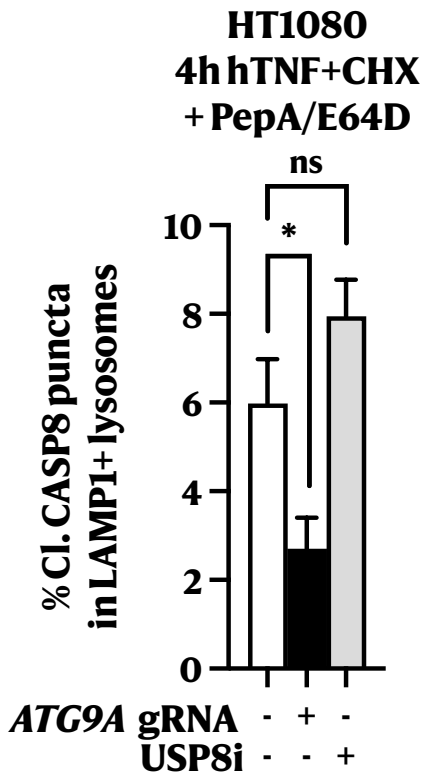

ED Fig. 4l

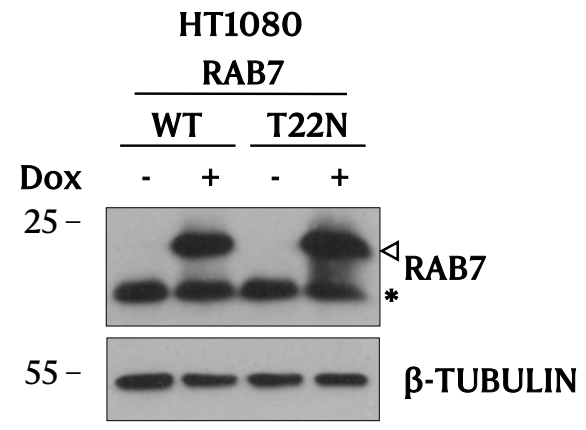

Fig. 4f

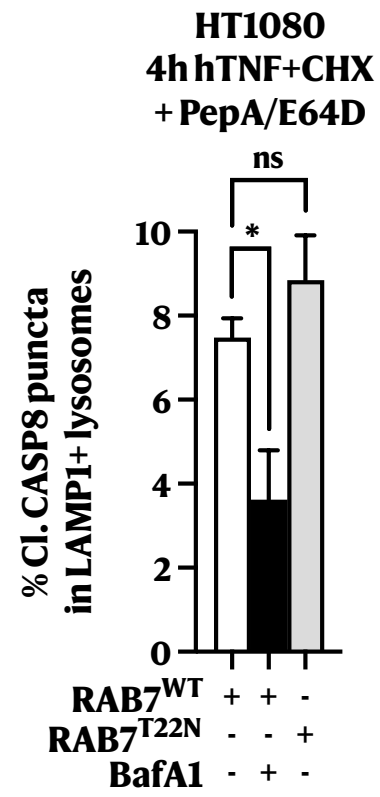

Extended Data (ED) Fig. 4k-l and Fig. 4e-f. ED Fig. 4k and Fig. 4e, HT1080 cells were electroporated with Cas9-RNPs targeting *ATG9A* or a non-targeting sequence (NT). **ED Fig. 4k**, Knockout efficiency was validated by immunoblot. **Fig. 4e**, Cells were pre-treated or not with CHX, USP8i and PepstatinA/E64D, as indicated, before stimulation with 2 µg/mL hTNF for 4h. Cells were then stained for the Cl. CASP8 and the lysosome marker LAMP1. The percentage of Cl. CASP8 puncta that colocalized with $LAMP1^{+}$ lysosomes was quantified and the average across images was determined. **ED Fig. 4l and Fig. 4f**, HT1080 cells were transduced with inducible expression vectors encoding either 3xHA-tagged wild-type (WT) RAB7, or the dominant-negative RAB7 T22N mutants ($RAB7^{T22N}$). Cells were treated with 2 µg/mL Doxycycline for 48h. **ED Fig. 4l**, Protein levels were determined by immunoblot. The endogenously expressed RAB7 and the ectopically expressed 3xHA-tagged RAB7 are indicated by (*) and (◁) respectively. **Fig. 4f**, Cells were pre-treated or not with CHX, BafA1 and PepstatinA/E64D, as indicated, before stimulation with 2 µg/mL hTNF for 4h. Cells were then stained for the Cl. CASP8 and the lysosome marker LAMP1. The percentage of Cl. CASP8 puncta that colocalized with $LAMP1^{+}$ lysosomes was quantified and the average across images was determined. Statistical significance was determined using a one-way ANOVA followed by a Dunn's multiple comparisons test. Significance between samples is indicated in the figure as follows: *$p < 0.05$; **$p < 0.01$; ***$p < 0.001$; ns non-significant.

6. In Figure 4, a model is proposed where M1-Ub RIPK1 is proposed as a scaffold for TAXBP1 promoting its transfer to endosomes. However, in cells that were not challenged with TNF, caspase 8 was found in extracellular vesicles (Figure 3f-g) without the presence of RIPK1, TAXBP1 or M1-ubiquitin. It would be important to understand how this is regulated and if Caspase 8 could be the initial substrate of eMI cargo selection.

*> We agree with the reviewer that the presence of FADD and CASP8 in extracellular vesicles (EVs) from unstimulated cells is intriguing (**Fig. 4g**). However, the fact that these proteins do not co-immunoprecipitate in these conditions suggests that they are not transferred as part of a pre-assembled complex (**Fig. 4h**). They may instead be recruited independently or reflect low-level stochastic sampling of cytosolic proteins.*

> We respectfully disagree with the reviewer's suggestion that CASP8 or FADD could function as the initiating substrate driving selective targeting of TNFR1 Complex II into ILVs. If this were the case, loss of linear ubiquitin chains, RIPK1, or TAX1BP1 should not impede the endosomal targeting of cleaved (Cl.) CASP8 - particularly given that its cytosolic abundance is increased under these conditions. In clear contrast, our results show that deficiency in any of these components markedly impairs the transfer of Cl. CASP8 into endosomes. Furthermore, the requirement for TAX1BP1 is consistent with previous studies demonstrating its role as an autophagy receptor in endosomal micro-autophagy, including the targeting of ferritin (Goodwin et al., Cell Rep. 2017; Ohshima et al., J. Cell Biol. 2022). Together, these findings support a model in which ubiquitin-dependent recognition - rather

than CASP8 or FADD themselves - governs the selective loading of TNFR1 Complex II into ILVs.

> These observations also strongly suggest that the basal transfer of CASP8 and FADD into EVs occurs via a mechanism distinct from the ESCRT-dependent sequestration of TNFR1 Complex II that we describe. As the focus of the present work is specifically on the TNF-induced cytotoxic complex, we believe that dissecting the steady-state release of CASP8 or FADD - while interesting - is beyond the scope of this manuscript.

7. On the contrary, in previous work not mentioned in this manuscript (Wu et al., Cell Res. 2023 Nov;33(11):851-866. doi: 10.1038/s41422-023-00859-3), and supporting a role for RIPK1 as a potential scaffold for eMI initiation, HSC70 has been reported to suppress necroptosis by specifically recognizing RHIM-containing proteins. In this report this is attributed to the ATPase activity of HSC70 but does not exclude the recognition of RIPK1 RHIM domain through a hydrophobic hexapeptide motif. This could be explored by reconstitution of RIPK1 KO MEFs with a RHIM mutant version of RIPK1 or any equivalent strategies.

>We thank the reviewer for this excellent remark. It was indeed recently reported that HSC70 suppresses necroptosis by binding RHIM-containing proteins and by acting as an amyloidase on RHIM oligomers in an ATPase-dependent manner. In contrast, during physiological TNF signaling, RHIM oligomers do not form, and our data indicate that HSC70 acts independently of its ATPase activity. Nevertheless, we agree that HSC70 could, in principle, engage TNFR1 Complex II through a related interaction with the RIPK1 RHIM domain. Wu et al. identified methionine 547 (M547) as a key determinant of the HSC70-RIPK1 interaction, since the RIPK1 M547G mutant showed substantially reduced binding. To assess whether such an interaction contributes to the detoxification of TNFR1 Complex II in our system, we generated the equivalent mutation in mouse RIPK1 (M536G) and reconstituted RIPK1-deficient MEFs with this mutant, as suggested by the reviewer. We then evaluated its effect on TNF-induced cell death. Despite the predicted disruption of HSC70 binding, the RIPK1^{M536G} mutant did not sensitize cells to TNF cytotoxicity, indicating that this interaction is unlikely to be essential for the ESCRT-HSC70-dependent targeting of TNFR1 Complex II. These new results have been added to and are discussed in the revised manuscript (Extended Data Fig. 5m-n).

ED Fig. 5m **ED Fig. 5n**

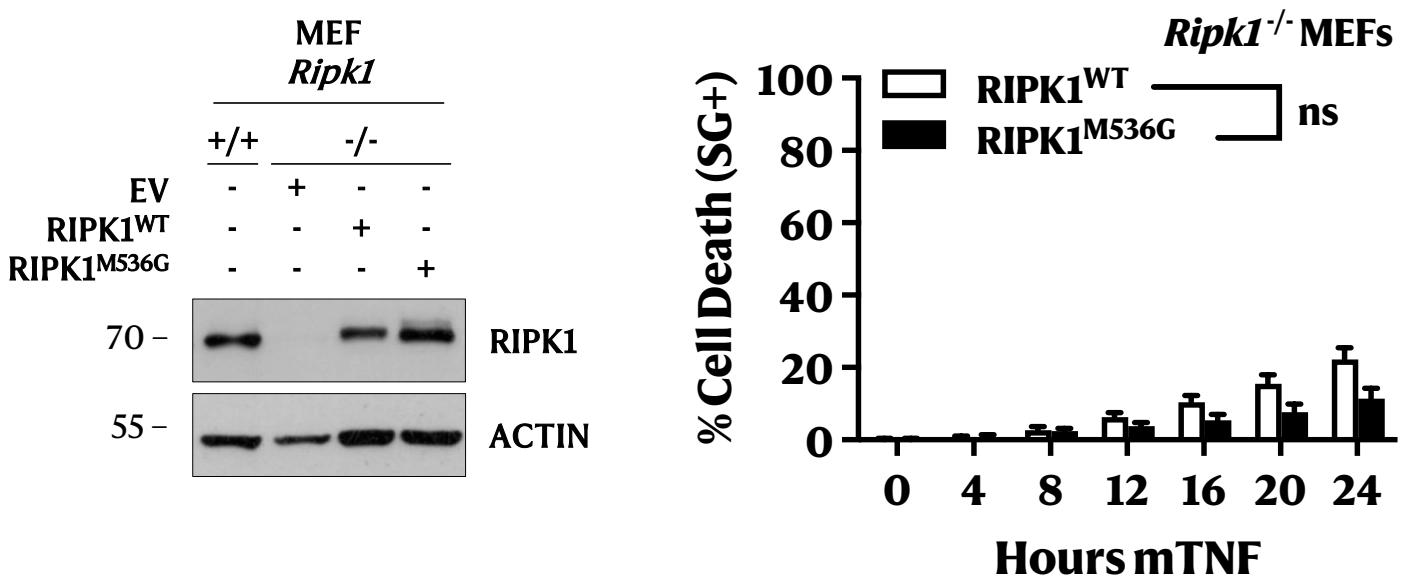

Extended Data Fig. 5m-n. *Ripk1*$^{-/-}$ MEFs were reconstituted with inducible expression vector encoding either wild-type RIPK1 (RIPK1^{WT}), the M536G RIPK1 mutant (RIPK1^{M536G}) or an empty vector (EV). Reconstituted cells were treated with 100 ng/mL Doxycycline for 24h. **ED Fig. 5m**, Reconstitution was validated and compared to *Ripk1*$^{+/+}$ MEFs via immunoblotting. **ED Fig. 5n**, Cells were treated with 20 ng/mL mTNF and cell death was measured over time by SytoxGreen (SG) positivity. Cell death experiments are presented as mean ± SEM of three independent experiments and statistical

significance was determined using a two-way ANOVA. Significance between samples is indicated in the figure as follows: $^{*}p < 0.05$; $^{**}p < 0.01$; $^{***}p < 0.001$; ns non-significant.

Reviewer #2

1. Since the effort is on EV formation upon MVB formation, it has to be questioned why the process is called Microautophagy. Simple 'export' rather than degradation. Thus, unless degradation is shown well, this is not eMI in the strict sense. It is not addressed here if true eMI actually also contributes to desensitization: is the complex stabilized in purified late endosomes upon inhibition of proteases? Perhaps in conjunction with prevention of exosome secretion? This would be required for the exact mechanism, although the authors show that protection from apoptosis is already achieved by sequestration of complex II from the cytoplasm.

> As a similar remark was raised by Reviewer #1, we invite Reviewer #2 to go through the answer provided to point 5 of Reviewer #1.

> Our new results indicate that ESCRT-dependent sequestration of TNFR1 Complex II into ILVs primarily serves to mediate its extracellular release via exosomes, rather than targeting the complex for lysosomal degradation. As suggested by the reviewer, we have adapted the title of the revised manuscript to better reflect the nature of the newly identified process: "Exosomal Export of TNFR1 Complex II Prevents TNF-Induced Cytotoxicity".

2. P8 Line 18 statement about effects or Rab variants on eMI degradation: There is a fast eMI that is starvation inducible and a slower one that is not. In addition to lacking a reference, it needs to be made sure that this statement is true in general.

> We thank the reviewer for this insightful comment. Indeed, both a constitutive form of eMI and a starvation-induced, accelerated form of eMI have been described (Mejlvang et al., J. Cell Biol., 2018; Olsvik et al., Autophagy, 2019). However, we can confidently exclude involvement of a starvation-induced pathway in our system, as all experiments in the manuscript were conducted under nutrient-replete conditions, and no starvation cues were applied. Moreover, the starvation-induced eMI pathway has been reported to operate independently of ESCRT-I components (TSG101, VPS28) and HSC70 - whereas our results clearly show that these factors are essential for targeting TNFR1 Complex II into endosomes. This argues strongly against participation of the starvation-responsive branch of eMI.

> We agree with the reviewer that the original manuscript did not directly demonstrate that the dominant-negative RAB mutants interfere with eMI. In response, we attempted to monitor two established eMI read-outs - the turnover of ferritin (induced by DFO) and of autophagy receptors (induced by starvation) - in ATG9A-deficient MEFs to eliminate macro-autophagy. However, unlike previous reports, we were unable to detect robust eMI-dependent turnover of these cargoes (Data not shown), preventing us from using these assays to assess the impact of the RAB mutants. Due to time and experimental limitations, we therefore cannot directly confirm whether these mutants block eMI in our hands. Nonetheless, the dominant-negative RAB variants used here are extensively characterized in the literature and routinely applied to disrupt specific steps of the endosomal pathway that are essential for eMI - namely ILV formation ($RAB5^{S34N}$), MVB maturation ($RAB5^{Q79L}$), and lysosomal fusion ($RAB7^{T22N}$). To avoid overstating their specificity, we have carefully rephrased the relevant sentence in the revised manuscript to reflect this nuance: "Thus, if the ESCRT-dependent formation of ILVs at the endosomal membrane is required to prevent TNF cytotoxicity, only $RAB5^{S34N}$ is expected to switch the TNF response to death, as the formation

*of ILVs is unaffected in the other RAB mutants (**Fig. 2a**). Accordingly, we found that while ectopic expression of RAB5^{Q79L} and RAB7^{T22N} had no impact on TNF cytotoxicity, cells expressing RAB5^{S34N} died in response to TNF sensing (**Fig. 2b-c** and **Extended Data Fig. 2a**).”- Page 8, lines 14-19.*

> Importantly, new data added to the revised manuscript demonstrate that the ESCRT-dependent sequestration of TNFR1 Complex II into ILVs primarily mediates its extracellular release rather than its lysosomal degradation. Consequently, this process no longer qualifies as eMI in the strict sense. In light of this, we hope the reviewer agrees that assessing the eMI-dependent turnover of canonical cargoes using the dominant-negative RAB mutants is therefore no longer essential for the interpretation of our study.

3. P9 Line 10…: the vps34 inhibitor argument about MA not being involved (Fig. 2K, not 1K) makes only sense if it shown that RabS34N itself does not affect MA (as it messes up the endosomal system).

*> We agree with the reviewer’s remark. To formally test this, we monitored LC3 lipidation under both nutrient-replete and starvation conditions and found that expression of RAB5^{S34N} did not impair macro-autophagy. These new results demonstrate that the sensitizing effect of RAB5^{S34N} is independent of macro-autophagy and is instead attributable to its inhibition of ILV formation. We have added these new data to the revised manuscript (**Extended Data Fig. 2c-e**).*

ED Fig. 2c **ED Fig. 2d** **ED Fig. 2e**

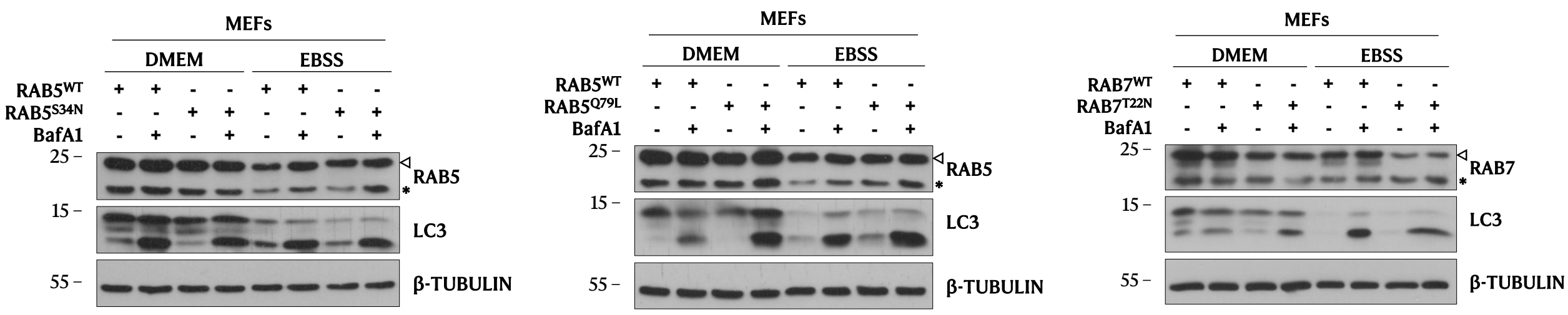

Extended Data (ED) Fig. 2c-e. MEFs were transduced with inducible expression vectors encoding either 3xHA-tagged wild-type (WT) RAB5 or RAB7, or the dominant-negative mutants RAB5^{S34N}(**ED Fig. 2c**), RAB5^{Q79L}(**ED Fig. 2d**), and RAB7^{T22N}(**ED Fig. 2e**). Cells were treated, or not, with 2 µg/mL Doxycycline (Dox) for 48h. Cells were subsequently treated with EBSS for 4h in the presence or absence of BafA1. Protein levels were determined by immunoblot.

4. P14 Line 22…: While it is clear that the bacterial toxins that inhibit ESCRT components sensitize for death, the interpretation that this effect is lost upon treatment with the ESCRT inhibitor USP8i is flawed and needs to be revised: both show the same effect that at one point is saturated. A geneticist would argue that the factors act in the same pathway. More telling would be a test for synergy using low doses of the inhibitor with sub-effect expression level of the toxins.

*>We agree with the reviewer that the interpretation of these results was not correctly phrased in this sentence. We have therefore rephrased it to increase clarity: “This notion was further supported by the fact that SopB and EsxG-H have no additional sensitizing effect when the ESCRT machinery is already inhibited by pre-treatment with USP8i (**Fig. 6e** and **Extended Data Fig. 6h**).”- Page 17, lines 29-31.*

5. Discussion why ESCRT II lack has no effect: one of the proposed reasons (redundancy) is testable. ESCRT II is used by all forms of eMI described in mammals and flies to the best knowledge of this reviewer.

>We thank the reviewer for raising this important point. As suggested, we considered the possibility that the absence of a phenotype in our ALIX- or ESCRT-II–deficient cells could reflect functional redundancy between these components. To directly address this, we generated cells lacking both ALIX and the ESCRT-II subunit VPS36. When challenged with TNF, these double-knockout cells did not exhibit increased cytotoxicity compared with wild-type or single-knockout controls. These results formally exclude a requirement for ALIX or ESCRT-II in directing TNFR1 Complex II into ILVs. These new data have been incorporated into the revised manuscript (Extended Data Fig. 1b-c).

ED Fig. 1b

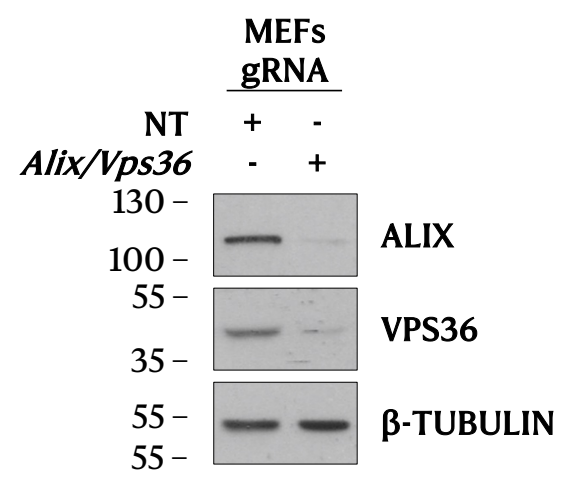

ED Fig. 1c

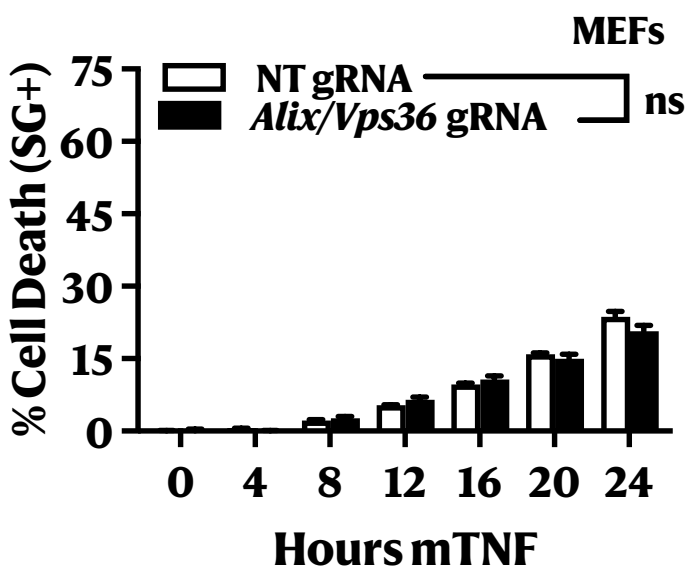

Extended Data (ED) Fig. 1b-c. MEFs were electroporated with Cas9-RNPs targeting *Alix* and *Vps36* or a non-targeting sequence (NT). **ED Fig. 1b**, Knockout efficiency was validated by immunoblot. **ED Fig. 1c**, Cells were stimulated with 20 ng/mL mTNF and cell death was measured over time by SytoxGreen (SG) positivity. Cell death experiments are presented as mean ± SEM of three independent experiments and statistical significance was determined using a two-way ANOVA, ns = non-significant.

> In line with our findings, a study published after our initial submission (Men et al., J Cell Biol., 2025) reported that tau aggregates are targeted into ILVs independently of ESCRT-II. Instead, PTPN23 was identified as the essential factor bridging ESCRT-I and ESCRT-III. These findings are fully consistent with our results and supports a model in which PTPN23, rather than ESCRT-II or ALIX, mediates TNFR1 Complex II sequestration during ESCRT-dependent detoxification. We have adapted our discussion to include a citation to this recent study.

6. Minor points:

- **Fig 1F: no lambda PPase in figure (but in legend). It would also be helpful to systematically mention the cells used (e.g. MEFs or not e.g. in Fig 1G; Fig S3 H, I).**
- **Fig S3 C: resolution not good enough to see ILVs in what is shown as MVBs.**
- **Endosomal localization of components: as these are enlarged MVBs this should be noted and they should be called something along 'Rab5S34N-endosomes'.**

> We thank the reviewer for these constructive remarks. We have adapted the manuscript accordingly.

Reviewer #3

1. It is not yet clear whether the observed effects on TNF-ICD are directly due to EV-mediated detoxification of Complex II, or if they may instead result from alterations in TNFR1 trafficking, which could independently influence receptor signaling and cell death sensitivity. TNFR1 trafficking must be assessed: Given that many of the proteins under study (HSC70, ESCRT components, RAB5, microbial effectors) are involved in receptor trafficking, TNFR1 surface expression should be analyzed by flow cytometry across all relevant cell lines (parental, CRISPR/CAS9 KD/KO, and overexpression models). This is critical to rule out receptor mislocalization as a confounding factor. Figure 2g shows increased Complex I–bound TNFR1 in RAB5-S34N cells. This supports the above concern. Please provide TNFR1 surface levels in all experimental conditions.

> We thank the reviewer for this important remark. TNFR1 has indeed been reported to engage the endolysosomal pathway, and its trafficking is therefore likely affected under several of the conditions we examined. To distinguish defective ILV-mediated detoxification of TNFR1 Complex II from altered TNFR1 trafficking as the cause of the observed TNF cytotoxicity, we directly tested whether blocking TNFR1 trafficking impacts cell viability. First, we verified that TNFR1 is normally targeted for lysosomal degradation by comparing its protein levels in the presence or absence of the lysosomal fusion inhibitor Bafilomycin A1 (BafA1) (Fig. 3c). Notably, preventing lysosomal delivery of TNFR1 with BafA1 did not affect cell viability (Fig. 3d). We next interfered with earlier steps of TNFR1 transport through the endosomal system. Inhibition of TNFR1 sorting into ILVs through loss of the ESCRT-II subunit VPS36—whose absence does not impair TNFR1 Complex II targeting—stabilized TNFR1 (Fig. 3e) but again failed to induce TNF cytotoxicity (Fig. 3f). Finally, blocking DYNAMIN-1–mediated endocytosis of TNFR1 using the pharmacological inhibitor Dyngo-4a similarly prevented receptor degradation without triggering cell death (Fig. 3g-h). Consistent results were obtained using the dominant-negative $DYN1^{S45N}$ mutant or the dominant-negative AP180 C-terminal fragment (Extended Data Fig. 3c-d). Together, these results demonstrate that interfering with TNFR1 trafficking at multiple stages of the endolysosomal pathway does not induce cell death.

Fig. 3c

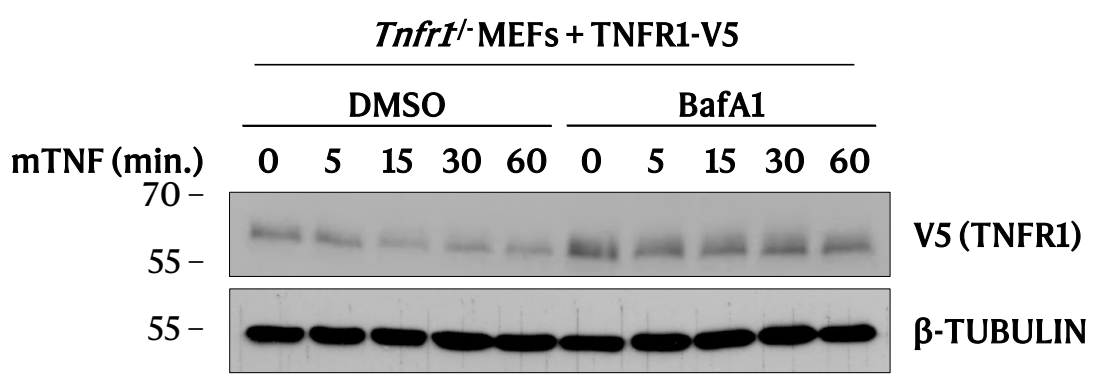

Fig. 3d

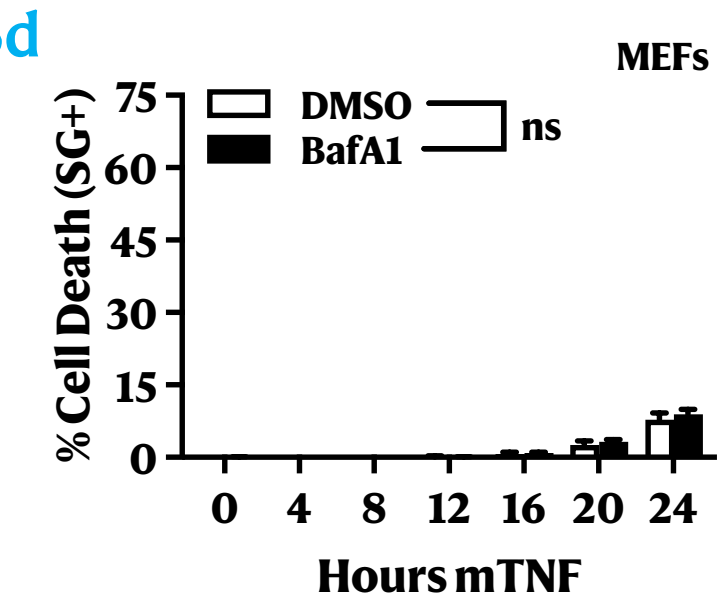

Fig. 3e

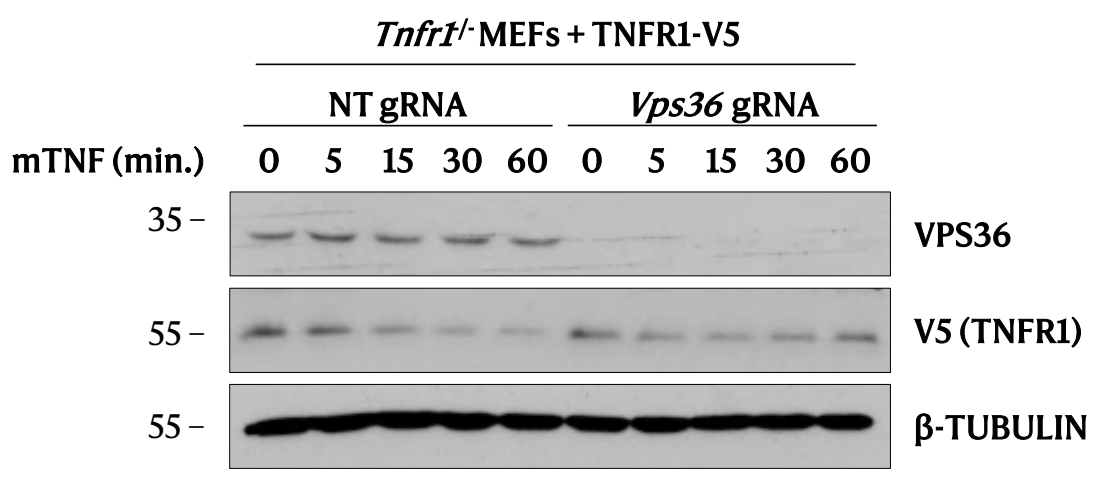

Fig. 3 f

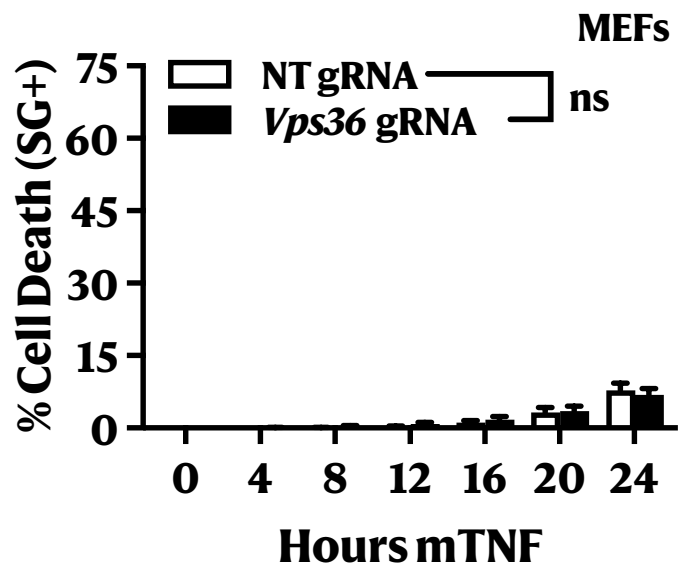

Fig. 3g

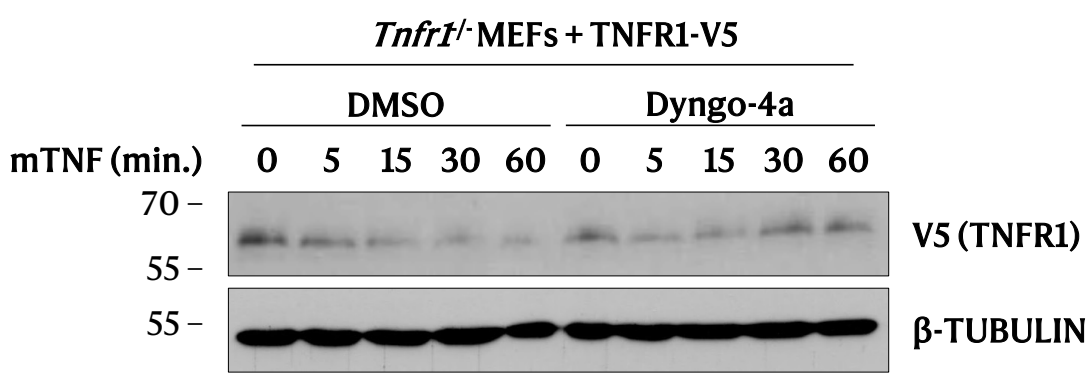

Fig. 3h

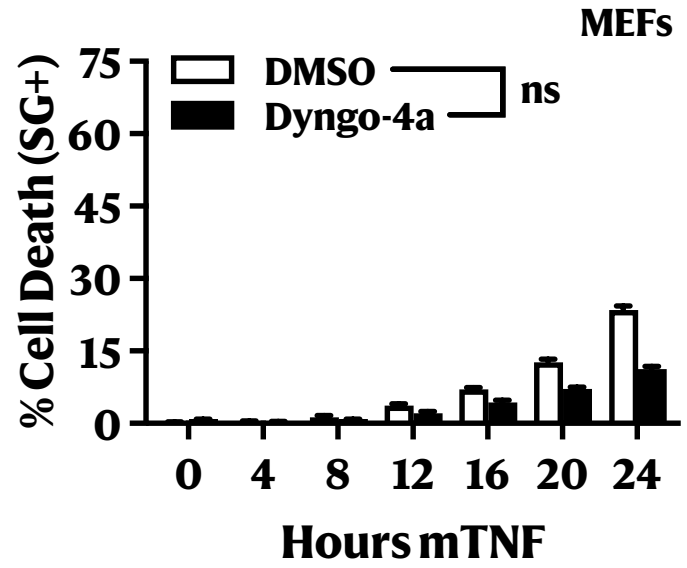

Fig. 3c-h. *Tnfr1* -/- MEFs were transduced with an inducible expression vector encoding V5-tagged TNFR1. Cells were either pre-treated with BafA1 (**Fig. 3c-d**), Dyngo-4a (**Fig. 3g-h**) or electroporated with Cas9-RNPs targeting *Vps36* or NT (**Fig. 3e-f**). Cells were then stimulated with 20 ng/mL mTNF for the indicated duration. Protein levels were determined by immunoblot (**c**, **e**, **g**) and cell death induction was measured over time by SytoxGreen (SG) positivity (**Fig. 3d**, **f**, **h**). Cell death experiments are presented as mean ± SEM of three independent experiments and statistical significance was determined using a two-way ANOVA.

ED Fig. 3c

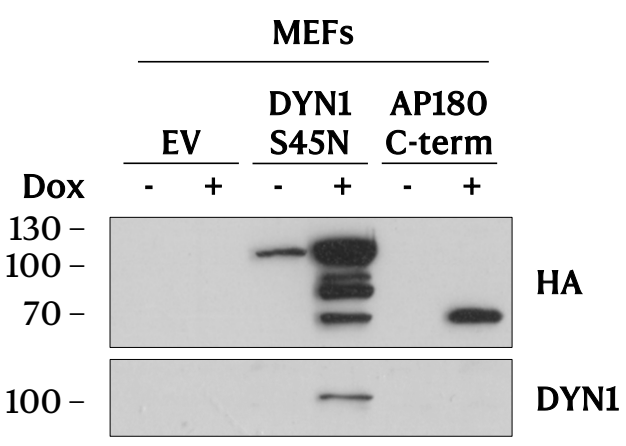

ED Fig. 3d

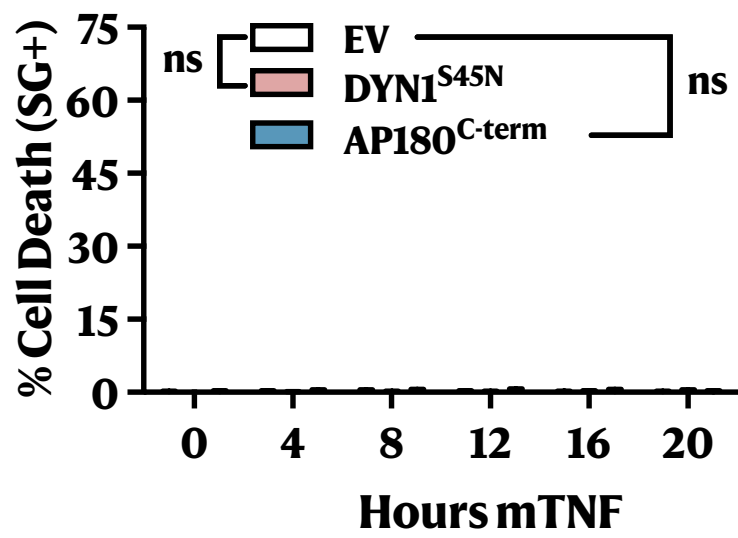

Extended Data (ED) Fig. 3c-d. MEFs were transduced with inducible expression vectors encoding HA-tagged versions of the dominant negative DYNAMIN-1 S45N mutant (DYN1^{S45N}), the dominant-negative C-terminal fragment of AP180 (AP180$^{C\text{-term}}$) or an empty vector (EV). **ED Fig.3c-d**, Cells were treated, or not, with 1 µg/mL Doxycycline (Dox) for 24h and protein levels were determined by immunoblot (**ED Fig. 3c**). Additionally, the cells were stimulated with 20ng/mL mTNF and cell death was measured over time by SytoxGreen (SG) positivity (**ED Fig. 3d**). Cell death experiments are presented as mean ± SEM of three independent experiments and statistical significance was determined using a two-way ANOVA.

> To further demonstrate that the cytoprotective effect of the ESCRTs is independent from the ESCRT-dependent sorting of TNFR1 into ILVs, we tested the effect of ESCRT inhibition by USP8i in cells in which TNFR1 internalization is blocked by Dyngo-4a. Remarkably, USP8i treatment still sensitized the cells to TNF-mediated apoptosis in these conditions (Fig. 3i-j).

Fig. 3i

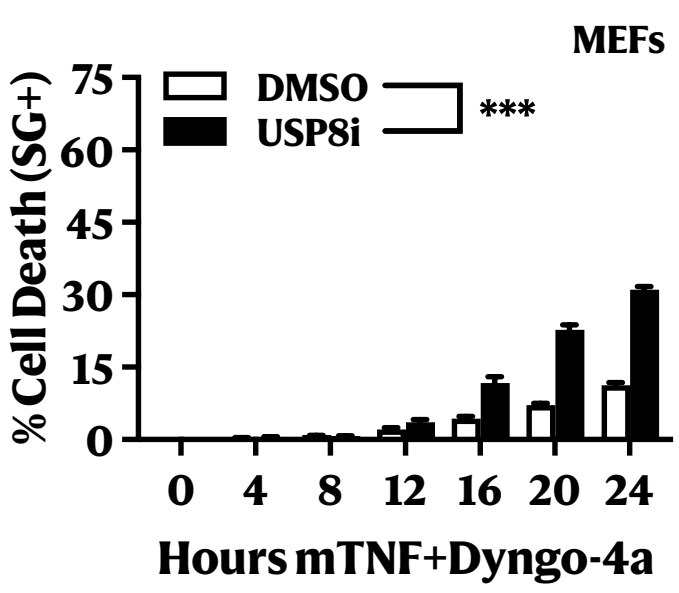

Fig. 3j

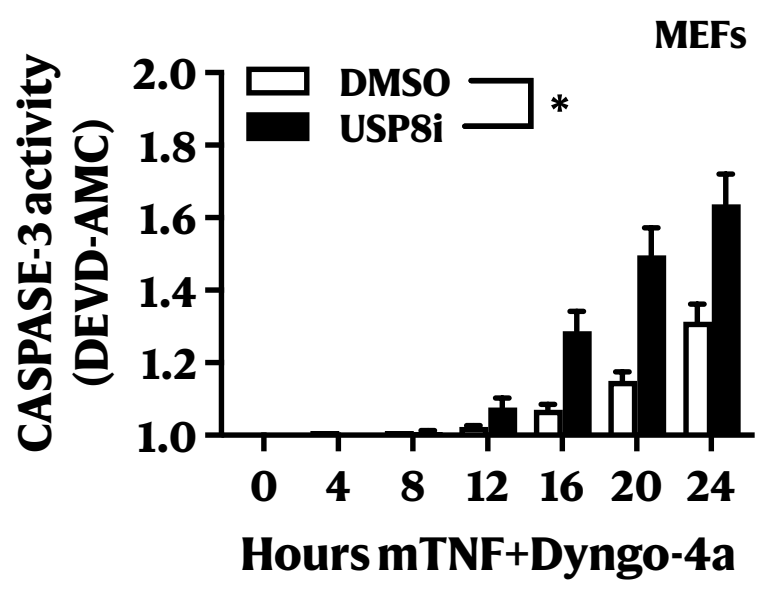

*Fig. 3i-j. MEFs were pre-treated with Dyngo-4a and USP8i, as indicated, prior to stimulation with 20 ng/mL mTNF. **Fig. 3i**, cell death was measured over time by SytoxGreen (SG) positivity. **Fig.3j**, Extracellular caspase activity was quantified over time using a fluorescent caspase-activity probe (DEVD-AMC). Cell death and CASPASE-3 activity experiments are presented as mean ± SEM of three independent experiments and statistical significance was determined using a two-way ANOVA.*

*> Together, these results demonstrate that the formation and cytotoxic potential of TNFR1 Complex II are independent of TNFR1 trafficking. The phenotypes observed in the manuscript cannot be attributed to receptor mislocalization but instead support the model in which the ESCRT machinery prevents TNF cytotoxicity by mediating the ILV-dependent sequestration and ultimately extracellular release- of TNFR1 Complex II. These new data have been incorporated into an entirely new figure in the revised manuscript dedicated to dissecting TNFR1 trafficking and its relationship to TNF-induced cell death (**Fig. 3** and **Extended Data Fig. 3**).*

> Given these findings, we believe that measuring TNFR1 surface expression by flow cytometry, as suggested by the reviewer, would not meaningfully contribute to the interpretation of our data. Moreover, ESCRT deficiency, RAB5^{S34N} expression, or microbial effector proteins do not impair internalization; they act downstream, by preventing ILV formation and thereby trapping receptors within endosomal compartments—not at the plasma membrane.

2. Clarify experimental design across conditions: There is inconsistency in the use of TNF Alone vs TNF + CHX, in WT or ATG9a−/− cells. A systematic side-by-side comparison of TNF-ICD in WT vs ATG9a−/− cells in the presence or absence of CHX would greatly improve interpretability and readability.

> We thank the reviewer for this helpful suggestion. To improve clarity, we have updated the labeling of all relevant figure panels to more clearly indicate the specific treatment conditions (TNF alone vs. TNF + CHX) and the corresponding genotypes (WT vs. ATG9A-deficient cells). This should enhance interpretability and ensure consistent comparison across experiments. Regarding a systematic side-by-side analysis of TNF-induced cell death in WT and ATG9A-deficient cells with or without CHX, we would like to refer the reviewer to our previous work (Huyghe et al., Science 2022, Fig. 1H), in which this comparison was performed.

3. Validate the nature of EVs: To confirm that the analyzed vesicles are not apoptotic bodies: Please include experiments using caspase inhibitors (e.g., ZVAD or QVD). Exclude Annexin V+ vesicles during EV isolation.

*> We agree with the reviewer that it is essential to exclude the possibility that the TNFR1 Complex II components detected in EVs originate from apoptotic bodies released by dying cells. Several lines of evidence in the original submission already argue strongly against this interpretation. First, HT1080 cells do not undergo apoptosis in response to the TNF concentration used in this study, as previously shown (Huyghe et al., Science 2022, Fig. S5C) and confirmed again in the revised manuscript (**Extended Data Fig. 4r**). Notably, this remains true at the 6-hour time point used for EV isolation. Second, apoptotic bodies are substantially larger than exosomes; however, nanoparticle tracking analysis (NTA) revealed no increase in EV particle size following TNF stimulation (**Extended Data Fig. 4x**). Third, ultrastructural analysis by electron microscopy showed no appearance of apoptotic bodies or morphological alterations in EV preparations from TNF-treated cells (**Extended Data Fig. 3w**). To address the reviewer's concern even more directly, we generated CASP3/7-deficient HT1080 cells, which are completely resistant to TNF-induced apoptosis (**Extended Data Fig. 4t**) and therefore incapable of forming apoptotic bodies. EVs isolated from these apoptosis-*

resistant cells after TNF treatment still contained activated (Cl.) CASP8 (Extended Data Fig. 4u), definitively ruling out contamination by apoptotic bodies as the source of TNFR1 Complex II components. These new results have been added to the revised manuscript.

ED Fig. 4t

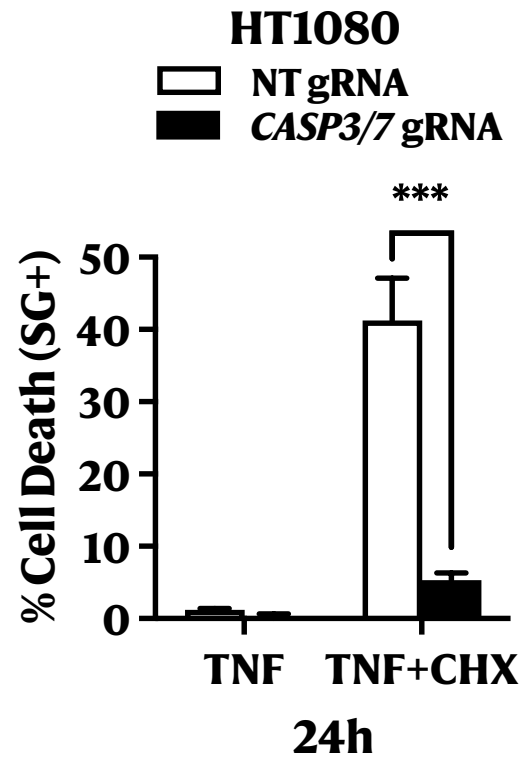

ED Fig. 4u

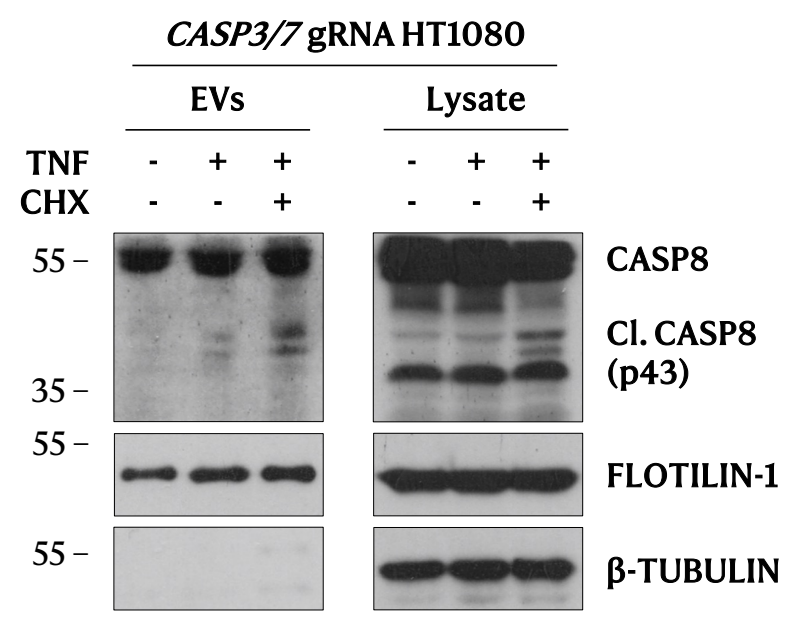

Extended Data (ED) Fig. 4t-u. ED Fig. 4t, HT1080 cells were electroporated with Cas9-RNPs targeting *CASP3* and *CASP7* or a non-targeting sequence (NT). Cells were pre-treated with CHX, as indicated, prior to stimulation with 20 ng/mL hTNF. Cell death was measured after 24h by SytoxGreen (SG) positivity.**ED Fig. 4u**, Cells were pre-treated with CHX, as indicated, prior to stimulation with 2 μg/mL hTNF for 6h. Cells were lysed and extracellular vesicles (EVs) were isolated from the conditioned medium using size exclusion chromatography (SEC) (Fractions 4-6). Protein levels were determined by immunoblot. Cell death experiments are presented as mean ± SEM of three independent experiments and statistical significance was determined using a two-way ANOVA or multiple unpaired t-tests.

4. EV profiling should be expanded to include the following TNFR1 CII components TRAF2, TRADD, c-FLIP, cIAP1, TRAF1, and compared to EVs obtained from cells stimulated with FasL and TRAIL to assess whether this detoxification process is TNF-specific.

*> We thank the reviewer for this suggestion. Regarding TRADD, the reviewer may have overlooked that its presence in EVs was already shown in the original manuscript (**Extended Data Fig. 4p**). Regarding the additional components proposed by the reviewer, many of these - such as TRAF2, cIAP1 and TRAF1 - are primarily associated with TNFR1 Complex I rather than being bona fide components of TNFR1 Complex II. In contrast, our profiling already demonstrates the presence of multiple well-established TNFR1 Complex II components (RIPK1, M1-Ub, TRADD, FADD, Cl. CASP8, TAX1BP1), providing strong evidence that the EVs indeed contain TNFR1 Complex II. Nevertheless, to address the reviewer's concern, we performed additional SEC-based EV isolations and examined the suggested proteins. While all factors were readily detectable in cell lysates, only TRAF2 exhibited a clear TNF-dependent enrichment in EVs. These new results have been incorporated into the revised manuscript (Extended Data Fig. 4q).*

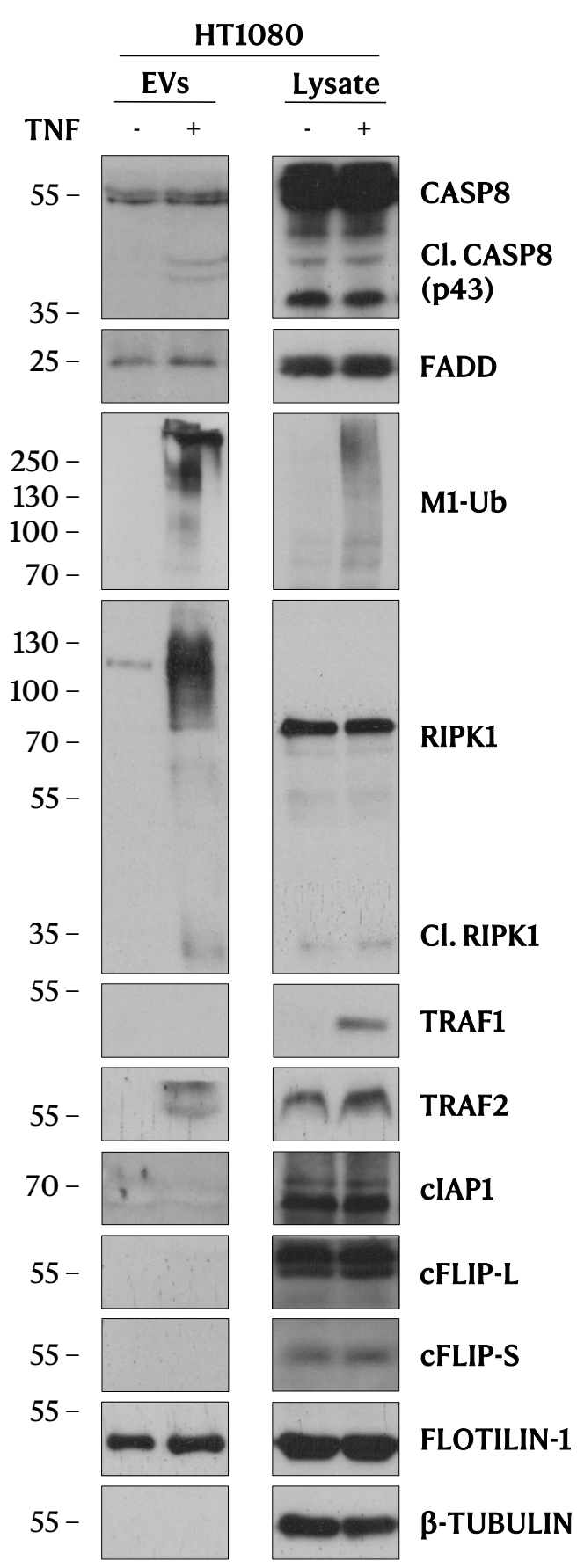

Extended Data Fig. 4q. HT1080 cells were stimulated, or not, with 2 μg/mL hTNF for 6h. Cells were lysed and extracellular vesicles (EVs) were isolated from the conditioned medium using SEC (Fractions 4-6). Protein levels were determined by immunoblot.

> We do not believe that comparing EV cargoes from TNF-, FasL-, or TRAIL-treated cells would yield mechanistically meaningful insight. It is well established that TNFR1 Complex II is a cytosolic complex that forms after dissociation of TNFR1 Complex I components from the receptor and their subsequent association with FADD and CASP8 (Micheau and Tschopp, Cell 2003). This distinguishes TNF signaling from FasL and TRAIL, whose Death-Inducing Signaling Complexes (DISCs) remain receptor-bound. The molecular mechanism that brings TNFR1 Complex II into EVs is therefore intrinsically different from the one that would bring those DISCs into EVs. To exclude the possibility that a DISC-like complex could eventually form at TNFR1 when ILV formation is blocked, we performed additional TNFR1 Complex I immunoprecipitations in Tsg101-deficient MEFs. Even at time points where activated (Cl.) CASP8 is readily detectable in the cytosol, we did not detect CASP8 or Cl. CASP8 in TNFR1 Complex I (Fig. 3b), demonstrating that DISC formation does not occur at the level of TNFR1 even when endolysosomal trafficking is impaired. For these reasons, we consider cross-comparison with FasL- or TRAIL-derived EVs not informative for the present study.

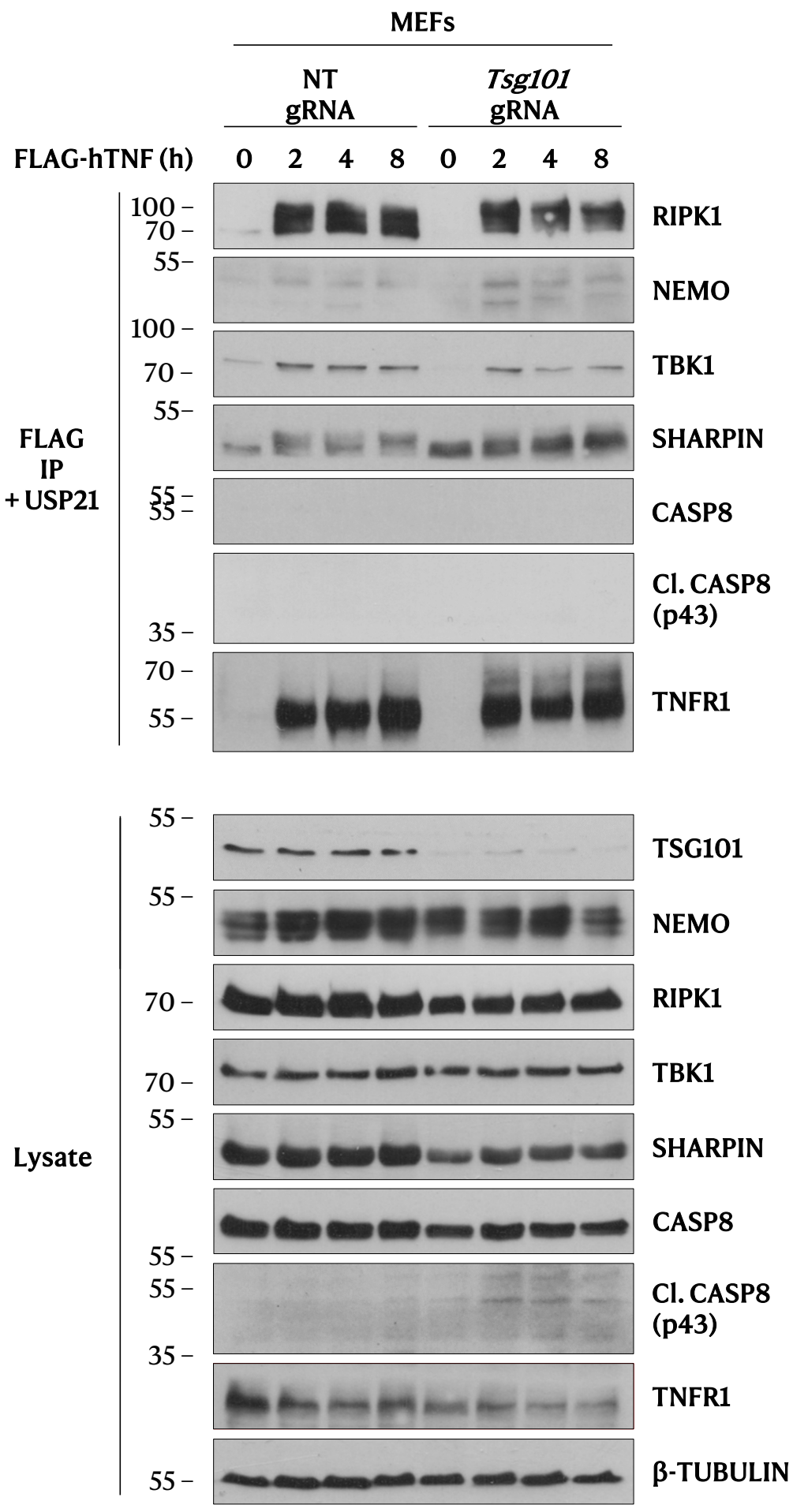

Fig. 3b. MEFs were transduced with an inducible expression vector encoding Cas9 (hereafter referred to as iCas9 MEFs) and subsequently transduced with inducible expression vectors encoding sgRNAs targeting *Tsg101* or a non-targeting sequence (NT). Cells were treated with 1 μg/mL Doxycycline for 48h and subsequently expanded for 72h. Cells were stimulated with 1 μg/mL FLAG-hTNF for the indicated duration. TNFR1 Complex I was FLAG-immunoprecipitated, followed by USP21 treatment. Protein levels were determined by immunoblot.

5. Broaden your discussion: Extend discussion to other ligands of the family ie : FasL and TRAIL ie the following published work (See https://www.cell.com/cell/abstract/S0092-8674(25)00728-7 and https://pubmed.ncbi.nlm.nih.gov/32807832/)

> We appreciate the reviewer's suggestion to broaden the Discussion by addressing FasL and TRAIL. For the reasons outlined above, we do not believe that integrating these ligands into the discussion of this study would provide meaningful insight. Indeed, TNFR1 Complex II is a cytosolic complex that forms after receptor dissociation, whereas FasL and TRAIL generate receptor-bound DISCs with distinct regulatory logic and trafficking requirements. Given these fundamental differences - and our new data demonstrating that TNF-induced cytotoxicity is uncoupled from TNFR1 trafficking - we do not expect FasL/TRAIL complexes

to be governed by the ESCRT-dependent detoxification mechanism described here. However, since our findings demonstrate that endosomal targeting of TNFR1 Complex II primarily leads to its extracellular release, we have expanded the Discussion to explore potential roles in intercellular communication and to speculate on the biological consequences of transferring TNFR1 Complex II components between cells (Page 22, line 1-30).

*> Additionally, our data argue against the possibility that increased TNF sensitivity in ESCRT-deficient cells stems from non-canonical CASP8 activation on unclosed phagophores. Even in macroautophagy-deficient conditions - where phagophores are absent - additional ESCRT deficiency further sensitizes cells to TNF-induced apoptosis (**Fig. 1i and Extended Data Fig. 1u–x, z**). Moreover, new results included in the revised manuscript show that expression of the dominant-negative RAB5^{S34N} mutant does not impair macro-autophagy (**Extended Data Fig. 2c**) and therefore is not expected to promote non-canonical CASP8 activation. Nevertheless, this mutant blocks ILV-mediated sequestration of TNFR1 Complex II and sensitizes cells to TNF-induced apoptosis (**Fig. 2b, d**). Together, these findings firmly establish that the ESCRT-dependent checkpoint operates independently of non-canonical CASP8 activation at phagophore membranes. This clarification is now briefly addressed in the Discussion section: "Moreover, the observation that ESCRT deficiency sensitizes cells to TNF cytotoxicity even in the absence of macro-autophagy also distinguishes our findings from the previously reported non-canonical, lethal activation of CASP8 on unsealed phagophores[42]. This conclusion is reinforced by the fact that expression of RAB5^{S34N} sensitizes cells to TNF-induced death without exerting any measurable effect on the macro-autophagy pathway." – Page 21, lines 15-21.*

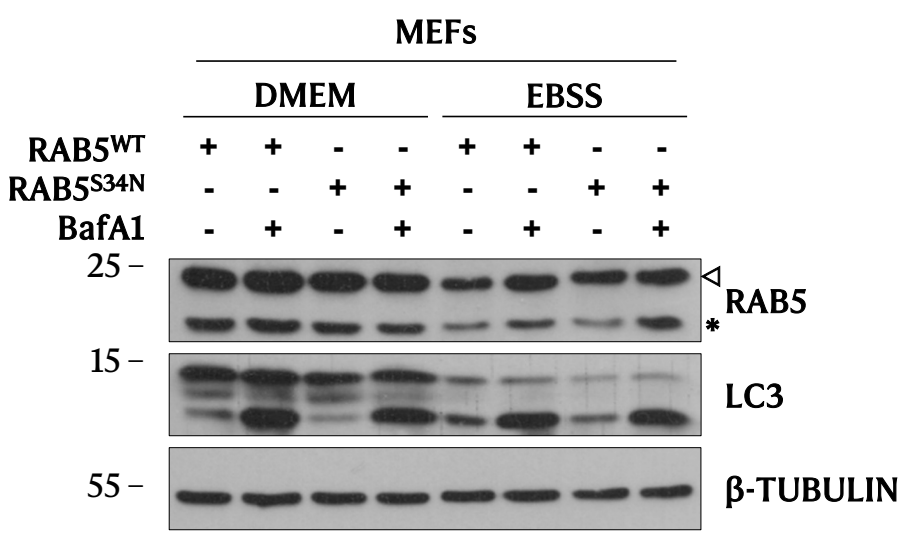

Extended Data Fig. 2c. MEFs were transduced with inducible expression vectors encoding either 3xHA-tagged wild-type (WT) RAB5 or dominant-negative mutants RAB5^{S34N}. Cells were treated, or not, with 2 µg/mL Doxycycline (Dox) for 48h. Cells were subsequently treated with EBSS for 4h in the presence or absence of BafA1. Protein levels were determined by immunoblot.

6. Please include immunoprecipitation of TNFR1 at time zero from lyzed cells (Anti-FLAG + FLAG-TNF pulldown from untreated cellular extracts) for proper validation.

> The control suggested by the reviewer is sometimes included in TNFR1 Complex I immunoprecipitation experiments, but our laboratory (Priem et al., Methods Mol. Biol., 2018) and others have routinely used the control strategy presented in our original manuscript without encountering difficulties in data interpretation or observing meaningful differences relative to time-zero controls. Importantly, none of our conclusions depend on subtle variations in baseline TNFR1 pulldown efficiency, and the newly added data - which formally demonstrate that ILV formation regulates TNF cytotoxicity independently of TNFR1 trafficking - further reinforce that including an additional time-zero control would not alter the interpretation of the results. For these reasons, we respectfully believe that repeating all TNFR1 immunoprecipitations with an additional time-zero control would not provide added value or lead to different conclusions.

7. Comment and confirm the reduced RIPK1 ubiquitination in TSG101-deficient cells. Is this reduction seen in the other experiments? Have they been performed at least 3 times independently? If so please indicate so in the manuscript and show for the referee the other two repeats.

*> While we acknowledge the reviewer's observation of a modest decrease in RIPK1 ubiquitination in TNFR1 Complex I at 5 minutes after TNF stimulation in TSG101-deficient cells, we do not consider this difference biologically meaningful. The apparent reduction correlates with a slightly lower overall amount of TNFR1 and associated TNFR1 Complex I components recovered in the immunoprecipitation from TSG101-deficient cells, suggesting a technical rather than functional origin. Importantly, we do not observe RIPK1 activation within TNFR1 Complex I - a hallmark of defective ubiquitination - and TNF-induced cell death in TSG101-deficient cells is clearly RIPK1 kinase-independent (**Fig. 1e-f**). Both observations support the conclusion that RIPK1 ubiquitination remains functionally intact in the absence of TSG101. As indicated in the figure legends, all western blot experiments were performed independently at least twice. For transparency, we now provide the data from the additional independent repeat for the reviewer's inspection (**Rebuttal Fig. 1**).*

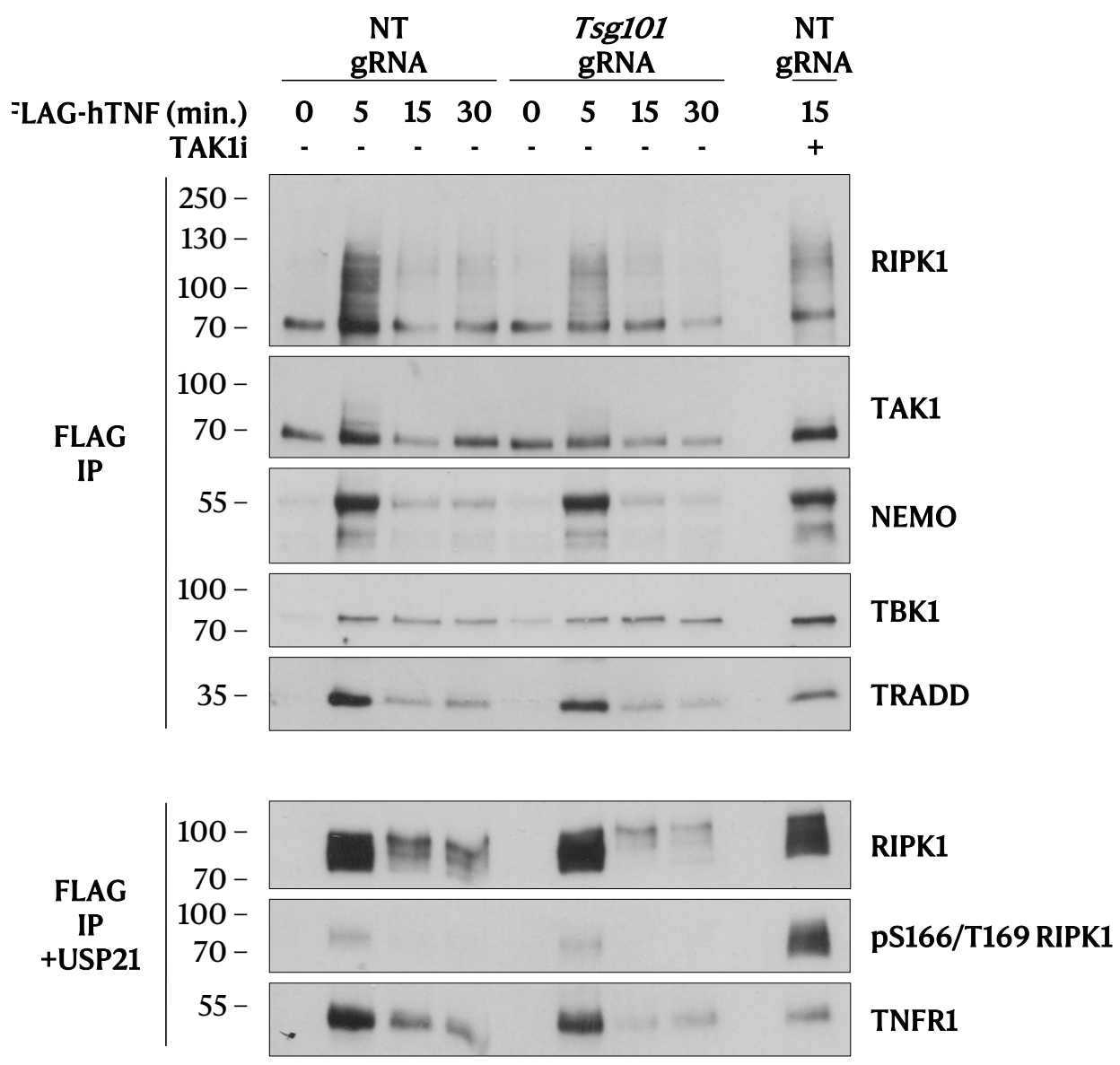

Rebuttal Fig. 1. MEFs were transduced with an inducible expression vector encoding Cas9 and subsequently transduced with inducible expression vectors encoding sgRNAs targeting *Tsg101* or NT. Cells were treated with 1 µg/mL Doxycycline for 48h and subsequently expanded for 72h. Cells were pre-treated, or not, with TAK1i prior to stimulation with 1 µg/mL FLAG-hTNF for the indicated duration. TNFR1 Complex I was FLAG-immunoprecipitated, followed by USP21 treatment, or not. Protein levels were determined by immunoblot.

8. It is unclear why VPS34 inhibition (with drug) enhances TNF-induced death in MEF cells, while not in HT1080 cells (e.g., S1U-V vs S1X). Please clarify these discrepancies and include side-by-side comparisons of VPS34 KD (gRNA) vs inhibitor treatments.

> We acknowledge the reviewer's observation that VPS34 inhibition results in higher TNF cytotoxicity in MEFs than HT1080 cells. Although the basis for this difference remains unclear, the effect is consistent with the results of our previous study, where it was found not to be specific to VPS34. Indeed, similar differences were also observed upon inactivation of other components of the LC3-independent macro-autophagy pathway, such as ATG9A or FIP200 (Huyghe et al., Science 2022 - Fig. S1F–G). These results therefore indicate that HT1080 cells are more resistant than MEFs to inactivation of the LC3-independent macro-autophagy checkpoint. In the same study, we also directly compared pharmacological inhibition of VPS34 with SAR405 to CRISPR-mediated VPS34 deletion and observed no

substantial differences between these two approaches (Huyghe et al., *Science 2022 - Fig. 2C, Fig. S3E–F).*

9. Were DGUC/SEC and EV analyses done in the presence or absence of CHX? This is not stated and is crucial for interpretation. If not a control including CHX would be needed as this would match confocal data.

*> We thank the reviewer for highlighting the importance of clarifying whether CHX was used during EV isolation. As stated in the original figure legends, all DGUC/SEC-based EV isolations were performed exclusively on cells not pre-treated with CHX. CHX was only used in microscopy experiments, where it is necessary to amplify CASP8 activation sufficiently to allow reliable detection of Cl. CASP8 puncta by confocal imaging. Without CHX, the signal remains below the single-cell detection threshold. To directly bridge the microscopy and EV datasets, we additionally performed EV isolations from TNF-treated cells in the presence of CHX. As expected, CHX pre-treatment increased the amount of Cl. CASP8 detected in EVs (**Extended Data Fig. 4s**), consistent with its ability to increase the cellular pool of TNFR1 Complex II above the imaging detection limit. However, CHX is a potent sensitizer of apoptosis (**Extended Data Fig. 4r**), raising the possibility of apoptotic body contamination—a concern also raised by the reviewer. To exclude this, we repeated the experiment in apoptosis-resistant CASP3/7-deficient HT1080 cells (**Extended Data Fig. 4t**), which cannot form apoptotic bodies. Even in this background, CHX pre-treatment similarly increased Cl. CASP8 levels in EVs (**Extended Data Fig. 4u**) confirming that the additional signal does not originate from apoptotic bodies. Importantly, and central to our conclusions, TNFR1 Complex II is readily detectable in EVs even in the absence of CHX, demonstrating that this phenomenon does not depend on artificially boosting CASP8 activation. We have now incorporated these additional data into the revised manuscript for clarity (**Extended Data Fig. 4r-u**).*

ED Fig. 4r

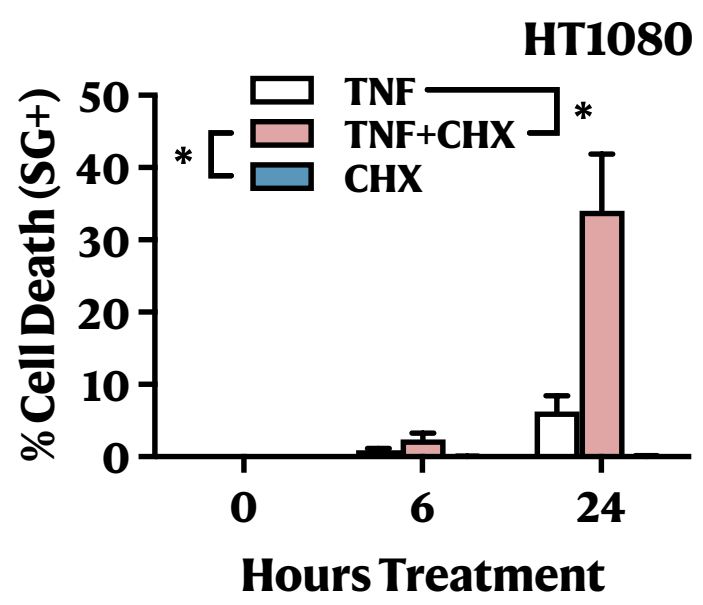

ED Fig. 4s

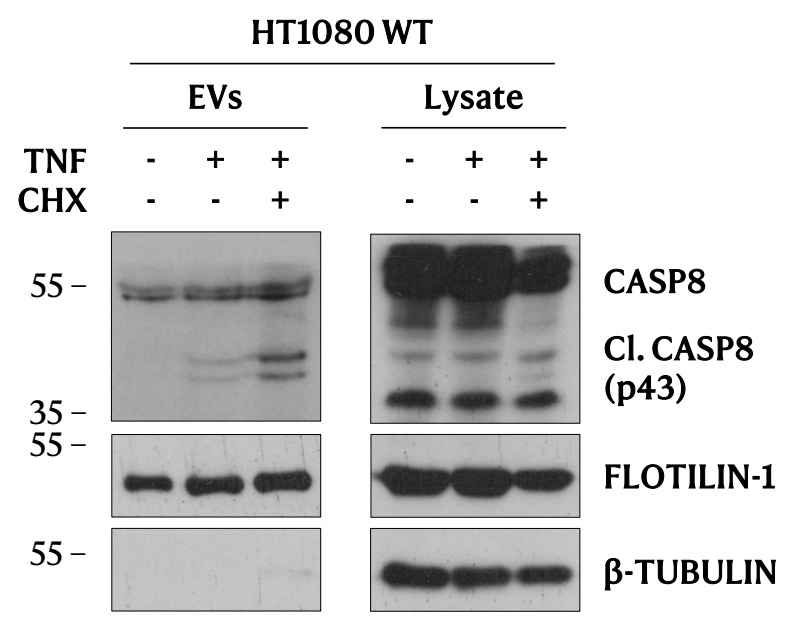

ED Fig. 4t

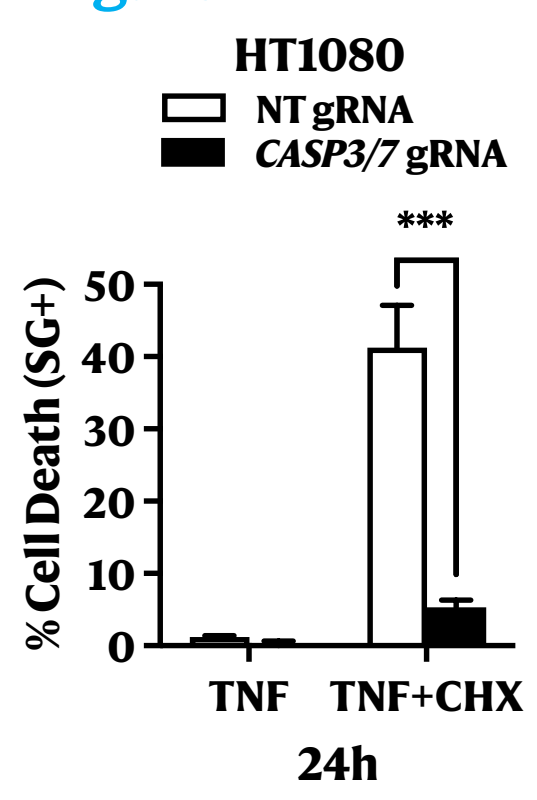

ED Fig. 4u

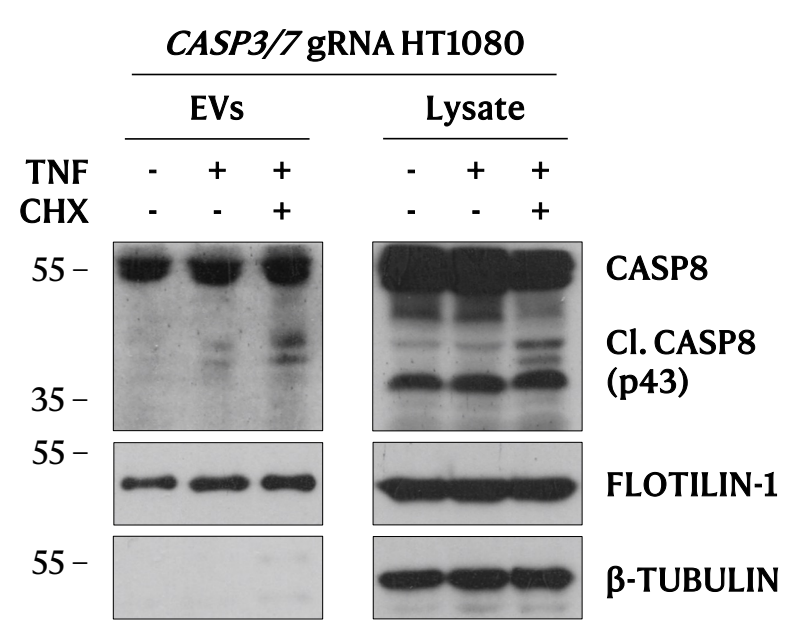

Extended Data (ED) Fig. 4r-u. ED Fig. 4r, HT1080 cells were pre-treated with CHX, as indicated, prior to stimulation with 20 ng/mL hTNF. Cell death was measured over time by SytoxGreen (SG) positivity. **ED Fig. 4t-u**, HT1080 cells were electroporated with Cas9-RNPs targeting *CASP3* and *CASP7* or a non-targeting sequence (NT). **ED Fig. 4t**, Cells were pre-treated with CHX, as indicated, prior to stimulation with 20 ng/mL hTNF. Cell death was measured after 24h by SytoxGreen (SG) positivity. **ED Fig. 4s, u**, Cells were pre-treated with CHX, as indicated, prior to stimulation with 2 μg/mL hTNF for 6h. Cells were lysed and extracellular vesicles (EVs) were isolated from the conditioned medium using size exclusion chromatography (SEC) (Fractions 4-6). Protein levels were determined by immunoblot. Cell death experiments are presented as mean ± SEM of three independent experiments and statistical significance was determined using a two-way ANOVA or multiple unpaired t-tests.

10. Why is FADD not detected in HT1080 EVs (Fig. S3J)?

*> A closer examination of the figure (**Extended Data Fig. 4p**) shows weaker bands corresponding to FADD, which is consistent with our other data on the EV content of TNF-treated cells. The reduced signal in this particular experiment most likely reflects a shorter exposure time. Importantly, the EV lysates analyzed in **Fig. 4h** were prepared using the same SEC protocol and show much stronger FADD signals, confirming the reproducibility of this finding.*

11. The presence of HSC70 in EVs is not shown. Only indirect evidence via sensitivity to TNF is provided to point to HSC70 as a cargo protein for eMI. Please include HSC70 in your biochemical EV analyses.

> We thank the reviewer for this valuable suggestion. In the original manuscript, we demonstrated that HSC70—independently of its ATPase activity—is required for the transfer of TNFR1 Complex II into endosomes and for protection against TNF cytotoxicity. However, we agree that directly assessing the presence of HSC70 in EVs provides important complementary evidence. To address this, we performed additional biochemical analyses of EVs isolated by SEC following TNF stimulation. In addition to the expected TNF-dependent enrichment of linear ubiquitin chains, ubiquitinated RIPK1, and TAX1BP1, we now observe increased levels of HSC70 in SEC fractions enriched for established exosome markers (TSG101, ALIX, and FLOTILLIN-1) (Fig. 5a). These findings are fully consistent with the proposed role of HSC70 in selectively facilitating the loading of TNFR1 Complex II into ILVs. The new data have been incorporated into the revised manuscript.

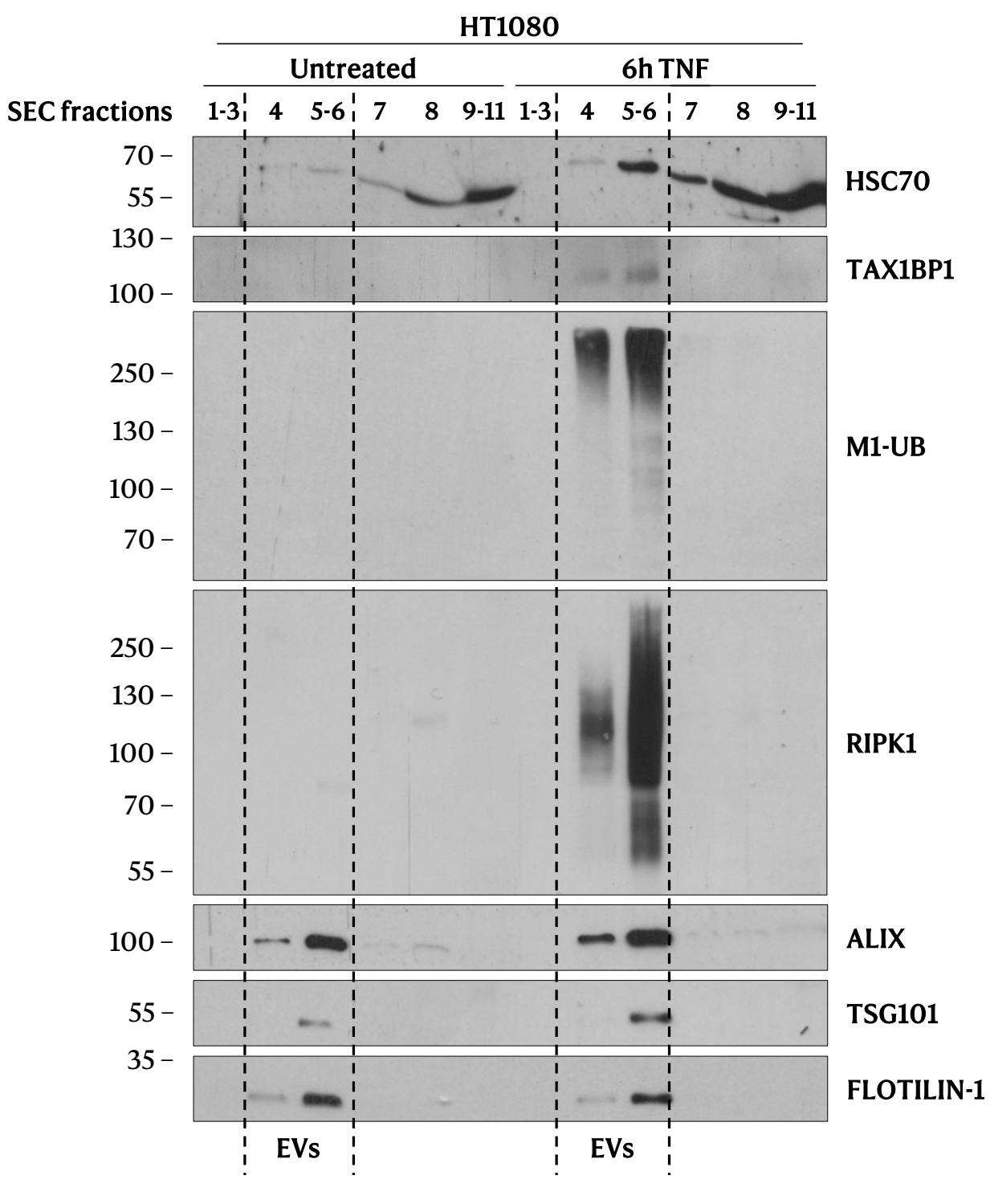

Fig. 5a. HT1080 cells were stimulated, or not, with 2 μg/mL hTNF for 6h. Cells were lysed and conditioned medium was harvested and separated into different fractions using size exclusion chromatography (SEC). Protein levels in each fraction were determined by immunoblot.

12. Clearly label each panel with the relevant cell line (MEF, HT1080, etc.), as it is not always obvious.

> We thank the reviewer for this suggestion and have updated the labeling of the figure panels accordingly to improve interpretability and readability.

13. Figure S1S: Immunoblots showing TSG101 deficiency in MEF-ATG9a−/− cells should include WT MEFs for reference.

> The purpose of introducing TSG101 deficiency into ATG9A-deficient MEFs was solely to assess the impact of ESCRT loss in a macro-autophagy-deficient background. At no point in the manuscript do we directly compare ATG9A-deficient cells with wild-type MEFs. For this reason, we respectfully disagree that including such a comparison would represent an informative control or alter the interpretation of our results.

14. Figure 3g: The caspase-8 blot shows three bands—please clarify the molecular identity of each (e.g., p55, p43/p41, p18).

> We thank the reviewer for this suggestion and have updated the labeling of the figure panels accordingly to improve interpretability and readability.

15. Figure 5: Textual Issue: The sentence "...sensitizing effects of SopB and EsxG-H were lost when cells were pre-treated with USPsi..." contradicts Fig. 5b vs 5e. At most, you can conclude that SopB's effect is additive to or masked by USP8 inhibition, but not abolished. Please revise for accuracy.

*> We agree with the reviewer that the interpretation of these results was not correctly phrased in this sentence. We have therefore rephrased to increase clarity: "This notion was further supported by the fact that SopB and EsxG-H have no additional sensitizing effect when the ESCRT machinery is already inhibited by pre-treatment with USP8i (**Fig. 6e** and **Extended Data Fig. 6h**)." - Page 17, lines 29-31.*

16. "..The discrepancy between the two studies concerning the effect of ESCRT deletion on TNF-mediated NF- κB/MAPK signaling is unclear, but may reflect the cell type-specific targeting of TNFR1 Complex Ito the endolysosomal system..." Which studies, please add references.

> We note that the study by Song et al. *was referenced earlier in the manuscript when discussing discrepancies with our findings. However, we agree with the reviewer that an additional citation at this point is helpful to improve clarity and have therefore added this to the revised manuscript.*

17. "...phosphorylation of RIPK3 and MLKL (markers of necroptosis induction) were not observed upon stimulation of either wild type (WT) or ESCRT-deficient MEFs with TNF..." Could you dd the precision of the time at which you did this analysis as this may not be true at other time points!

> We thank the reviewer for pointing this out and have rephrased the Results section to describe the data more precisely.

18. The Original title: "Endosomal micro-phagy prevents TNF-induced cell death by targeting TNFR1 complex II" is vague, could you consider the following one "Endosomal Microautophagy Limits TNF-Induced Apoptosis by Redirecting TNFR1 Complex II for Exosomal Secretion" to describe ore precisely your conclusions?

> We thank the reviewer for this constructive comment. In line with remarks from other reviewers, we performed additional experiments to evaluate whether ESCRT-dependent sequestration of TNFR1 Complex II into ILVs leads to lysosomal degradation. The new results indicate that this process primarily mediates extracellular release of the complex via exosomes rather than lysosomal targeting. These data have been added to the revised manuscript. Since this mechanism does not strictly qualify as autophagy, we have rephrased the title and adjusted the manuscript to more accurately reflect our findings: "Exosomal Export of TNFR1 Complex II Prevents TNF-Induced Cytotoxicity".

Reviewer #4

1. Graphical abstract to explain the mechanism in the manuscript

*> We agree with the reviewer that a graphical abstract would be a valuable addition to improve accessibility for readers. We have therefore incorporated a graphical abstract that provides a graphical summary of the key findings and a detailed overview of the proposed mechanism in the last figure panel of the revised manuscript (**Fig. 6j**).*

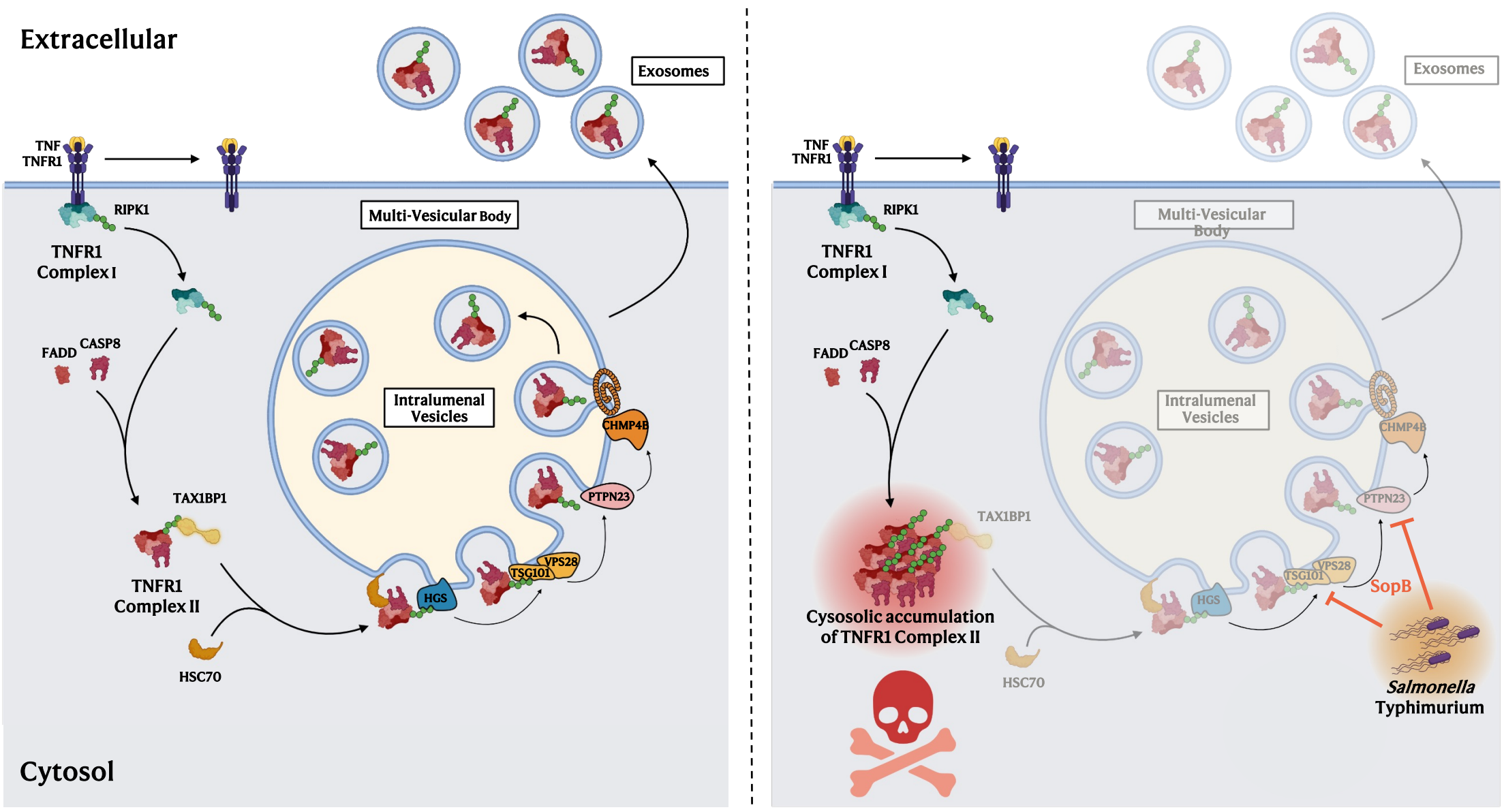

Fig. 6i. Graphical overview of the ESCRT-dependent detoxification of TNFR1 Complex II via extracellular release. Upon TNF sensing, TNFR1 Complex I assembles at the plasma membrane. RIPK1 and TRADD subsequently dissociate from the receptor-associated complex and join FADD and CASP8 to form the cytosolic TNFR1 Complex II. LUBAC decorates RIPK1 and additional TNFR1 Complex I/II components with M1-linked ubiquitin chains, which are recognized by the autophagy receptor TAX1BP1. Together with HSC70, TAX1BP1 then promotes the recruitment of TNFR1 Complex II to the limiting membrane of endosomes, where the ESCRT machinery mediates its incorporation into intralumenal vesicles (ILVs). The resulting Multivesicular body (MVB) can then fuse with the plasma membrane to release ILV-embedded TNFR1 Complex II into the extracellular environment as part of exosomes. Disruption of this pathway - such as by ESCRT inhibition due to the action of the *Salmonella* Typhimurium effector protein SopB - prevents the ILV-dependent detoxification of TNFR1 Complex II, leading to its cytosolic accumulation and the induction of TNF-induced RIPK1 kinase–independent apoptosis.

2. More detailed explanation of medical implications of the pathway explored is needed

> We agree with the reviewer that the original manuscript contained only a limited discussion of the potential medical implications. In the revised version, we have expanded the Discussion to more thoroughly speculate on the pathophysiological relevance of the identified pathway and its potential impact in disease contexts: "While transient inactivation of a cell death checkpoint can be beneficial in the context of infection, its constitutive inhibition is instead detrimental and causes pathogenesis[1]. Interestingly, numerous human mutations in components of the ESCRT machinery have been identified across patients presenting with diverse clinical symptoms[73]. Because the ESCRT components fulfil many functions beyond ILV formation, pinpointing the specific contribution of impaired TNFR1 Complex II detoxification to these disease manifestations will be challenging. Nevertheless,

our findings indicate that excessive TNF-induced cell death may contribute to the pathology in at least a subset of cases. Notably, together with recent reports, our data suggest a more specific role of PTPN23 in directing cytosolic cargo into ILVs[15,29]. Homozygous or compound heterozygous mutations in PTPN23 cause the neurologic disorder NEDBASS, characterized by neurodevelopmental delay, structural brain abnormalities, impaired intellectual development with delayed or absent speech, axial hypotonia, and peripheral spasticity[74–76]. It will be important to determine whether these mutations disrupt the exosomal detoxification of TNFR1 Complex II and whether uncontrolled TNF-induced cell death contributes to disease progression. If so, anti-TNF biologics could represent a promising therapeutic avenue for affected patients." – Page 24, lines 25-31 and Page 25, lines 1-10.

3. Fig 1 shows USP8 inhibitor, along with the TNF, induces TNF-mediated cell death. Additionally, TNF-mediated cell death is been induced in ESCRT mutant components.

*> We regret that the reviewer's remark is not entirely clear to us. The data in **Fig. 1** illustrates that both pharmacological and genetic targeting of the ESCRT machinery sensitize cells to TNF-induced death, highlighting the importance of the ESCRT components in counteracting TNF cytotoxicity.*

4. Fig 1c- Why there are repeated cleaved Casp. 3 blots? It is mentioned in the text (page 5, line 25) for the PARP but not shown in the results.

*> We assume that the reviewer is referring to repeated Cl. CASP8 blots? The different blots for Cl. CASP8 represent different cleavage fragments. For clarity, we have now explicitly labeled the individual fragments in the figure. The text refers to the cleavage of both CASP8, CASP3 and PARP. The reviewer may have missed that Cl. PARP was already shown in **Fig. 1c** of the original manuscript.*

5. Fig 1b, c, d- No information for the used cell lines.

> We thank the reviewer for this remark. We have updated the figure panel labels to clearly indicate the respective cell lines used, thereby improving readability and interpretability.

6. Most of the graphs of % cell death (Fig 1e, extended 1g) show high cell death (more than or up to 50%) in the DMSO. How is it true?

> In Fig. 1e of the original manuscript, the data are from MEFs with CRISPR-mediated Tsg101 deficiency. In these cells, treatment with TNF in combination with DMSO (control for Nec1s) results in sensitization to TNF cytotoxicity due to loss of TSG101. In Extended Data Fig. 1g of the original manuscript, the data are from HT1080 cells treated with TNF and USP8i, with or without Nec1s. Here, DMSO also serves as the control for Nec1s, and these cells are sensitized to TNF cytotoxicity due to USP8 inhibition.

7. Page 6, line 23 "In contrast to....". This statement cannot be aligned with the fig 1f.

*> We acknowledge the reviewer's observation that minor differences in TNFR1 Complex I assembly could be inferred from **Fig. 1f**. However, we do not consider these to represent a substantial biological effect, but rather technical variation. Indeed, we do not observe major differences in the ability of TSG101-deficient cells to activate the NF-kB and MAPKs pathways in response to TNF (**Fig. 1g**). In addition, we also do not detect RIPK1 activation within TNFR1 Complex I in these cells, which is a hallmark of defective TNFR1 Complex I assembly. Furthermore, the cell death observed in TSG101-deficient cells is RIPK1 kinase-independent, supporting the conclusion that TNFR1 Complex I assembly remains intact. Finally, in the compared publication, the authors report enhanced, and not reduced, signaling by TNFR1.*

8. Ext. fig. 1J- TNFR1 blot is not clear

> We agree with the reviewer that this blot is not optimal in clarity. We have therefore replaced it with a higher-quality version in the revised manuscript.

We were happy to learn that the extensive revisions brought to our initial submission adequately addressed all the comments from 2 (Reviewer #2 and #4) out of the 4 reviewers, pending minor textual changes for Reviewer #2. We were also pleased to know that Reviewer #2 found that our revision had properly addressed all the points initially raised by Reviewer #1.

Please find below our point-by-point answers to the remaining remarks raised by Reviewer #3, as well as to the further comments brought by Reviewer #1 after the editorial decision was made.

Changes made to the manuscript and Figures are highlighted in green in this secondly revised manuscript.

Reviewer #1

1. Regarding my original point 2 ("The protective role of ESCRT components on TNF-induced necroptosis should be further explored…"), if the WT MEFs die at < 50% with TNF+zVAD, this means they are capable of dying by necroptosis. If the detoxification pathway in ILVs is via RIPK1 and its M1-Ub (and RIPK1 is necessary in complex II to allow for necroptosis), how can disruption of this pathway not impact cell death? This remains to be clarified.

> > We appreciate the reviewer's thoughtful comment. We would like to emphasize that the necrosome formed under TNF+zVAD conditions is mechanistically and structurally distinct from the TNFR1 Complex II that forms upon TNF stimulation alone. Although both complexes contain RIPK1 and linear ubiquitin chains, the necrosome is formed via RHIM-dependent RIPK1–RIPK3 oligomerization into large amyloid-like fibrillar structures, as previously reported. We believe that the supramolecular size and architecture of the necrosome likely preclude its efficient sequestration into intraluminal vesicles (ILVs), which typically have diameters of ~50 nm.

2. Regarding my original point 4 ("In Figure 3, the authors aim to demonstrate the presence of TNF complex II…"), the claim that FADD and EEA1 colocalize is not substantiated by the data presented. For this claim to be made there would need to be a 3D reconstitution or other type of analysis. In the presented images it could well be that they are simply sitting on top of each other. The presence of Caspase 8 and FADD in basal conditions is the control for this experiment. The authors show that only in TNF they co-immunoprecipitate, yet they are still there in the control. In my view, addressing this point is not only in the scope of this study but needs to be adequately addressed in it.

> > We thank the reviewer for this thoughtful comment. We agree that rigorous assessment of FADD–EEA1 colocalization is important for proper interpretation. Accordingly, we have now included a 3D reconstruction of the confocal datasets in the revised manuscript (**Extended Data Fig. 4h**), which demonstrates spatial association between TNF-induced FADD puncta and EEA1^{+} endosomes.
>
> > Because FADD and CASP8 do not form discrete puncta in unstimulated cells, their basal trafficking cannot be meaningfully interrogated by confocal imaging. CASP8 and FADD can therefore not be used as controls in untreated conditions for this experiment.

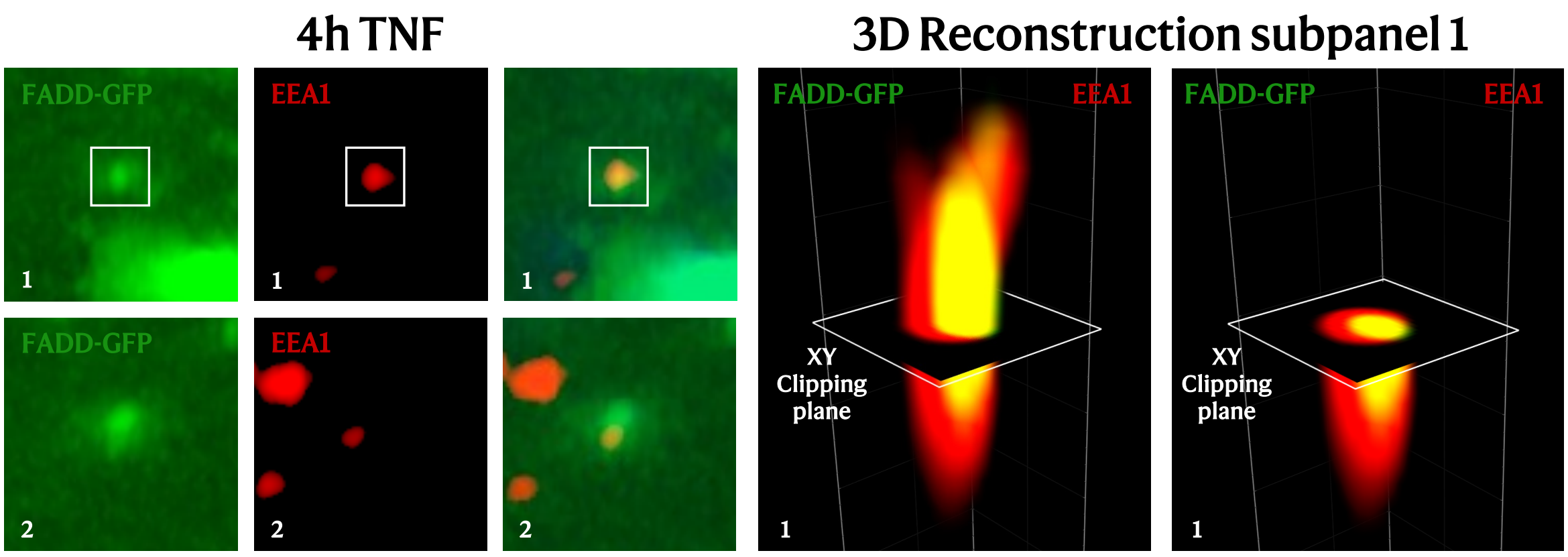

Extended Data Fig. 4h. Representative images of EEA1 immunostainings are shown. Images represent maximum intensity projections along the Z-plane of imaged Z-stacks. h, Single and merged channel images of the zoomed subpanels of cells treated with TNF for 4h are shown including a 3D reconstruction of zoomed subpanel 1. The reconstructed image was clipped along an XY-plane to allow visualization of the contents of EEA1+ endosomes.

3. Regarding my original point 6 ("In Figure 4, a model is proposed where M1-Ub RIPK1 is proposed as a scaffold for TAXBP1..."), to formally conclude that Lin-Ub is necessary for the cargo recognition, the possibility that Lin-Ub is involved in the formation and stability of these vesicles would need to be excluded. In the figures shown in 5f the number of vesicles in the mCherry-RAB5Q79L HOIP gRNA conditions appears significantly lower than in other genotypes (in addition, not only the number but also size of these vesicles should be evaluated). The authors show a clear decrease in cleaved Caspase 8 punctae in RIPK1 KO cells. Furthermore, they see RIPK1 KO sensitizing Atg9a-deficient MEFs. However, does RIPK1 KO MEFs WT vs Atg9a look any different in terms of cell death sensitivity? This important controls for the cell death experiments needs to be included.

> We appreciate the reviewer's thoughtful comment that loss of TAX1BP1, HOIP, or RIPK1 could, in principle, affect MVB biogenesis or stability rather than selectively impair cargo targeting. To address this directly, we reanalyzed our imaging data and quantified both the number and size of mCherry–RAB5^{Q79L+} endosomes across the indicated genotypes. Importantly, inhibition of TSG101, USP8, HOIP, RIPK1 or TAX1BP1 did not reduce endosome abundance or size, arguing against a major defect in endosome formation or stability. Notably, inhibition of HOIP, RIPK1, and TAX1BP1 even resulted in a modest increase in endosome number or size, potentially reflecting a compensatory response. These quantifications have now been included in the revised manuscript (**Extended Data Fig. 4k**; **Extended Data Fig. 5e, i, l**). These results support our model that Lin-Ub is necessary for the cargo recognition.

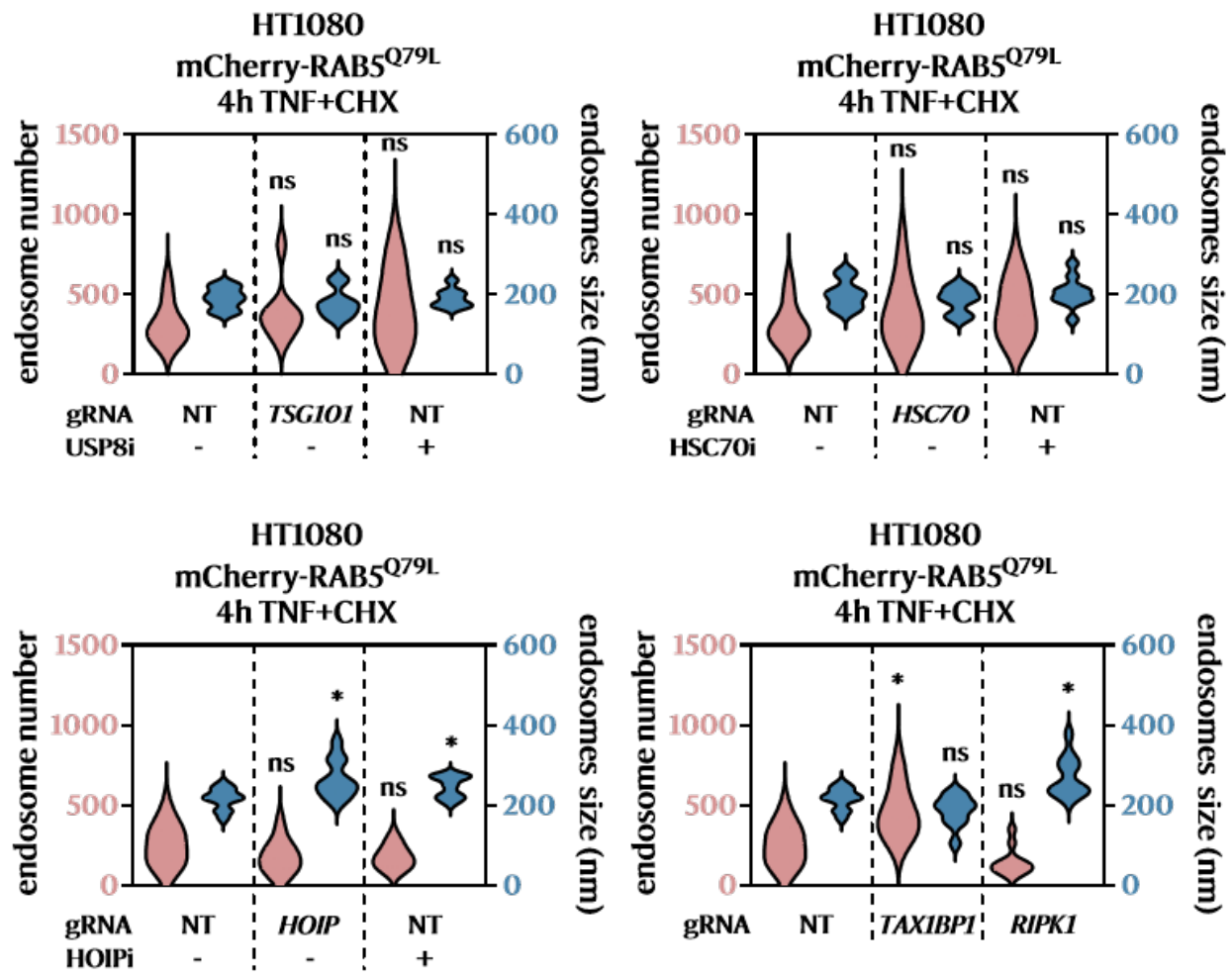

Extended Data Fig. 4k & 5e, i, l HT1080 cells were transduced with an inducible expression vector encoding mCherry-RAB5^{Q79L}. Transduced cells were electroporated with Cas9-RNPs targeting the indicated genes or a non-targeting sequence (NT). Cells were treated with 2 µg/mL Dox for 48h. Subsequently, cells were pre-treated with CHX and USP8i, HSC70i or HOIPi as indicated before stimulation with 1 µg/mL hTNF for 4h. Cells were then stained for Cl. CASP8. The average number and size of mCherry-RAB5^{Q79L+} endosomes were quantified.

> Regarding the suggested comparison of RIPK1-deficient MEFs with or without additional ATG9A deficiency, we would like to clarify that this experiment primarily informs on a potential RIPK1-independent pro-survival function of ATG9A rather than on the role of RIPK1 in cargo recognition during exosomal detoxification of TNFR1 Complex II. Notably, in our previous study (Huyghe et al., *Science* 2022) we examined ATG9A deficiency in a HOIP-deficient background and observed no additive effect on TNF cytotoxicity (Fig. 1I), indicating that the cytoprotective function of ATG9A fully depends on linear ubiquitination. This finding is consistent with a model in which RIPK1 functions as a linearly ubiquitinated scaffold. Given that ATG9A is not the primary focus of the present study, and that existing data already address this relationship, we respectfully consider additional experiments in this direction to be beyond the scope required to support our conclusions.

4. No pRIPK1 is shown in any input blot, only in the IPs. This is, however, an essential control and needs to be included.

> We appreciate the reviewer's comment. However, under TNF stimulation alone, the total cellular levels of phosphorylated RIPK1 (pRIPK1) are very low and fall below the detection limit in whole-cell lysates. Reliable detection is only achieved after enrichment via TNFR1 Complex I immunoprecipitation. For this reason, inclusion of an input blot would not provide informative signal and was therefore not included in the final figure.

5. The experiments for all these graphs added in the revision to check TNFR1 degradation employ exogenous TNF-V5. The authors need to test whether or not the V5 tag plays a role in rendering TNFR1 more susceptible to degradation via selective autophagy.

> The use of TNFR1–V5 to monitor receptor dynamics is primarily technical, as reliable detection of endogenous TNFR1 in whole-cell lysates is challenging due to the limited sensitivity of available antibodies. The V5 tag therefore enables accurate assessment of TNFR1 trafficking. In agreement with previous reports on WT TNFR1, we show that TNF sensing also induces endolysosomal degradation of TNFR1–V5, which was effectively blocked by Dyngo-4a, BafA1 pre-treatment, or VPS36 deficiency, demonstrating that the observed trafficking behavior of TNFR1–V5 remains regulatable and pathway-dependent. Importantly, the impact of perturbing TNFR1 trafficking on TNF cytotoxicity was assessed in WT MEFs expressing endogenous TNFR1, ensuring that our conclusions on TNFR1-driven cytotoxicity are true in WT cells. Moreover, TNFR1–V5 MEFs retain full competence to undergo RIPK1 kinase-independent apoptosis, RIPK1 kinase-dependent apoptosis, and RIPK1 kinase-dependent necroptosis (data not shown), indicating that the tag does not impact receptor functionally.

Reviewer #2

1. Legend of Fig S3A: The asterisk is not explained. I assume that that band is a background band and the real one is the smaller one for FADD.

> We apologize for the lack of clarity. The assumption of the reviewer was correct, and the legend of Fig. 3A has now been adapted accordingly.

P14, line 25: TNF treatment leads to targeting of receptor complex II to exosomes…. Delete the word 'selective' or specify why selective. There likely still are other proteins in these exosomes. Key is, the complex goes there.

> The text has been adapted as suggested by the reviewer.

Reviewer #3

1. Despite extensive revision, your manuscript still does not fully support your claim that complex II is being "detoxified" at the steady state preventing TNF-induced cell death. First of all because you fail to demonstrate convincingly that the complex II is present in these vesicles.

> We respectfully disagree with the reviewer's assessment and believe that the revised manuscript provides multiple, independent lines of evidence supporting the conclusion that TNFR1 Complex II is targeted for exosomal disposal, thereby limiting TNF-induced cytotoxicity under steady-state conditions.

First, using confocal microscopy, we demonstrate that TNFR1 Complex II components are targeted to intraluminal vesicles in an ESCRT, HSC70 and TAX1BP1-dependent manner. This observation is fully consistent with the established role of the ESCRT machinery in multivesicular body formation and exosome biogenesis, and provides spatial evidence that TNFR1 Complex II is sorted into the exosomal pathway.

Second, we show that the core components of TNFR1 Complex II are detected in exosomes isolated from TNF-treated cells. Exosomes were purified using three independent and complementary approaches - ultracentrifugation, size exclusion chromatography (SEC), and density-gradient ultracentrifugation - thereby minimizing the possibility of contamination by non-exosomal material. The isolated vesicles were rigorously characterized by nanoparticle tracking analysis and electron microscopy, confirming their size, morphology, and purity. Moreover, potential contamination by apoptotic bodies was definitively excluded using the most appropriated genetic approach.

Third, we demonstrate that these components are not merely present individually, but are part of an intact signaling complex within exosomes. Specifically, we show a TNF-dependent interaction between CASP8 and FADD by co-immunoprecipitation performed on purified exosomal fractions, providing biochemical evidence for the presence of assembled TNFR1 Complex II in these vesicles.

We would like to highlight the fact that the isolation and biochemical analysis of exosome-associated signaling complexes are technically demanding and require substantial input material and stringent experimental controls. Nonetheless, the convergence of imaging, biochemical purification, vesicle characterization, and interaction data collectively supports the conclusion that TNFR1 Complex II is selectively targeted to exosomes. We therefore believe that the revised manuscript adequately addressed the reviewer's concern and substantiate our claim that exosomal export contributes to the detoxification of TNFR1 Complex II following TNF sensing.

2. While the additional experiments addressing TNFR1 trafficking are appreciated, they do not fully resolve the concern raised. In particular, the authors do not directly address the observation highlighted in Figure 2g, where increased amounts of TNFR1 are detected in Complex I in RAB5-S34N–expressing cells. This point is neither discussed nor reconciled with the revised model, and the removal of the TNFR1 blot from the revised figure (now Fig. 2h) further complicates interpretation. More fundamentally, although TNFR1 is not retained within Complex II after engagement, the formation of TNFR1 Complex II depends on multiple upstream events, including proteolytic processing of TNFR1 by γ-secretases (see doi:10.1074/jbc.M115.679076). The activity, localization, or availability of these proteases, as well as the pool of membrane-associated TNFR1, could plausibly be altered in cells stably expressing RAB5-S34N or lacking ESCRT components. Such effects could influence Complex II formation independently of ILV-mediated sequestration.

>We agree with the reviewer that TNFR1 trafficking is probably altered in some of our experimental models. For this reason, we directly addressed the central question of whether defects in TNFR1 trafficking can switch (or contribute to the switch of) the TNF response from survival to cell death. To this end, we introduced a completely new set of experiments, presented as a new figure in the revised manuscript.

In these experiments, we selectively interfered with TNFR1 trafficking at distinct stages of the endocytic pathway: at the plasma membrane (DYNAMIN-1 inhibition by Dyngo-4a), at the endosomal membrane (VPS36/ESCRT-II depletion), and at the level of intraluminal vesicle formation (V-ATPase inhibition using BafA1). Importantly, none of these interventions resulted in increased TNF cytotoxicity. These data therefore conclusively exclude a protective role for endolysosomal TNFR1 trafficking in preventing TNF-induced cell death. This conclusion is mechanistically consistent with the established notion that TNF-induced cell death is mediated by the cytosolic, receptor-dissociated TNFR1 Complex II rather than the receptor-bound Complex I.

To further demonstrate that the cytoprotective role of the ESCRTs is dependent on the sorting of Complex II, and not of TNFR1, into ILVs, we tested the effect of ESCRT inhibition by USP8i in cells in which TNFR1 internalization is blocked by Dyngo-4a. Remarkably, USP8i treatment still switched the TNF response from survival to apoptosis in these conditions. Together, these data conclusively demonstrate that defective ILV-mediated detoxification of Complex II, and not altered TNFR1 trafficking, is the cause of the observed TNF cytotoxicity.

Given that most experimental models used in this study involve stable expression or stable knockout of trafficking regulators, a direct assessment of TNFR1 surface expression would be straightforward and informative. Measuring membrane-bound TNFR1 by flow cytometry across the relevant conditions would help exclude the possibility that variations in TNFR1 availability at the plasma or altered receptor processing contribute, at least in part, to the increased sensitivity to TNF-induced cell death. Moreover, because the presence of TNFR1 Complex II within extracellular vesicles has not been fully established, in my opinion (see point 10) such controls remain important to support the authors' central conclusion.

> In response to the reviewer's request, we assessed TNFR1 surface expression. To this end, we isolated surface-exposed, ligand-binding–competent TNFR1 by immunoprecipitation under steady-state conditions (by 2h FLAG-hTNF pre-treatment on ice). We did not detect significant changes in TNFR1 surface levels across conditions (Extended Data Fig. 3e–g). These data demonstrate that the increased sensitivity to TNF cytotoxicity observed in ESCRT-deficient cells and in cells expressing the RAB5 S34N mutant is not due TNFR1 accumulation at the plasma membrane.

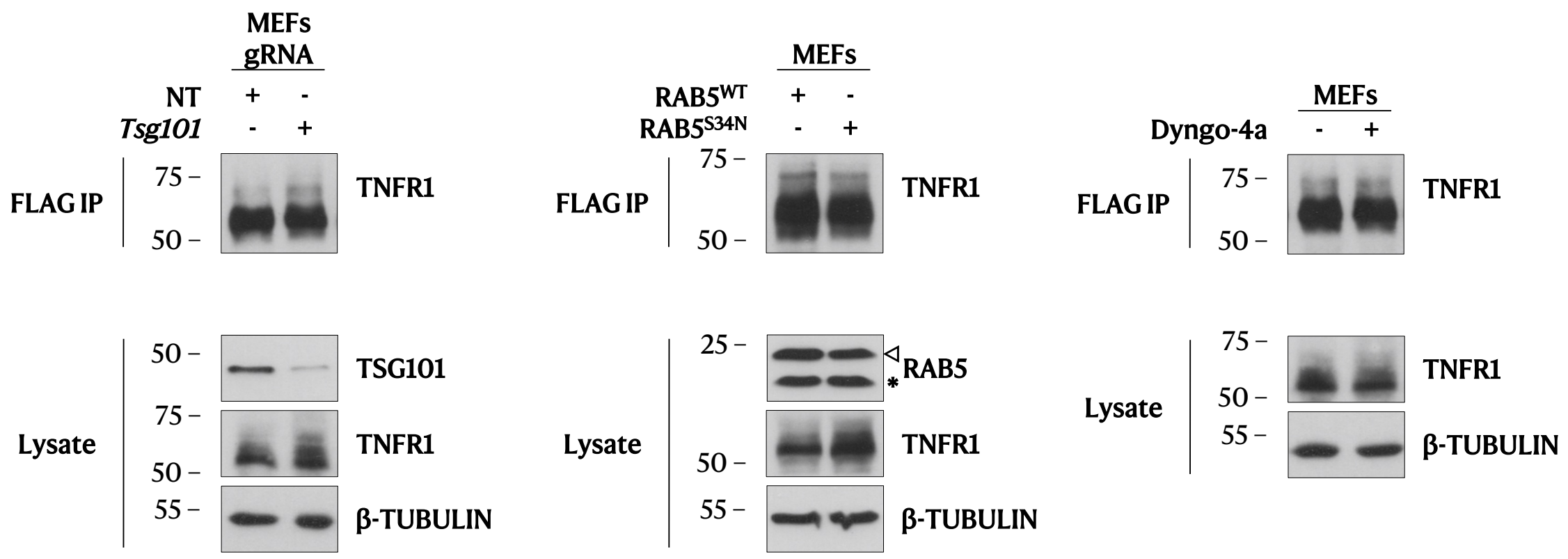

Extended Data Fig. 3e-g. e, MEFs were transduced with an inducible expression vector encoding Cas9 (hereafter referred to as iCas9 MEFs) and subsequently transduced with inducible expression vectors encoding sgRNAs targeting *Tsg101* or a non-targeting sequence (NT). Cells were treated with 1 µg/mL Doxycycline (Dox) for 48h and subsequently expanded for 72h. **f**, MEFs were transduced with inducible expression vectors encoding either 3xHA-tagged wild-type RAB5 ($RAB5^{WT}$) or the dominant-negative RAB5 S34N mutant ($RAB5^{S34N}$). Cells were treated, or not, with 2 µg/mL Doxycycline (Dox) for 48h. **g**, MEFs were pre-treated with Dyngo-4a as indicated. **e-g**, Cells were subsequently incubated with ice-cold medium containing 1 µg/mL FLAG-hTNF for 2h. Surface-accessible TNFR1 was subsequently FLAG-immunoprecipitated. Protein levels were determined by immunoblot.

3. The clarification regarding figure annotation and labeling is appreciated. However, the second part of my concern is not fully addressed. While reference to prior work is useful, the current manuscript would benefit from a clear, systematic side-by-side comparison of TNF-induced cell death in WT and ATG9A-deficient cells, in the presence and absence of CHX, performed within the same experimental framework. Such direct comparison would substantially improve the readability and interpretability of the results and would allow the reader to assess more clearly how ATG9A loss modulates TNF-induced cytotoxicity under different conditions. Relying exclusively on previously published data limits the self-contained nature of the present study.

> At the reviewer's request, we repeated this comparison within the current experimental framework and the results can be found below. Because this experiment is only confirmatory in nature and does not impact on the interpretation or conclusions of the present study, we did not include it in the revised version of the manuscript.

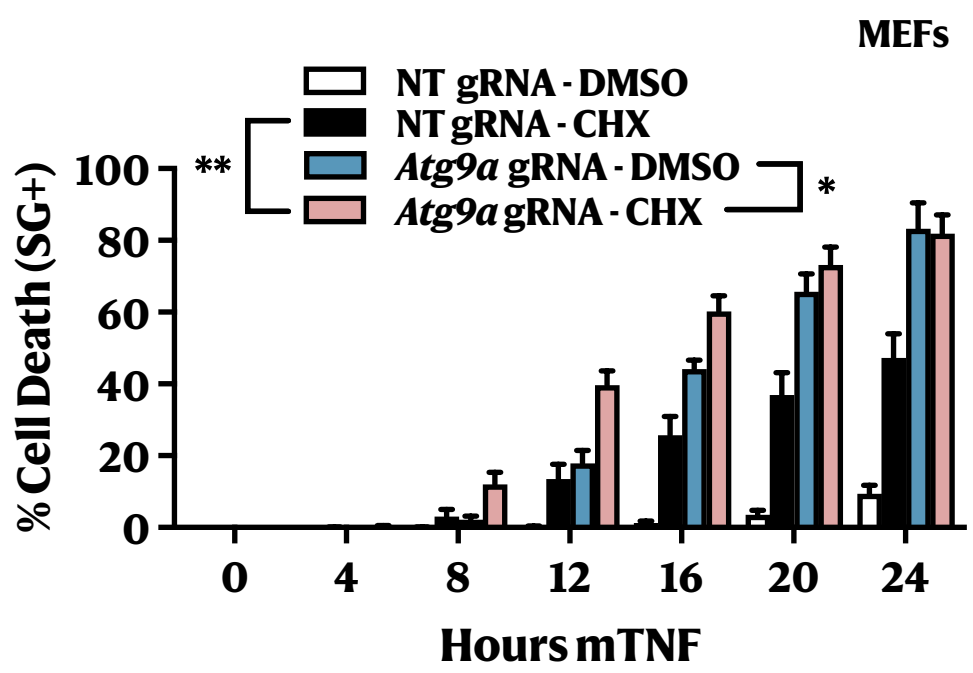

ATG9A prevents TNF cytotoxicity independently of translation. MEFs were electroporated with Cas9-RNPs targeting *Atg9a* or a non-targeting sequence (NT). Cells were pre-treated with CHX, as indicated, before stimulation with 20 ng/mL mTNF. Cell death was measured over time by SG positivity.

4. The additional experiments using CASP3/7-deficient cells and the accompanying analyses are appreciated and represent a step toward addressing the concern that the detected vesicles might originate from apoptotic cells. Nevertheless, the evidence provided remains incomplete. Rather than reporting average EV size distributions, the manuscript should present full particle size distributions under all relevant conditions, including WT cells, CASP3/7-deficient cells, and cells treated with a pan-caspase inhibitor, to more rigorously exclude contamination by vesicles derived from spontaneous or low-level cell death, which is common in cell culture. Moreover, the central concern remains that FADD is absent or barely detectable in EVs isolated by size exclusion chromatography, even following TNF stimulation. While the DGUC experiments are convincing in that FADD, CASP8, and RIPK1 (including modified forms) are detected in specific density fractions after TNF treatment (Fig. 4g), the manuscript would be strengthened by additional comparisons. In particular, given that both SEC and DGUC experiments were performed in the absence of CHX, it would be informative to include DGUC analyses in the presence of CHX and in the presence of a pan-caspase inhibitor, in both WT and CASP3/7-deficient cells. These controls would help exclude the possibility that apoptotic vesicles of exosome-like size contribute to the detected signal. Similarly, extending DGUC analyses to WT and RAB5-S34N MEFs, with and without TNF stimulation, would further strengthen the claim that EV-mediated detoxification of TNFR1 Complex II operates under the proposed conditions.

> We appreciate the reviewer's continued careful evaluation of the extracellular vesicle (EV) analyses and the concern regarding potential contamination by vesicles derived from apoptotic cells. We are convinced, however, that the revised manuscript already conclusively excluded this possibility.

First, we show that under the conditions used for EV isolation (6h TNF stimulation in WT cells), no detectable cytotoxicity is observed. This argues against significant spontaneous or TNF-induced apoptosis contributing to the EV pool under these conditions. Second, ultrastructural analysis by electron microscopy demonstrates that the isolated vesicles lack morphological features characteristic of apoptotic bodies, further supporting their non-apoptotic origin. Third, and most importantly, we generated CASP3/7-deficient cells, which are incapable of executing apoptosis downstream of TNF signaling, including under sensitizing conditions such as TNF + CHX, as demonstrated. EVs isolated from these cells display the same qualitative features as those obtained from WT cells, providing an indisputable genetic control to exclude contamination by apoptosis-derived vesicles.

With respect to the use of a pan-caspase inhibitor, we would like to emphasize that TNF stimulation in the presence of zVAD is known to redirect signaling toward necroptosis due to inhibition of CASP8. We show that exosomal disposal of TNFR1 Complex II selectively protects against TNF-induced apoptosis but does not counteract necroptosis. As necroptosis is a lytic form of cell death associated with membrane rupture and release of intracellular contents, EV isolation under these conditions would be particularly prone to confounding contamination and would therefore not provide a clean or interpretable control for the question at hand. For this reason, we instead opted for the generation and use of CASP3/7-deficient cells, which is the most experimentally sound approach to exclude apoptotic vesicle contamination.

Regarding the detection of FADD, CASP8, and RIPK1 in EVs, we demonstrate that EVs isolated by ultracentrifugation, size exclusion chromatography (SEC), and density-gradient ultracentrifugation (DGUC) yield consistent results. While DGUC provides high-resolution separation and was therefore used to establish the presence of intact TNFR1 Complex II components in specific density fractions, it is a highly labor-intensive and time-consuming method. SEC is a widely accepted and reliable EV isolation technique and was therefore employed for the majority of experiments once the nature of the vesicles had been established.

Extending DGUC analyses to all experimental conditions suggested by the reviewer (including CHX treatment, pan-caspase inhibition, multiple genetic backgrounds, and RAB5-S34N expression) is not realistic and would not increase the interpretability or robustness of the conclusions. Importantly, the key mechanistic claim - that TNFR1 Complex II components are selectively incorporated into bona fide EVs under non-apoptotic conditions - is already supported by convergent evidence from genetic, biochemical, and ultrastructural analyses.

5. Thank you, indeed TRADD was present. However, if TRADD originates from TNFR1 Complex II, one might expect it to display post-translational modifications, as reported in previous studies (e.g., Fullsack et al., doi:10.1038/s41419-019-1396-5; Micheau and Tschopp, Cell 2003). The absence of such modifications raises questions about the nature of the TRADD species detected in EVs. The authors may wish to consider testing additional TRADD antibodies to assess whether modified forms can be detected. Conversely, the newly presented data show post-translationally modified TRAF2 in EVs (Fig. 4q). It remains unclear to me whether this observation is expected or mechanistically consistent with the proposed model. Additional clarification or discussion would be helpful.

>We acknowledge that TRADD can undergo post-translational modification downstream of TNF sensing, as reported in previous studies. In our experiments, we detect TRADD in extracellular vesicles in a TNF-dependent manner, supporting its association with TNFR1-derived signaling complexes. The apparent absence of detectable post-translationally modified TRADD species in EVs may have several, non-mutually exclusive, explanations. First, post-translational modifications are known to be dynamic and context-dependent. We monitor the presence of TRADD after 6h of TNF stimulation, which is later than in the two refs mentioned by the reviewer. We also monitor TRADD in WT cells only exposed to TNF, which also differs from these other studies. Moreover, only a portion of TRADD is reported to be modified, and we may not be able to additionally detect this specific pool of TRADD in our settings due to the limited material. It is also conceivable that post-translational modifications on TRADD are removed prior to packaging TNFR1 Complex II into intralumenal vesicles.

Finally, we acknowledge the reviewer's suggestion that antibody specificity or sensitivity could influence the detection of modified TRADD species. Notably, a recent study (PMID: 41081715) using the same TRADD antibody similarly did not report modified TRADD species following TNFR1 Complex I or II immunoprecipitation. Importantly, we do not draw mechanistic conclusions based on the modification status of TRADD, but rather on its TNF-dependent recruitment to EVs together with other core components of TNFR1 Complex II. We therefore believe that testing additional TRADD antibodies is beyond the scope of our study, especially since the specificity of the antibody used in our study has already been verified.

With respect to TRAF2, the detection of post-translationally modified forms in EVs may be consistent with its previously reported TNF-mediated phosphorylation (PMID: 18981220, PMID: 21119000). The presence of modified TRAF2 in EVs therefore supports the notion that assembled and signaling-competent TNFR1-associated complexes are selectively incorporated into EVs.

6. While the technical effort required to extend EV profiling to FasL- or TRAIL-stimulated cells is appreciated, the rationale for excluding such comparisons is not entirely convincing. Although the reviewer fully recognizes the mechanistic differences between TNFR1 Complex II and DISC formation downstream of Fas or TRAIL receptors, it remains plausible that cells may deploy similar protective strategies to eliminate cytotoxic signaling complexes under suboptimal or limiting activation conditions. In this context, it would not be surprising if ESCRT inhibition also sensitized cells to FasL- or TRAIL-induced cell death. At minimum, this possibility should be discussed explicitly; ideally, limited experimental testing would further strengthen the manuscript.

> We agree with the reviewer that ESCRT inhibition could, in principle, also sensitize cells to FasL- or TRAIL-induced cell death. In such a scenario, however, the underlying mechanism would be fundamentally different from the one described in this study, as cytotoxicity would most likely arise from impaired receptor trafficking and prolonged accumulation of the receptor-bound death-inducing signaling complex (DISC) downstream of Fas or TRAIL receptors. As the reviewer also notes, this process is mechanistically distinct from TNF-induced apoptosis, which proceeds via the cytosolic, receptor-dissociated TNFR1 Complex II. Because the central aim of the present manuscript is to define a specific mechanism for ESCRT-dependent detoxification of TNFR1 Complex II, we consider experimental testing of FasL- or TRAIL-induced signaling to fall outside the scope of this study. Moreover, as other reviewers already found the discussion too lengthy, we decided not to further increase it by speculating on regulation of distinct cytokine signaling pathways.

7. While the authors note that time-zero TNFR1 immunoprecipitation controls are not routinely included in their experimental workflow, the inclusion of such controls would help exclude the possibility of steady-state differences in TNFR1 abundance or trafficking prior to TNF stimulation. This is particularly relevant given the observed alterations in MAPK/NF-κB signaling, TNFR1 levels, and RIPK1 ubiquitination in some experimental conditions (e.g., Fig. 2i; comparison of RAB5-WT and RAB5-S34N cells). Inclusion of a time-zero control would therefore strengthen confidence that the reported differences arise from TNF-induced signaling rather than pre-existing alterations.

> Comparative analysis of the steady-state abundance of surface TNFR1 is now provided in Extended Data Fig. 3e-g (see our answer to remark 2).

8. The authors in their rebbutal show a second immunoblot after TNF IP, which like the first one displays limited but consistent reduction of ubiquitinated RIPK1 in TSG101 KO cells. And contrary to their rebuttal, this clearly suggests that ESCORT I KO affects also complex I formation, potentially explaining the subtle differences observed in MAPK and NFkB signalling. One may also conclude from this observation that in TSG101 KO cells, the transition from CI to CII occurs faster than in WT cells, which would also fit the proposed working hypothesis.

> Defects in TNFR1 Complex I assembly (including reduced RIPK1 ubiquitination) typically result in RIPK1 activation (monitored by auto-phosphorylation on Ser166) and induction of RIPK1 kinase-dependent cell death. However, we formally demonstrated in the manuscript that deficiency in the ESCRT components TSG101, VPS28 and USP8 and expression of the RAB5 S34N mutant specifically sensitize cells to RIPK1 kinase-independent apoptosis (and do not induce pS166 RIPK1). These results therefore suggests that the subtle alterations highlighted by the reviewer do not represent biologically relevant changes in the context of cell death induction. Indeed, we also show that deficiency in TSG101, VPS28 and USP8 and expression of RAB5 S34N still sensitize cells to death when pre-treated with the translational inhibitor CHX, further supporting a cytoprotective function of these components outside of TNFR1 Complex I and independent of TNF-mediated NF-κB/MAPK signaling. Together with our other data, we can conclude that the pro-survival function of the tested components originates from their role in sequestering TNFR1 Complex II, and not from their potential function at the level of TNFR1 Complex I or TNFR1 trafficking.

9. Thanks for your answer. The clarification regarding the use of CHX during DGUC/SEC-based EV isolations is indeed important. Please see new comment (Point N°4). I would probably have generated a caspase-8 KO HT1080 cell line and re-introduced a Protease-deficient version of the caspase-8 to formely demonstrate that ILV-mediated secretion of complex II is occuring independent of caspase activation.

>The generation of CASP8 KO HT1080 cells followed by their reconstitution with an inactive version of CASP8 would require a substantial amount of extra work and would constitute an experimental set up of inferior strength compared with the CASP3/7 dKO approach we already employed (see our response to Remark 4 for details).

10. You are right, looking closer shows a feint band of FADD, yet there is no change whether the cells have been treated or not with TNF, arguing against your working hypothesis, as one may expect to see some more in stimulated cells, given that complex II is not supposed to be formed in the absence of TNF.

>We clearly showed that interaction between FADD and CASP8, representative of complex II assembly, only occurs upon TNF stimulation in extracellular vesicles (EVs), demonstrating a TNF-dependent extracellular export of active TNFR1 Complex II (which is completely in line with the proposed model). As previously acknowledged, the observation that non-interacting, inactive, FADD and CASP8 are also detected in EVs from unstimulated cells is interesting, but likely involves an independent targeting mechanism. As the amount of Complex II–associated FADD and CASP8 targeted to EVs may be limited compared with the pool of free FADD and CASP8 constitutively targeted to EVs, this may explain why the Complex II–associated fraction is difficult to detect.

We thank the editor and reviewers for their positive feedback and for the constructive comments on our study. In response, we have conducted several additional experiments to address all points raised by the reviewers. The revised manuscript now includes **41** new or adapted figure panels, some of which have been assembled into an entirely new figure. We believe that these new data substantially strengthen the conclusions of the study and improve the overall quality of the manuscript.

Please find below our point-by-point responses to the reviewers' remarks. New figure panels and textual revisions in the manuscript are highlighted in **blue**.

Reviewer #1

1. In order to clarify the cytoprotective function of USP8 in the context of TNF-induced complex II formation the authors would need to test the previously described (Jeong et al., Oncogene. 2017 Jan 26;36(4):458-470. doi: 10.1038/onc.2016.215) USP8 mediated regulation of cFLIP-L via its deubiquitylation, thereby protecting it from proteasomal degradation. Furthermore, it would be important to speculate on why the chemical inhibition of USP8 with DC-U4106 proves to be a stronger sensitizer compared to its genetic removal (Fig 1a and 1b).

*> We thank the reviewer for this valuable remark. It is correct that USP8 was previously reported to protect cells from TNF-induced death by promoting cFLIP-L stabilization. Our work does not dispute these earlier findings. Instead, we identify an additional protective function of USP8 in this pathway-namely, the ESCRT-dependent sequestration of cytosolic TNFR1 Complex II into ILVs. In support of this, we show that USP8 inhibition, like TSG101 inactivation, significantly reduces the endosomal localization of cleaved (Cl.) CASP8 puncta (**Fig. 4c-d, Extended Data Fig. 4j**), a readout that is independent of USP8's role in regulating cFLIP.*

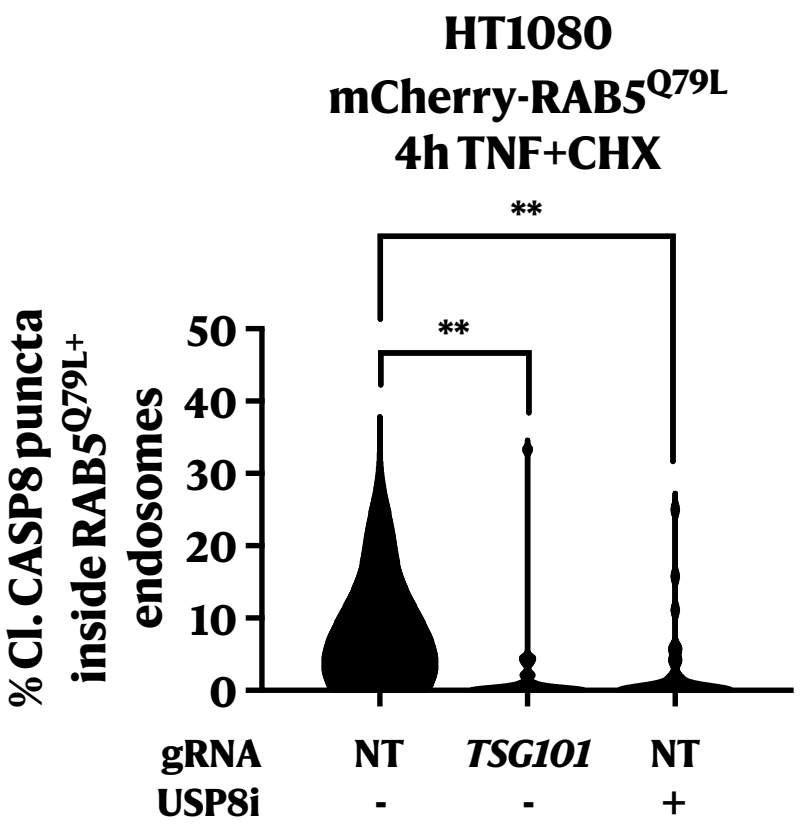

Extended Data Fig. 4d. HT1080 cells were transduced with an inducible expression vector encoding an mCherry-tagged version of the RAB5 Q79L mutant (RAB5^{Q79L}). Transduced cells were electroporated with Cas9-RNPs targeting *TSG101* or a non-targeting sequence (NT). Cells were treated with 2 μg/mL Doxycycline for 48h. Subsequently, cells were pre-treated with CHX and USP8i as indicated before stimulation with 1 μg/mL hTNF for 4h. Quantification of the percentage of Cl. CASP8 found inside mCherry^{+} endosomes was performed. Violin plots represent the distribution of the determined percentage over individual imaged cells. Statistical significance of the microscopy data was determined using a Kruskal-Wallis test followed by a Dunn's multiple comparisons test.

*> We agree, however, that the cytotoxicity assays in **Fig. 1a-b** cannot discriminate between these two protective functions of USP8. To disentangle them in this specific context, we additionally assessed TNF-induced cell death upon USP8 inactivation in the presence of the translation inhibitor cycloheximide (CHX). Since cFLIP is an NF-κB target gene whose cytoprotective effect depends on TNF-induced NF-κB–mediated upregulation, CHX is*

*routinely used to block its synthesis and abolish its inhibitory function on CASP8 activation. Importantly, our results show that USP8 inactivation further sensitizes cells to TNF cytotoxicity even when CHX is present (**Extended Data Fig. 1q, 1y**). These findings therefore suggest a cFLIP-independent protective role of USP8. Notably, the microscopy assay showing USP8-dependent targeting of TNFR1 Complex II into ILVs (**Fig. 4d, Extended Data Fig. 4j**) was also performed in the presence of CHX, further excluding any contribution of cFLIP to this readout. We have clarified this point and included the reference in the text: "A cytoprotective role of USP8 downstream of TNF sensing has previously been attributed to its ability to deubiquitinate cFLIP, thereby preventing its proteasomal degradation[38]. Our data now strongly support an additional function for USP8 in counteracting TNF cytotoxicity that operates independently of NF-κB-mediated cFLIP upregulation." – page 7, lines 7-12.*

ED Fig. 1q

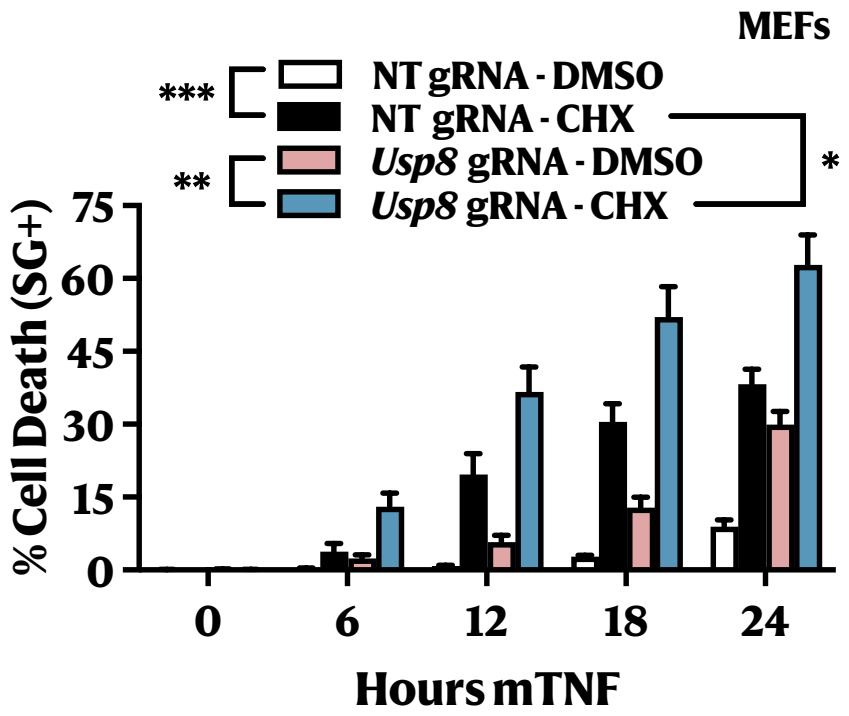

ED Fig. 1y

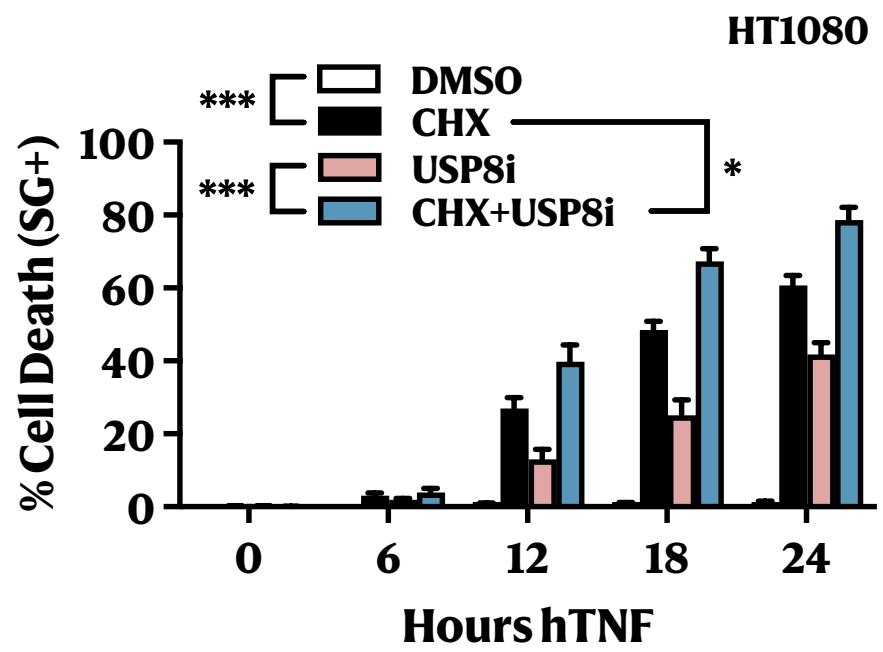

Extended Data (ED) Fig. 1q and 1y. ED Fig. 1q, MEFs electroporated with Cas9-RNPs targeting *Usp8* or NT were pre-treated with CHX as indicated before stimulation with 20 ng/mL mTNF. **ED Fig. 1y**, HT1080 cells were pre-treated with CHX and USP8i as indicated before stimulation with 20 ng/mL hTNF. Cell death was measured over time by SG positivity. Cell death experiments are presented as mean ± SEM of three independent experiments and statistical significance was determined using a two-way ANOVA. Significance between samples is indicated in the figure as follows: *p < 0.05; **p < 0.01; ***p < 0.001; ns non-significant.

> *Regarding the stronger sensitizing effect of the USP8 inhibitor DC-U4106 compared to CRISPR-mediated depletion of USP8, we believe this difference arises from the incomplete nature of the genetic knockout at the population level. CRISPR-Cas9 editing is inherently stochastic, and despite enrichment for gene-targeted cells, residual USP8-proficient cells are likely to remain within the population. These cells can partially mask the cytotoxic phenotype, leading to an apparently weaker effect compared to acute pharmacological inhibition, which uniformly suppresses USP8 catalytic activity across the entire culture. We therefore attribute the discrepancy primarily to this technical limitation.*

2. The protective role of ESCRT components on TNF-induced necroptosis should be further explored by treatment of the ESCRT-deficient KO lines with TNF together with zVAD. This would be important since the same methodology is used for inhibition of ESCRT-machinery activation with Ca2+ chelator on Extended Data Figure 1k-l.

*> The cytoprotective role of ESCRT components in TNF-induced necroptosis is well established and has been attributed to ESCRT-mediated shedding of damaged plasma membrane regions (Gong et al., Cell 2017). The experiments suggested by the reviewer were already addressed in that study, where ESCRT deficiency was shown to sensitize cells to TNF in the presence of zVAD (Fig. 4, Fig. S5). In our work, we specifically used the Ca^{2+} chelator BAPTA-AM to block this ESCRT-dependent plasma membrane repair function without interfering with the other roles of the ESCRTs. Our data show that BAPTA-AM sensitizes cells to TNF only when combined with zVAD, whereas ESCRT deficiency sensitizes cells even in the absence of caspase inhibition. This distinction demonstrates that ESCRT components also act downstream of TNFR1 activation via a mechanism that is independent of plasma membrane repair. We rephrased this section in the revised manuscript for improved clarity: "We further excluded a contribution of ESCRT-mediated plasma membrane repair by interfering with extracellular calcium influx, which is required to trigger ESCRT recruitment to sites of membrane damage. The entry of extracellular Ca^{2+} through membrane pores was shown to activate the ESCRT-dependent outward budding and shedding of damaged membrane fragments, thereby restoring plasma membrane integrity[32–35]. In line with previous litterature[32], chelation of Ca^{2+} with BAPTA-AM, preventing ESCRT recruitment and activation at the plasma membrane, markedly sensitized cells to TNF-induced necroptosis obtained by pre-treatment with the pan-caspase inhibitor zVAD-fmk (zVAD) (**Extended Data Fig. 1m-n**). However, BAPTA-AM did not mimic ESCRT deficiency in the absence of zVAD, thereby excluding plasma membrane repair as the primary cytoprotective function of the ESCRT machinery downstream of TNF sensing." – page 6, lines 10-21.*

3. The addition of cycloheximide was tested for MEFs lacking different components of the ESCRT machinery and for HT1080 under USP8 inhibition (Fig 1h; Extended Data Fig. 1m-o, w). Here, the authors show bar plots with the direct comparison of ESCRT-proficient and -deficient cells under the challenge of TNF+CHX; however, there is lack of the direct comparison with TNF treatment alone as a control. Only with this comparison it is possible to understand if the pro-survival effects are dependent or not on gene activation. By comparing Fig 1e DMSO and 1h on Tsg101 KO MEFs it appears that the sensitization to TNF-induced cell death is lost; it would be important to speculate on why this is the case as this would further strengthen the claim of a new checkpoint.

*> We agree with the reviewer that a direct comparison with TNF treatment alone is essential for full interpretation. We have consequently included these additional results to the cell death graphs in the revised version of the manuscript (**Fig. 1h**, **Extended Data Fig. 1p-q**, **y**). The new graphs show that either ESCRT component deficiency or CHX treatment is sufficient to shift the TNF response toward cell death, and that the combination of both conditions results in even greater cytotoxicity than either treatment alone. This demonstrates that the NF-κB checkpoint and the newly discovered ESCRT-dependent checkpoint operate independently of one another. Together, these results strengthen the conclusion that ESCRT-mediated sequestration of TNFR1 Complex II into ILVs constitutes a distinct cell death checkpoint within the TNF pathway.*

Fig. 1h

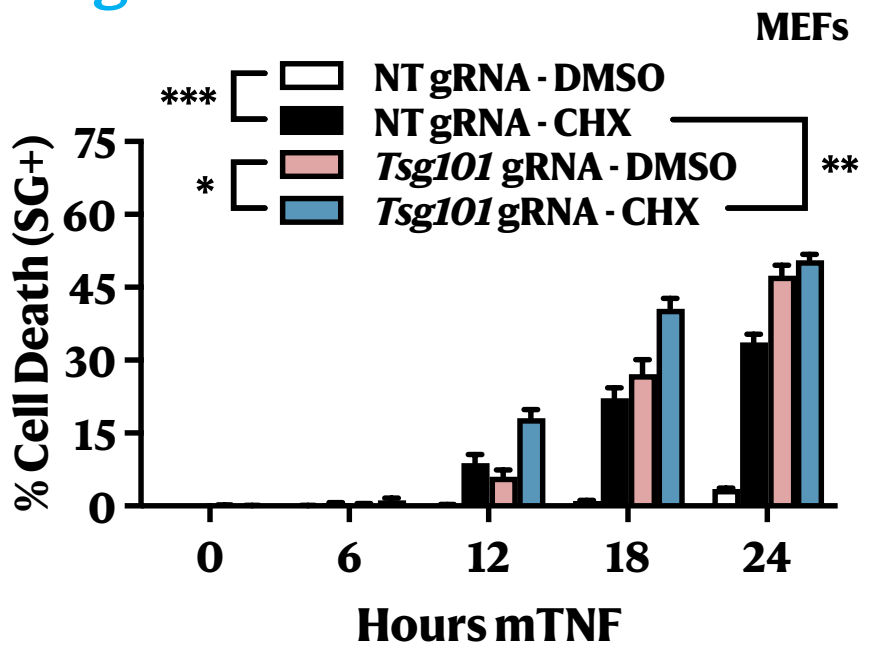

ED Fig. 1p

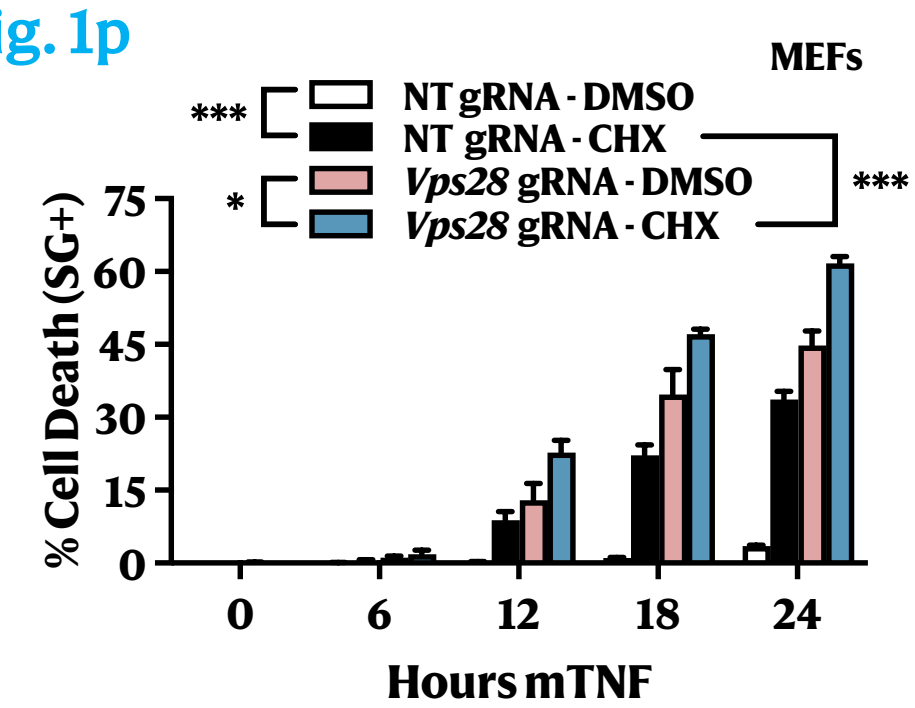

ED Fig. 1q

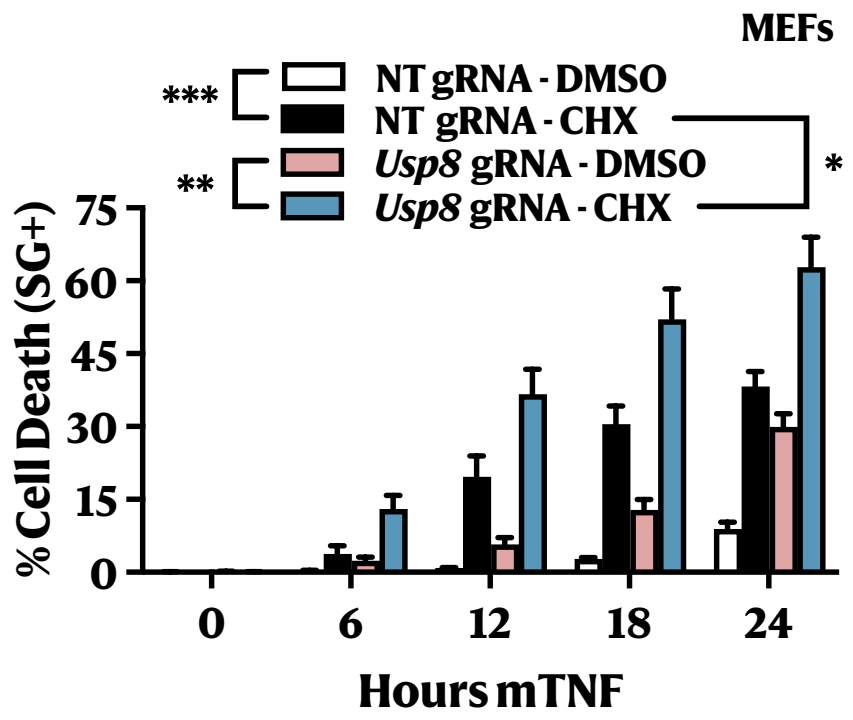

ED Fig. 1y

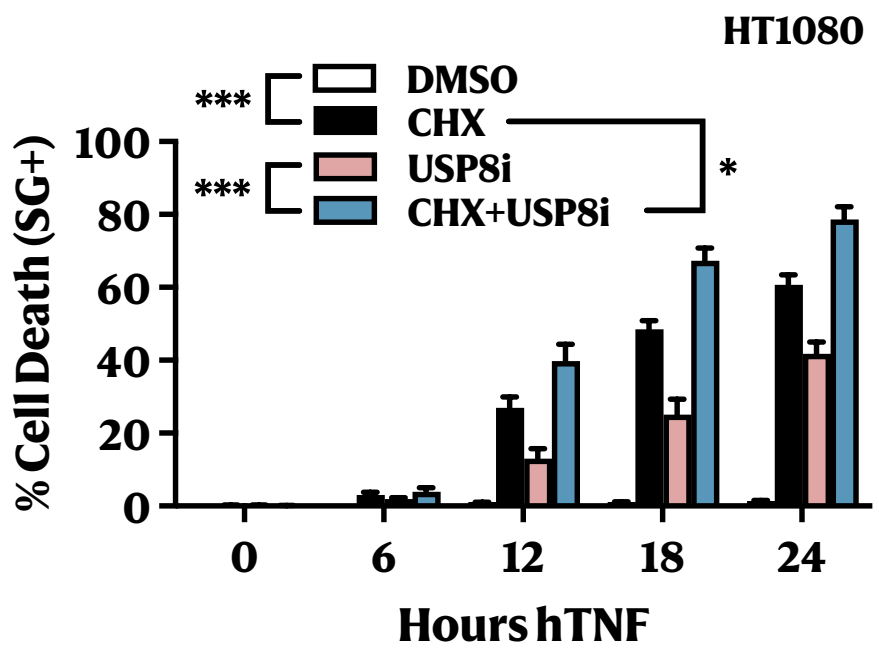

Fig. 1h, Extended Data (ED) Fig. 1p-q, y.and Fig. 2J. Fig. 1h, MEFs were transduced with an inducible expression vector encoding Cas9 (hereafter referred to as iCas9 MEFs) and subsequently transduced with inducible expression vectors encoding sgRNAs targeting *Tsg101* or NT. Cells were treated with 1 µg/mL Doxycycline for 48h and subsequently expanded for 72h. Cells were pre-treated with CHX, as indicated, before stimulation with 20 ng/mL mTNF. Cell death was measured over time by SG positivity. **ED Fig. 1p-q**, MEFs were transduced with LentiCRISPRv2 constructs or electroporated with Cas9-RNPs targeting the indicated genes or a non-targeting sequence (NT). Cells were pre-treated with CHX, as indicated, before stimulation with 20 ng/mL mTNF. Cell death was measured over time by SG positivity. **ED Fig. 1y**, HT1080 cells were pre-treated with CHX and USP8i, as indicated, before stimulation with 20 ng/mL hTNF. Cell death was measured over time by SG positivity.

4. In Figure 3 the authors aim to demonstrate the presence of TNF complex II in intralumenal vesicles. The use of immunofluorescence for assessment of localization of cleaved caspase 8 should be complemented with the detection of other components of complex II, furthermore, immunostaining of full-length caspase 8 would be essential to complement Fig3 f-g, where full length caspase 8 is found in extracellular vesicles, both with and without TNF stimulation. Similarly, Caspase 8 immunoprecipitation in Figure 3e should include markers of intralumenal vesicles such as Rab5 to corroborate the findings from the immunofluorescence panels.

*> To complement the extracellular vesicle (EV) data and further strengthen the immunofluorescence-based evidence, we followed the reviewer's suggestion and attempted additional stainings of endogenous FADD and RIPK1 (other components of TNFR1 Complex II). Unfortunately, available commercial antibodies did not yield reliable immunofluorescence signals (Data not shown). To overcome this limitation, we generated MEFs expressing GFP-tagged FADD (Fadd$^{-/-}$ MEFs transduced with a Doxycycline-inducible FADD-GFP construct). Of all prototypical TNFR1 Complex II core components, CASP8 and FADD are the best suited for the specific monitoring of this complex by microscopy as they are absent from TNFR1 Complex I (unlike TRADD and RIPK1). FADD-GFP appeared as a diffuse signal in untreated MEFs, while it formed distinct puncta 4h after TNF stimulation, consistent with TNFR1 Complex II assembly (**Extended Data Fig. 4g**). Importantly, a subset of these puncta colocalized with the early endosome marker EEA1 (**Extended Data Fig. 4h**). These findings therefore support the transfer of an additional component of TNFR1 Complex II into the endosomal system prior to its exosomal release. These additional data have been included in the revised manuscript (**Extended Data Fig. 4g-h**).*

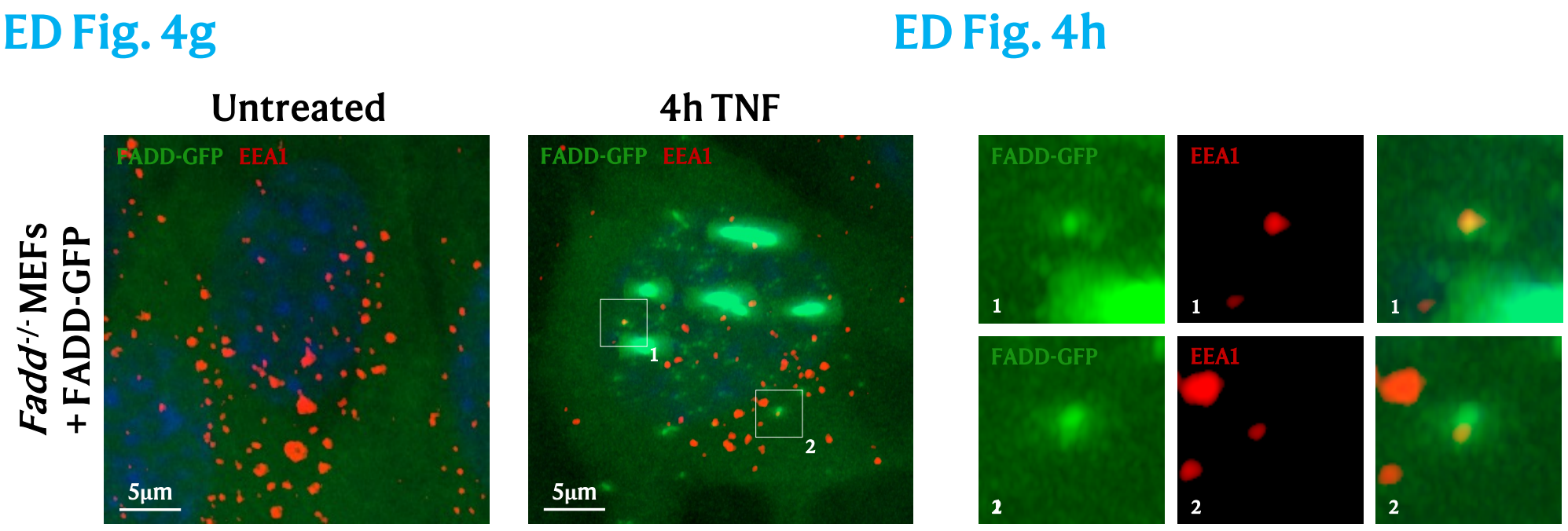

Extended Data (ED) Fig. 4g-h. *Fadd$^{-/-}$* MEFs were reconstituted with an inducible expression vector encoding GFP-tagged FADD. Cells were treated with 100 ng/mL Doxycycline for 24h. Subsequently, cells were stimulated with 20 ng/mL mTNF for 4h. Cells were then stained for the early endosome marker EEA1. **ED Fig. 4g**, Representative images of EEA1 immunostainings are shown. **ED Fig. 4h**, Single and merged channel images of the zoomed subpanels of cells treated with TNF for 4h are shown.

*> Regarding the presence of full-length CASP8 and unbound FADD in EVs isolated from unstimulated cells (**Fig. 4g-h**), we agree that this is an interesting observation and likely reflects a biologically meaningful—though mechanistically distinct—process. However, the focus of the present manuscript is specifically on the regulation and fate of TNFR1 Complex II, which is only detected upon TNF stimulation (**Fig. 4g-h**). A detailed investigation of the mechanisms governing basal incorporation of unbound FADD or CASP8 into EVs would therefore extend beyond the scope of this study.*

> We thank the reviewer for the suggestion to include ILV markers to the CASP8 immunoprecipitations. Unfortunately, spatial relationship between TNFR1 Complex II and ILV markers (such as RAB5 or EEA1) cannot be expected from a co-immunoprecipitation. Indeed, the detergent contained in the lysis buffer disrupts endosomes and their ILVs, releasing TNFR1 Complex II into the cytosolic fraction.

5. Later, the authors explore the fate of the vesicles originated by ESCRT-dependent endosomal micro-autophagy. As mentioned in the discussion "lysosomal degradation is a possible convergence point of both macro-autophagy and eMI", as such, the localization of complex II onto the lysosomal compartment should be evaluated by immunofluorescence by co-localization of cleaved caspase 8 and known lysosomal markers such as LAMP1/LAMP2; this should be performed under Atg9a-proficient and -deficient background. Another possible approach could be the immunoprecipitation of LAMP1 as used in reference 12 of the present manuscript, previous work by the same group.

*> The sequestration of cargo into intralumenal vesicles (ILVs) is a decisive event that can direct cargo toward either lysosomal degradation or extracellular release in exosomes. In the original submission, we provided strong evidence that ILV sequestration of TNFR1 Complex II leads to its extracellular release via exosomes. To address the reviewer's question of whether this process also contributes to lysosomal degradation of TNFR1 Complex II, we followed the suggestion to compare the effects of macro-autophagy versus eMI inhibition on the co-localization of cleaved (Cl.) CASP8 puncta with the lysosome marker LAMP1. These experiments were performed in the presence of the protease inhibitors PepA/E64D to prevent lysosomal degradation and thereby allow accurate assessment of co-localization. Consistent with our previous work demonstrating that LC3-independent macro-autophagy mediates lysosomal degradation of TNFR1 Complex II, we found that ATG9A deficiency significantly reduced the proportion of LAMP1$^+$ Cl. CASP8 puncta. In contrast, ESCRT inhibition with the USP8 inhibitor (USP8i) did not alter lysosomal localization of Cl. CASP8, indicating that ESCRT-dependent ILV formation does not contribute to the targeting TNFR1 Complex II to lysosomes (**Fig. 4e, Extended Data Fig. 4k**). Supporting these conclusions, we further observed that blocking all lysosomal fusion events with Bafilomycin A1 (BafA1) markedly reduced the lysosomal localization of Cl. CASP8 puncta, whereas selective inhibition of Late Endosome-lysosome fusion through expression of the dominant-negative RAB7^{T22N} mutant had no such effect (**Fig. 4f, Extended Data Fig. 4l**).*

*> Together, these findings indicate that the ESCRT-dependent sequestration of TNFR1 Complex II into ILVs primarily serves to mediate its extracellular release via exosomes, rather than targeting the complex for lysosomal degradation. These important new findings have been added to and are discussed in the revised manuscript (**Fig. 4e-f** and **Extended Data Fig. 4k-l**). We have additionally adapted the title of the manuscript to better reflect the nature of the newly identified process: "Exosomal Export of TNFR1 Complex II Prevents TNF-Induced Cytotoxicity".*

ED Fig. 4k

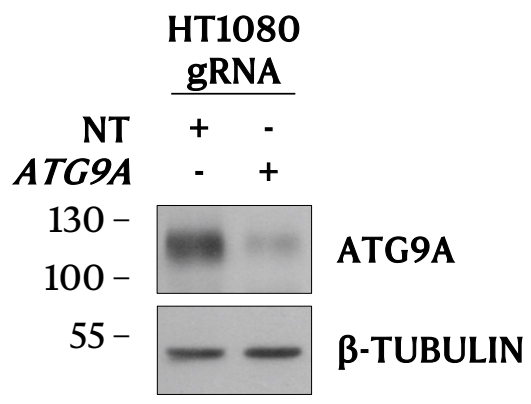

Fig. 4e

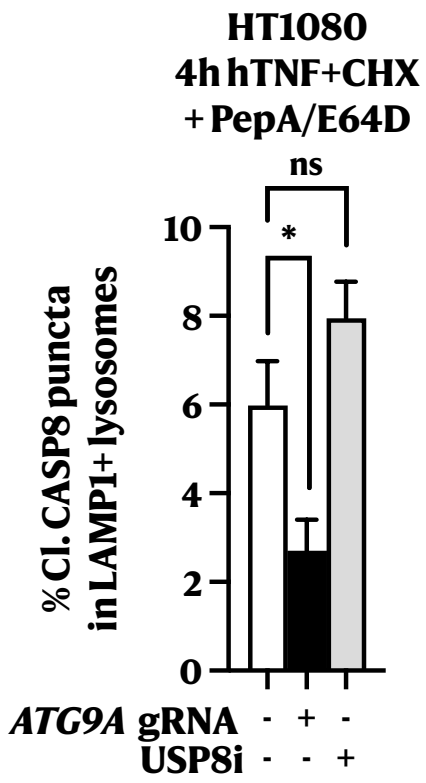

ED Fig. 4l

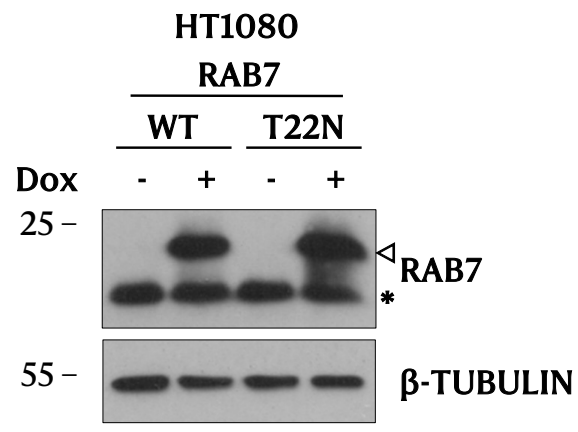

Fig. 4f

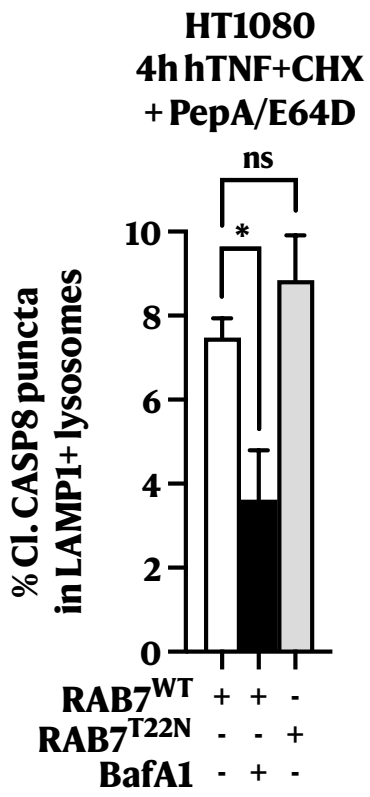

Extended Data (ED) Fig. 4k-l and Fig. 4e-f. ED Fig. 4k and Fig. 4e, HT1080 cells were electroporated with Cas9-RNPs targeting *ATG9A* or a non-targeting sequence (NT). **ED Fig. 4k**, Knockout efficiency was validated by immunoblot. **Fig. 4e**, Cells were pre-treated or not with CHX, USP8i and PepstatinA/E64D, as indicated, before stimulation with 2 µg/mL hTNF for 4h. Cells were then stained for the Cl. CASP8 and the lysosome marker LAMP1. The percentage of Cl. CASP8 puncta that colocalized with $LAMP1^{+}$ lysosomes was quantified and the average across images was determined. **ED Fig. 4l and Fig. 4f**, HT1080 cells were transduced with inducible expression vectors encoding either 3xHA-tagged wild-type (WT) RAB7, or the dominant-negative RAB7 T22N mutants ($RAB7^{T22N}$). Cells were treated with 2 µg/mL Doxycycline for 48h. **ED Fig. 4l**, Protein levels were determined by immunoblot. The endogenously expressed RAB7 and the ectopically expressed 3xHA-tagged RAB7 are indicated by (*) and (◁) respectively. **Fig. 4f**, Cells were pre-treated or not with CHX, BafA1 and PepstatinA/E64D, as indicated, before stimulation with 2 µg/mL hTNF for 4h. Cells were then stained for the Cl. CASP8 and the lysosome marker LAMP1. The percentage of Cl. CASP8 puncta that colocalized with $LAMP1^{+}$ lysosomes was quantified and the average across images was determined. Statistical significance was determined using a one-way ANOVA followed by a Dunn's multiple comparisons test. Significance between samples is indicated in the figure as follows: *p < 0.05; **p < 0.01; ***p < 0.001; ns non-significant.

6. In Figure 4, a model is proposed where M1-Ub RIPK1 is proposed as a scaffold for TAXBP1 promoting its transfer to endosomes. However, in cells that were not challenged with TNF, caspase 8 was found in extracellular vesicles (Figure 3f-g) without the presence of RIPK1, TAXBP1 or M1-ubiquitin. It would be important to understand how this is regulated and if Caspase 8 could be the initial substrate of eMI cargo selection.

*> We agree with the reviewer that the presence of FADD and CASP8 in extracellular vesicles (EVs) from unstimulated cells is intriguing (**Fig. 4g**). However, the fact that these proteins do not co-immunoprecipitate in these conditions suggests that they are not transferred as part of a pre-assembled complex (**Fig. 4h**). They may instead be recruited independently or reflect low-level stochastic sampling of cytosolic proteins.*

> We respectfully disagree with the reviewer's suggestion that CASP8 or FADD could function as the initiating substrate driving selective targeting of TNFR1 Complex II into ILVs. If this were the case, loss of linear ubiquitin chains, RIPK1, or TAX1BP1 should not impede the endosomal targeting of cleaved (Cl.) CASP8 - particularly given that its cytosolic abundance is increased under these conditions. In clear contrast, our results show that deficiency in any of these components markedly impairs the transfer of Cl. CASP8 into endosomes. Furthermore, the requirement for TAX1BP1 is consistent with previous studies demonstrating its role as an autophagy receptor in endosomal micro-autophagy, including the targeting of ferritin (Goodwin et al., Cell Rep. 2017; Ohshima et al., J. Cell Biol. 2022). Together, these findings support a model in which ubiquitin-dependent recognition - rather

than CASP8 or FADD themselves - governs the selective loading of TNFR1 Complex II into ILVs.

> These observations also strongly suggest that the basal transfer of CASP8 and FADD into EVs occurs via a mechanism distinct from the ESCRT-dependent sequestration of TNFR1 Complex II that we describe. As the focus of the present work is specifically on the TNF-induced cytotoxic complex, we believe that dissecting the steady-state release of CASP8 or FADD - while interesting - is beyond the scope of this manuscript.

7. On the contrary, in previous work not mentioned in this manuscript (Wu et al., Cell Res. 2023 Nov;33(11):851-866. doi: 10.1038/s41422-023-00859-3), and supporting a role for RIPK1 as a potential scaffold for eMI initiation, HSC70 has been reported to suppress necroptosis by specifically recognizing RHIM-containing proteins. In this report this is attributed to the ATPase activity of HSC70 but does not exclude the recognition of RIPK1 RHIM domain through a hydrophobic hexapeptide motif. This could be explored by reconstitution of RIPK1 KO MEFs with a RHIM mutant version of RIPK1 or any equivalent strategies.

>We thank the reviewer for this excellent remark. It was indeed recently reported that HSC70 suppresses necroptosis by binding RHIM-containing proteins and by acting as an amyloidase on RHIM oligomers in an ATPase-dependent manner. In contrast, during physiological TNF signaling, RHIM oligomers do not form, and our data indicate that HSC70 acts independently of its ATPase activity. Nevertheless, we agree that HSC70 could, in principle, engage TNFR1 Complex II through a related interaction with the RIPK1 RHIM domain. Wu et al. identified methionine 547 (M547) as a key determinant of the HSC70-RIPK1 interaction, since the RIPK1 M547G mutant showed substantially reduced binding. To assess whether such an interaction contributes to the detoxification of TNFR1 Complex II in our system, we generated the equivalent mutation in mouse RIPK1 (M536G) and reconstituted RIPK1-deficient MEFs with this mutant, as suggested by the reviewer. We then evaluated its effect on TNF-induced cell death. Despite the predicted disruption of HSC70 binding, the RIPK1^{M536G} mutant did not sensitize cells to TNF cytotoxicity, indicating that this interaction is unlikely to be essential for the ESCRT-HSC70-dependent targeting of TNFR1 Complex II. These new results have been added to and are discussed in the revised manuscript (Extended Data Fig. 5m-n).

ED Fig. 5m **ED Fig. 5n**

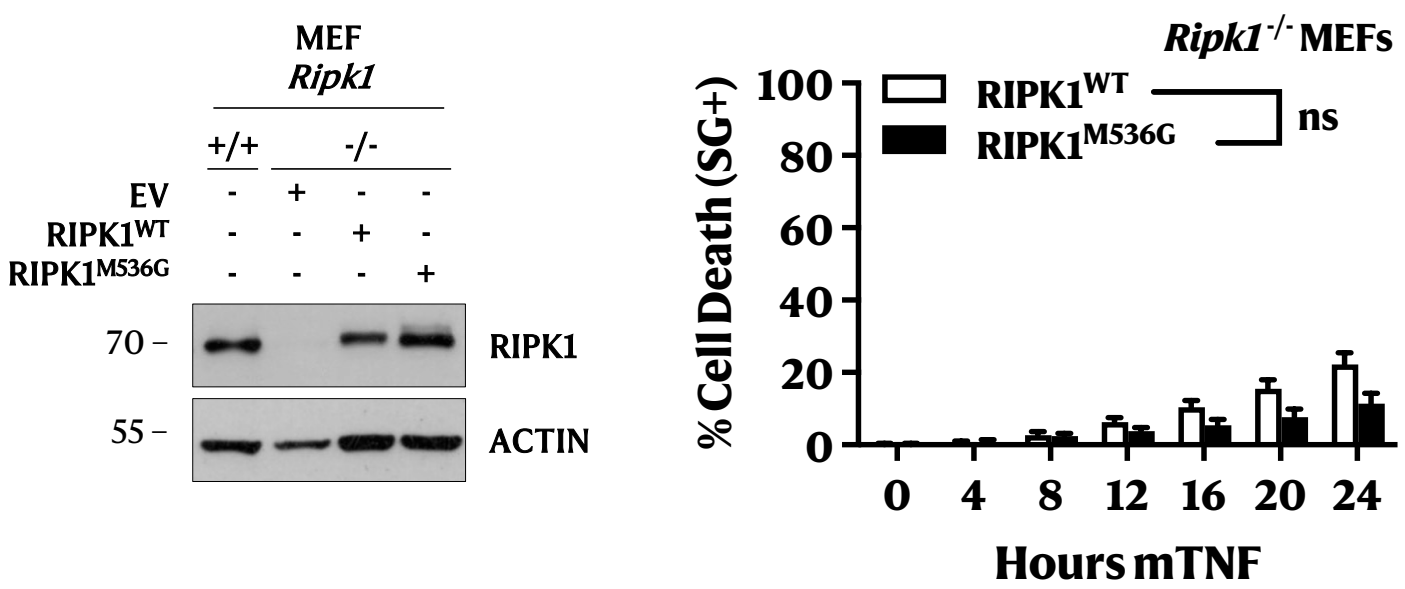

Extended Data Fig. 5m-n. *Ripk1*$^{-/-}$ MEFs were reconstituted with inducible expression vector encoding either wild-type RIPK1 (RIPK1^{WT}), the M536G RIPK1 mutant (RIPK1^{M536G}) or an empty vector (EV). Reconstituted cells were treated with 100 ng/mL Doxycycline for 24h. **ED Fig. 5m**, Reconstitution was validated and compared to *Ripk1*$^{+/+}$ MEFs via immunoblotting. **ED Fig. 5n**, Cells were treated with 20 ng/mL mTNF and cell death was measured over time by SytoxGreen (SG) positivity. Cell death experiments are presented as mean ± SEM of three independent experiments and statistical

significance was determined using a two-way ANOVA. Significance between samples is indicated in the figure as follows: *$p < 0.05$; **$p < 0.01$; ***$p < 0.001$; ns non-significant.

Reviewer #2

1. Since the effort is on EV formation upon MVB formation, it has to be questioned why the process is called Microautophagy. Simple 'export' rather than degradation. Thus, unless degradation is shown well, this is not eMI in the strict sense. It is not addressed here if true eMI actually also contributes to desensitization: is the complex stabilized in purified late endosomes upon inhibition of proteases? Perhaps in conjunction with prevention of exosome secretion? This would be required for the exact mechanism, although the authors show that protection from apoptosis is already achieved by sequestration of complex II from the cytoplasm.

> As a similar remark was raised by Reviewer #1, we invite Reviewer #2 to go through the answer provided to point 5 of Reviewer #1.

> Our new results indicate that ESCRT-dependent sequestration of TNFR1 Complex II into ILVs primarily serves to mediate its extracellular release via exosomes, rather than targeting the complex for lysosomal degradation. As suggested by the reviewer, we have adapted the title of the revised manuscript to better reflect the nature of the newly identified process: "Exosomal Export of TNFR1 Complex II Prevents TNF-Induced Cytotoxicity".

2. P8 Line 18 statement about effects or Rab variants on eMI degradation: There is a fast eMI that is starvation inducible and a slower one that is not. In addition to lacking a reference, it needs to be made sure that this statement is true in general.

> We thank the reviewer for this insightful comment. Indeed, both a constitutive form of eMI and a starvation-induced, accelerated form of eMI have been described (Mejlvang et al., J. Cell Biol., 2018; Olsvik et al., Autophagy, 2019). However, we can confidently exclude involvement of a starvation-induced pathway in our system, as all experiments in the manuscript were conducted under nutrient-replete conditions, and no starvation cues were applied. Moreover, the starvation-induced eMI pathway has been reported to operate independently of ESCRT-I components (TSG101, VPS28) and HSC70 - whereas our results clearly show that these factors are essential for targeting TNFR1 Complex II into endosomes. This argues strongly against participation of the starvation-responsive branch of eMI.

> We agree with the reviewer that the original manuscript did not directly demonstrate that the dominant-negative RAB mutants interfere with eMI. In response, we attempted to monitor two established eMI read-outs - the turnover of ferritin (induced by DFO) and of autophagy receptors (induced by starvation) - in ATG9A-deficient MEFs to eliminate macro-autophagy. However, unlike previous reports, we were unable to detect robust eMI-dependent turnover of these cargoes (Data not shown), preventing us from using these assays to assess the impact of the RAB mutants. Due to time and experimental limitations, we therefore cannot directly confirm whether these mutants block eMI in our hands. Nonetheless, the dominant-negative RAB variants used here are extensively characterized in the literature and routinely applied to disrupt specific steps of the endosomal pathway that are essential for eMI - namely ILV formation ($RAB5^{S34N}$), MVB maturation ($RAB5^{Q79L}$), and lysosomal fusion ($RAB7^{T22N}$). To avoid overstating their specificity, we have carefully rephrased the relevant sentence in the revised manuscript to reflect this nuance: "Thus, if the ESCRT-dependent formation of ILVs at the endosomal membrane is required to prevent TNF cytotoxicity, only $RAB5^{S34N}$ is expected to switch the TNF response to death, as the formation

*of ILVs is unaffected in the other RAB mutants (**Fig. 2a**). Accordingly, we found that while ectopic expression of $RAB5^{Q79L}$ and $RAB7^{T22N}$ had no impact on TNF cytotoxicity, cells expressing $RAB5^{S34N}$ died in response to TNF sensing (**Fig. 2b-c** and **Extended Data Fig. 2a**)."- Page 8, lines 14-19.*

> Importantly, new data added to the revised manuscript demonstrate that the ESCRT-dependent sequestration of TNFR1 Complex II into ILVs primarily mediates its extracellular release rather than its lysosomal degradation. Consequently, this process no longer qualifies as eMI in the strict sense. In light of this, we hope the reviewer agrees that assessing the eMI-dependent turnover of canonical cargoes using the dominant-negative RAB mutants is therefore no longer essential for the interpretation of our study.

3. P9 Line 10...: the vps34 inhibitor argument about MA not being involved (Fig. 2K, not 1K) makes only sense if it shown that RabS34N itself does not affect MA (as it messes up the endosomal system).

*> We agree with the reviewer's remark. To formally test this, we monitored LC3 lipidation under both nutrient-replete and starvation conditions and found that expression of $RAB5^{S34N}$ did not impair macro-autophagy. These new results demonstrate that the sensitizing effect of $RAB5^{S34N}$ is independent of macro-autophagy and is instead attributable to its inhibition of ILV formation. We have added these new data to the revised manuscript (**Extended Data Fig. 2c-e**).*

ED Fig. 2c **ED Fig. 2d** **ED Fig. 2e**

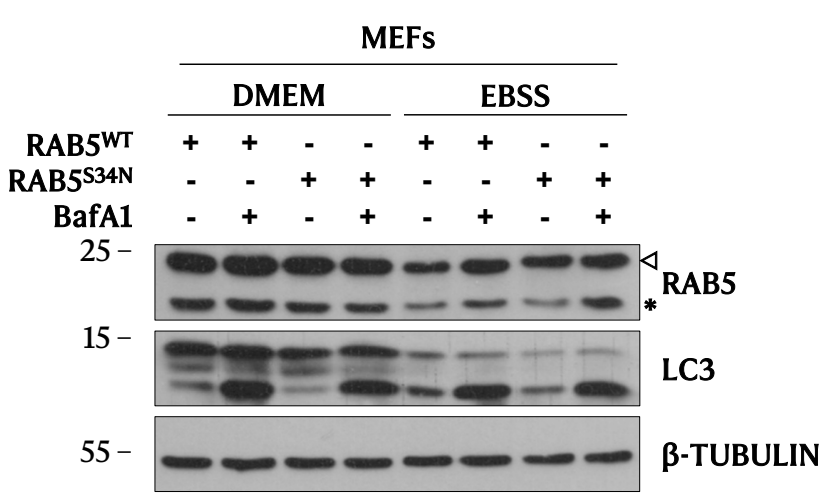

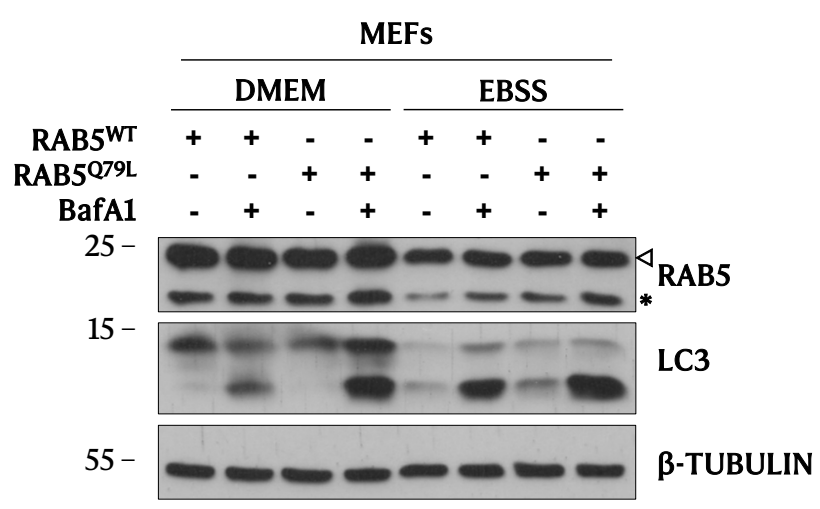

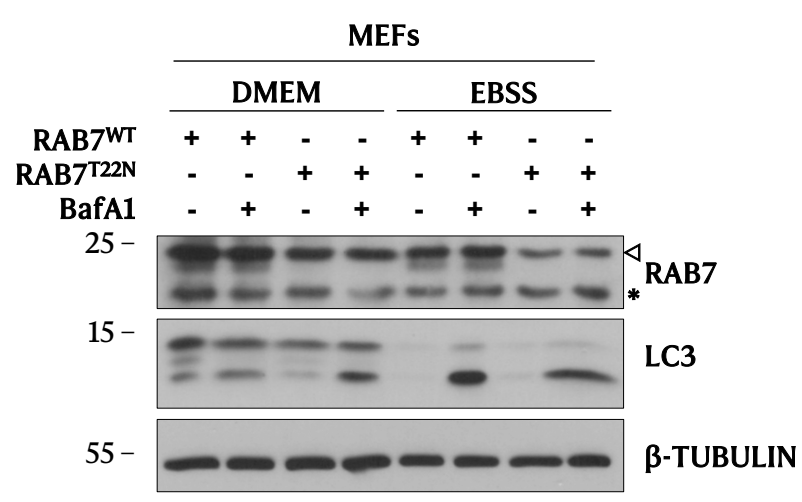

Extended Data (ED) Fig. 2c-e. MEFs were transduced with inducible expression vectors encoding either 3xHA-tagged wild-type (WT) RAB5 or RAB7, or the dominant-negative mutants $RAB5^{S34N}$(**ED Fig. 2c**), $RAB5^{Q79L}$(**ED Fig. 2d**), and $RAB7^{T22N}$(**ED Fig. 2e**). Cells were treated, or not, with 2 µg/mL Doxycycline (Dox) for 48h. Cells were subsequently treated with EBSS for 4h in the presence or absence of BafA1. Protein levels were determined by immunoblot.

4. P14 Line 22...: While it is clear that the bacterial toxins that inhibit ESCRT components sensitize for death, the interpretation that this effect is lost upon treatment with the ESCRT inhibitor USP8i is flawed and needs to be revised: both show the same effect that at one point is saturated. A geneticist would argue that the factors act in the same pathway. More telling would be a test for synergy using low doses of the inhibitor with sub-effect expression level of the toxins.

*>We agree with the reviewer that the interpretation of these results was not correctly phrased in this sentence. We have therefore rephrased it to increase clarity: "This notion was further supported by the fact that SopB and EsxG-H have no additional sensitizing effect when the ESCRT machinery is already inhibited by pre-treatment with USP8i (**Fig. 6e** and **Extended Data Fig. 6h**)."- Page 17, lines 29-31.*

5. Discussion why ESCRT II lack has no effect: one of the proposed reasons (redundancy) is testable. ESCRT II is used by all forms of eMI described in mammals and flies to the best knowledge of this reviewer.

>We thank the reviewer for raising this important point. As suggested, we considered the possibility that the absence of a phenotype in our ALIX- or ESCRT-II–deficient cells could reflect functional redundancy between these components. To directly address this, we generated cells lacking both ALIX and the ESCRT-II subunit VPS36. When challenged with TNF, these double-knockout cells did not exhibit increased cytotoxicity compared with wild-type or single-knockout controls. These results formally exclude a requirement for ALIX or ESCRT-II in directing TNFR1 Complex II into ILVs. These new data have been incorporated into the revised manuscript (Extended Data Fig. 1b-c).

ED Fig. 1b

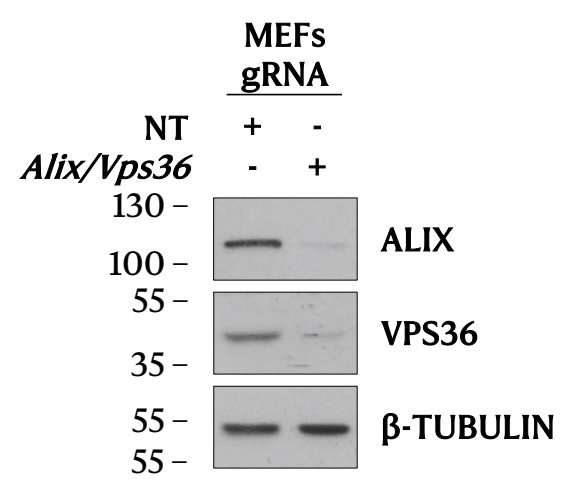

ED Fig. 1c

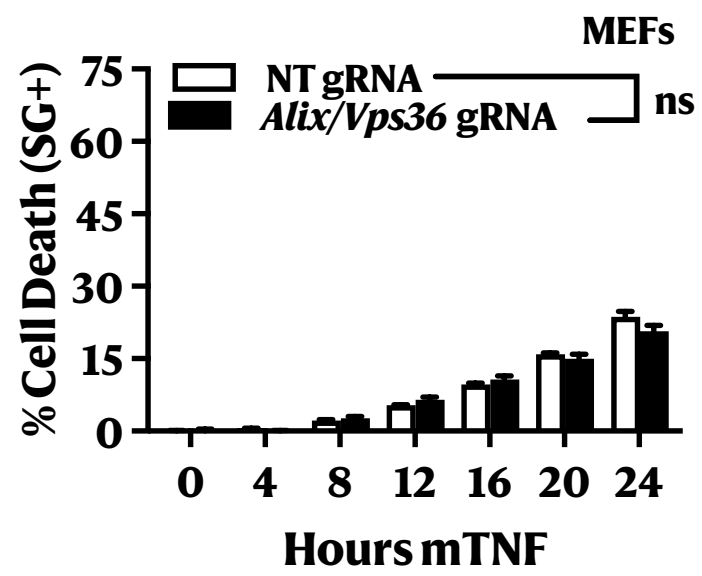

Extended Data (ED) Fig. 1b-c. MEFs were electroporated with Cas9-RNPs targeting *Alix* and *Vps36* or a non-targeting sequence (NT). **ED Fig. 1b**, Knockout efficiency was validated by immunoblot. **ED Fig. 1c**, Cells were stimulated with 20 ng/mL mTNF and cell death was measured over time by SytoxGreen (SG) positivity. Cell death experiments are presented as mean ± SEM of three independent experiments and statistical significance was determined using a two-way ANOVA, ns = non-significant.

> In line with our findings, a study published after our initial submission (Men et al., J Cell Biol., 2025) reported that tau aggregates are targeted into ILVs independently of ESCRT-II. Instead, PTPN23 was identified as the essential factor bridging ESCRT-I and ESCRT-III. These findings are fully consistent with our results and supports a model in which PTPN23, rather than ESCRT-II or ALIX, mediates TNFR1 Complex II sequestration during ESCRT-dependent detoxification. We have adapted our discussion to include a citation to this recent study.

6. Minor points:

- **Fig 1F: no lambda PPase in figure (but in legend). It would also be helpful to systematically mention the cells used (e.g. MEFs or not e.g. in Fig 1G; Fig S3 H, I).**
- **Fig S3 C: resolution not good enough to see ILVs in what is shown as MVBs.**
- **Endosomal localization of components: as these are enlarged MVBs this should be noted and they should be called something along 'Rab5S34N-endosomes'.**

> We thank the reviewer for these constructive remarks. We have adapted the manuscript accordingly.

Reviewer #3

1. It is not yet clear whether the observed effects on TNF-ICD are directly due to EV-mediated detoxification of Complex II, or if they may instead result from alterations in TNFR1 trafficking, which could independently influence receptor signaling and cell death sensitivity. TNFR1 trafficking must be assessed: Given that many of the proteins under study (HSC70, ESCRT components, RAB5, microbial effectors) are involved in receptor trafficking, TNFR1 surface expression should be analyzed by flow cytometry across all relevant cell lines (parental, CRISPR/CAS9 KD/KO, and overexpression models). This is critical to rule out receptor mislocalization as a confounding factor. Figure 2g shows increased Complex I–bound TNFR1 in RAB5-S34N cells. This supports the above concern. Please provide TNFR1 surface levels in all experimental conditions.

> We thank the reviewer for this important remark. TNFR1 has indeed been reported to engage the endolysosomal pathway, and its trafficking is therefore likely affected under several of the conditions we examined. To distinguish defective ILV-mediated detoxification of TNFR1 Complex II from altered TNFR1 trafficking as the cause of the observed TNF cytotoxicity, we directly tested whether blocking TNFR1 trafficking impacts cell viability. First, we verified that TNFR1 is normally targeted for lysosomal degradation by comparing its protein levels in the presence or absence of the lysosomal fusion inhibitor Bafilomycin A1 (BafA1) (Fig. 3c). Notably, preventing lysosomal delivery of TNFR1 with BafA1 did not affect cell viability (Fig. 3d). We next interfered with earlier steps of TNFR1 transport through the endosomal system. Inhibition of TNFR1 sorting into ILVs through loss of the ESCRT-II subunit VPS36—whose absence does not impair TNFR1 Complex II targeting—stabilized TNFR1 (Fig. 3e) but again failed to induce TNF cytotoxicity (Fig. 3f). Finally, blocking DYNAMIN-1–mediated endocytosis of TNFR1 using the pharmacological inhibitor Dyngo-4a similarly prevented receptor degradation without triggering cell death (Fig. 3g-h). Consistent results were obtained using the dominant-negative DYN1^{S45N} mutant or the dominant-negative AP180 C-terminal fragment (Extended Data Fig. 3c-d). Together, these results demonstrate that interfering with TNFR1 trafficking at multiple stages of the endolysosomal pathway does not induce cell death.

Fig. 3c

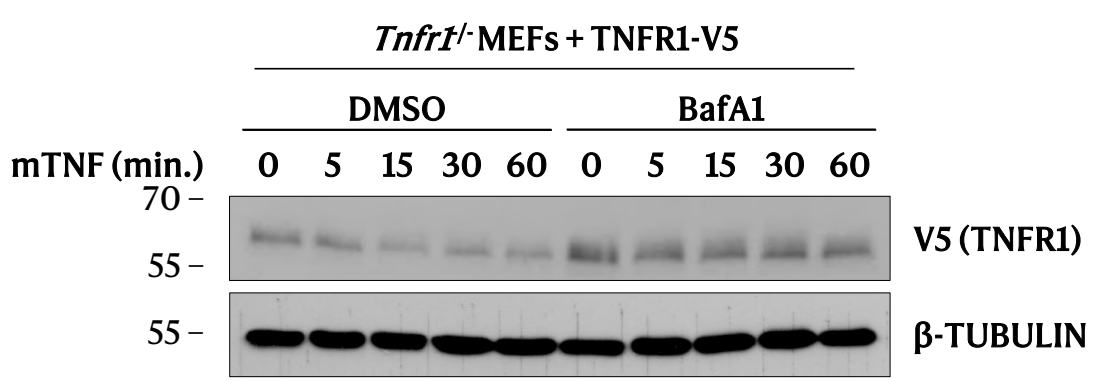

Fig. 3d

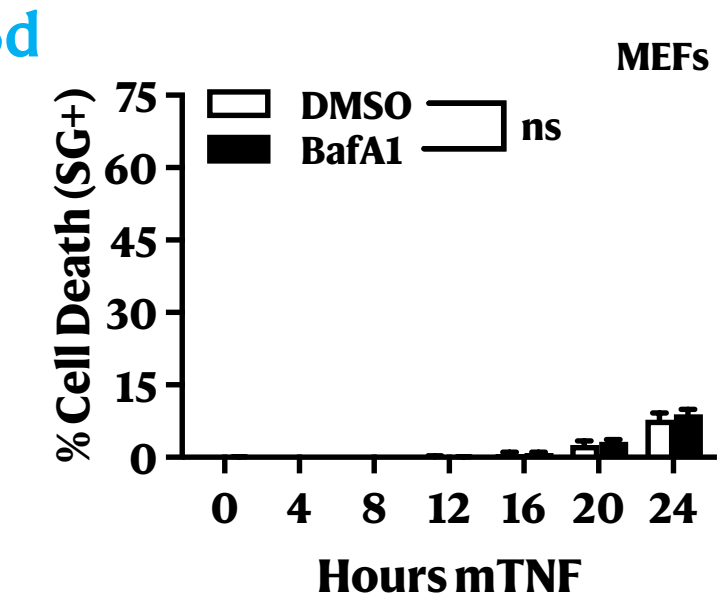

Fig. 3e

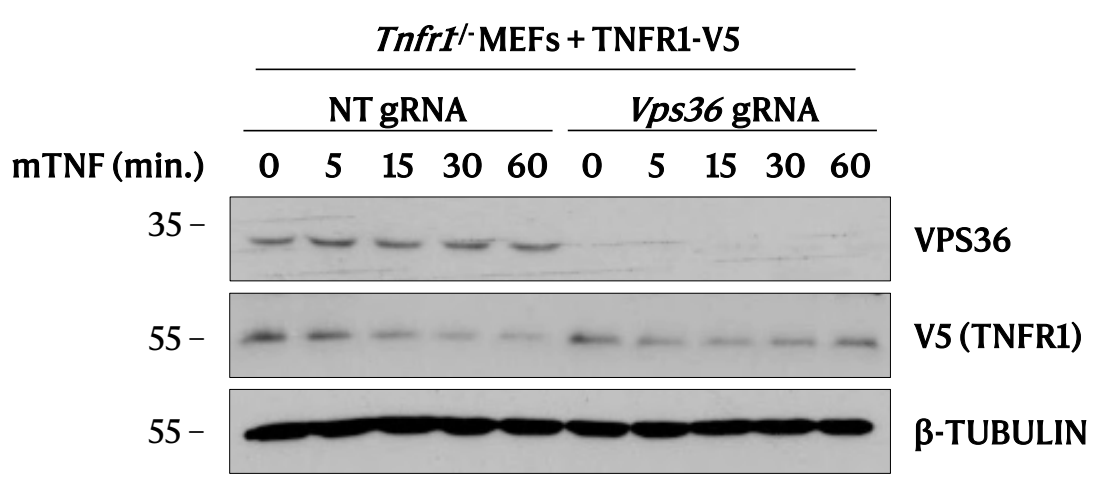

Fig. 3 f

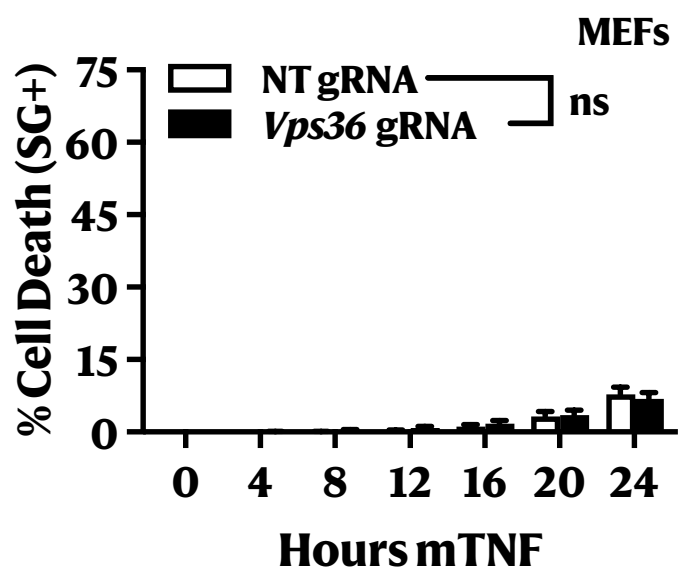

Fig. 3g

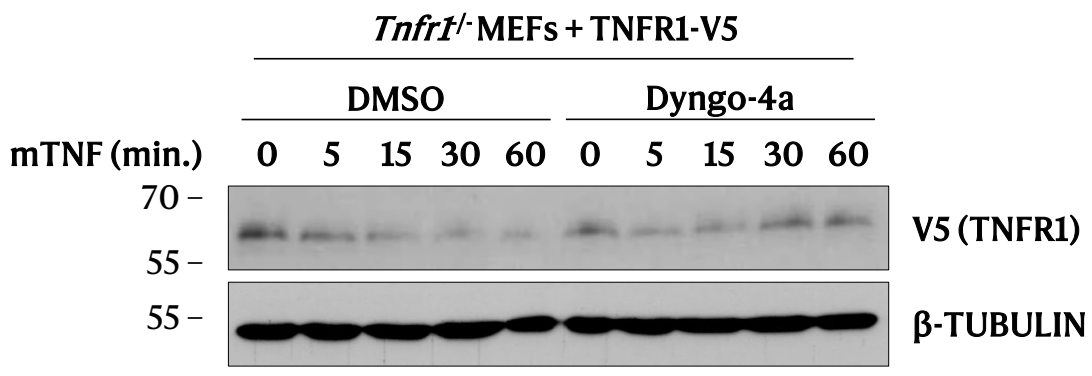

Fig. 3h

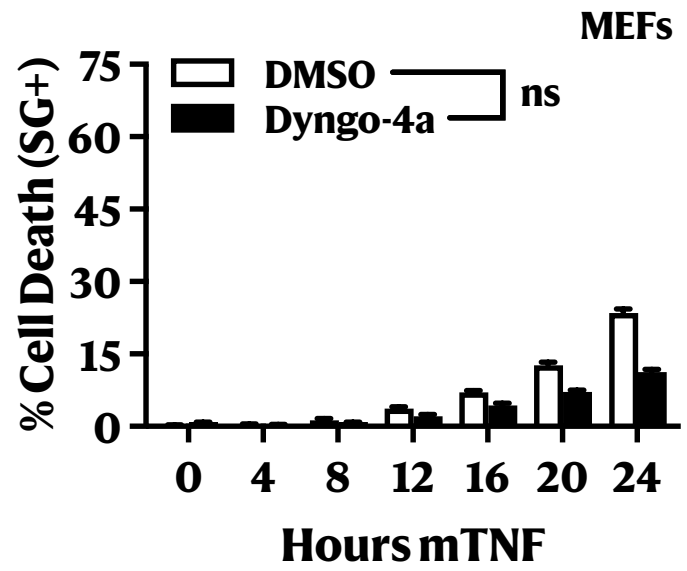

Fig. 3c-h. *Tnfr1* -/- MEFs were transduced with an inducible expression vector encoding V5-tagged TNFR1. Cells were either pre-treated with BafA1 (**Fig. 3c-d**), Dyngo-4a (**Fig. 3g-h**) or electroporated with Cas9-RNPs targeting *Vps36* or NT (**Fig. 3e-f**). Cells were then stimulated with 20 ng/mL mTNF for the indicated duration. Protein levels were determined by immunoblot (**c**, **e**, **g**) and cell death induction was measured over time by SytoxGreen (SG) positivity (**Fig. 3d**, **f**, **h**). Cell death experiments are presented as mean ± SEM of three independent experiments and statistical significance was determined using a two-way ANOVA.

ED Fig. 3c

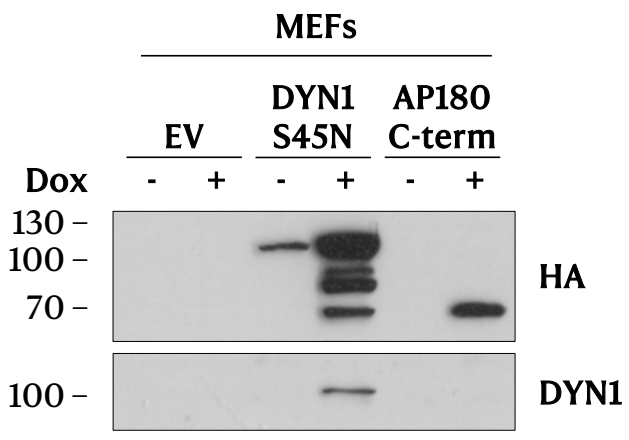

ED Fig. 3d

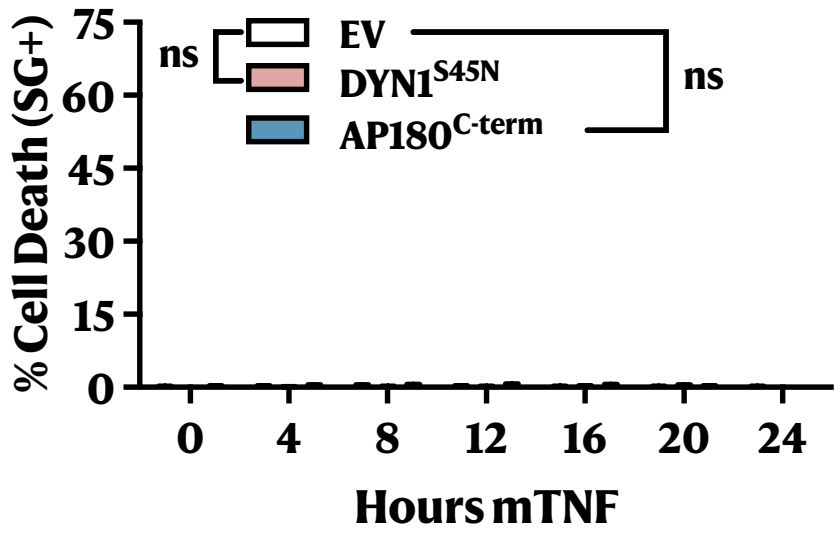

Extended Data (ED) Fig. 3c-d. MEFs were transduced with inducible expression vectors encoding HA-tagged versions of the dominant negative DYNAMIN-1 S45N mutant (DYN1^{S45N}), the dominant-negative C-terminal fragment of AP180 (AP180$^{C\text{-}term}$) or an empty vector (EV). **ED Fig.3c-d**, Cells were treated, or not, with 1 µg/mL Doxycycline (Dox) for 24h and protein levels were determined by immunoblot (**ED Fig. 3c**). Additionally, the cells were stimulated with 20ng/mL mTNF and cell death was measured over time by SytoxGreen (SG) positivity (**ED Fig. 3d**). Cell death experiments are presented as mean ± SEM of three independent experiments and statistical significance was determined using a two-way ANOVA.

> To further demonstrate that the cytoprotective effect of the ESCRTs is independent from the ESCRT-dependent sorting of TNFR1 into ILVs, we tested the effect of ESCRT inhibition by USP8i in cells in which TNFR1 internalization is blocked by Dyngo-4a. Remarkably, USP8i treatment still sensitized the cells to TNF-mediated apoptosis in these conditions (Fig. 3i-j).

Fig. 3i

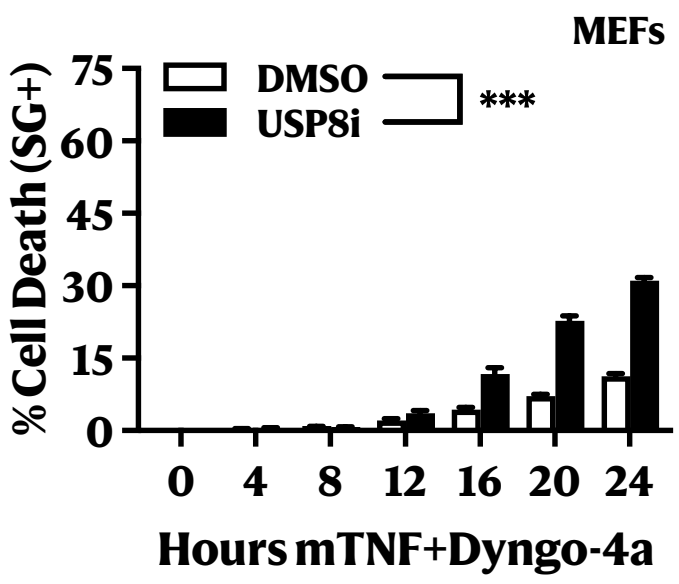

Fig. 3j

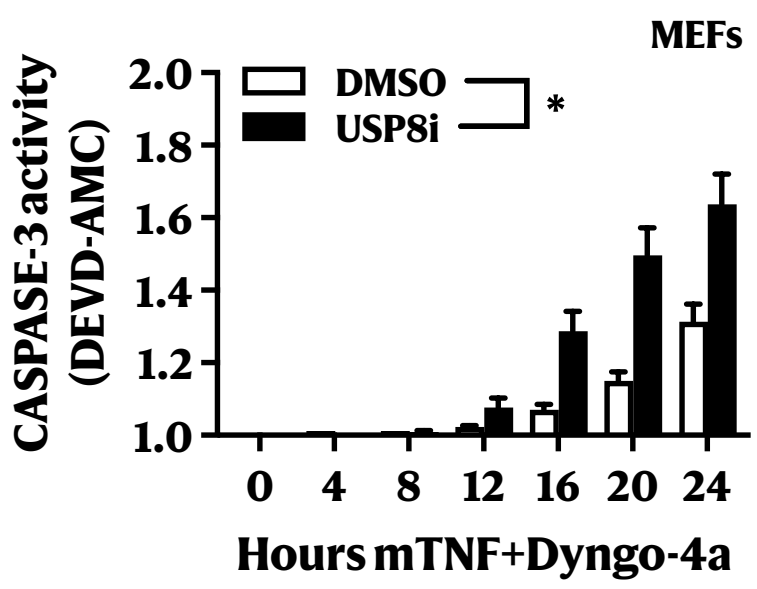

Fig. 3i-j. MEFs were pre-treated with Dyngo-4a and USP8i, as indicated, prior to stimulation with 20 ng/mL mTNF. ***Fig. 3i****, cell death was measured over time by SytoxGreen (SG) positivity.* ***Fig.3j****, Extracellular caspase activity was quantified over time using a fluorescent caspase-activity probe (DEVD-AMC). Cell death and CASPASE-3 activity experiments are presented as mean ± SEM of three independent experiments and statistical significance was determined using a two-way ANOVA.*

*> Together, these results demonstrate that the formation and cytotoxic potential of TNFR1 Complex II are independent of TNFR1 trafficking. The phenotypes observed in the manuscript cannot be attributed to receptor mislocalization but instead support the model in which the ESCRT machinery prevents TNF cytotoxicity by mediating the ILV-dependent sequestration and ultimately extracellular release- of TNFR1 Complex II. These new data have been incorporated into an entirely new figure in the revised manuscript dedicated to dissecting TNFR1 trafficking and its relationship to TNF-induced cell death (**Fig. 3** and **Extended Data Fig. 3**).*

> Given these findings, we believe that measuring TNFR1 surface expression by flow cytometry, as suggested by the reviewer, would not meaningfully contribute to the interpretation of our data. Moreover, ESCRT deficiency, RAB5^{S34N} expression, or microbial effector proteins do not impair internalization; they act downstream, by preventing ILV formation and thereby trapping receptors within endosomal compartments—not at the plasma membrane.

2. Clarify experimental design across conditions: There is inconsistency in the use of TNF Alone vs TNF + CHX, in WT or ATG9a−/− cells. A systematic side-by-side comparison of TNF-ICD in WT vs ATG9a−/− cells in the presence or absence of CHX would greatly improve interpretability and readability.

> We thank the reviewer for this helpful suggestion. To improve clarity, we have updated the labeling of all relevant figure panels to more clearly indicate the specific treatment conditions (TNF alone vs. TNF + CHX) and the corresponding genotypes (WT vs. ATG9A-deficient cells). This should enhance interpretability and ensure consistent comparison across experiments. Regarding a systematic side-by-side analysis of TNF-induced cell death in WT and ATG9A-deficient cells with or without CHX, we would like to refer the reviewer to our previous work (Huyghe et al., Science 2022, Fig. 1H), in which this comparison was performed.

3. Validate the nature of EVs: To confirm that the analyzed vesicles are not apoptotic bodies: Please include experiments using caspase inhibitors (e.g., ZVAD or QVD). Exclude Annexin V+ vesicles during EV isolation.

*> We agree with the reviewer that it is essential to exclude the possibility that the TNFR1 Complex II components detected in EVs originate from apoptotic bodies released by dying cells. Several lines of evidence in the original submission already argue strongly against this interpretation. First, HT1080 cells do not undergo apoptosis in response to the TNF concentration used in this study, as previously shown (Huyghe et al., Science 2022, Fig. S5C) and confirmed again in the revised manuscript (**Extended Data Fig. 4r**). Notably, this remains true at the 6-hour time point used for EV isolation. Second, apoptotic bodies are substantially larger than exosomes; however, nanoparticle tracking analysis (NTA) revealed no increase in EV particle size following TNF stimulation (**Extended Data Fig. 4x**). Third, ultrastructural analysis by electron microscopy showed no appearance of apoptotic bodies or morphological alterations in EV preparations from TNF-treated cells (**Extended Data Fig. 3w**). To address the reviewer's concern even more directly, we generated CASP3/7-deficient HT1080 cells, which are completely resistant to TNF-induced apoptosis (**Extended Data Fig. 4t**) and therefore incapable of forming apoptotic bodies. EVs isolated from these apoptosis-*

*resistant cells after TNF treatment still contained activated (Cl.) CASP8 (**Extended Data Fig. 4u**), definitively ruling out contamination by apoptotic bodies as the source of TNFR1 Complex II components. These new results have been added to the revised manuscript.*

ED Fig. 4t

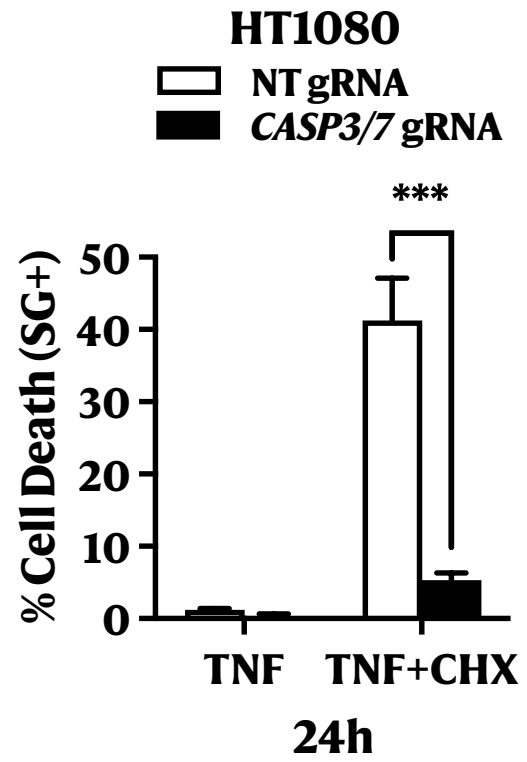

ED Fig. 4u

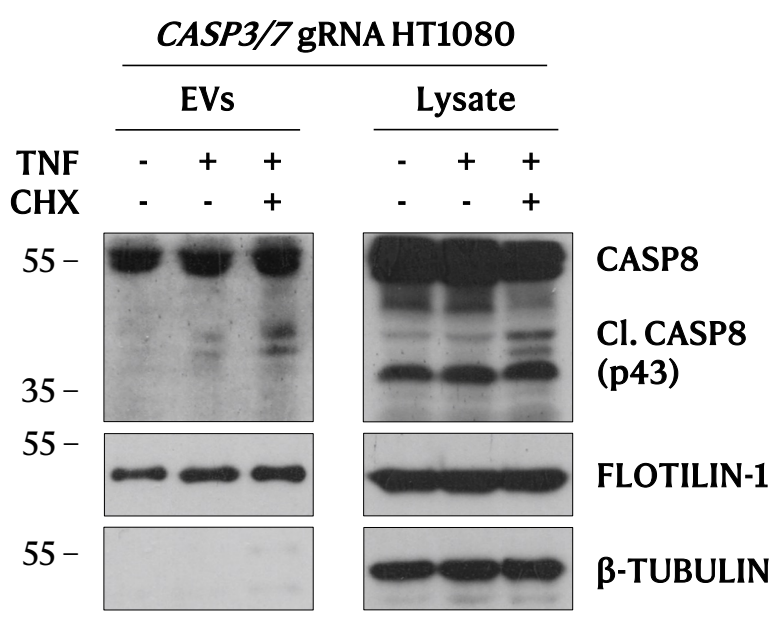

Extended Data (ED) Fig. 4t-u. ED Fig. 4t, HT1080 cells were electroporated with Cas9-RNPs targeting *CASP3* and *CASP7* or a non-targeting sequence (NT). Cells were pre-treated with CHX, as indicated, prior to stimulation with 20 ng/mL hTNF. Cell death was measured after 24h by SytoxGreen (SG) positivity.**ED Fig. 4u**, Cells were pre-treated with CHX, as indicated, prior to stimulation with 2 μg/mL hTNF for 6h. Cells were lysed and extracellular vesicles (EVs) were isolated from the conditioned medium using size exclusion chromatography (SEC) (Fractions 4-6). Protein levels were determined by immunoblot. Cell death experiments are presented as mean ± SEM of three independent experiments and statistical significance was determined using a two-way ANOVA or multiple unpaired t-tests.

4. EV profiling should be expanded to include the following TNFR1 CII components TRAF2, TRADD, c-FLIP, cIAP1, TRAF1, and compared to EVs obtained from cells stimulated with FasL and TRAIL to assess whether this detoxification process is TNF-specific.

*> We thank the reviewer for this suggestion. Regarding TRADD, the reviewer may have overlooked that its presence in EVs was already shown in the original manuscript (**Extended Data Fig. 4p**). Regarding the additional components proposed by the reviewer, many of these - such as TRAF2, cIAP1 and TRAF1 - are primarily associated with TNFR1 Complex I rather than being bona fide components of TNFR1 Complex II. In contrast, our profiling already demonstrates the presence of multiple well-established TNFR1 Complex II components (RIPK1, M1-Ub, TRADD, FADD, Cl. CASP8, TAX1BP1), providing strong evidence that the EVs indeed contain TNFR1 Complex II. Nevertheless, to address the reviewer's concern, we performed additional SEC-based EV isolations and examined the suggested proteins. While all factors were readily detectable in cell lysates, only TRAF2 exhibited a clear TNF-dependent enrichment in EVs. These new results have been incorporated into the revised manuscript (**Extended Data Fig. 4q**).*

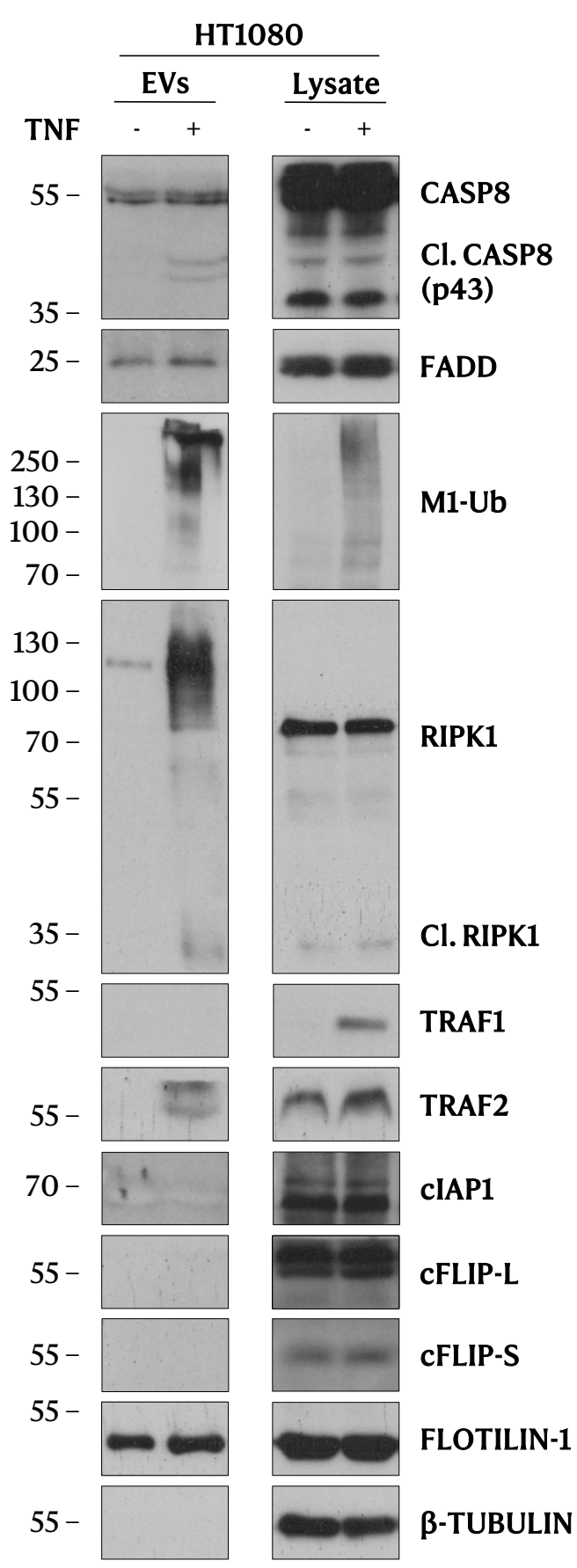

Extended Data Fig. 4q. HT1080 cells were stimulated, or not, with 2 µg/mL hTNF for 6h. Cells were lysed and extracellular vesicles (EVs) were isolated from the conditioned medium using SEC (Fractions 4-6). Protein levels were determined by immunoblot.

> We do not believe that comparing EV cargoes from TNF-, FasL-, or TRAIL-treated cells would yield mechanistically meaningful insight. It is well established that TNFR1 Complex II is a cytosolic complex that forms after dissociation of TNFR1 Complex I components from the receptor and their subsequent association with FADD and CASP8 (Micheau and Tschopp, Cell 2003). This distinguishes TNF signaling from FasL and TRAIL, whose Death-Inducing Signaling Complexes (DISCs) remain receptor-bound. The molecular mechanism that brings TNFR1 Complex II into EVs is therefore intrinsically different from the one that would bring those DISCs into EVs. To exclude the possibility that a DISC-like complex could eventually form at TNFR1 when ILV formation is blocked, we performed additional TNFR1 Complex I immunoprecipitations in Tsg101-deficient MEFs. Even at time points where activated (Cl.) CASP8 is readily detectable in the cytosol, we did not detect CASP8 or Cl. CASP8 in TNFR1 Complex I (Fig. 3b), demonstrating that DISC formation does not occur at the level of TNFR1 even when endolysosomal trafficking is impaired. For these reasons, we consider cross-comparison with FasL- or TRAIL-derived EVs not informative for the present study.

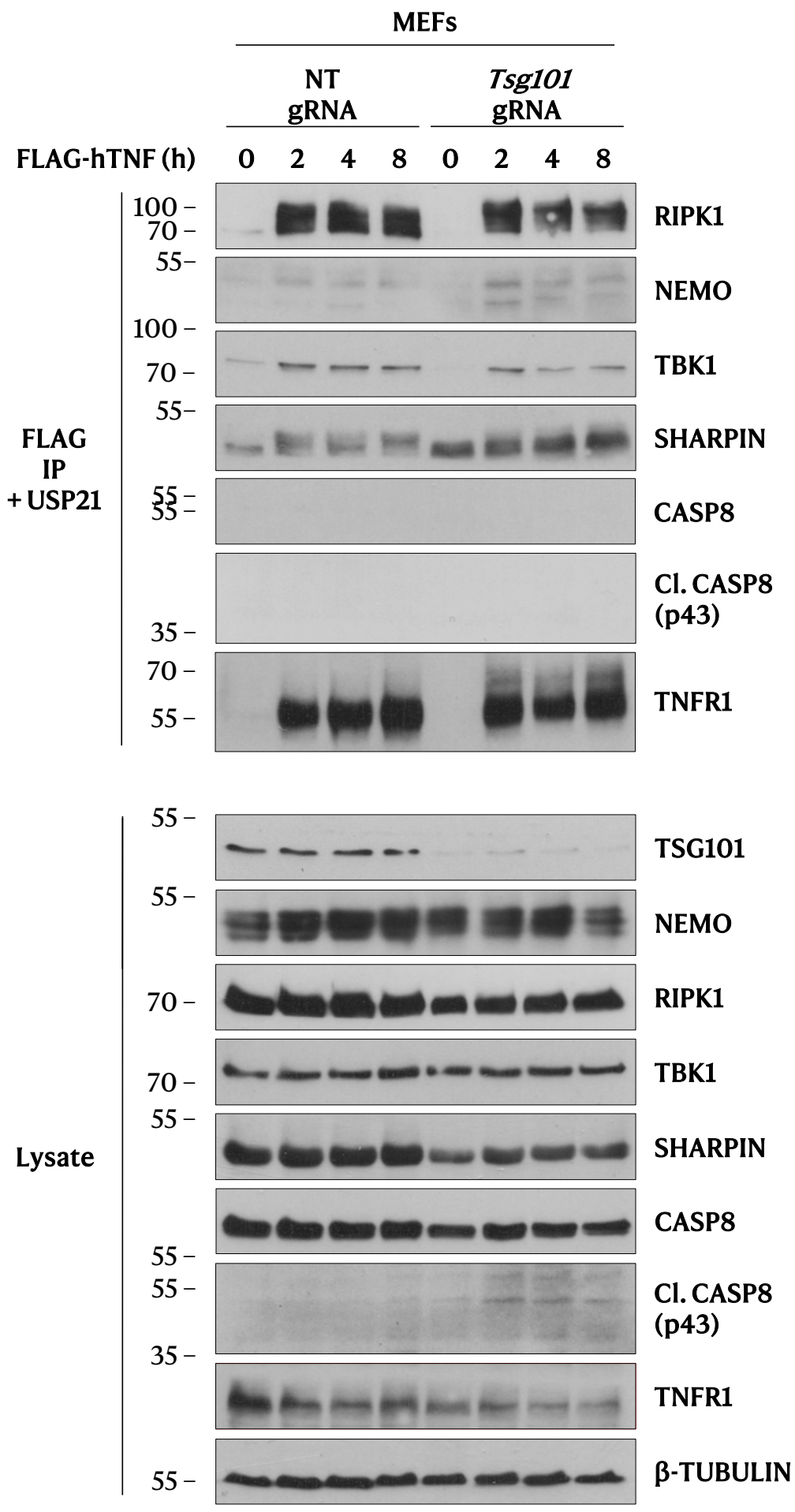

Fig. 3b. MEFs were transduced with an inducible expression vector encoding Cas9 (hereafter referred to as iCas9 MEFs) and subsequently transduced with inducible expression vectors encoding sgRNAs targeting *Tsg101* or a non-targeting sequence (NT). Cells were treated with 1 µg/mL Doxycycline for 48h and subsequently expanded for 72h. Cells were stimulated with 1 µg/mL FLAG-hTNF for the indicated duration. TNFR1 Complex I was FLAG-immunoprecipitated, followed by USP21 treatment. Protein levels were determined by immunoblot.

5. Broaden your discussion: Extend discussion to other ligands of the family ie : FasL and TRAIL ie the following published work (See https://www.cell.com/cell/abstract/S0092-8674(25)00728-7 and https://pubmed.ncbi.nlm.nih.gov/32807832/)

> We appreciate the reviewer's suggestion to broaden the Discussion by addressing FasL and TRAIL. For the reasons outlined above, we do not believe that integrating these ligands into the discussion of this study would provide meaningful insight. Indeed, TNFR1 Complex II is a cytosolic complex that forms after receptor dissociation, whereas FasL and TRAIL generate receptor-bound DISCs with distinct regulatory logic and trafficking requirements. Given these fundamental differences - and our new data demonstrating that TNF-induced cytotoxicity is uncoupled from TNFR1 trafficking - we do not expect FasL/TRAIL complexes

to be governed by the ESCRT-dependent detoxification mechanism described here. However, since our findings demonstrate that endosomal targeting of TNFR1 Complex II primarily leads to its extracellular release, we have expanded the Discussion to explore potential roles in intercellular communication and to speculate on the biological consequences of transferring TNFR1 Complex II components between cells (Page 22, line 1-30).

*> Additionally, our data argue against the possibility that increased TNF sensitivity in ESCRT-deficient cells stems from non-canonical CASP8 activation on unclosed phagophores. Even in macroautophagy-deficient conditions - where phagophores are absent - additional ESCRT deficiency further sensitizes cells to TNF-induced apoptosis (**Fig. 1i and Extended Data Fig. 1u–x, z**). Moreover, new results included in the revised manuscript show that expression of the dominant-negative RAB5^{S34N} mutant does not impair macro-autophagy (**Extended Data Fig. 2c**) and therefore is not expected to promote non-canonical CASP8 activation. Nevertheless, this mutant blocks ILV-mediated sequestration of TNFR1 Complex II and sensitizes cells to TNF-induced apoptosis (**Fig. 2b, d**). Together, these findings firmly establish that the ESCRT-dependent checkpoint operates independently of non-canonical CASP8 activation at phagophore membranes. This clarification is now briefly addressed in the Discussion section: "Moreover, the observation that ESCRT deficiency sensitizes cells to TNF cytotoxicity even in the absence of macro-autophagy also distinguishes our findings from the previously reported non-canonical, lethal activation of CASP8 on unsealed phagophores[42]. This conclusion is reinforced by the fact that expression of RAB5^{S34N} sensitizes cells to TNF-induced death without exerting any measurable effect on the macro-autophagy pathway." – Page 21, lines 15-21.*

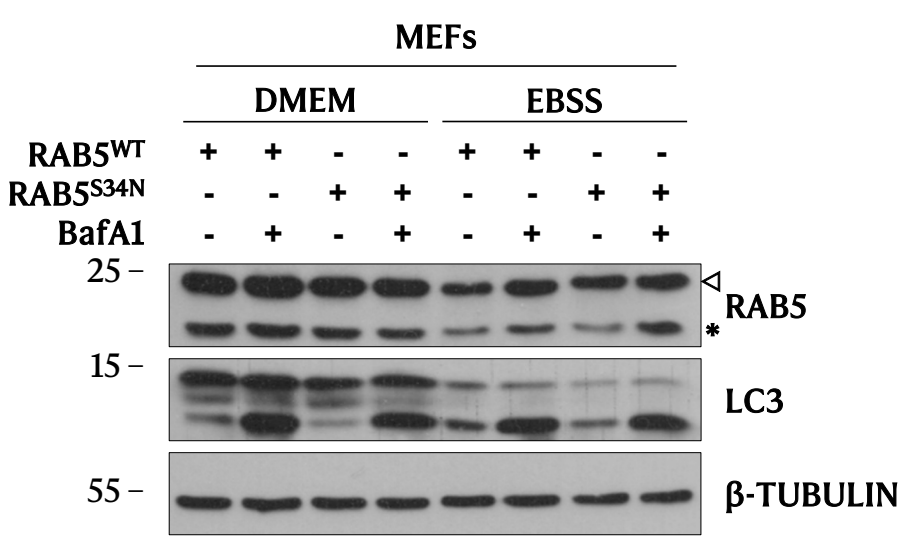

Extended Data Fig. 2c. MEFs were transduced with inducible expression vectors encoding either 3xHA-tagged wild-type (WT) RAB5 or dominant-negative mutants RAB5^{S34N}. Cells were treated, or not, with 2 μg/mL Doxycycline (Dox) for 48h. Cells were subsequently treated with EBSS for 4h in the presence or absence of BafA1. Protein levels were determined by immunoblot.

6. Please include immunoprecipitation of TNFR1 at time zero from lyzed cells (Anti-FLAG + FLAG-TNF pulldown from untreated cellular extracts) for proper validation.

> The control suggested by the reviewer is sometimes included in TNFR1 Complex I immunoprecipitation experiments, but our laboratory (Priem et al., Methods Mol. Biol., 2018) and others have routinely used the control strategy presented in our original manuscript without encountering difficulties in data interpretation or observing meaningful differences relative to time-zero controls. Importantly, none of our conclusions depend on subtle variations in baseline TNFR1 pulldown efficiency, and the newly added data - which formally demonstrate that ILV formation regulates TNF cytotoxicity independently of TNFR1 trafficking - further reinforce that including an additional time-zero control would not alter the interpretation of the results. For these reasons, we respectfully believe that repeating all TNFR1 immunoprecipitations with an additional time-zero control would not provide added value or lead to different conclusions.

7. Comment and confirm the reduced RIPK1 ubiquitination in TSG101-deficient cells. Is this reduction seen in the other experiments? Have they been performed at least 3 times independently? If so please indicate so in the manuscript and show for the referee the other two repeats.

*> While we acknowledge the reviewer's observation of a modest decrease in RIPK1 ubiquitination in TNFR1 Complex I at 5 minutes after TNF stimulation in TSG101-deficient cells, we do not consider this difference biologically meaningful. The apparent reduction correlates with a slightly lower overall amount of TNFR1 and associated TNFR1 Complex I components recovered in the immunoprecipitation from TSG101-deficient cells, suggesting a technical rather than functional origin. Importantly, we do not observe RIPK1 activation within TNFR1 Complex I - a hallmark of defective ubiquitination - and TNF-induced cell death in TSG101-deficient cells is clearly RIPK1 kinase-independent (**Fig. 1e-f**). Both observations support the conclusion that RIPK1 ubiquitination remains functionally intact in the absence of TSG101. As indicated in the figure legends, all western blot experiments were performed independently at least twice. For transparency, we now provide the data from the additional independent repeat for the reviewer's inspection (**Rebuttal Fig. 1**).*

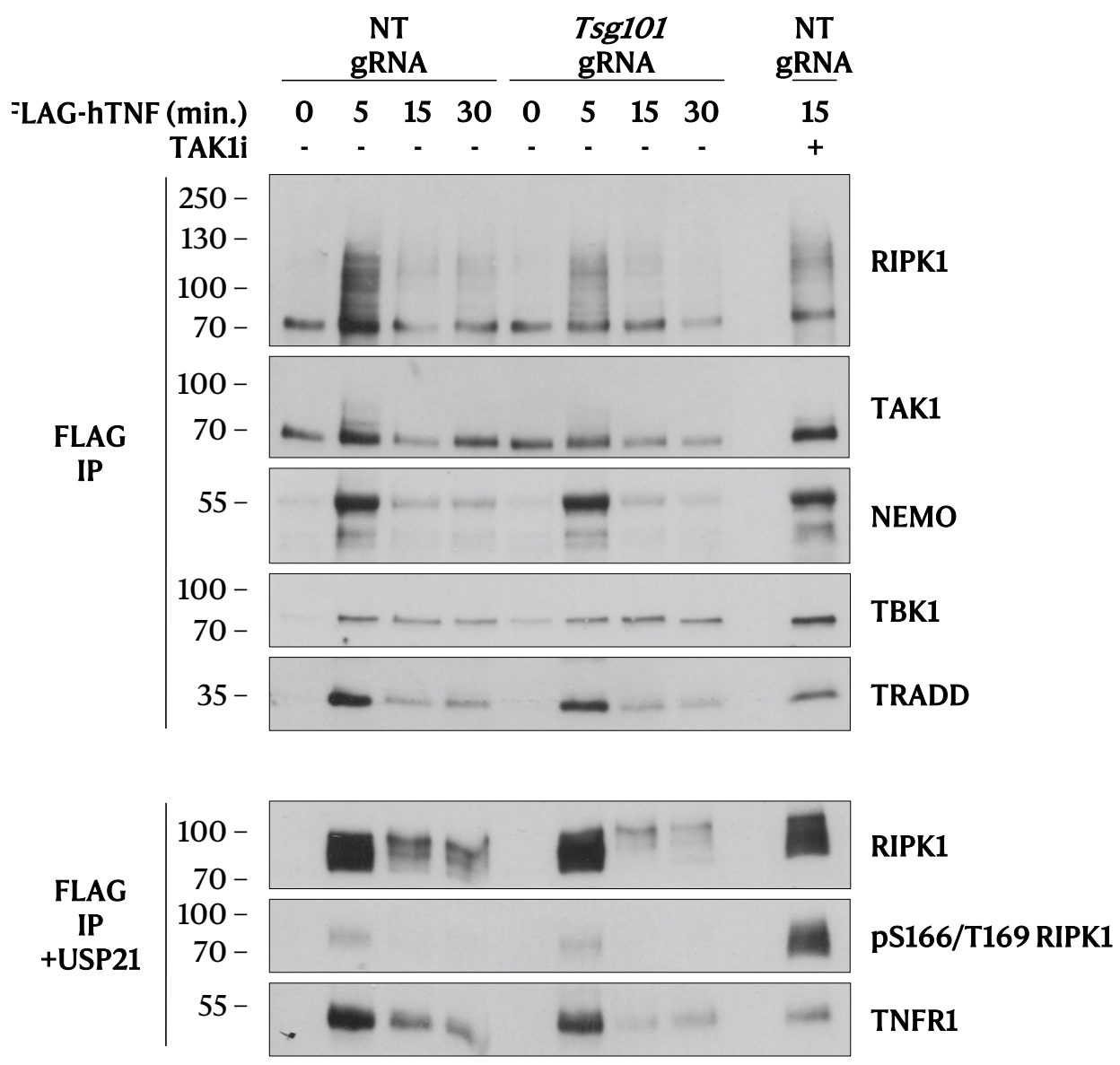

Rebuttal Fig. 1. MEFs were transduced with an inducible expression vector encoding Cas9 and subsequently transduced with inducible expression vectors encoding sgRNAs targeting *Tsg101* or NT. Cells were treated with 1 µg/mL Doxycycline for 48h and subsequently expanded for 72h. Cells were pre-treated, or not, with TAK1i prior to stimulation with 1 µg/mL FLAG-hTNF for the indicated duration. TNFR1 Complex I was FLAG-immunoprecipitated, followed by USP21 treatment, or not. Protein levels were determined by immunoblot.

8. It is unclear why VPS34 inhibition (with drug) enhances TNF-induced death in MEF cells, while not in HT1080 cells (e.g., S1U-V vs S1X). Please clarify these discrepancies and include side-by-side comparisons of VPS34 KD (gRNA) vs inhibitor treatments.

> We acknowledge the reviewer's observation that VPS34 inhibition results in higher TNF cytotoxicity in MEFs than HT1080 cells. Although the basis for this difference remains unclear, the effect is consistent with the results of our previous study, where it was found not to be specific to VPS34. Indeed, similar differences were also observed upon inactivation of other components of the LC3-independent macro-autophagy pathway, such as ATG9A or FIP200 (Huyghe et al., Science 2022 - Fig. S1F–G). These results therefore indicate that HT1080 cells are more resistant than MEFs to inactivation of the LC3-independent macro-autophagy checkpoint. In the same study, we also directly compared pharmacological inhibition of VPS34 with SAR405 to CRISPR-mediated VPS34 deletion and observed no

substantial differences between these two approaches (Huyghe et al., *Science 2022 - Fig. 2C, Fig. S3E–F).*

9. Were DGUC/SEC and EV analyses done in the presence or absence of CHX? This is not stated and is crucial for interpretation. If not a control including CHX would be needed as this would match confocal data.

*> We thank the reviewer for highlighting the importance of clarifying whether CHX was used during EV isolation. As stated in the original figure legends, all DGUC/SEC-based EV isolations were performed exclusively on cells not pre-treated with CHX. CHX was only used in microscopy experiments, where it is necessary to amplify CASP8 activation sufficiently to allow reliable detection of Cl. CASP8 puncta by confocal imaging. Without CHX, the signal remains below the single-cell detection threshold. To directly bridge the microscopy and EV datasets, we additionally performed EV isolations from TNF-treated cells in the presence of CHX. As expected, CHX pre-treatment increased the amount of Cl. CASP8 detected in EVs (**Extended Data Fig. 4s**), consistent with its ability to increase the cellular pool of TNFR1 Complex II above the imaging detection limit. However, CHX is a potent sensitizer of apoptosis (**Extended Data Fig. 4r**), raising the possibility of apoptotic body contamination—a concern also raised by the reviewer. To exclude this, we repeated the experiment in apoptosis-resistant CASP3/7-deficient HT1080 cells (**Extended Data Fig. 4t**), which cannot form apoptotic bodies. Even in this background, CHX pre-treatment similarly increased Cl. CASP8 levels in EVs (**Extended Data Fig. 4u**) confirming that the additional signal does not originate from apoptotic bodies. Importantly, and central to our conclusions, TNFR1 Complex II is readily detectable in EVs even in the absence of CHX, demonstrating that this phenomenon does not depend on artificially boosting CASP8 activation. We have now incorporated these additional data into the revised manuscript for clarity (**Extended Data Fig. 4r-u**).*

ED Fig. 4r

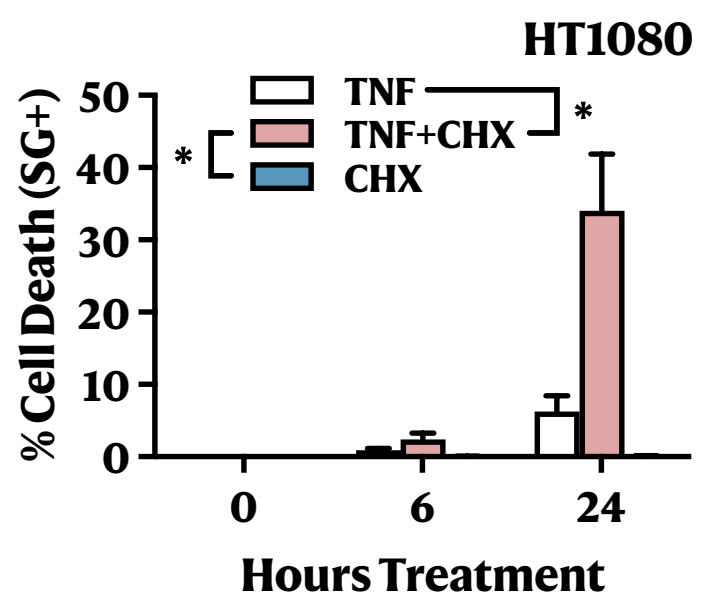

ED Fig. 4s

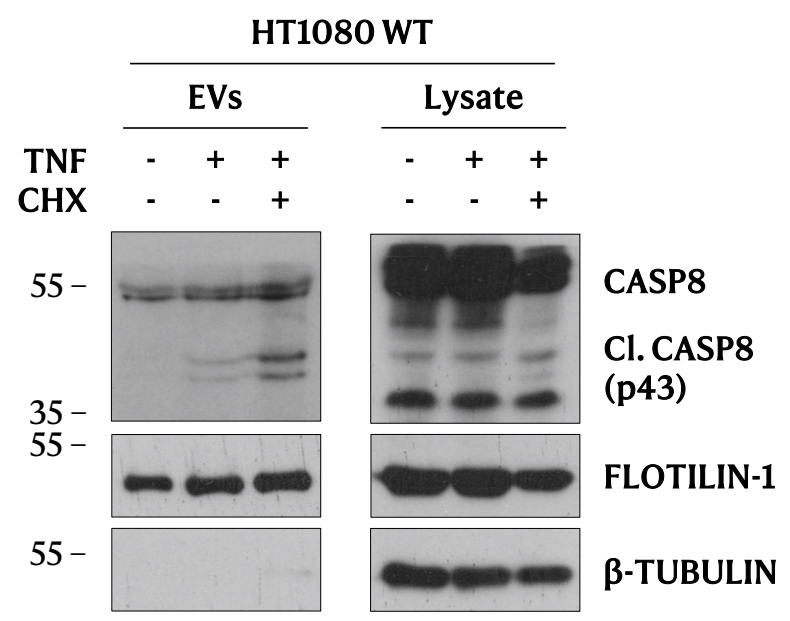

ED Fig. 4t

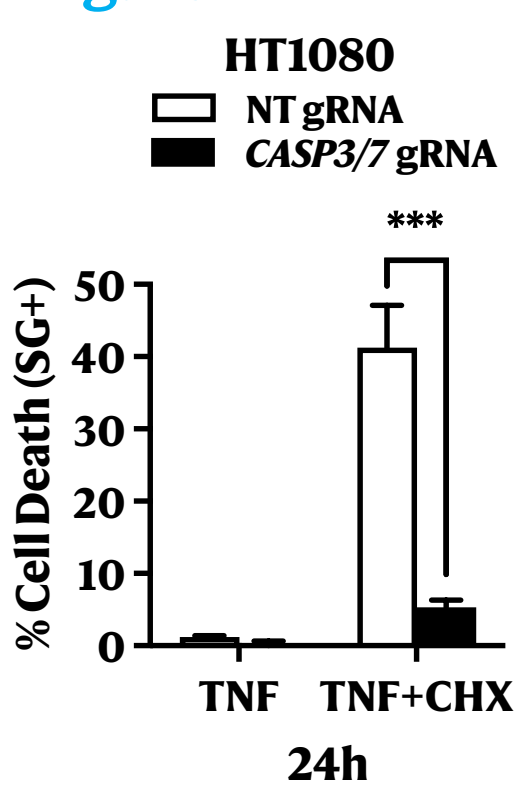

ED Fig. 4u

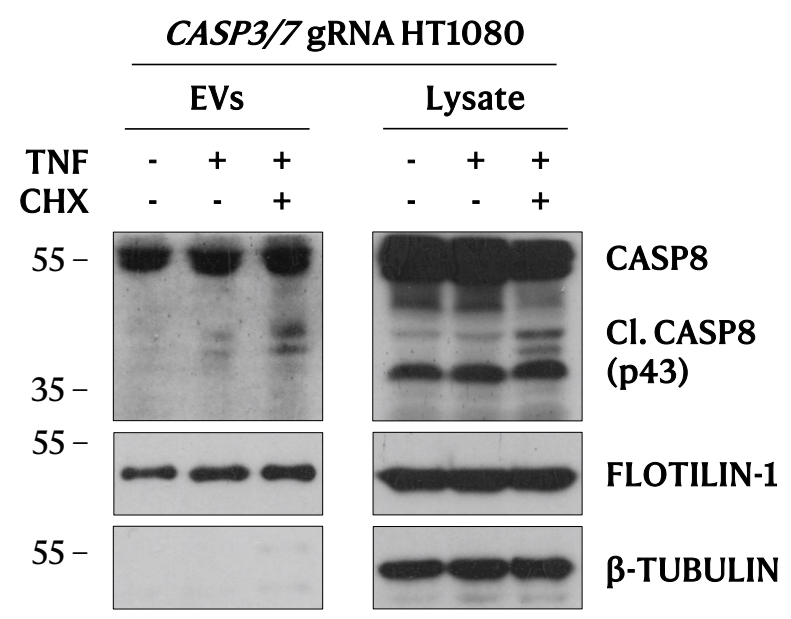

Extended Data (ED) Fig. 4r-u. ED Fig. 4r, HT1080 cells were pre-treated with CHX, as indicated, prior to stimulation with 20 ng/mL hTNF. Cell death was measured over time by SytoxGreen (SG) positivity. **ED Fig. 4t-u**, HT1080 cells were electroporated with Cas9-RNPs targeting *CASP3* and *CASP7* or a non-targeting sequence (NT). **ED Fig. 4t**, Cells were pre-treated with CHX, as indicated, prior to stimulation with 20 ng/mL hTNF. Cell death was measured after 24h by SytoxGreen (SG) positivity. **ED Fig. 4s, u**, Cells were pre-treated with CHX, as indicated, prior to stimulation with 2 μg/mL hTNF for 6h. Cells were lysed and extracellular vesicles (EVs) were isolated from the conditioned medium using size exclusion chromatography (SEC) (Fractions 4-6). Protein levels were determined by immunoblot. Cell death experiments are presented as mean ± SEM of three independent experiments and statistical significance was determined using a two-way ANOVA or multiple unpaired t-tests.

10. Why is FADD not detected in HT1080 EVs (Fig. S3J)?

*> A closer examination of the figure (**Extended Data Fig. 4p**) shows weaker bands corresponding to FADD, which is consistent with our other data on the EV content of TNF-treated cells. The reduced signal in this particular experiment most likely reflects a shorter exposure time. Importantly, the EV lysates analyzed in **Fig. 4h** were prepared using the same SEC protocol and show much stronger FADD signals, confirming the reproducibility of this finding.*

11. The presence of HSC70 in EVs is not shown. Only indirect evidence via sensitivity to TNF is provided to point to HSC70 as a cargo protein for eMI. Please include HSC70 in your biochemical EV analyses.

> We thank the reviewer for this valuable suggestion. In the original manuscript, we demonstrated that HSC70—independently of its ATPase activity—is required for the transfer of TNFR1 Complex II into endosomes and for protection against TNF cytotoxicity. However, we agree that directly assessing the presence of HSC70 in EVs provides important complementary evidence. To address this, we performed additional biochemical analyses of EVs isolated by SEC following TNF stimulation. In addition to the expected TNF-dependent enrichment of linear ubiquitin chains, ubiquitinated RIPK1, and TAX1BP1, we now observe increased levels of HSC70 in SEC fractions enriched for established exosome markers (TSG101, ALIX, and FLOTILLIN-1) (Fig. 5a). These findings are fully consistent with the proposed role of HSC70 in selectively facilitating the loading of TNFR1 Complex II into ILVs. The new data have been incorporated into the revised manuscript.

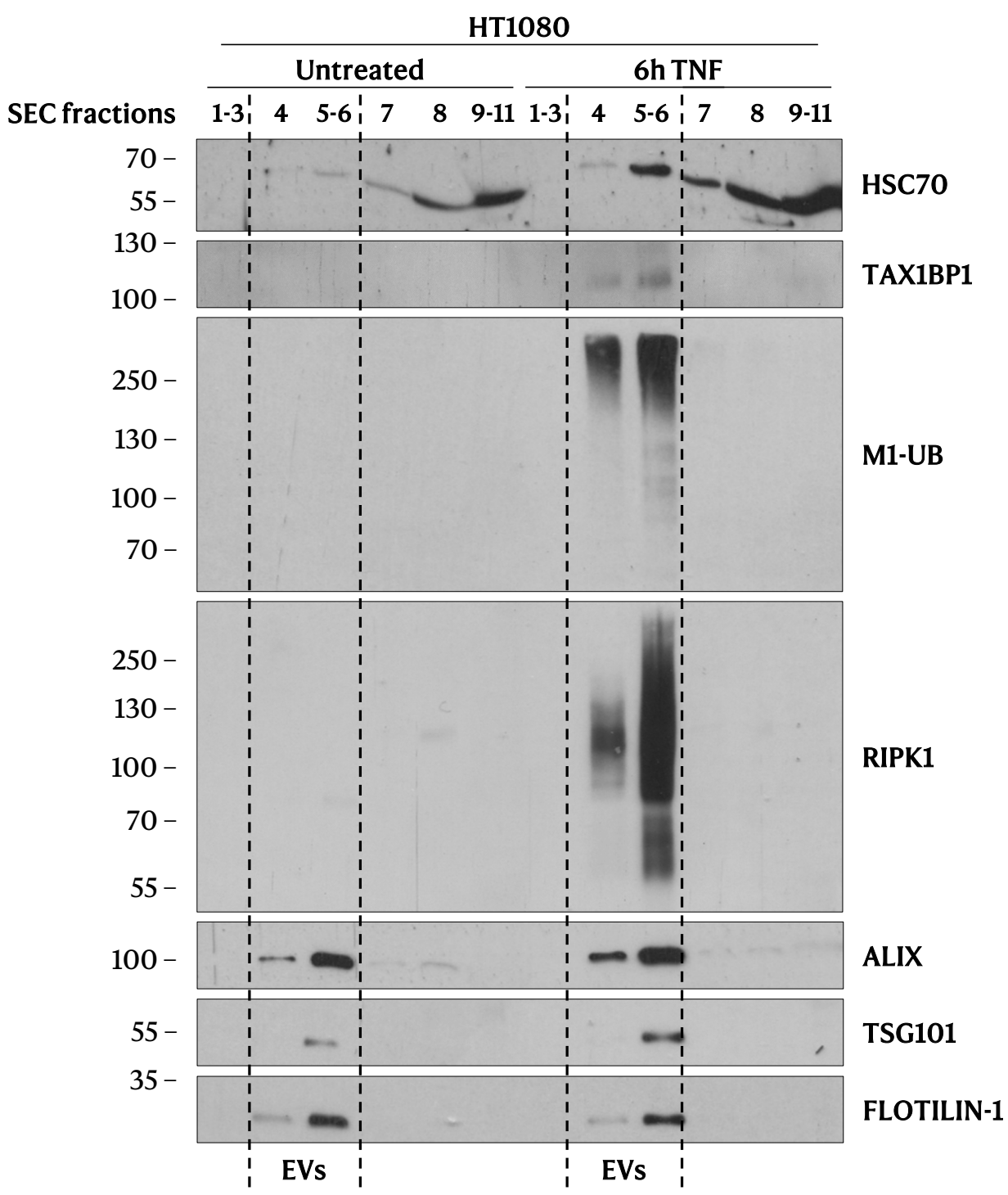

Fig. 5a. HT1080 cells were stimulated, or not, with 2 μg/mL hTNF for 6h. Cells were lysed and conditioned medium was harvested and separated into different fractions using size exclusion chromatography (SEC). Protein levels in each fraction were determined by immunoblot.

12. Clearly label each panel with the relevant cell line (MEF, HT1080, etc.), as it is not always obvious.

> We thank the reviewer for this suggestion and have updated the labeling of the figure panels accordingly to improve interpretability and readability.

13. Figure S1S: Immunoblots showing TSG101 deficiency in MEF-ATG9a−/− cells should include WT MEFs for reference.

> The purpose of introducing TSG101 deficiency into ATG9A-deficient MEFs was solely to assess the impact of ESCRT loss in a macro-autophagy-deficient background. At no point in the manuscript do we directly compare ATG9A-deficient cells with wild-type MEFs. For this reason, we respectfully disagree that including such a comparison would represent an informative control or alter the interpretation of our results.

14. Figure 3g: The caspase-8 blot shows three bands—please clarify the molecular identity of each (e.g., p55, p43/p41, p18).

> We thank the reviewer for this suggestion and have updated the labeling of the figure panels accordingly to improve interpretability and readability.

15. Figure 5: Textual Issue: The sentence "...sensitizing effects of SopB and EsxG-H were lost when cells were pre-treated with USPsi..." contradicts Fig. 5b vs 5e. At most, you can conclude that SopB's effect is additive to or masked by USP8 inhibition, but not abolished. Please revise for accuracy.

*> We agree with the reviewer that the interpretation of these results was not correctly phrased in this sentence. We have therefore rephrased to increase clarity: "This notion was further supported by the fact that SopB and EsxG-H have no additional sensitizing effect when the ESCRT machinery is already inhibited by pre-treatment with USP8i (**Fig. 6e** and **Extended Data Fig. 6h**)."- Page 17, lines 29-31.*

16. "..The discrepancy between the two studies concerning the effect of ESCRT deletion on TNF-mediated NF- κB/MAPK signaling is unclear, but may reflect the cell type-specific targeting of TNFR1 Complex Ito the endolysosomal system..." Which studies, please add references.

> We note that the study by Song et al. *was referenced earlier in the manuscript when discussing discrepancies with our findings. However, we agree with the reviewer that an additional citation at this point is helpful to improve clarity and have therefore added this to the revised manuscript.*

17. "...phosphorylation of RIPK3 and MLKL (markers of necroptosis induction) were not observed upon stimulation of either wild type (WT) or ESCRT-deficient MEFs with TNF..." Could you dd the precision of the time at which you did this analysis as this may not be true at other time points!

> We thank the reviewer for pointing this out and have rephrased the Results section to describe the data more precisely.

18. The Original title: "Endosomal micro-phagy prevents TNF-induced cell death by targeting TNFR1 complex II" is vague, could you consider the following one "Endosomal Microautophagy Limits TNF-Induced Apoptosis by Redirecting TNFR1 Complex II for Exosomal Secretion" to describe ore precisely your conclusions?

> We thank the reviewer for this constructive comment. In line with remarks from other reviewers, we performed additional experiments to evaluate whether ESCRT-dependent sequestration of TNFR1 Complex II into ILVs leads to lysosomal degradation. The new results indicate that this process primarily mediates extracellular release of the complex via exosomes rather than lysosomal targeting. These data have been added to the revised manuscript. Since this mechanism does not strictly qualify as autophagy, we have rephrased the title and adjusted the manuscript to more accurately reflect our findings: "Exosomal Export of TNFR1 Complex II Prevents TNF-Induced Cytotoxicity".

Reviewer #4

1. Graphical abstract to explain the mechanism in the manuscript

*> We agree with the reviewer that a graphical abstract would be a valuable addition to improve accessibility for readers. We have therefore incorporated a graphical abstract that provides a graphical summary of the key findings and a detailed overview of the proposed mechanism in the last figure panel of the revised manuscript (**Fig. 6j**).*

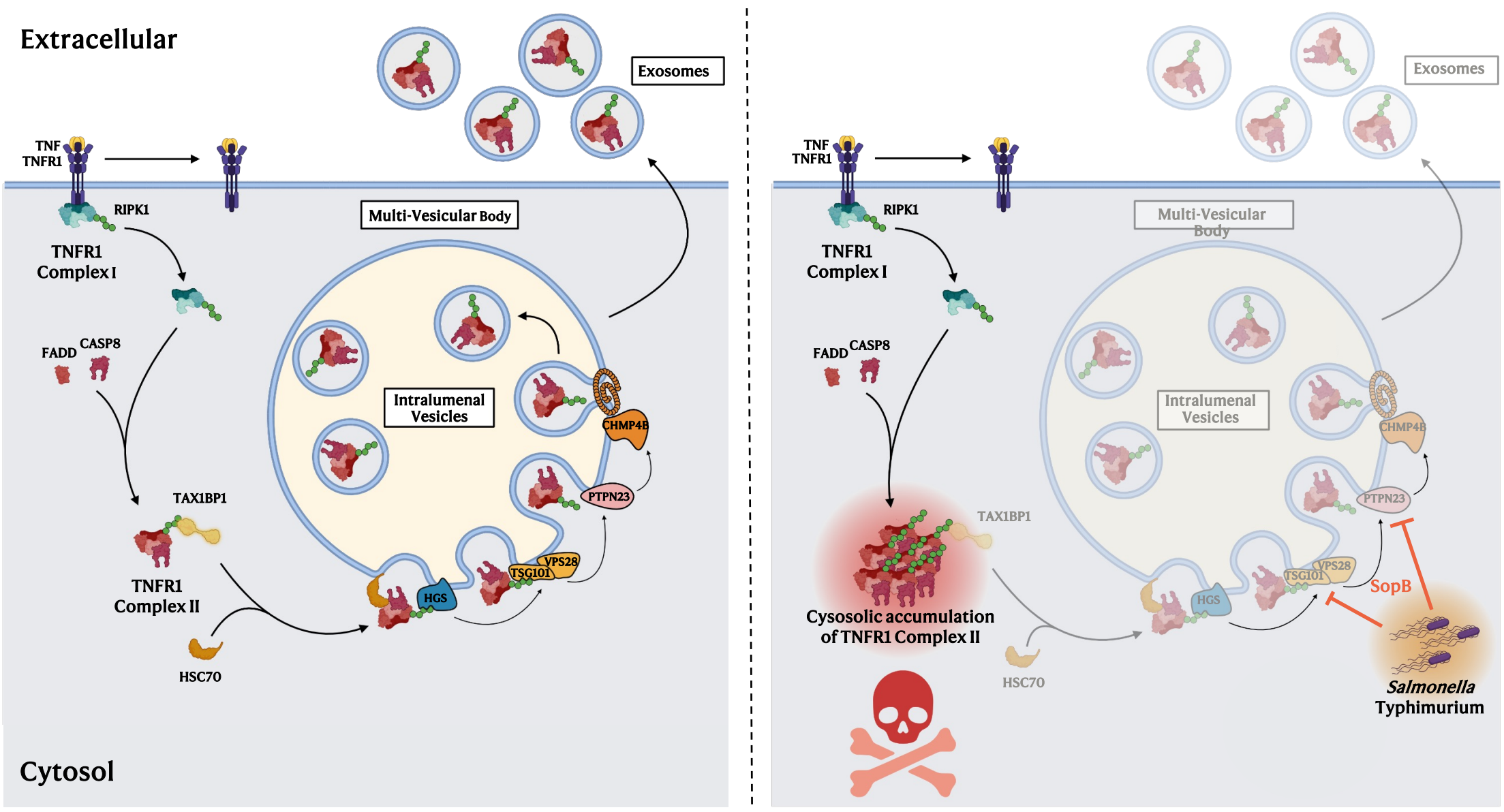

Fig. 6i. Graphical overview of the ESCRT-dependent detoxification of TNFR1 Complex II via extracellular release. Upon TNF sensing, TNFR1 Complex I assembles at the plasma membrane. RIPK1 and TRADD subsequently dissociate from the receptor-associated complex and join FADD and CASP8 to form the cytosolic TNFR1 Complex II. LUBAC decorates RIPK1 and additional TNFR1 Complex I/II components with M1-linked ubiquitin chains, which are recognized by the autophagy receptor TAX1BP1. Together with HSC70, TAX1BP1 then promotes the recruitment of TNFR1 Complex II to the limiting membrane of endosomes, where the ESCRT machinery mediates its incorporation into intralumenal vesicles (ILVs). The resulting Multivesicular body (MVB) can then fuse with the plasma membrane to release ILV-embedded TNFR1 Complex II into the extracellular environment as part of exosomes. Disruption of this pathway - such as by ESCRT inhibition due to the action of the *Salmonella* Typhimurium effector protein SopB - prevents the ILV-dependent detoxification of TNFR1 Complex II, leading to its cytosolic accumulation and the induction of TNF-induced RIPK1 kinase–independent apoptosis.

2. More detailed explanation of medical implications of the pathway explored is needed

> We agree with the reviewer that the original manuscript contained only a limited discussion of the potential medical implications. In the revised version, we have expanded the Discussion to more thoroughly speculate on the pathophysiological relevance of the identified pathway and its potential impact in disease contexts: "While transient inactivation of a cell death checkpoint can be beneficial in the context of infection, its constitutive inhibition is instead detrimental and causes pathogenesis[1]. Interestingly, numerous human mutations in components of the ESCRT machinery have been identified across patients presenting with diverse clinical symptoms[73]. Because the ESCRT components fulfil many functions beyond ILV formation, pinpointing the specific contribution of impaired TNFR1 Complex II detoxification to these disease manifestations will be challenging. Nevertheless,

our findings indicate that excessive TNF-induced cell death may contribute to the pathology in at least a subset of cases. Notably, together with recent reports, our data suggest a more specific role of PTPN23 in directing cytosolic cargo into ILVs[15,29]. Homozygous or compound heterozygous mutations in PTPN23 cause the neurologic disorder NEDBASS, characterized by neurodevelopmental delay, structural brain abnormalities, impaired intellectual development with delayed or absent speech, axial hypotonia, and peripheral spasticity[74–76]. It will be important to determine whether these mutations disrupt the exosomal detoxification of TNFR1 Complex II and whether uncontrolled TNF-induced cell death contributes to disease progression. If so, anti-TNF biologics could represent a promising therapeutic avenue for affected patients." – Page 24, lines 25-31 and Page 25, lines 1-10.

3. Fig 1 shows USP8 inhibitor, along with the TNF, induces TNF-mediated cell death. Additionally, TNF-mediated cell death is been induced in ESCRT mutant components.

*> We regret that the reviewer's remark is not entirely clear to us. The data in **Fig. 1** illustrates that both pharmacological and genetic targeting of the ESCRT machinery sensitize cells to TNF-induced death, highlighting the importance of the ESCRT components in counteracting TNF cytotoxicity.*

4. Fig 1c- Why there are repeated cleaved Casp. 3 blots? It is mentioned in the text (page 5, line 25) for the PARP but not shown in the results.

*> We assume that the reviewer is referring to repeated Cl. CASP8 blots? The different blots for Cl. CASP8 represent different cleavage fragments. For clarity, we have now explicitly labeled the individual fragments in the figure. The text refers to the cleavage of both CASP8, CASP3 and PARP. The reviewer may have missed that Cl. PARP was already shown in **Fig. 1c** of the original manuscript.*

5. Fig 1b, c, d- No information for the used cell lines.

> We thank the reviewer for this remark. We have updated the figure panel labels to clearly indicate the respective cell lines used, thereby improving readability and interpretability.

6. Most of the graphs of % cell death (Fig 1e, extended 1g) show high cell death (more than or up to 50%) in the DMSO. How is it true?

> In Fig. 1e of the original manuscript, the data are from MEFs with CRISPR-mediated Tsg101 deficiency. In these cells, treatment with TNF in combination with DMSO (control for Nec1s) results in sensitization to TNF cytotoxicity due to loss of TSG101. In Extended Data Fig. 1g of the original manuscript, the data are from HT1080 cells treated with TNF and USP8i, with or without Nec1s. Here, DMSO also serves as the control for Nec1s, and these cells are sensitized to TNF cytotoxicity due to USP8 inhibition.

7. Page 6, line 23 "In contrast to....". This statement cannot be aligned with the fig 1f.

*> We acknowledge the reviewer's observation that minor differences in TNFR1 Complex I assembly could be inferred from **Fig. 1f**. However, we do not consider these to represent a substantial biological effect, but rather technical variation. Indeed, we do not observe major differences in the ability of TSG101-deficient cells to activate the NF-kB and MAPKs pathways in response to TNF (**Fig. 1g**). In addition, we also do not detect RIPK1 activation within TNFR1 Complex I in these cells, which is a hallmark of defective TNFR1 Complex I assembly. Furthermore, the cell death observed in TSG101-deficient cells is RIPK1 kinase-independent, supporting the conclusion that TNFR1 Complex I assembly remains intact. Finally, in the compared publication, the authors report enhanced, and not reduced, signaling by TNFR1.*

8. Ext. fig. 1J- TNFR1 blot is not clear

> We agree with the reviewer that this blot is not optimal in clarity. We have therefore replaced it with a higher-quality version in the revised manuscript.
