# Supplementary material for: Exosomal export of activated CASPASE-8 is linked to the prevention of TNF-induced cytotoxicity
Source: Nat Commun. 2026 Aug 25;17:10178. doi: 10.1038/s41467-026-75544-1 (PMC13612411; doi:10.1038/s41467-026-75544-1)

Exosomal Export of Activated CASPASE-8 is linked to the prevention of TNF-Induced Cytotoxicity

Jon Huyghe[1,2,*], Dario Priem[1,2], Annelore Haems[1,2], Lien Lippens[3,4], Margaux De Meyer[5,6], Tom Delanghe[1,2], Yves Dondelinger[1,2], Inge Bruggeman[1,2], Sven Eyckerman[5,6], An Hendrix[3,4], Mathieu JM Bertrand[1,2,*]

[1]VIB Center for Inflammation Research; 9052 Ghent, Belgium. [2]Department of Biomedical Molecular Biology, Ghent University; 9052 Ghent, Belgium. [3]Laboratory of Experimental Cancer Research, Department of Human Structure and Repair, Ghent University, Ghent, Belgium. [4]Cancer Research Institute Ghent, Ghent, Belgium. [5]VIB-UGent Center for Medical Biotechnology, Ghent, Belgium. [6]Department of Biomolecular Medicine, Ghent University, Ghent, Belgium.
*Corresponding authors: jon.huyghe@irc.vib-ugent.be, mathieu.bertrand@irc.vib-ugent.be

Fig. 1 | The ESCRT machinery prevents TNF-induced RIPK1 kinase-independent apoptosis independently of the known cell death checkpoints

c

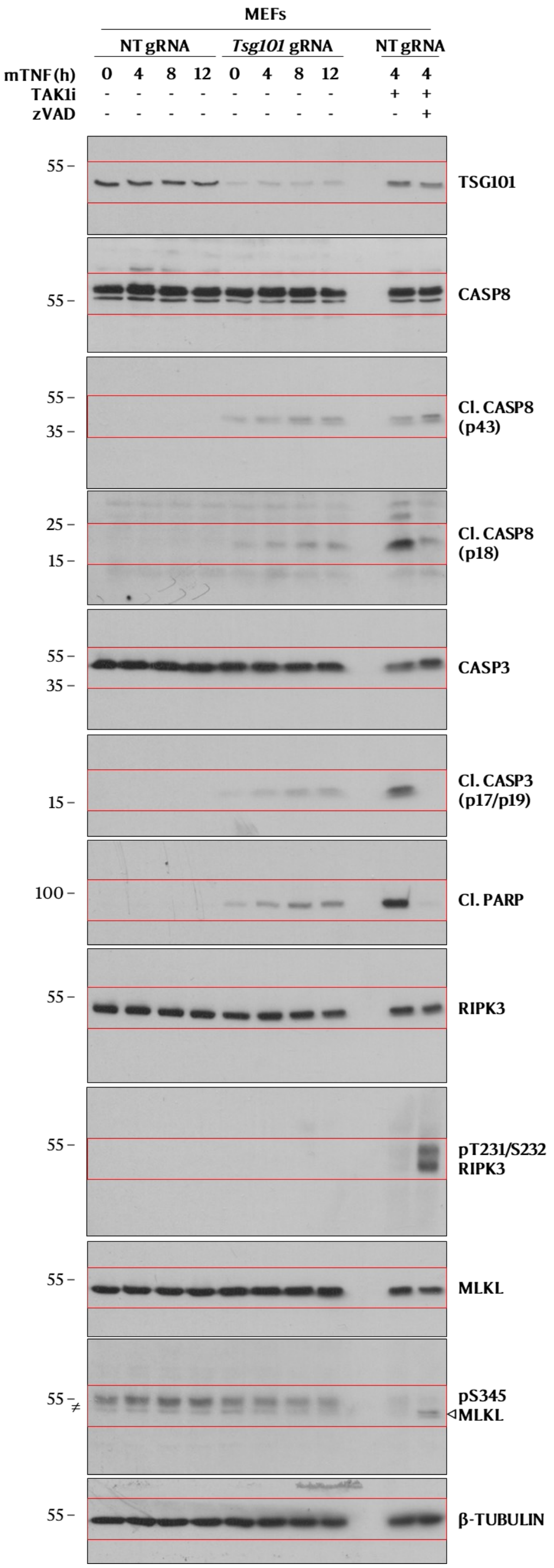

f

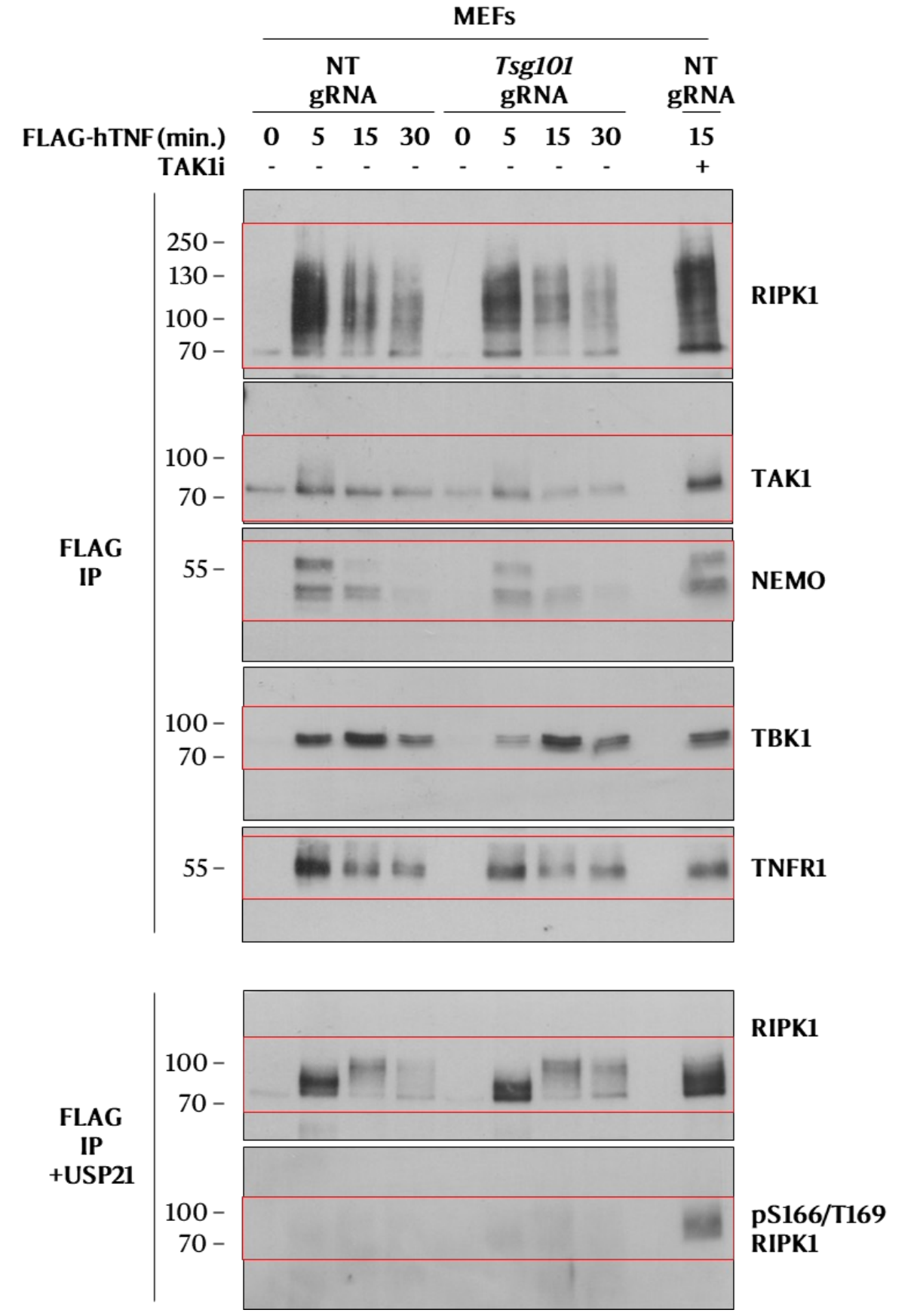

g

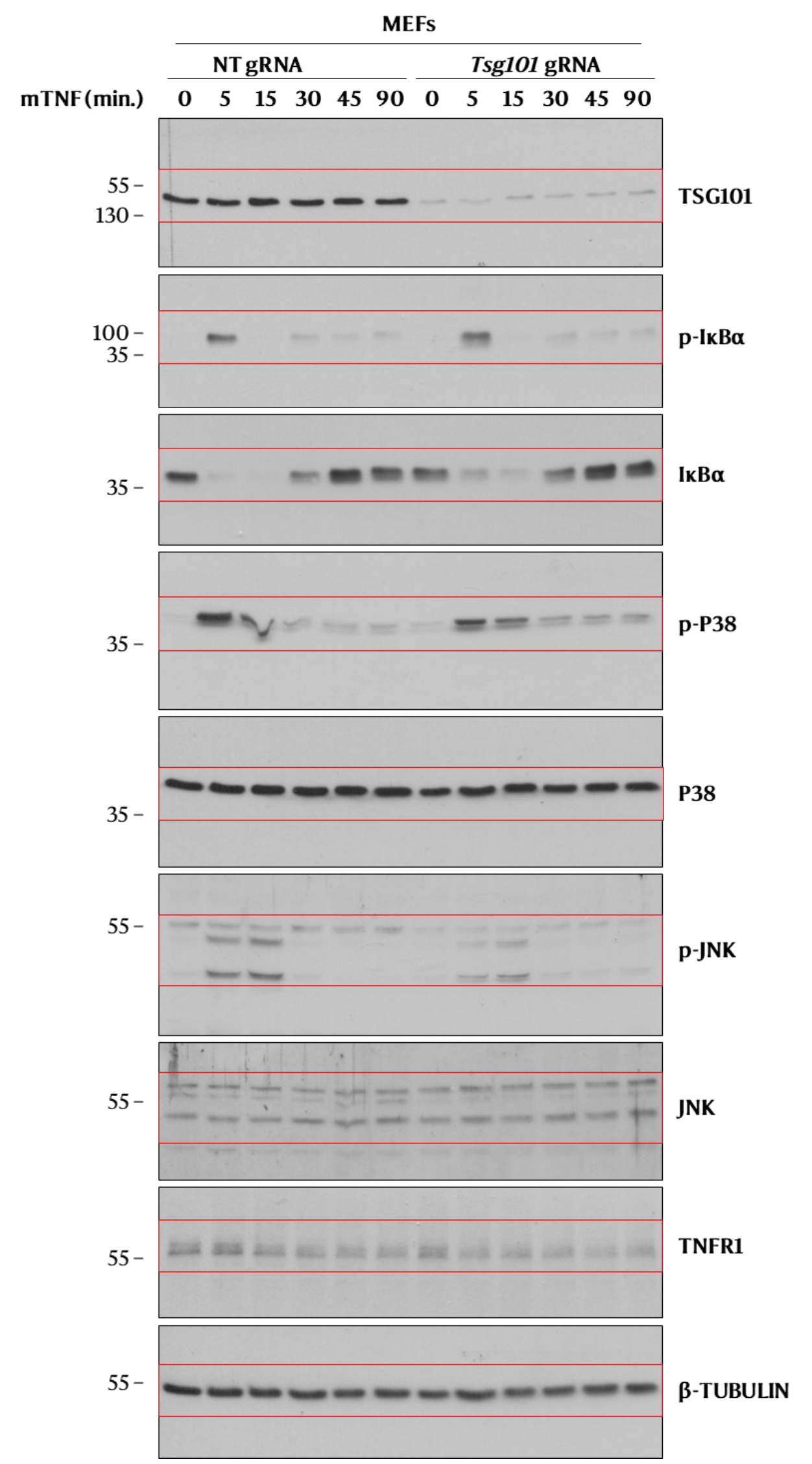

Fig. 2 | Endosomal ILV formation prevents TNF-induced RIPK1 kinase-independent apoptosis

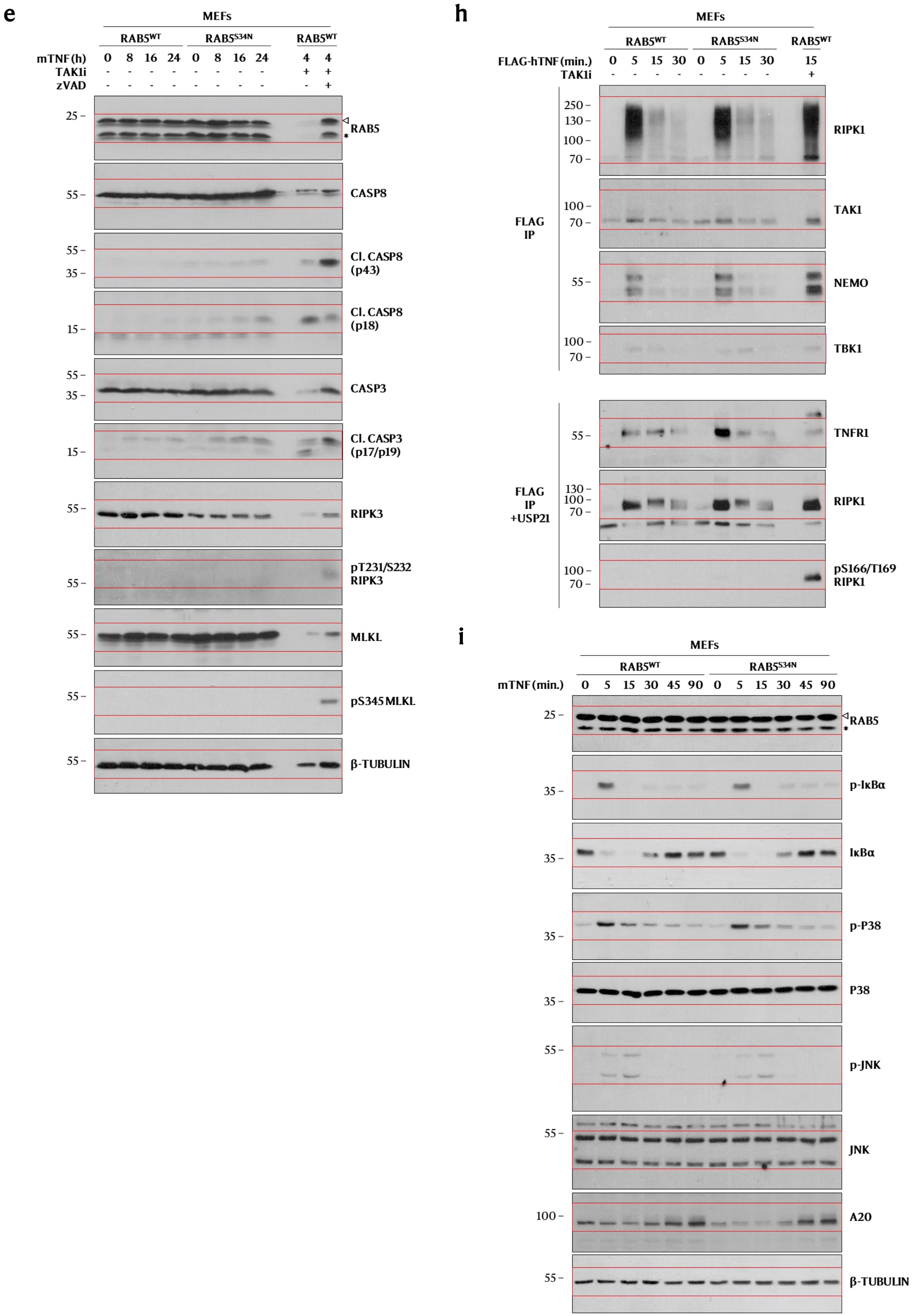

Fig. 3 | ILV formation protects against TNF cytotoxicity independently of TNFR1 trafficking

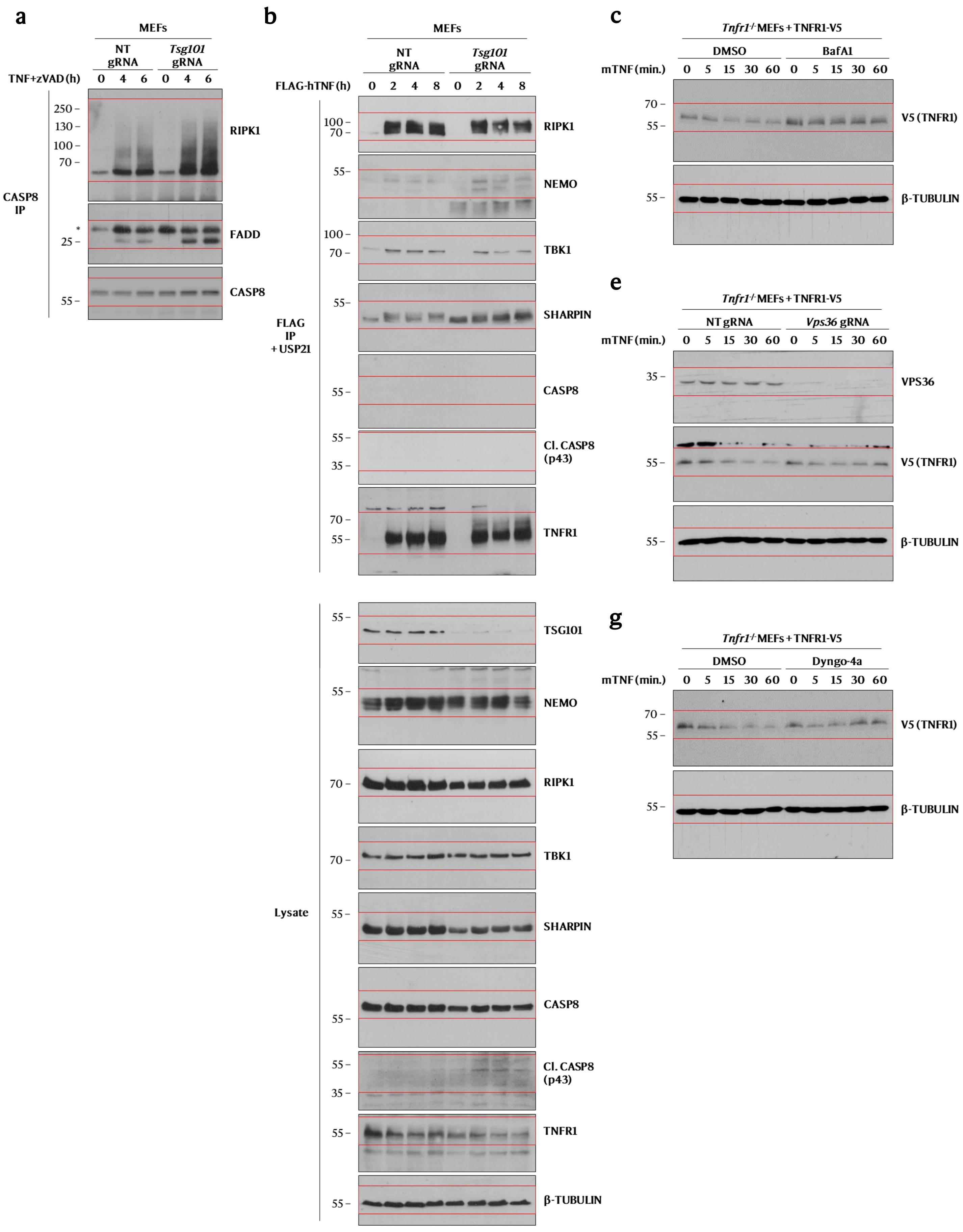

Fig. 4 | Targeting of activated CASP8 into ILVs prevents TNF-induced cytotoxicity

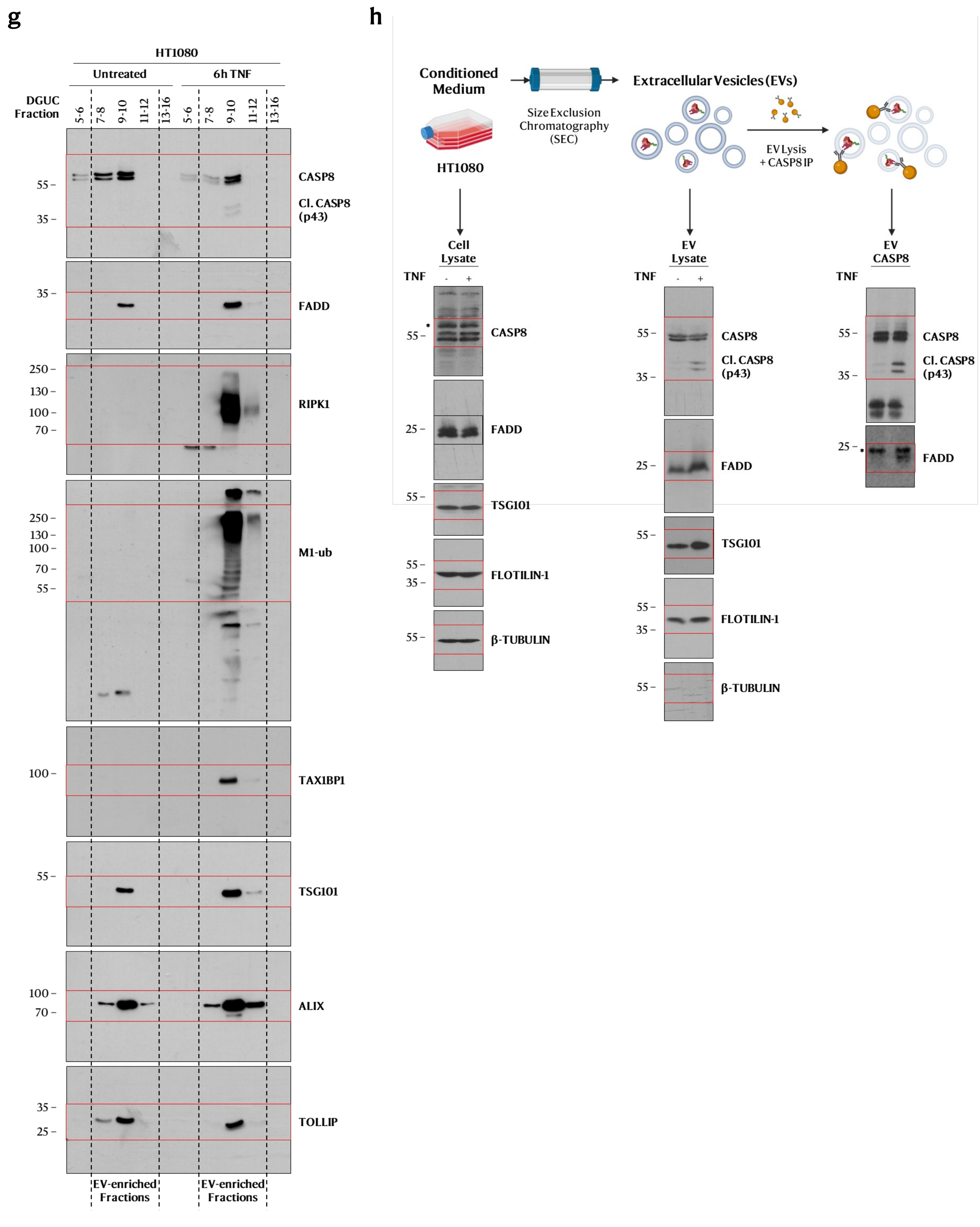

Fig. 5 | Linear ubiquitination of RIPK1 promotes the TAX1BP1- and HSC70-dependent targeting of activated CASP8 into ILVs

a

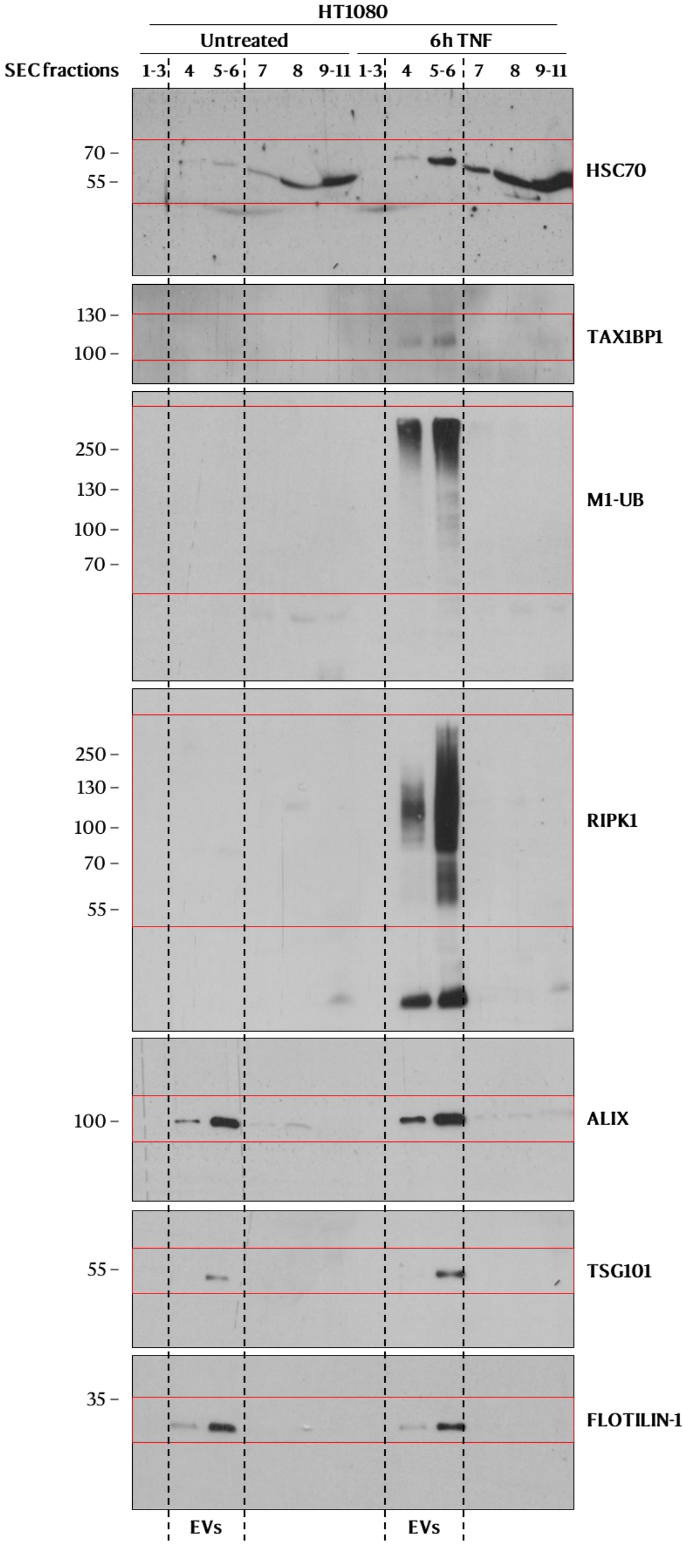

Fig. 6 | Microbial effector proteins shift the TNF response from life to death by interfering with the ILV-dependent detoxification of activated CASP8

a

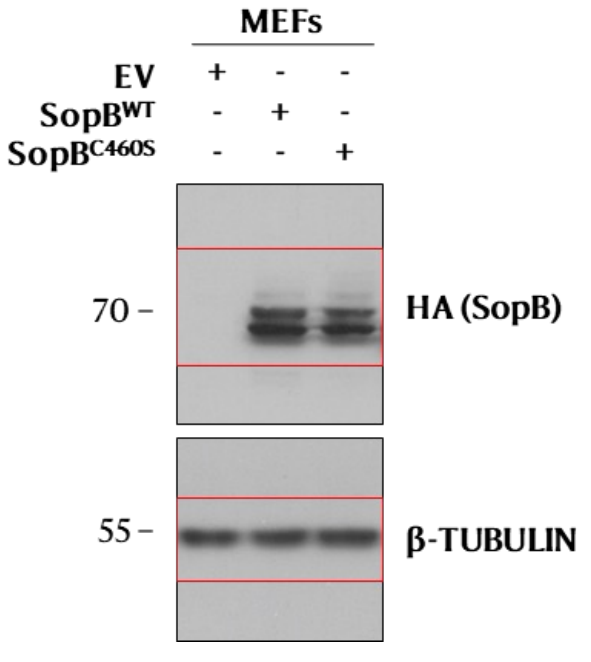

Extended Data Fig. 1 | The ESCRT machinery prevents TNF-induced RIPK1 kinase-independent apoptosis independently of the known cell death checkpoints

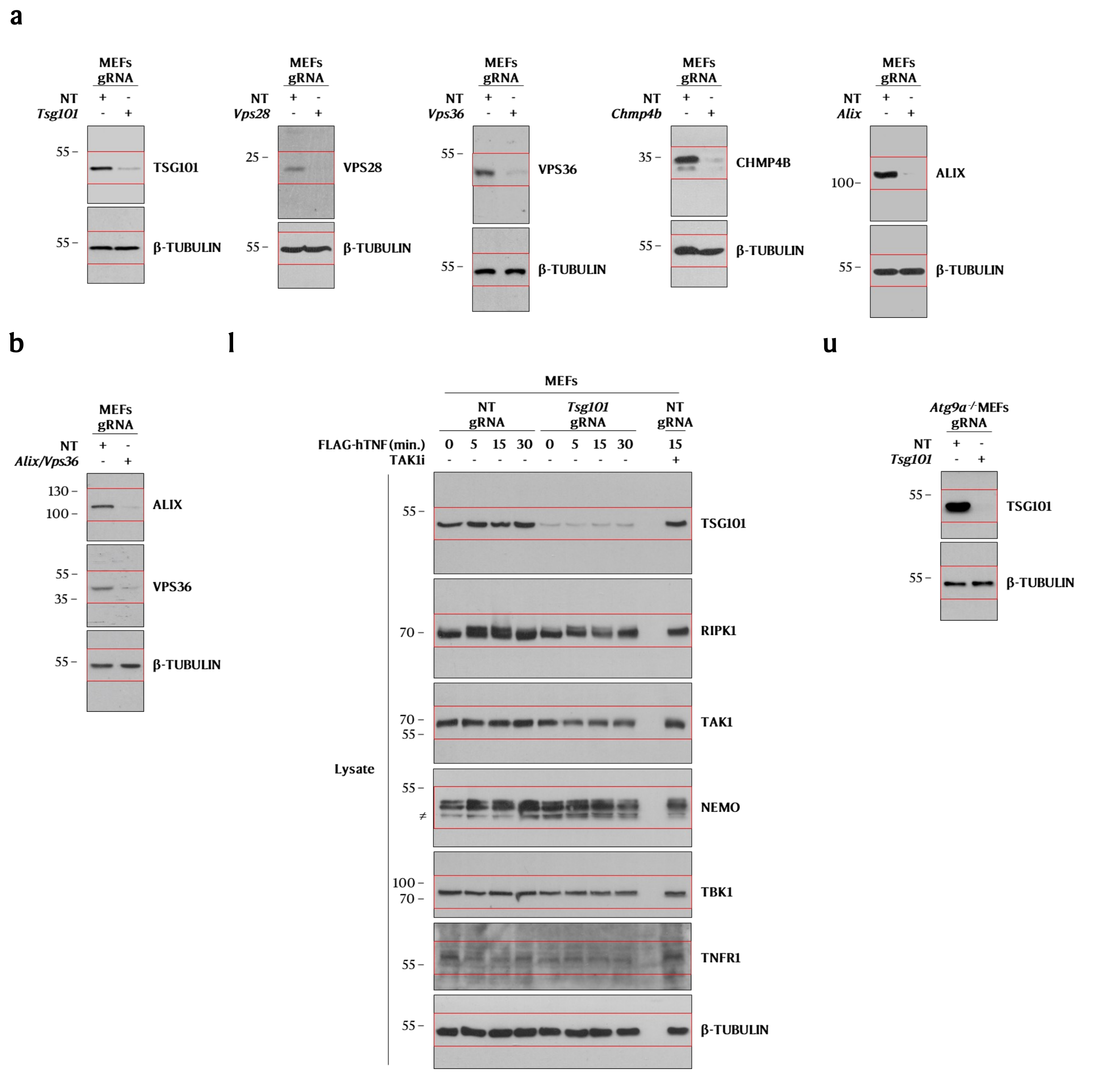

Extended Data Fig. 2 | Endosomal ILV formation prevents TNF-induced RIPK1 kinase-independent apoptosis

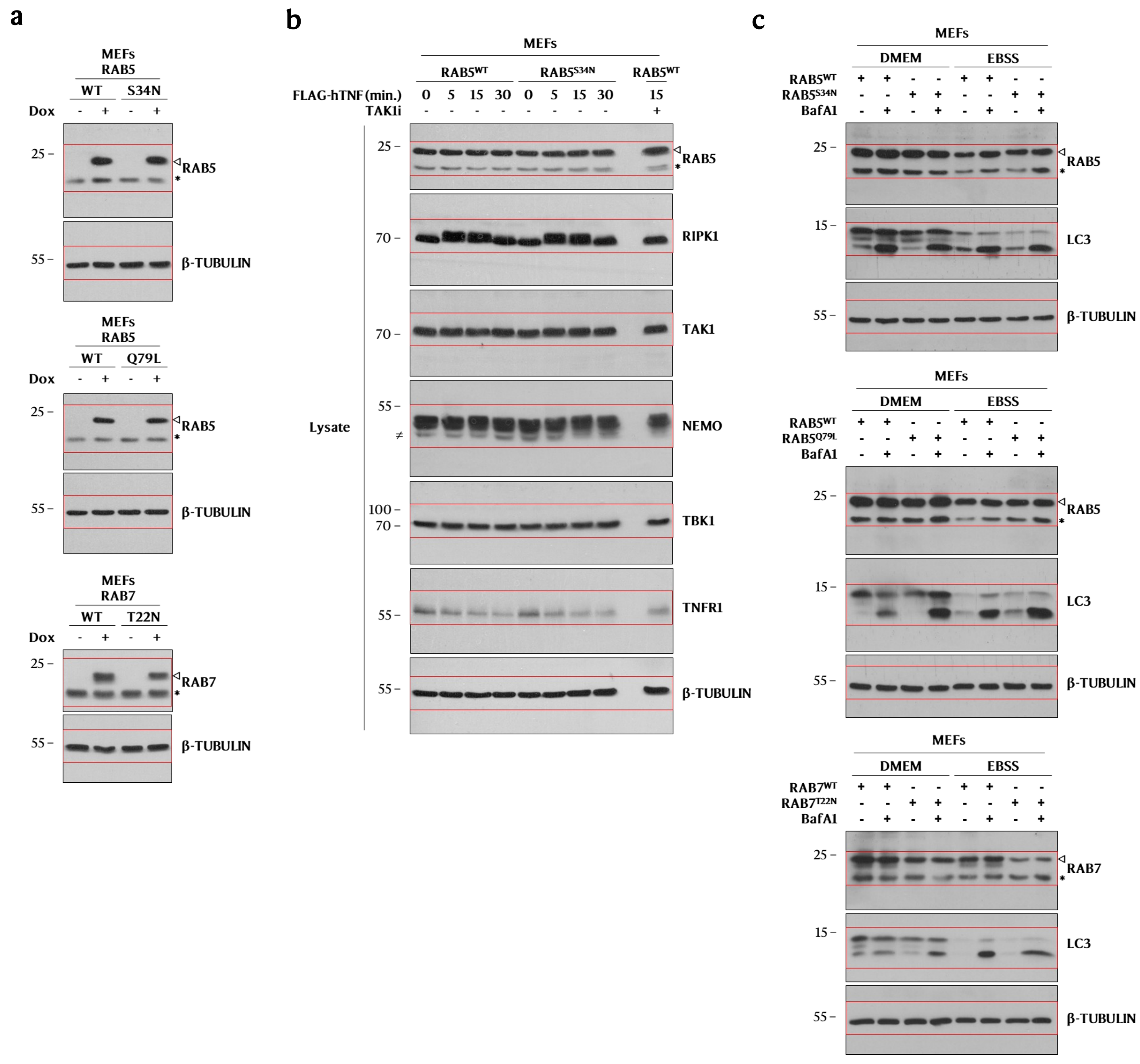

Extended Data Fig. 3 | ILV formation protects against TNF cytotoxicity independently of TNFR1 trafficking

a

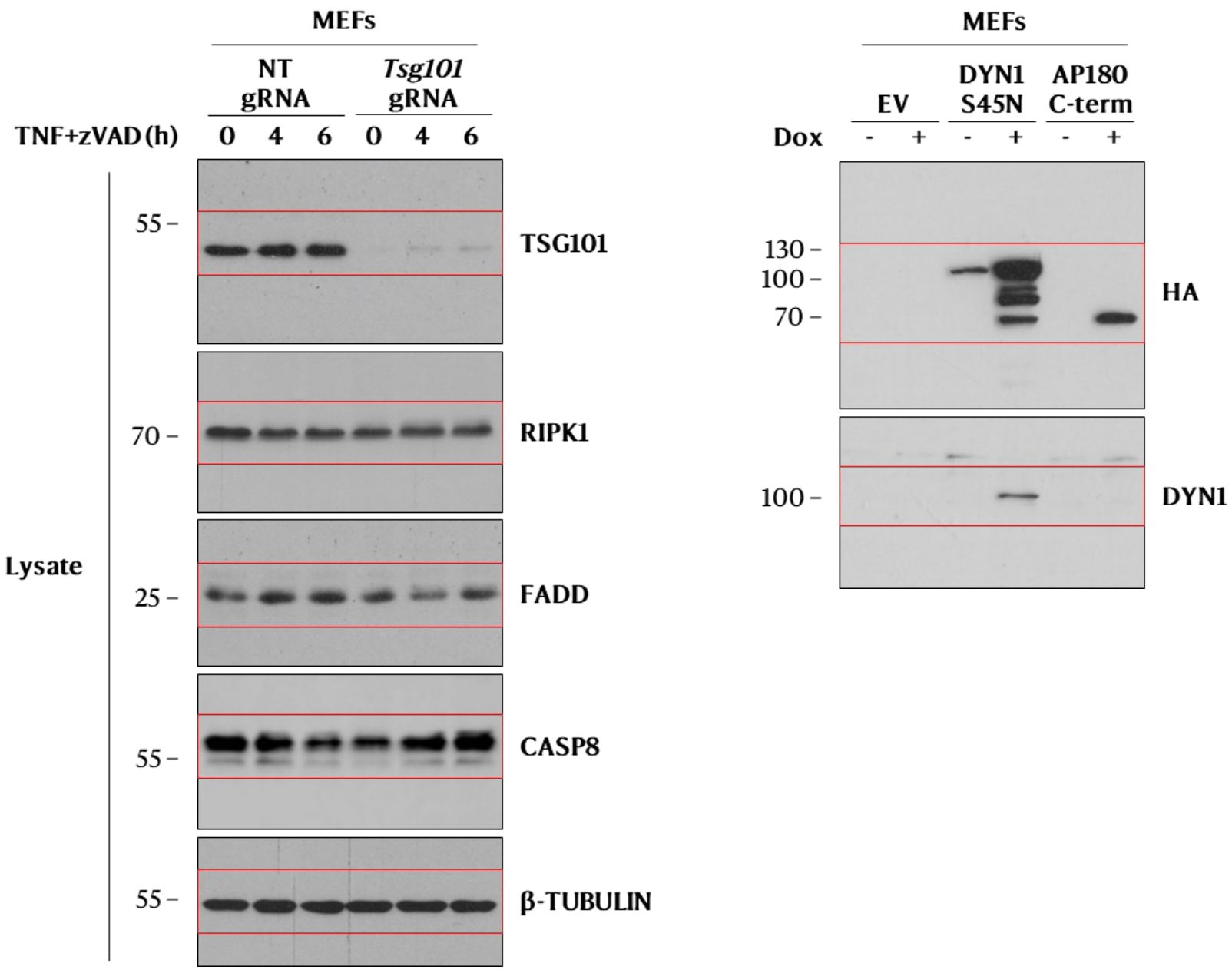

e

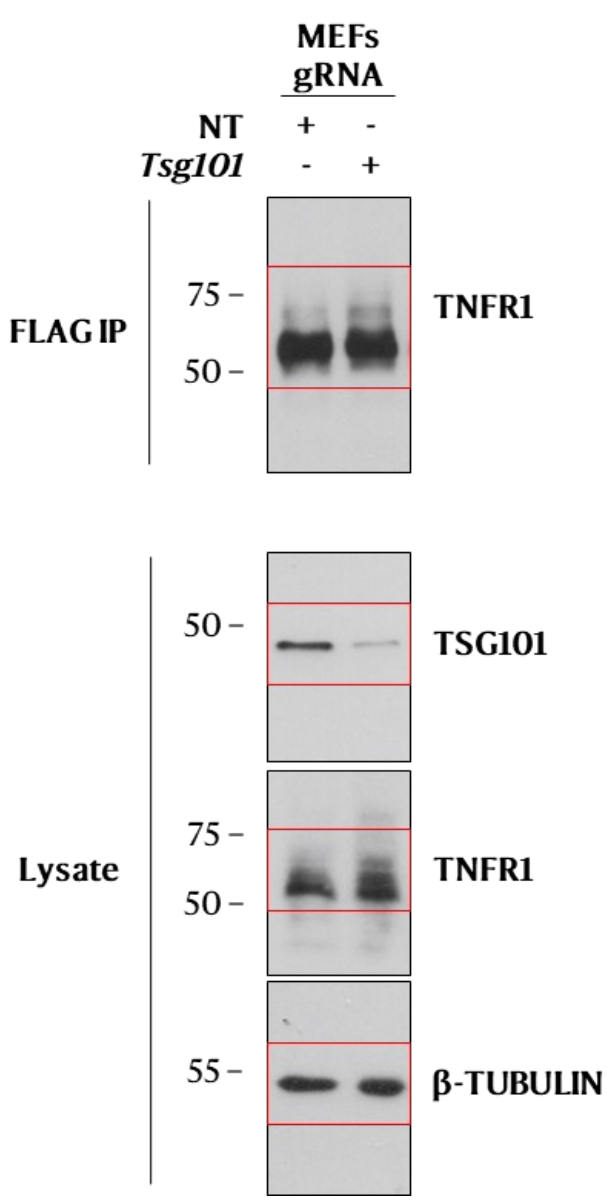

f

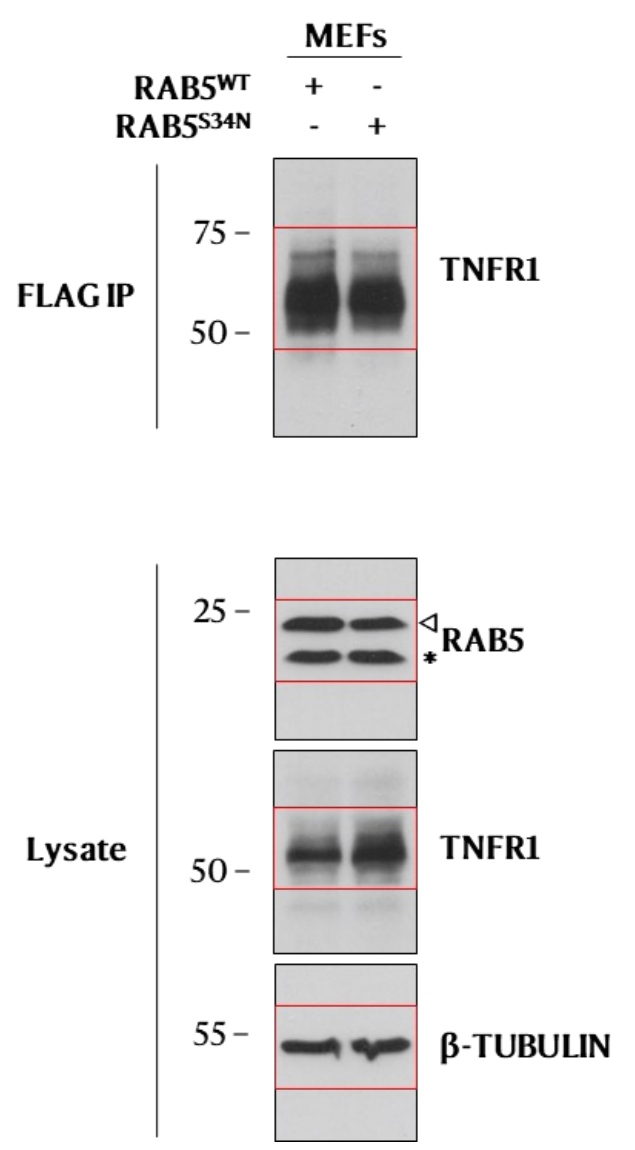

g

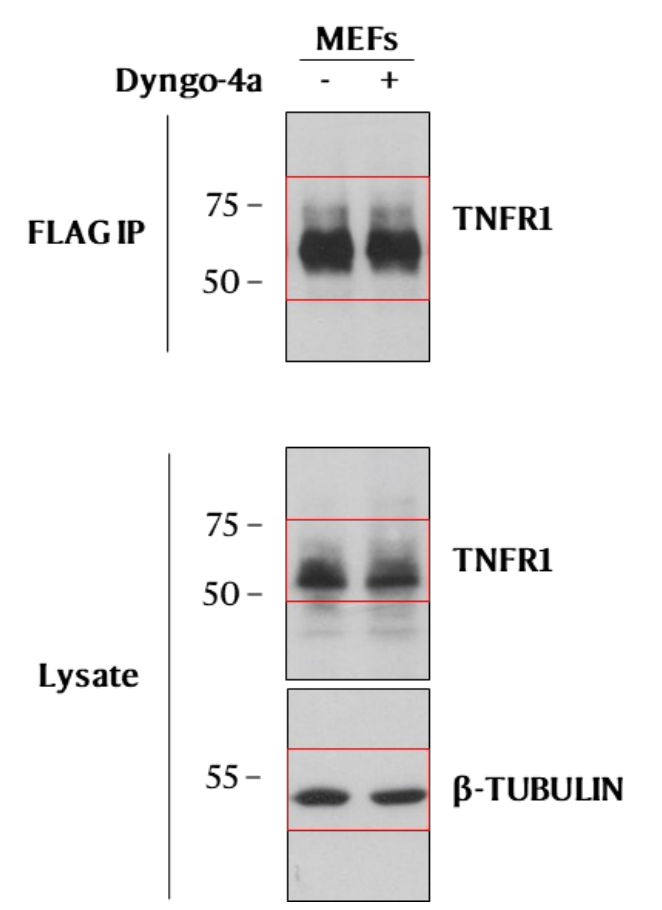

Extended Data Fig. 3 | ILV formation protects against TNF cytotoxicity independently of TNFR1 trafficking

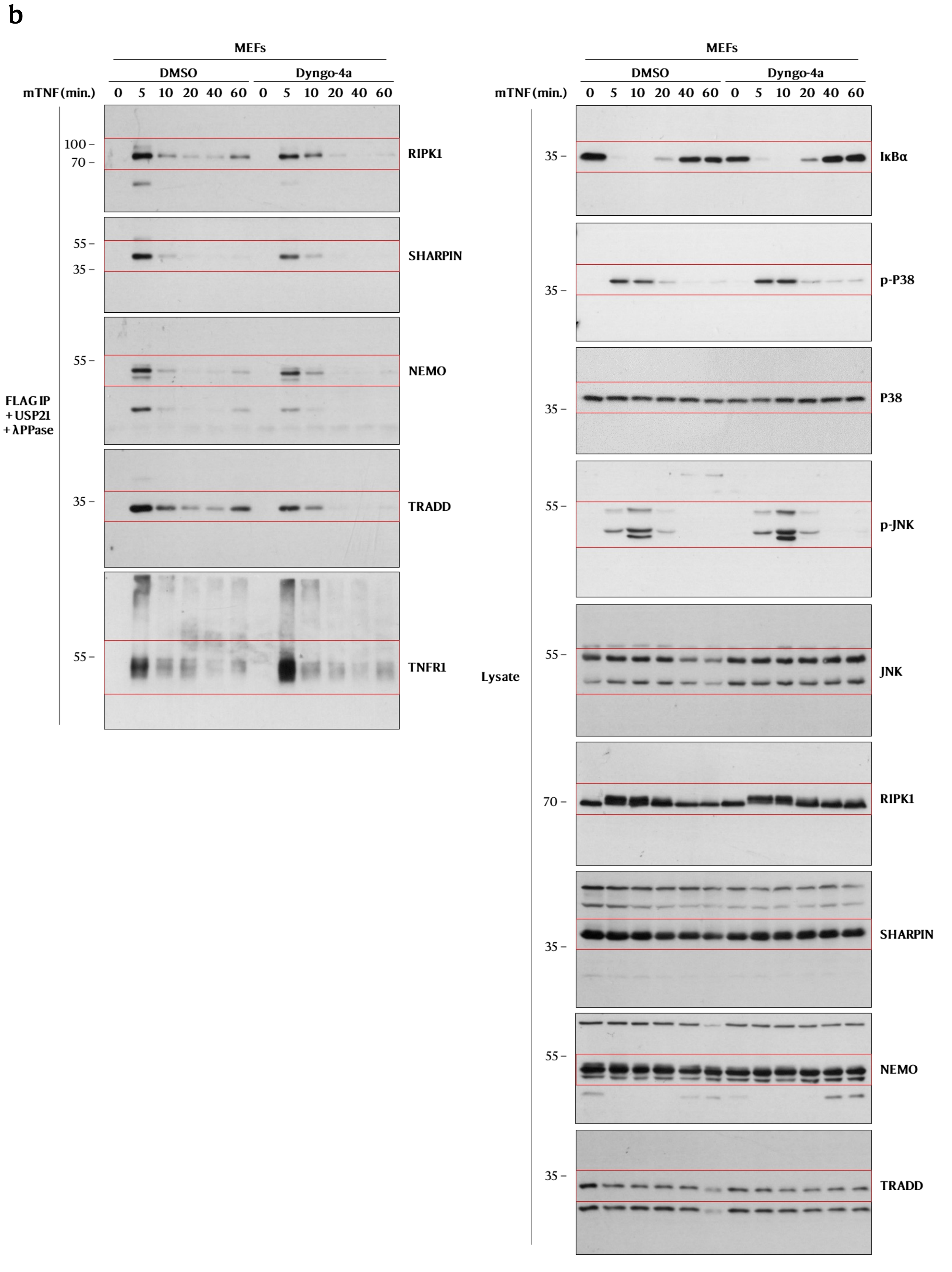

Extended Data Fig. 4 | Targeting of activated CASP8 into ILVs prevents TNF-induced cytotoxicity

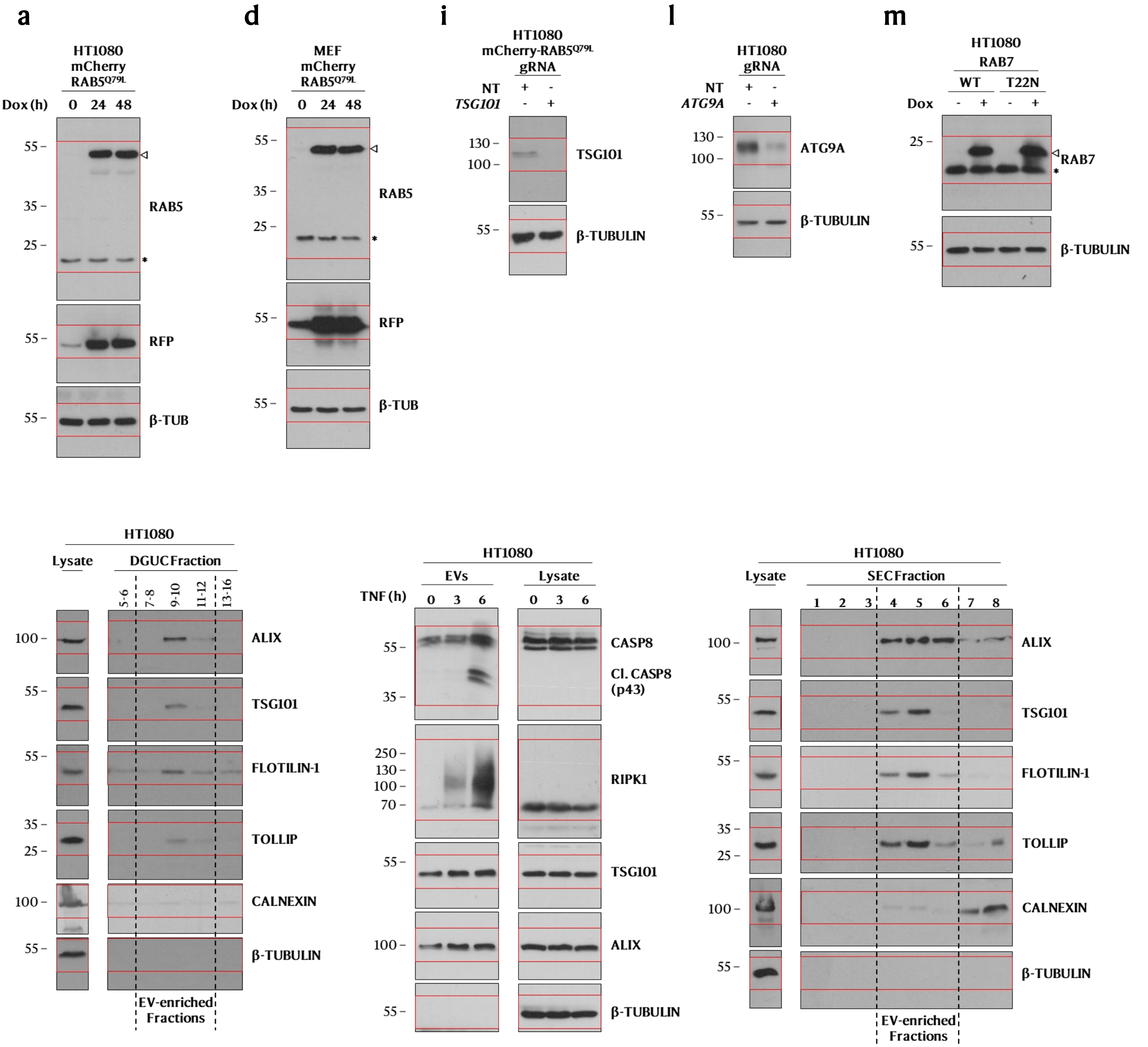

Extended Data Fig. 4 | Targeting of activated CASP8 into ILVs prevents TNF-induced cytotoxicity

q

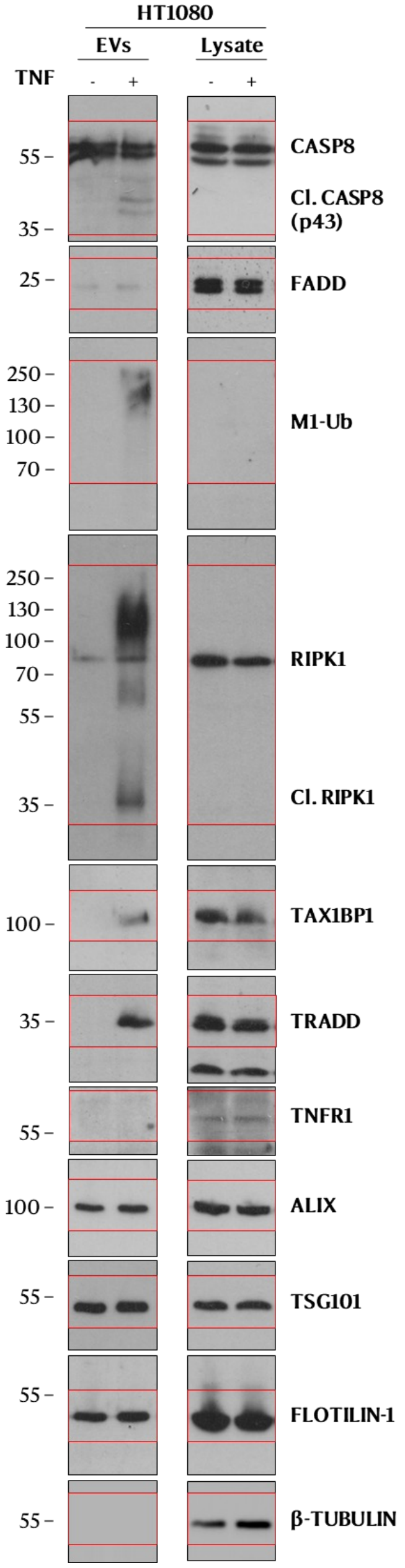

r

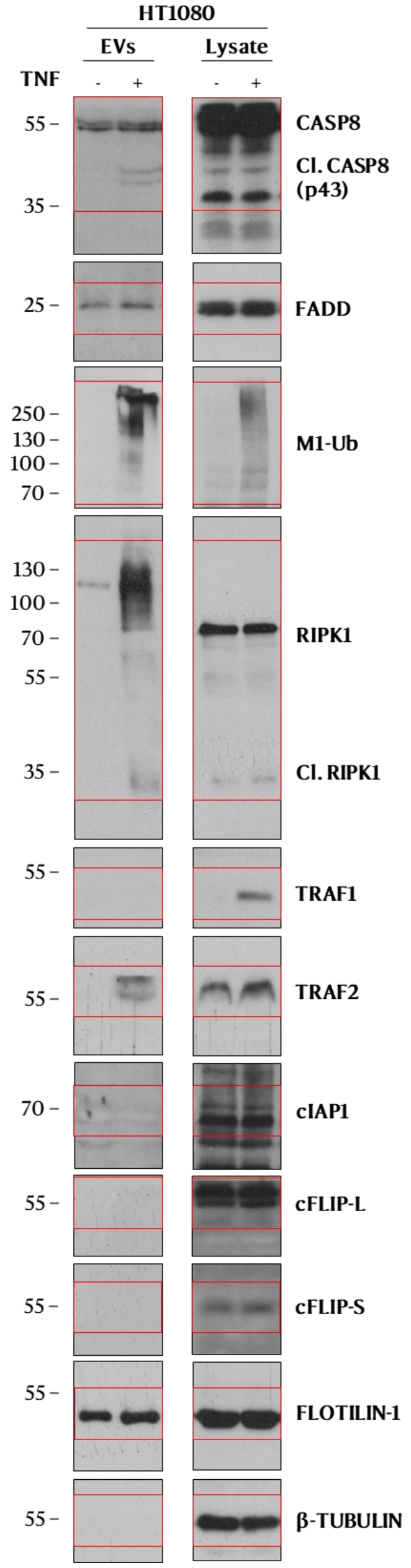

t

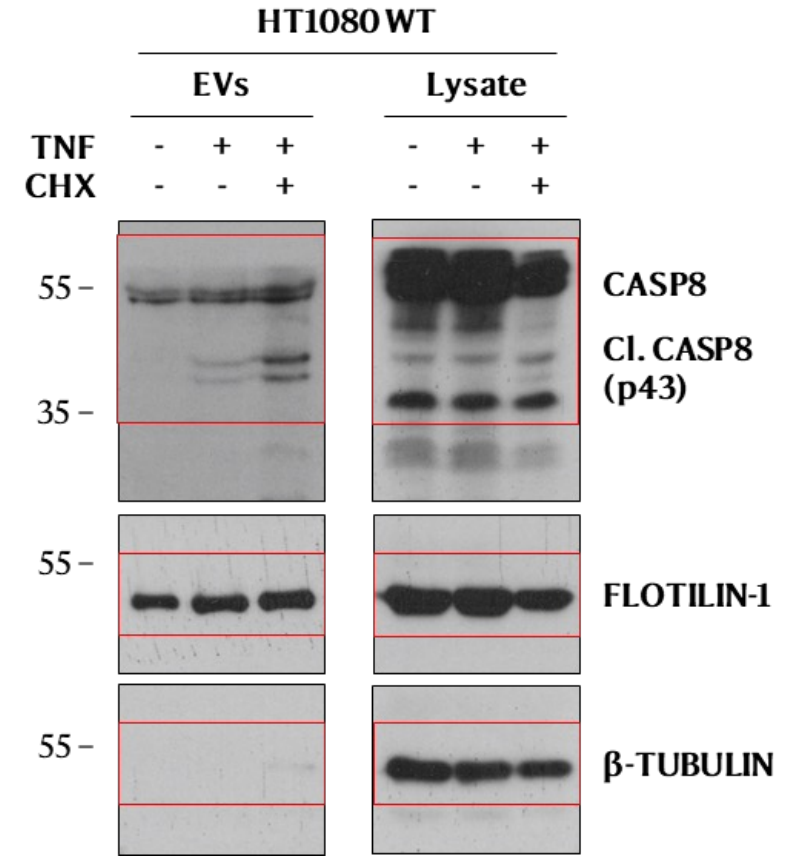

v

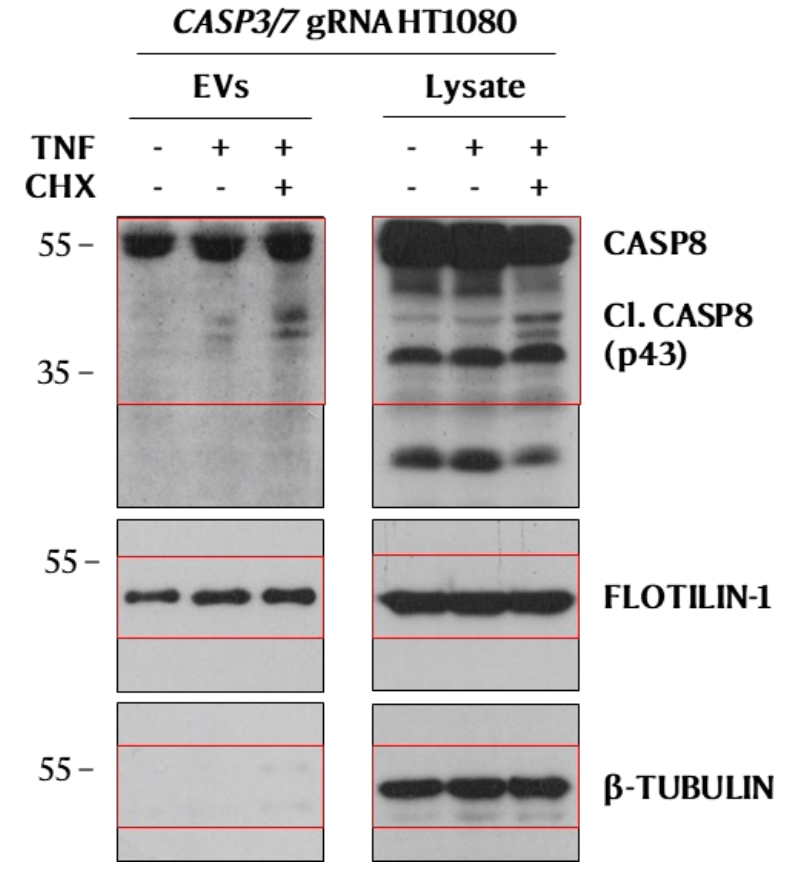

Extended Data Fig. 5 | Linear ubiquitination of RIPK1 promotes the TAX1BP1- and HSC70-dependent targeting of activated CASP8 into ILVs

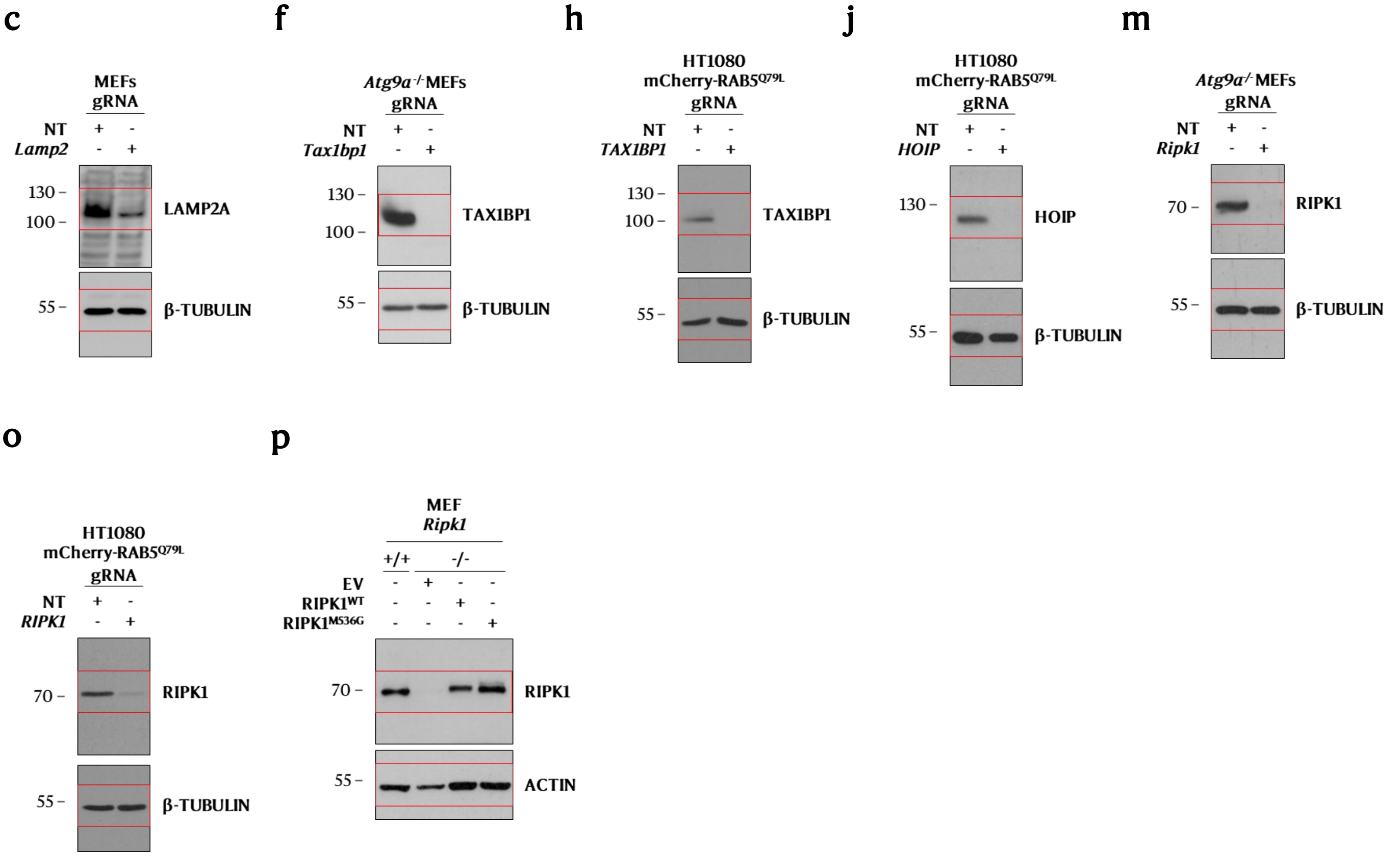

Extended Data Fig. 6 | Microbial effector proteins shift the TNF response from life to death by interfering with the ILV-dependent detoxification of activated CASP8

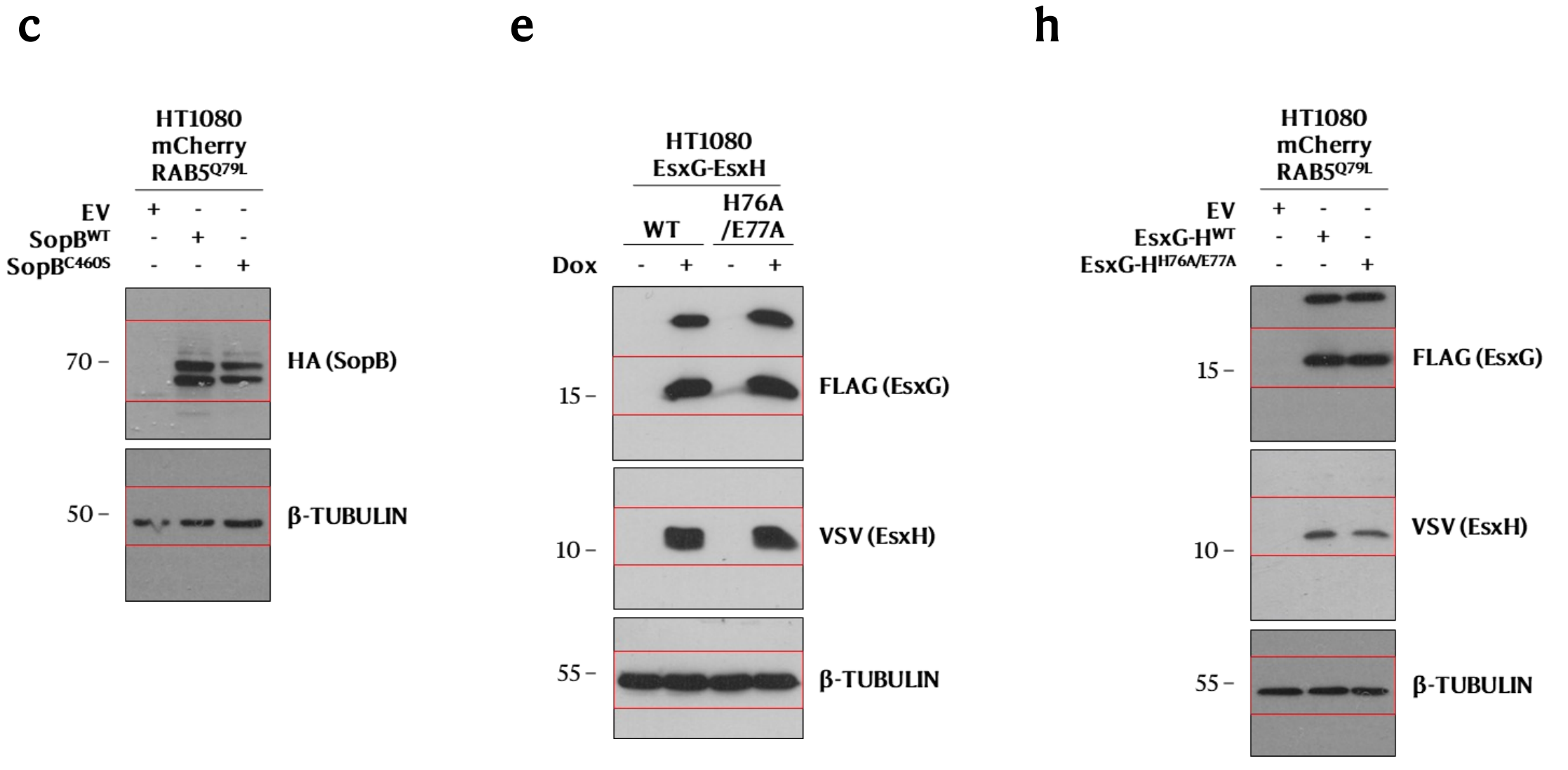

Supplement: Supplementary file 4 [file 41467_2026_75544_MOESM5_ESM.pdf]
